# Design, Synthesis, and Biological Evaluation of Mono- and Diamino-Substituted Squaramide Derivatives as Potent Inhibitors of Mycobacterial Adenosine Triphosphate (ATP) Synthase

**DOI:** 10.1021/acs.jmedchem.5c02284

**Published:** 2025-11-24

**Authors:** Paul R. Palme, Shipra Grover, Rana Abdelaziz, Lea Mann, Andreas M. Kany, Lina Ouologuem, Karin Bartel, Lindsay Sonnenkalb, Norbert Reiling, Anna K. H. Hirsch, Dirk Schnappinger, John L. Rubinstein, Peter Imming, Adrian Richter

**Affiliations:** 1 Pharmaceutical Chemistry, Institute of Pharmacy, 9176 Martin-Luther-Universität Halle-Wittenberg, Halle (Saale) 06120, Germany; 2 Department of Microbiology and Immunology, 12295 Weill Cornell Medical College, New York, New York 10065, United States; 3 Molecular Medicine Program, 7979 The Hospital for Sick Children, Toronto, ON M5G1H3, Canada; 4 Helmholtz Institute for Pharmaceutical Research Saarland (HIPS)−Helmholtz Centre for Infection Research (HZI), Saarbrücken 66123, Germany; 5 PharmaScienceHub, Saarbrücken 66123, Germany; 6 Department of Pharmacy, Faculty of Chemistry and Pharmacy, 9183 Ludwig Maximilian University of Munich, Munich 81377, Germany; 7 Molecular and Experimental Mycobacteriology, Research Center Borstel, Leibniz Lung Center, Borstel 23845, Germany; 8 Microbial Interface Biology, Research Center Borstel, Leibniz Lung Center, Borstel 23845, Germany; 9 Department of Pharmacy, Campus E8.1, Saarland University, Saarbrücken 66123, Germany; 10 Departments of Biochemistry and Medical Biophysics, The University of Toronto, Toronto, ON M5G 1L7, Canada; 11 German Center for Infection Research, DZIF, Partner Site Hamburg-Lübeck-Borstel-Riems, Borstel 23845, Germany

## Abstract

Amides of squaric acid are new drug candidates with activity against mycobacteria. Like the approved drug bedaquiline, these compounds achieve efficacy by inhibiting mycobacterial ATP synthase. However, squaramides have a different binding site than bedaquiline and possess the potential to inhibit bedaquiline-resistant strains. We developed an optimized synthesis for monoamino-substituted squaric acid analogues. Guided by an atomic model of a squaramide compound bound to its target, we synthesized 31 new monoamino/diamino-substituted squaric acid derivates. The efficacy of these compounds was determined in whole-cell assays against *Mycobacterium tuberculosis* and *Mycobacterium avium*. The molecular target was confirmed with measurement of inhibition of *Mycobacterium smegmatis* ATP synthase and by using *M. tuberculosis* strains that modulate the expression of ATP synthase. Compared to earlier squaramides, several analogues demonstrated micromolar activity against *M. tuberculosis*, improved microsomal stability *in vitro*, and reduced cytotoxicity. These properties contribute to the preclinical development of this class of compound.

## Graphical abstract

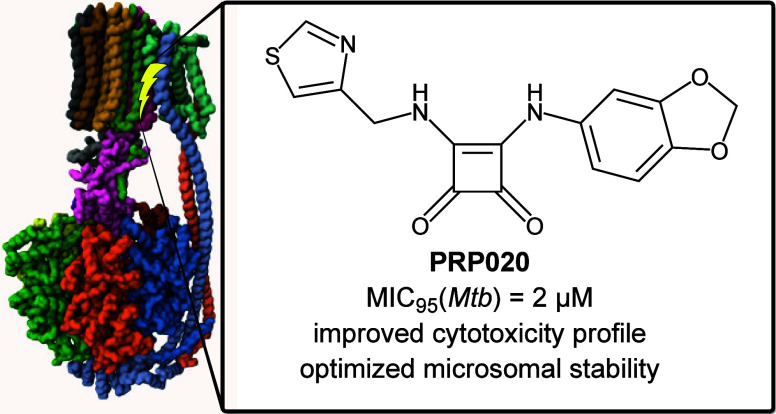

## 1 Introduction

Tuberculosis (TB) remains one of the most serious infectious diseases worldwide, claiming numerous lives, particularly in vulnerable populations. TB is caused by infection with *Mycobacterium tuberculosis* (*Mtb*) and is usually fatal unless treated with antimycobacterial drugs. The latest WHO TB report estimates 8.2 million new cases and 1.25 million deaths in 2023.^1^ *Mtb* most commonly infects the lungs, causing pulmonary TB, but can also cause extrapulmonary disease.^2,3^ Approximately 85% of patients infected with drug-susceptible TB can be cured with an optimized 4-month (rifapentine, isoniazid, pyrazinamide, and moxifloxacin) or 6-month (rifampicin, isoniazid, pyrazinamide, and ethambutol) treatment regimen.^4^ However, misuse and incomplete use of first-line drugs, which is exacerbated by side effects, long treatment regimes, and limited access to diagnosis and treatment in under-resourced communities, have led to the emergence of drug-resistant strains.^5–7^ Multidrug-resistant (MDR) and extensively drug-resistant (XDR) TB strains pose a major challenge for treatment.^8^ Infection with nontuberculous mycobacteria (NTM) can be even more difficult to treat. In contrast to *Mtb*, which is primarily confined to humans, NTM like *Mycobacterium avium* (*Mav*) and *Mycobacterium abscessus* (*Mabs*) are ubiquitous in the environment. These opportunistic pathogens cause pulmonary infections in patients with structural lung disease^9^ and are typically treated with multiple antibiotics for at least a year until sputum cultures remain negative for 12 months. Drugs used to treat NTM include injectable agents, such as aminoglycosides, which can have serious side effects,^10^ and antibiotics that can interact pharmacologically with drugs used to treat common comorbidities, leading to compliance issues. Even with strict adherence to established drug regimens, cure rates for NTM are generally worse than for MDR-TB and XDR-TB.^11^ For example, there is no reliable cure for *M. abscessus* pulmonary disease.^12^ NTM pulmonary disease and drug-resistant TB require development of antimycobacterial drugs with new molecular scaffolds.

The antimycobacterial drug bedaquiline (**BDQ**) was first described in 2005.^13^ **BDQ** was developed using a phenotypic screen against *Mycobacterium smegmatis* (*Msmeg*), followed by chemical optimization of a lead compound identified in the screen. *Msmeg* is a fast-growing nonpathogenic model for *Mtb.* The target of **BDQ** was determined to be mycobacterial adenosine triphosphate (ATP) synthase, an enzyme that catalyzes the efficient production of ATP and is essential in obligate aerobic organisms like mycobacteria. ATP synthase is also essential in human mitochondria, and consequently species selectivity is a requirement for compounds that target it. **BDQ** is effective against both drug-susceptible and MDR *Mtb*. It was approved by the US Food and Drug Administration (FDA) in 2012, making it the first new antimycobacterial drug approved in more than 40 years.^14^ However, this success is jeopardized by the rapid development and spread of **BDQ** resistance.^15^ **BDQ** also has potent antibacterial activity against several NTM species *in vitro* but not against most other bacteria, suggesting promise as a drug for the treatment of NTM infections.^16^ Unfortunately, **BDQ** showed poor clinical results against NTM, having no bactericidal effect against *Mabs* in patients.^17^ Nonetheless, **BDQ** established mycobacterial ATP synthase as a validated target for antimycobacterial drugs.

In 2017, a group at AstraZeneca performed a target-based screen of ∼900,000 compounds to detect *Mtb* ATP synthase inhibitors.^18^ This effort identified an active compound class based on the squaramide (SQA) scaffold. Subsequent lead optimization resulted in **SQ31f**, a specific and selective antimycobacterial ATP synthase inhibitor shown in Figure 1. **SQ31f** has a minimal inhibitory concentration (MIC_80_) of 0.5 μM against *Mtb* and undetectable cytotoxicity against human lung adenocarcinoma cells (A549). Importantly, **BDQ**-resistant mutants did not demonstrate cross-resistance to **SQ31f**.^18^ Subsequent work demonstrated potent inhibition of growth and ATP synthesis by **SQ31f** against various NTM (MIC_90_ in a range of 0.7 to 23.4 μM) and the isolate *Mabs Bamboo* (MIC_90_ of 16.6 μM).^19^ Furthermore, **SQ31f** complements the anti-*Mabs* activity of clofazimine, amikacin, clarithromycin, linezolid, rifabutin, and the oral combination tebipenem/avibactam, which target electron transport, transcription, protein synthesis, and cell wall formation, respectively. Similar drug combinations could allow for simultaneous inhibition of multiple cellular processes in NTM, which could reduce the potential for development of drug resistance and reduce inhibitor concentrations of the individual drugs within the cocktails, ultimately reducing toxicity and side effects.^19^ Our study is partly built on a publication by Li et al. (2020) concerning antimycobacterial SQAs: Diamino-substitution of the squaric acid scaffold resulted in **SQ6ab**, which had an MIC_90_ of 1.4 μM against *Mtb* and no cytotoxicity in Vero cells.^20^ Promisingly, **SQ6ab** exhibited improved metabolic stability compared to **SQ31f**, with no inhibition of the hERG channel and oral bioavailability in mice. However, the half-life of less than an hour is still low. While **SQ6ab** was presumed to inhibit ATP synthase of *Mtb*, its target was not confirmed.

**Figure 1:**
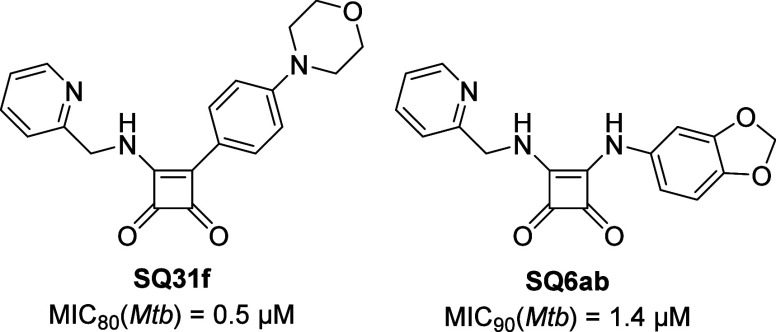
Published *Mtb* ATP synthesis inhibitors based on the squaramide scaffold.^18,20^

Here, we report a simplified synthetic procedure for the preparation of monoarylated SQAs such as **SQ31f**. We describe the structure-guided synthesis and characterization of promising new diamino-substituted SQAs. Characterization of *in vitro* activity against *Mtb* and NTM, microsomal stability, toxicity, and target validation show clear progress.

## 2 Results and Discussion

### 2.1 Rational Design of SQAs

We recently determined the structure of ATP synthase from *Msmeg* bound to **SQ31f**.^21^ This analysis enabled the construction of an atomic model showing how **SQ31f** interacts with a single site in the F_O_ region of the complex (Figure 2 A). The squaramide binds in a cavity corresponding to the cytosolic proton half-channel at the interface between subunits a and c. This half-channel, which allows protons to travel from the middle of the membrane to the cytosol, is thought to be filled with water in active ATP synthases.^22,23^ The nitrogen atoms of the picolyl group of **SQ31f** appear to form hydrogen bonds with Glu65 from subunit c, while the aromatic pyridine ring of **SQ31f** appears to form pi-stacking interactions with Phe69 and Tyr68 from the c subunit. The carbonyl groups of the squaramide scaffold form apparent hydrogen bonds with Arg188, Tyr240, and Ser184 from subunit a. The phenyl ring of the inhibitor also forms van der Waals interactions with Val61 and Phe58 from subunit c.^21^ The morpholine ring interacts with Asn174 and His166 of subunit a, with Asn174 forming an obvious hydrogen bond. Using this detailed insight into the binding site of **SQ31f**, we designed and prepared a library of SQA compounds. As shown in Figure 2B, we focused on replacing the pyridyl ring so that the new group was both a proton acceptor and an enlarged pi-electron system that can interact with Phe69 and Tyr68 from subunit c. The introduction of aniline derivatives instead of a direct aryl linkage increases the polarity of the molecule, and thus presumably its aqueous solubility, and allows for further interactions through hydrogen bonding. Active diamino SQA has already been described.^20^ In lieu of the morpholine ring, we introduced groups such as polar proton acceptors and halogens, which filled available space in the binding pocket but would likely not form additional interactions.

**Figure 2:**
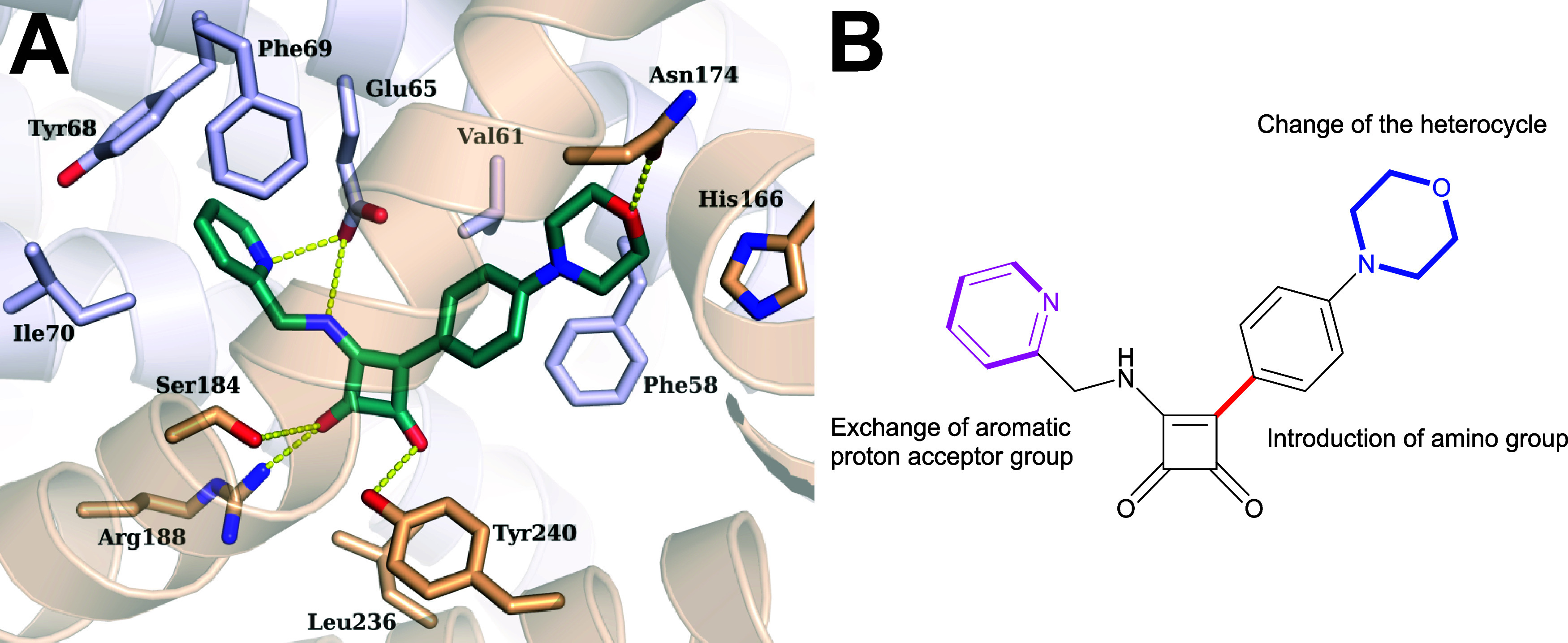
Inhibition of *Msmeg* ATP synthase by SQ31f. (A) Interaction between SQ31f (green) and subunits a (gold) and c (gray). Putative hydrogen bonds are shown as yellow dashed lines (PDB: 8G07). (B) Moieties subjected to change based on structural biology data.

### 2.2 Chemistry

The synthetic pathway for the target compounds **SQ31f** and **PRP001–004** is shown in Scheme 1. Commercially available 3,4-dihydroxycyclobut-3-ene-1,2-dione was converted to the corresponding dichloride with thionyl chloride and catalytic amounts of *N*,*N*-dimethylformamide (DMF). After evaporation of excess thionyl chloride, 3,4-dichlorocyclobut-3-ene-1,2-dione was washed with hot heptane and used without further purification.^24^ After addition of *N*-phenylmorpholine, this reaction was followed by a Lewis acid free Friedel–Crafts-like acylation at 85 °C in dry toluene for 4 h.^25^ The second chloride was substituted by the addition of methanol. To prevent formation of hydrochlorides, two equivalents of *N*,*N*-diisopropylethylamine (DIPEA) were added. This simplification of the arylation of the squaric acid dichloride with *N*-phenylmorpholine resulted in an increase in yield from 16% under standard Friedel–Crafts acylation conditions^18^ to 44%. In the final step, the methoxy group was exchanged with the desired amine by nucleophilic substitution. If the amine was present as a hydrochloride, then an equimolar amount of DIPEA was added. Precipitation in isopropanol-heptane and subsequent centrifugation with washing steps provided a simple and efficient way to obtain pure final compounds.

**Scheme 1:**
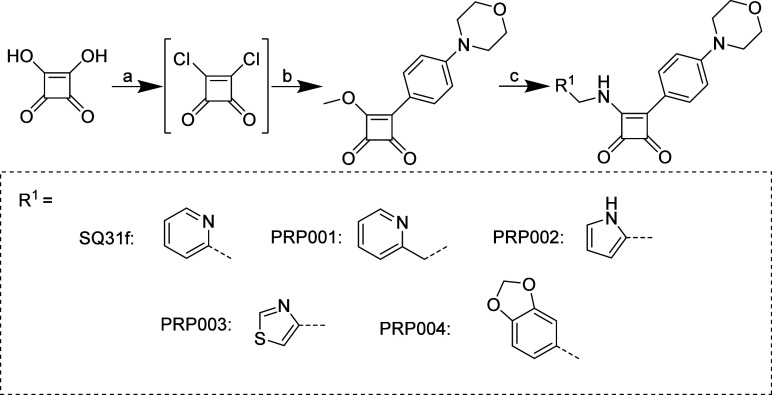
Syntheses of Target Compounds **SQ31f** and **PRP001**–**PRP004** ^a^

a Reagents and conditions: (a) thionyl chloride, DMF, 80 °C; (b) (1) *N*-phenylmorpholine, toluene, 85 °C, (2) methanol (MeOH), DIPEA, RT, yield 44%; (c) amine with R^1^, DIPEA (if hydrochloride is used), MeOH, RT, yield 61–85%.

Based on the previous work of Li et al. (2020), the synthesis of diamino-substituted compounds was planned, with an overview of the modifications depicted in Scheme 2.^20^ Commercially available 3,4-dimethoxycyclobut-3-ene-1,2-dione was dissolved in methanol, and the corresponding amine was added, leading to precipitation of the desired monosubstituted squaramide. If no precipitation was observed, then the nucleophilic substitution was monitored by TLC and the intermediate product was recovered by precipitation in isopropanol-heptane. The remaining methoxy group was exchanged with the desired amine by nucleophilic substitution in dry methanol at room temperature. If the amine was present as a hydrochloride, then equimolar amounts of DIPEA were added. The final SQA was obtained by precipitation in isopropanol-heptane with subsequent centrifugation and washing steps. Scheme 2 provides an overview of the substituents used for chemical modification.

**Scheme 2:**
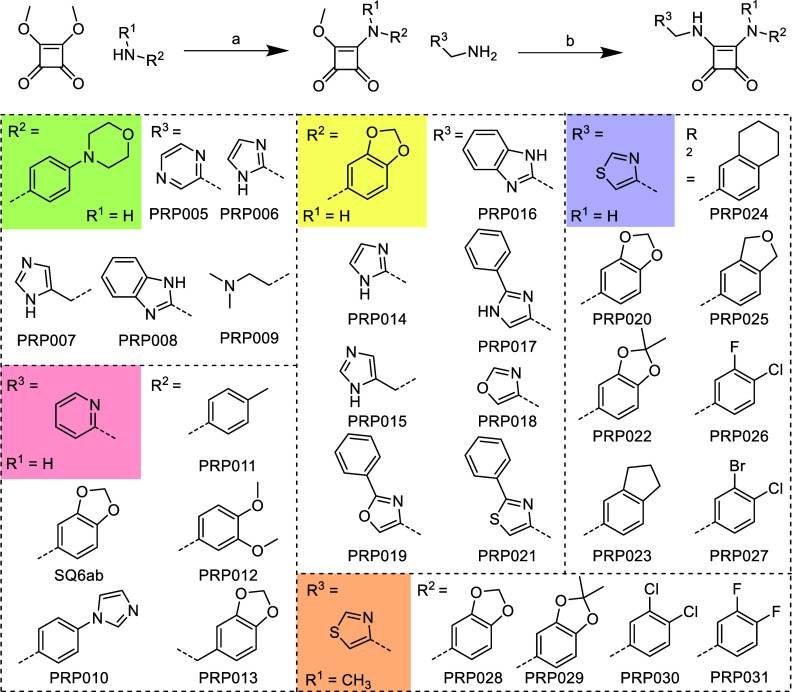
Syntheses of Target Compounds **SQ6ab** and **PRP005**–**PRP031** ^a^

a Reagents and conditions: (a) amine with R^1^/R^2^, DIPEA, (if HCl-salt is used), MeOH, RT, yield 44–92%; (b) amine with R^3^, DIPEA (if hydrochloride is used), MeOH, RT, yield 47–98%.

### 2.3 Inhibition of Mycobacterial ATP Synthase

To test if the SQA analogues that were synthesized inhibit mycobacterial ATP synthase, assays with *Msmeg* inverted membrane vesicles (IMVs) were performed.^29^ The comparison of F_O_ region sequences among mycobacterial species shown in Figure 3 revealed a high degree of similarity between *Msmeg* and the pathogenic mycobacteria *Mtb*, *Mabs*, and *Mav*. Because the binding pocket of **SQ31f** is conserved among mycobacterial species, the *Msmeg* enzyme is suitable for studying the effectiveness of SQA analogues enzyme inhibition assays. In these assays, the *Msmeg* strain GMC_MSM2,^30^ which has a genetically modified ATP synthase capable of functioning as an ATP-driven proton pump, is used to prepare IMVs.^21^ The IMVs are incubated with ATP, which results in acidification of the vesicles and fluorescence quenching of the fluorophore 9-amino-6-chloro-2-methoxyacridine (ACMA). Fluorescence recovery on addition of the H^+^/K^+^ antiporter nigericin allows precise measurement of the extent of acidification and therefore ATP synthase activity. Without an inhibitor, robust ATP synthase activity is detected in the assay (Figure 4, *gray—DMSO negative control*). In the presence of 10 μM of the ATP synthase inhibitors **BDQ**, **SQ31f**, or **SQ6ab**, proton pumping is inhibited (Figure 4, *green—positive controls*). Assays were then performed to test the activities of the squaramide analogues at 10 μM, with the extent of fluorescence recovery in each experiment normalized by the fluorescence recovery for the inhibitor-free control (Figure 4, *blue*). These results indicated that 18 compounds exhibited more than 50% inhibition of ATP synthase, with 15 compounds showing near complete inhibition.

**Figure 3:**
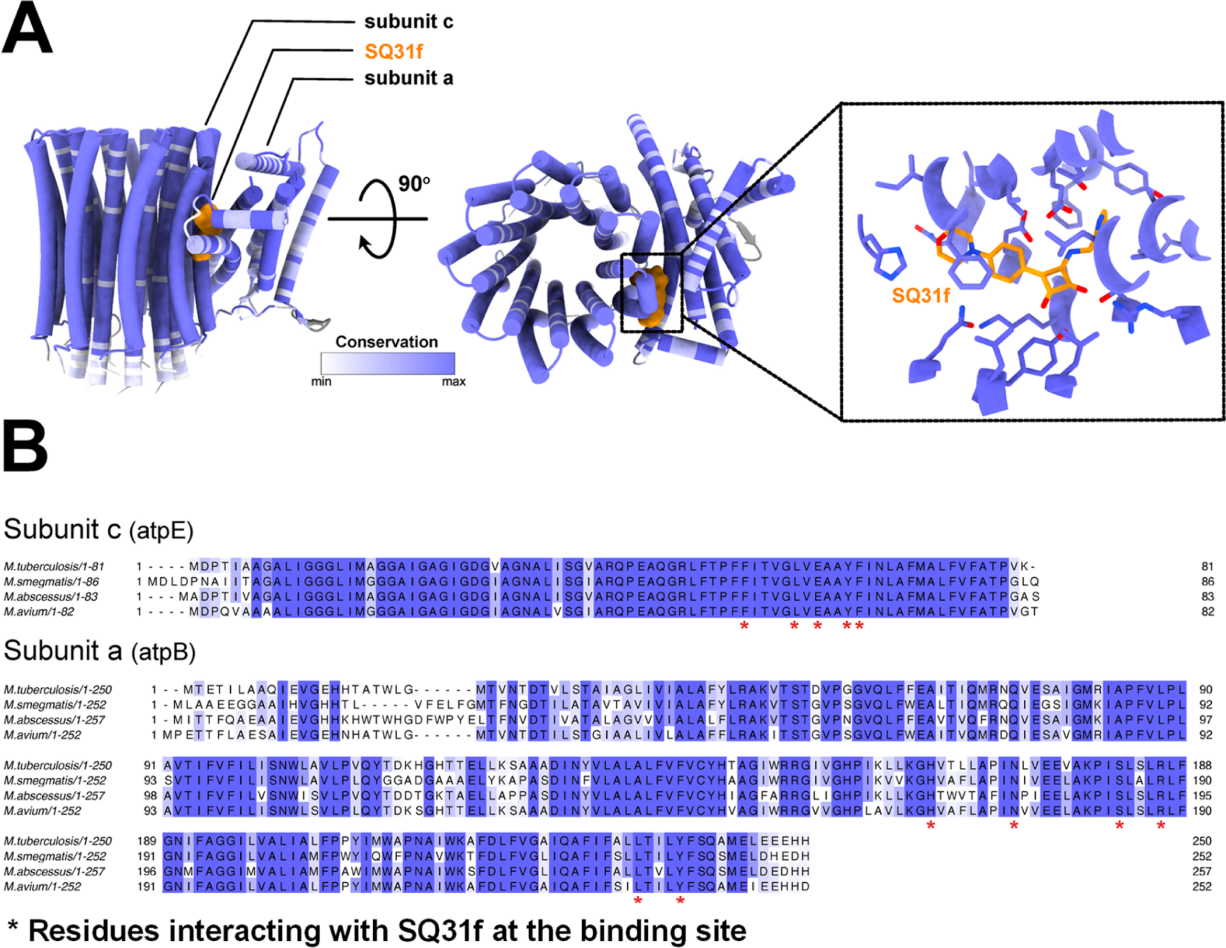
Similarity of the F_O_ region among mycobacterial species. (A) Atomic model of the F_O_ region from *Msmeg* bound to SQ31f (PDB: 8G07) colored by similarity between *Mtb*, *Mabs*, and *Mav*. This comparison shows that the F_O_ structure is highly conserved among the four species, and all amino acids in the binding pocket of SQ31f are identical. The figure was rendered with UCSF ChimeraX.^26^ (B) Multiple sequence alignment of ATP synthase subunits c (*atpE*) and a (*atpB*) from *Mtb*, *Msmeg*, *Mabs*, and *Mav*. Sequence identity is depicted in a gradient from white to dark blue, while amino acids interacting with SQ31f are indicated with asterisks. Sequences were aligned with Clustal omega^27^ and rendered with Jalview.^28^

**Figure 4:**
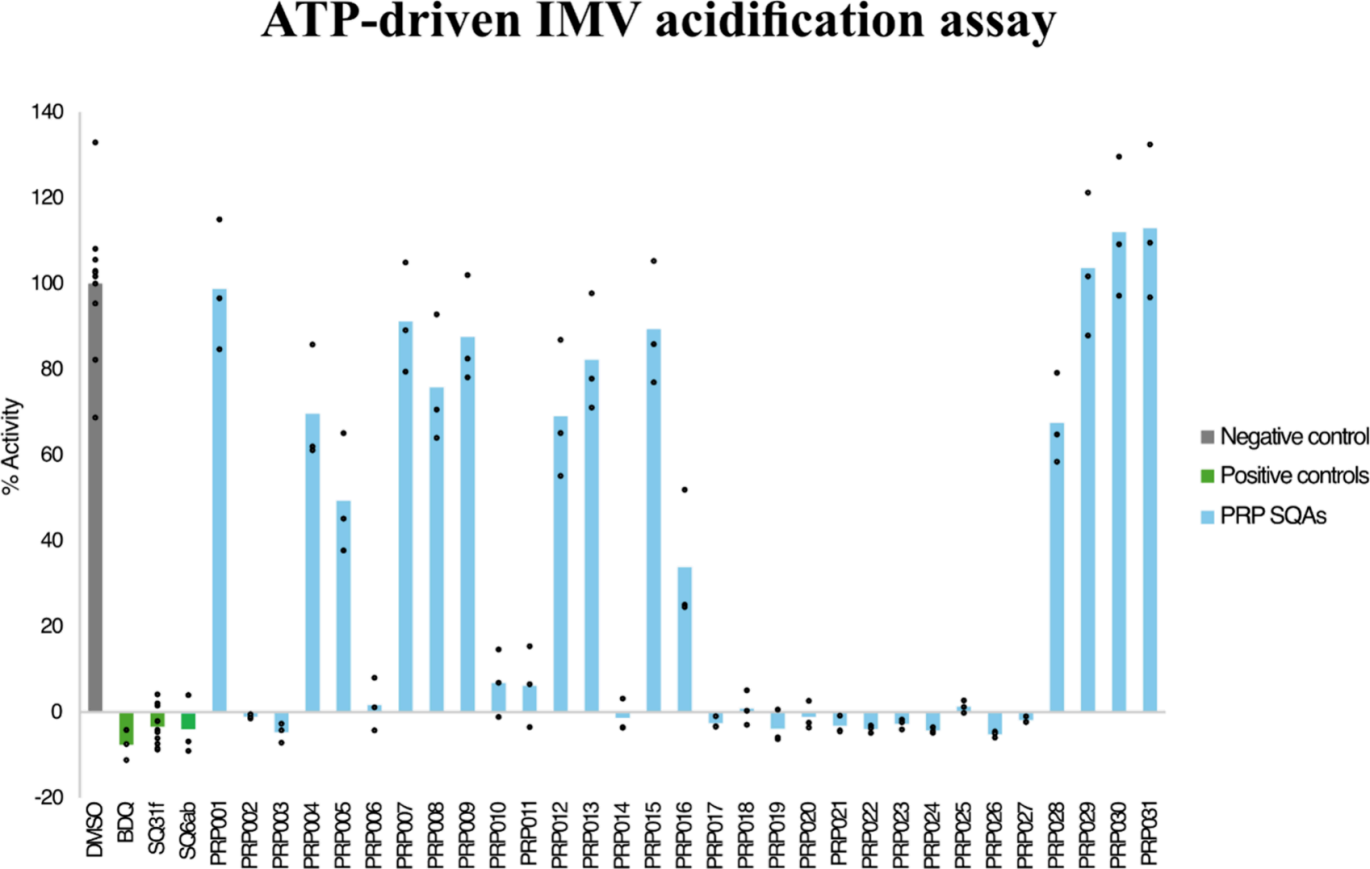
Screening SQAs at 10 μM for inhibition of ATP-driven IMV acidification. All compounds were screened for activity against *Msmeg* IMVs. DMSO (gray) was used as negative control. Of all novel SQAs tested (blue), 18 compounds showed >50% inhibition of IMV acidification, 15 of which showed ∼100% inhibition. SQ31f, SQ6ab, and BDQ (green) were used as controls. Results show mean ± standard deviation, from *n* = 10 (DMSO, SQ31f) and *n* = 3 (BDQ, SQ6ab, and PRP SQAs) independent assays using the same preparation of IMVs.

While the IMV assay can detect ATP synthase inhibitors,^29^ at high concentrations, some compounds, including **BDQ**, appear to nonselectively collapse the proton motive force (PMF) across vesicle membranes. This uncoupling behavior does not necessarily occur with the compounds in cells.^31^ To determine if the active compounds are selective ATP synthase inhibitors or simply disrupt the PMF, we tested the compounds in a succinate-driven IMV acidification assay also at 10 μM (Figure 5 A). Compounds **SQ6ab**, **PRP003**, **PRP006**, **PRP010**, **PRP011**, **PRP014**, **PRP018**, **PRP020**, **PRP022**, **PRP023**, and **PRP025** (see Tables 1 and 2 for structures) did not show inhibition of succinate-driven IMV acidification. **PRP002, PRP016**, **PRP019**, and **PRP024** showed ∼20–50% inhibition of succinate-driven IMV, similar to **BDQ** at 10 μM (Figure 5 A). Compounds **PRP017**, **PRP021**, **PRP026**, and **PRP027** exhibited >50% inhibition of succinate-driven IMV acidification, indicating that they might function by disrupting the transmembrane PMF (uncouplers, Figure 5 A). However, when tested for their ability to inhibit both ATP- and succinate-driven IMV acidification at a lower concentration (1 μM, Figure 5 B), **PRP017**, **PRP021**, and **PRP026** exhibited nearly complete inhibition (∼95–100% inhibition) of ATP-driven acidification but no inhibition of succinate-driven acidification, indicating that they are also specific ATP synthase inhibitors. At 1 μM, **PRP027** showed ∼60% inhibition of ATP-driven acidification and weak inhibition (∼20%) of succinate-driven acidification. These results show that all tested compounds are selective ATP synthase inhibitors, with only **PRP027** having uncoupling activity at a concentration close to the concentration at which it inhibits ATP synthase.

**Figure 5:**
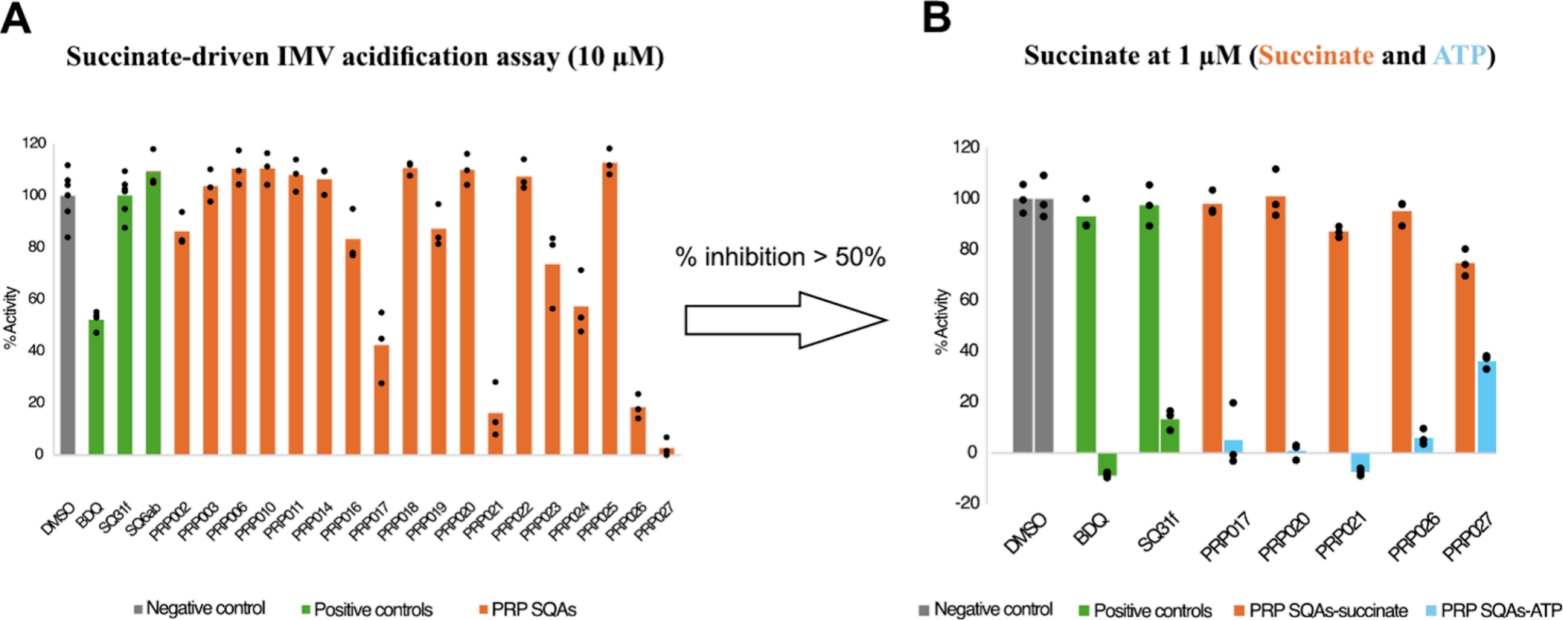
Screening SQAs in succinate-driven IMV acidification assays at 10 to 1 μM. (A) DMSO (gray) was used as negative control. SQ31f, SQ6ab, and BDQ (green) were used as controls. PRP SQAs tested in succinate-driven IMV acidification assays (orange) at 10 μM. PRP017, PRP021, PRP026, and PRP027 showed more activity than the BDQ control. (B) Testing PRP017, PRP021, PRP026, and PRP027 in ATP-driven (blue) and succinate-driven acidification assays (orange) at 1 μM. PRP017, PRP021, and PRP026 showed 95–100% inhibition of ATP-driven acidification, while PRP027 showed ∼60% inhibition. The negative control DMSO does not show inhibition of ATP- (right bar) or succinate-driven (left bar) IMV acidification, while BDQ and SQ31f show inhibition of ATP- (right bar) but not succinate-driven (left bar) IMV acidification. Results show mean ± standard deviation, from *n* = 3 separate experiments from the same preparation of IMVs.

**Table 1:**
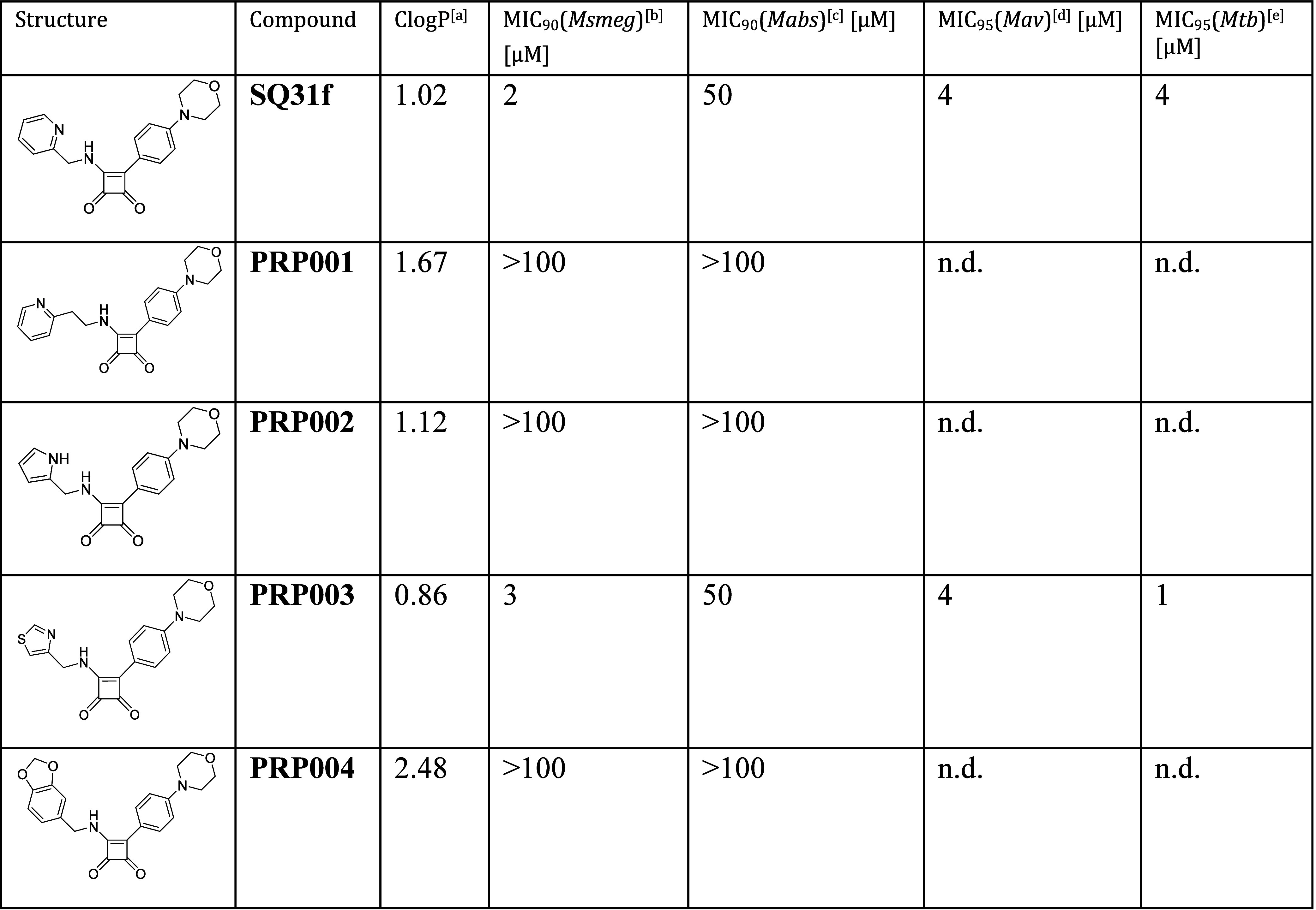
Antimycobacterial Activity of Monoamino SQA with ClogP (Compounds **PRP001**–**PRP004**)

a Calculated with ChemDraw Professional 15.1.

b *Msmeg mc* ^2^155.

c *Mabs* ATCC19977.

d *Mav* Chester 1901, ATCC 25291.

e *Mtb H37Rv* ATCC 27294. Performed in duplicate, results shown as average. Data were obtained via optical density (OD) measurements. For detailed information on the methodology of the assays, see Section 4.

**Table 2:**
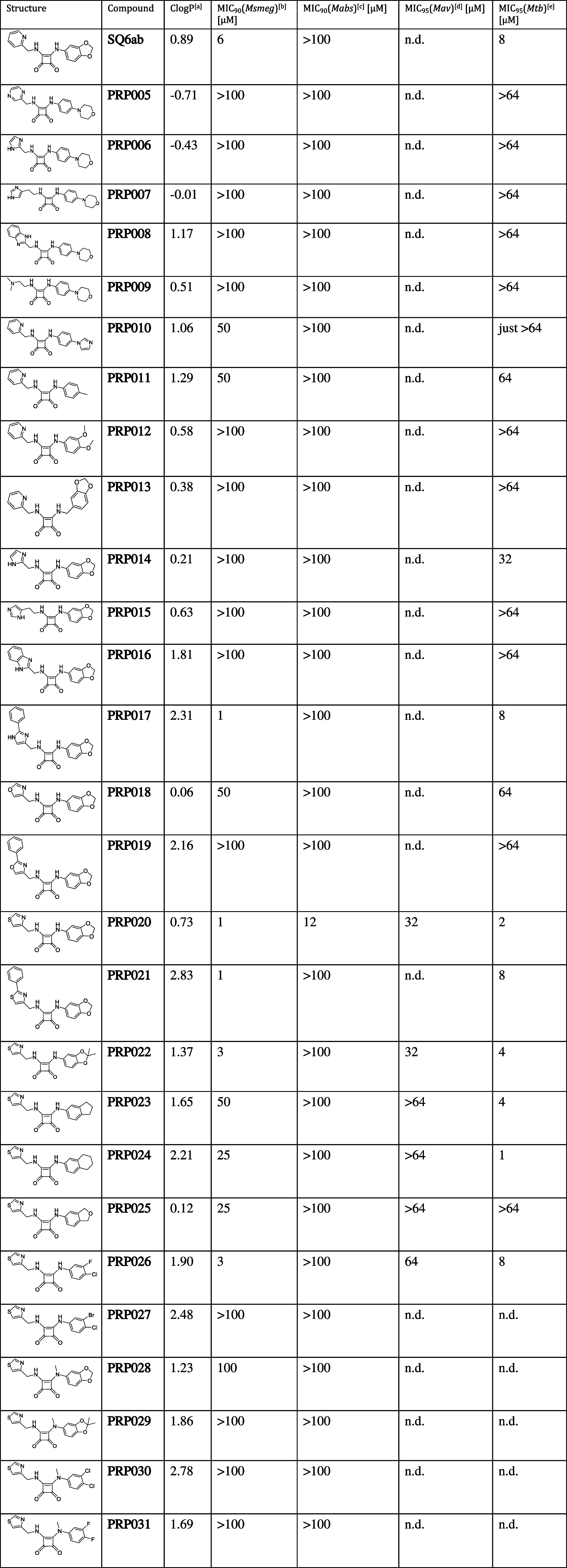
Antimycobacterial Activity of Diamino SQA with ClogP (Compounds **PRP005**–**PRP031**)

a Calculated with ChemDraw Professional 15.1.

b *Msmeg mc^2^* 155.

c *Mabs* ATCC19977.

d *Mav Chester 1901,* ATCC 25291.

e *Mtb H37Rv* ATCC 27294. Performed in duplicate, results were averaged. Data was obtained via OD measurements. For detailed information on the methodology of the assays, see Section 4.

### 2.4 Antimycobacterial Activity of SQA Analogues in Whole-Cell Assays

**SQ31f** was used as a reference for monoamino SQAs. Because the SAR was based on the cryo-EM structure of ATP synthase from *Msmeg*, assays measuring MICs for *Msmeg* were performed (Table 1, third column). If activity was detected, we performed further assays with *Mabs*, *Mav*, and *Mtb* (Table 1, columns 4 to 6). With **SQ31f**, we found a higher MIC for activity against *Mtb* than was previously described,^18^ although we determined a MIC_95_ (95% growth inhibition) rather than the MIC_80_ that was reported in the literature. However, when testing against *Mabs*, **SQ31f** had a MIC_90_ ∼5-fold higher than reported in the literature.^19^ Of four novel monoamino SQAs we prepared, only **PRP003** had an activity comparable to **SQ31f**. This observation suggested that the thiazol-5-ylmethanamine moiety is a promising substituent and showed that there is an alternative to the picolyl group. With **PRP004**, the heteroatom was removed from the aromatic ring system and instead proton acceptor groups are attached to a benzyl ring, leading to a complete loss of activity. This finding suggests that the proton acceptor group must be part of the aromatic system, which is consistent with the lack of activity of **PRP004** in the IMV acidification assay (Figure 4). A greater distance between the pyridine ring and Glu65 due to extension with a CH_2_ group, as in **PRP001**, also leads to a complete loss of activity in whole-cell and IMV acidification assays. Apart from a high activity in the IMV assay, it appears that (1*H*-pyrrol-2-yl)­methanamine is not a promising option, as **PRP002** proves in the whole-cell assay. Despite the poor activity against *Mabs*, the two active monoarylated SQAs show potent activity against *Mav*. These data underline the value of the substance class against slow-growing mycobacteria and indicate for the first time that **SQAs** are also promising drug candidates against the difficult-to-treat pathogen *Mav.*

**SQ6ab,** the optimized compound described by Li et al.,^20^ served as a reference substance for diamino SQAs (Table 2). We were able to reproduce antimycobacterial activity of **SQ6ab** against *Msmeg* and *Mtb* in these experiments; however, the determined MIC (8 μM) is higher than the value reported in the literature.^20^ Of the 27 new diamino SQAs, five showed MICs >20 μM and seven MICs <10 μM. Notably, **PRP020** demonstrated pronounced activity against all mycobacteria tested. Structurally, the aniline moiety of **PRP020** could form a pi–pi interaction with Phe58 from the c subunit of the target while accepting protons from Asn174 with its methylenedioxy group (see Figure 2). The thiazol-5-ylmethanamine moiety could be an even better interaction partner for Phe69 and Tyr68 than the picolylamine of **SQ6ab**. These hypotheses are supported by the results of the ATP-driven IMV acidification assays. **PRP021** showed activity similar to **SQ6ab**. In **PRP021**, the phenyl ring on the thiazole moiety provides an extension of the pi–electron system, which could interact with Phe69 and Tyr68. NTM showed no sensitivity to **PRP026**, suggesting that insertion of an amine between the squaric acid skeleton and the phenyl substituent results in loss of activity, regardless of the substitution on the other side, as seen in **PRP005** to **PRP009**. With **PRP005** and **PRP006** being the only active compounds in the IMV assay, *N*-morpholino aniline is not a promising substituent. By switching the morpholine ring to imidazole, activity in the IMV assay was detected and it was possible to regain activity in the whole-cell assay, shown by **PRP010**. The first attempt to find a better substitution option than the 1,3-benzodioxol-5-amine was unsuccessful, as shown by **PRP011**, **PRP012**, and **PRP013**. Of four different imidazole derivatives (**PRP014** to **PRP017**), only 4-aminomethyl-2-phenylimidazole proved to be a promising substituent, with **PRP017** having a strong inhibitory effect on growth of *Mtb* (MIC = 8 μM). We therefore assume that the expansion of the pi–electron system should not be with a rigid moiety. Interestingly, a switch to oxazole derivatives led to a clear loss of activity in the whole-cell assay while still being active in the IMV assay (**PRP018** and **PRP019**). Using thiazol-5-ylmethanamine as the substituent leads to active compounds (**PRP003** and **PRP020**). Therefore, in a second attempt we tried to find a substitution option better than 1,3-benzodioxol-5-amine. Replacing the oxygens of the acetal structure by methylene groups, as in **PRP023** and **PRP024**, reduced the activity in Msmeg, but these compounds continued to exhibit high activity against *Mtb* (MICs of 1 to 4 μM). The opposite effect was seen with the 1,3-dihydroisobenzofuran-5-amine substituent (**PRP025**), which showed activity against *Msmeg* but not against other mycobacteria. Halogen substituents that replaced the methylene dioxo group of the aniline had different effects on activity: No loss of activity was observed for **PRP026** (4-chloro-3-fluorophenyl amino analogue), while **PRP027** (3-bromo-4-chlorophenyl amino analogue) was inactive against *Msmeg*. One possible factor contributing to the loss of **PRP027** activity is its binding to the ATP synthase target: As shown in Figure 5B, **PRP026** is an effective inhibitor of ATP-driven IMV acidification at a concentration of 1 μM (∼100% inhibition detected), while **PRP027** achieves only ∼60% inhibition at this concentration. One possible explanation for this observation is that the larger bromine atom in PRP027 is poorly tolerated by the binding pocket. Two additional methyl groups on the methylenedioxy group of the aniline only minimally reduced activity (**PRP022**), which indicates somewhat restricted space in this part of the target. Methylation of aniline derivatives led to complete loss of activity in all assays, showing that proton donation and acceptance through the side chains are both crucial in drug target interaction (**PRP028** to **PRP031**). We found that diamino-substituted SQAs generally had no or very low activity against *Mabs* and *Mav*, despite high activity against *Mtb* and *Msmeg*. This observation suggests the existence of intrinsic resistance mechanisms of NTM against this compound class.

### 2.5 Toxicity

A selection of the SQAs described in this study were tested for cytotoxicity against two mammalian cell lines: murine monocytes (J774A.1 cells) and human bronchial epithelial cells (16HBE14σ). The previously described monoamino-substituted SQA **SQ31f** showed a reduction of cell viability in J774A.1 cells at both 10 and 100 μM, whereas **SQ6ab** showed cytotoxic effects only at 100 μM (Figure 6A). For the novel antimycobacterial SQAs, a different cytotoxicity behavior was observed: The compounds **PRP023** and **PRP026** showed toxic effects at 100 μM, whereas no toxicity could be observed at 10 μM. The other SQAs did not demonstrate a reduction of cell viability. A similar pattern was observed in 16HBE14σ cells, with **SQ6ab**, **PRP023**, **PRP026**, and **PRP003** having a toxic effect at higher concentrations, while the other compounds tested did not (Figure 6 B). In summary, the tested antimycobacterial SQAs did not show any pronounced cytotoxic effects.

**Figure 6:**
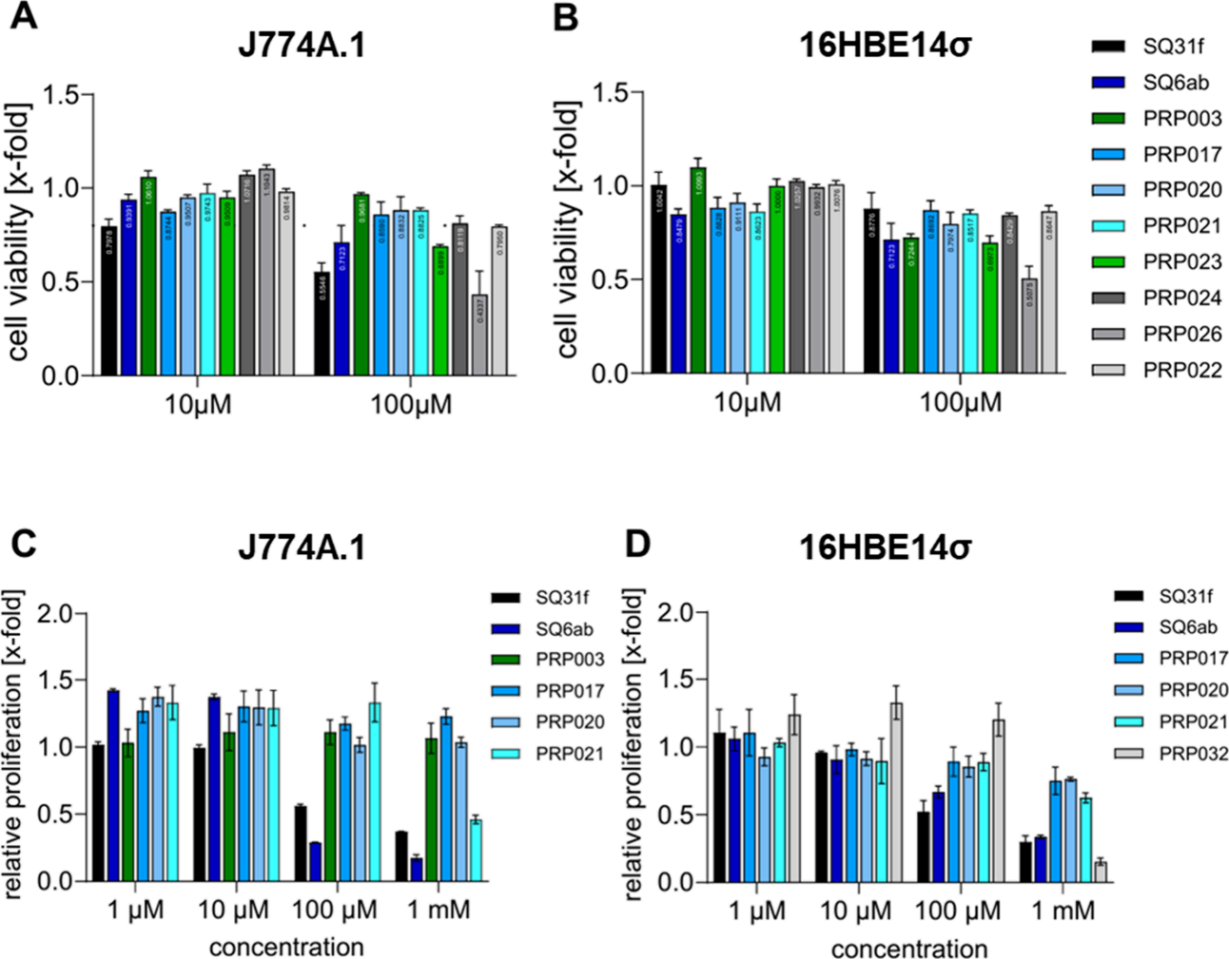
Cytotoxicity and antiproliferative effects of SQAs. (A, B) Acute toxicity—stimulation time: 24 h, readout: CellTiter-Blue-reagent against murine monocytes (J774A.1 cells) and human bronchial epithelial cells (16HBE14σ) at 10/100 μM. (C, D) Dose–response profiles—incubation time: 72 h, against murine monocytes (J774A.1 cells) and human bronchial epithelial cells (16HBE14σ). Color coding: SQ31f (black), SQ6ab (dark blue), PRP003 (dark green), PRP017 (cornflower blue), PRP020 (deep-sky blue), PRP021 (cyan), PRP023 (forest green), PRP024 (dark-slate gray), PRP026 (dim gray), PRP022 (cell viability)/PRP032 (relative proliferation) (light gray).

Apart from cytotoxicity experiments, the concentration-dependent influence of the substances on relative cell proliferation was investigated in murine monocytes (J774A.1 cells) and human bronchial epithelial cells (16HBE14σ) (Figure 6C,D). **SQ31f** and **SQ6ab** showed a clear negative effect on proliferation of J744A.1 cells at 100 μM, while the SQA analogues described in this study did not. Notably, **PRP003**, **PRP017**, and **PRP020** had no negative effect on cell proliferation even at 1 mM. With 16HBE14σ cells, **SQ31f** and **SQ6ab** showed an antiproliferative effect, with **PRP032** having a similar effect only at 1 mM, the highest concentration studied. These data demonstrate that several of the SQAs produced in this study, e.g., **PRP017**, **PRP020**, and **PRP021**, do not show the cytotoxic effects seen with **SQ31f** and **SQ6ab** in the cell types characterized.

### 2.6 Metabolic Stability

Previously published data indicated that the SQAs **SQ31f** and **SQ6ab** undergo rapid hepatic metabolism in mice. For **SQ31f**, sufficient plasma concentrations could only be achieved by coadministration with the cytochrome P450 inhibitor aminobenzotriazole (ABT).^18^ **SQ6ab** showed improved stability *in vitro* against hepatocytes and bioavailability *in vivo* in a mouse model without addition of ABT.^20^ However, the half-life was still too low for stringent *in vivo* experiments. Therefore, we considered improving metabolic stability as a main objective of the study to increase bioavailability. A selection of the most active SQAs was tested for microsomal stability compared to **SQ31f** and **SQ6ab**. **SQ31f** showed microsomal degradation (*t*_1/2_ = 45 min) too rapid for sufficient bioavailability, while **SQ6ab** had a promising half-life (*t*_1/2_ = 105 min). As shown in Table 3, compounds **PRP003**, **PRP017**, **PRP021**, **PRP022**, and **PRP027** did not show sufficient microsomal stability (*t*_1/2_ < 60 min). It is also observed that the derivatives **PRP023** and **PRP024**, which contain 2,3-dihydro-1*H*-indene and 5,6,7,8-tetrahydronaphthalene structures, respectively, have a short *t*_1/2_, indicating rapid metabolic attack on the saturated aliphatic moiety. **PRP020** was the most stable compound tested in the assay, with a half-life of >120 min and the lowest clearance. Therefore, **PRP020** has markedly better metabolic stability than the literature reference compounds.

**Table 3:** Microsomal Stability of SQAs against Mouse Liver Microsomes

compound	liver microsomes *t*_1/2_ [min]	Cl_int_ [μL/min/mg]
**SQ31f**	45 ± 19	37 ± 21
**SQ6ab**	105 ± 4.4	13 ± 0.6
**PRP003**	29 ± 11	55 ± 22
**PRP017**	51 ± 10	28 ± 5
**PRP020**	>120	<11.6
**PRP021**	26 ± 4	55 ± 9
**PRP022**	52 ± 17	29 ± 10
**PRP023**	3.0 ± 0.2	467 ± 30
**PRP024**	2.7 ± 0.1	510 ± 14
**PRP026**	49 ± 7	29 ± 5

### 2.7 Testing against **BDQ**-Resistant TB Strains

Table 4 lists the results of testing **SQ31f** and one of the new SQAs, **PRP020**, against **BDQ**-resistant *Mtb* strains. Due to the flat dose-inhibitory curves and for better comparability, MIC_95_ and MIC_80_ values are given. We observed an ∼8- to 16-fold increase in MIC for both compounds depending on the *atpE* mutant. **SQ31f** shows a strong increase in MIC in the *atpE* (subunit c) D28G mutant and a 4-fold increase in MIC in the A63P variant. Therefore, both **PRP020** and **SQ31f** appear to target *atpE*. However, in contrast to **SQ31f**, we see a higher MIC in the E61D mutant for **PRP020**. Mutants were selected based on high BDQ resistance and clinical relevance. BDQ interacts directly with residues E61 and A63, blocking the rotation of the ATP synthase subunit c, as observed by cryo-EM.^32^ Although residue D28 is not near the other residues in the primary sequence of subunit c, it is near to them in the three-dimensional structure of the folded protein and is hypothesized to affect drug binding at residue E61, which could explain the elevated MIC observed in both laboratory-selected and clinical strains.

**Table 4:** Antimycobacterial Activity of SQ31f and PRP020 against BDQ-Resistant TB Strains

strain	MIC_95_ **SQ31f** [μM]	MIC_80_ **SQ31f** [μM]	MIC_95_ **PRP020** [μM]	MIC_80_ **PRP020** [μM]
H37Rv_atpE_D28G	>64	64	>64	32
H37Rv_atpE_A63P	16	8	64	16
H37Rv_atpE_E61D	4	2	32	16
Rv0678 Q51R	16	8	8	2
Rv0678 R38stop	16	8	8	2
H37Rv_WT	4	4	2	2

With few exceptions, clinical resistance to BDQ is almost solely linked to mutations in *Rv0678*, which cause an increased expression of the MmpS5-MmpL5 efflux system.^33–35^ Therefore, we tested the activity of SQAs against BDQ strains harboring Rv0678 variants to evaluate potential cross-resistance. For these strains, a 4-fold increase in the MIC was observed for **SQ31f** and **PRP020.**

### 2.8 Target Validation

To confirm that modification of the **SQAs** did not alter their cellular target, we tested the compounds in a transcriptionally regulated *Mtb* strain (*Mtb atpDC-38%*) (Grover et al., manuscript in preparation). When grown without anhydrotetracycline (ATc), the strain expressed ATP synthase at 38% of the level expressed by wild-type (WT) *Mtb*. As expected, compared to WT, **SQ31f** was ≥7.5-fold more potent against *Mtb atpDC-38%* in absence of ATc but remained similarly potent to WT when ATP synthase expression of *atpDC-38%* was restored to that of WT *Mtb* by supplementing ATc at 600 ng/mL (Figure 7A). The change in potency described above was calculated as the ratio of MIC_50_ values for the different *Mtb* strains (0.089 μM/0.012 μM = 7.4). Next, using an *Msmeg* strain (*Msmeg* AtpSynOE) that harbored a chromosomal copy of ATc-inducible *Mtb* ATP synthase in addition to the native ATP synthase (Grover et al., manuscript in preparation), we demonstrated that adding ATc at 300 ng/mL reduced the potency of **SQ31f** by up to 4-fold relative to WT *Msmeg* (Figure 7B). In absence of ATc, **SQ31f** remained similarly potent against *Msmeg* AtpSynOE and WT Msmeg, thereby confirming ATP synthase as the target of **SQ31f.**

**Figure 7:**
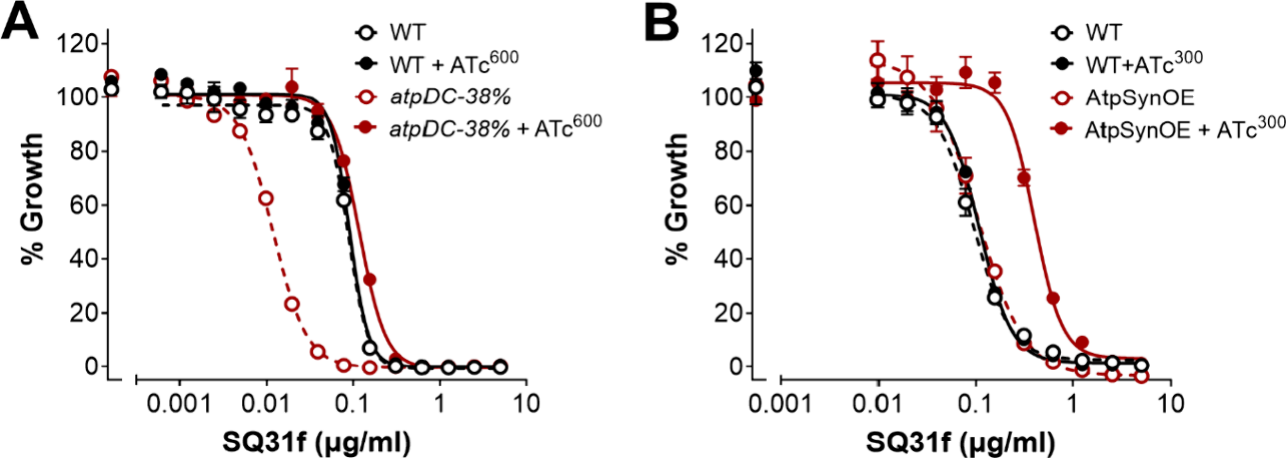
Susceptibility of parent squaramide SQ31f directly correlates with ATP synthase expression. (A) Growth of *Mtb* (black) and *atpDC*-*38%* (red) with increasing concentrations of SQ31f without (-ATc, open, dashed) or with inducer (+ATc at 600 ng/mL, closed, solid). (B) Growth of *Msmeg* (black) and AtpSynOE (red) with increasing concentrations of SQ31f without (−ATc, open, dashed) and with the inducer (+ATc at 300 ng/mL, closed, solid). Data are averages of three replicates (±S.E.M) and representative of two independent experiments.

We next proceeded to examine how the potency of diamino-substituted SQA derivatives changes response to expression levels of ATP synthase. In comparison to WT *Mtb*, the potency of **PRP020**, **PRP021**, and **PRP017** increased by 8.1-, 9.8-, and 6.4-fold, respectively, when ATP synthase levels were reduced to 38% (Figure 8A) and were reduced by 3.6-, 3.1-, and 4.0-fold, respectively, when *Mtb* ATP synthase was expressed in addition to *Msmeg* ATP synthase (Figure 8B). Together, these data confirmed that the potency of SQA scaffold directly correlated with ATP synthase expression, confirming that ATP synthase is the target of the monoarylated SQA **SQ31f** and its diamino-substituted derivatives **PRP020**, **PRP021**, and **PRP017.**

**Figure 8:**
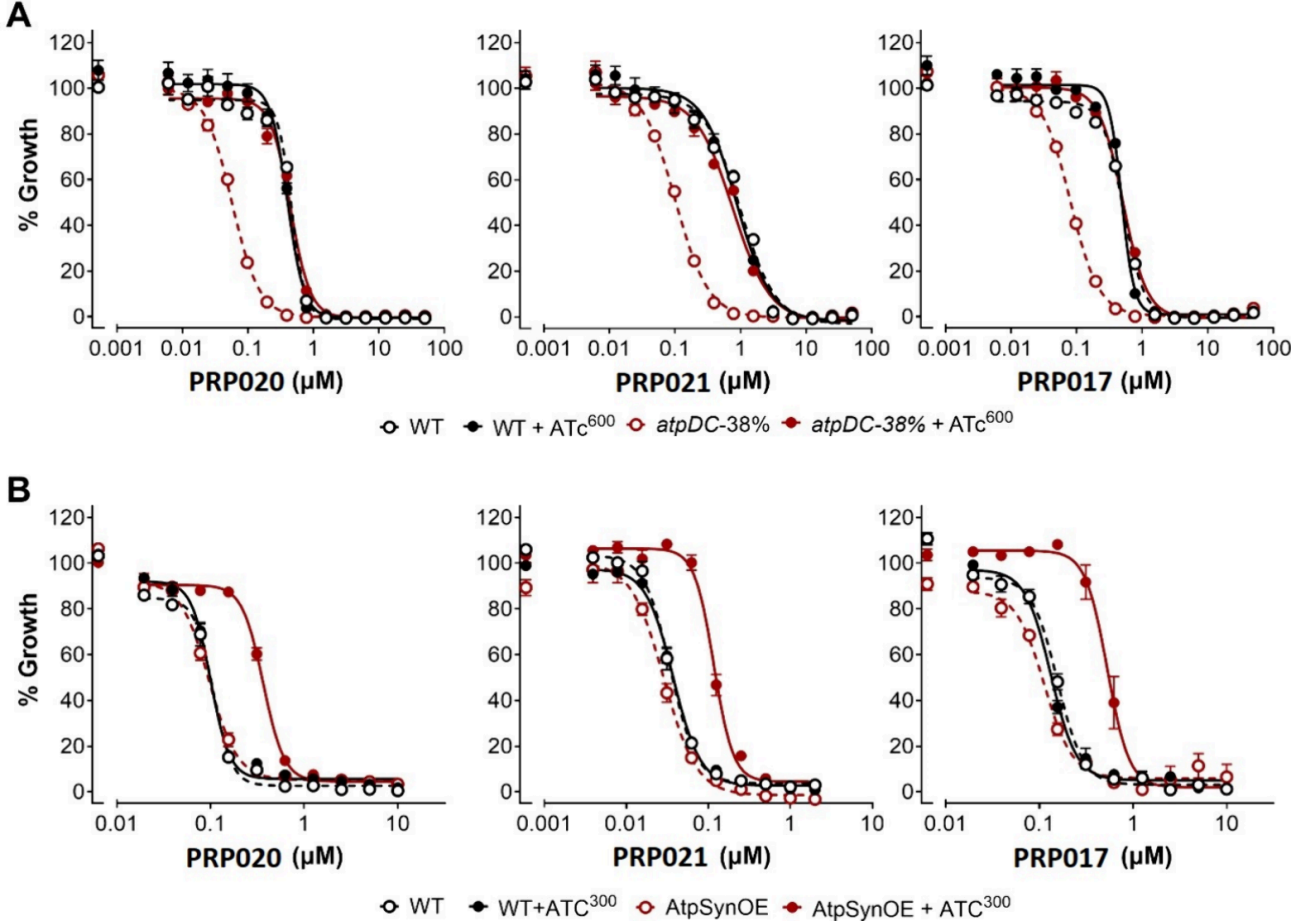
Susceptibility of *Mtb* to growth inhibition correlates with ATP synthase expression. (A) Growth of *Mtb* (black) and atpDC-38% (red) with increasing concentrations of PRP020, PRP021, and PRP017 without (−ATc, open, dashed) or with the inducer (+ATc at 600 ng/mL, closed, solid). (B) Growth of *Msmeg* (black) and AtpSynOE (red) with increasing concentrations of PRP020, PRP021, and PRP017 without (−ATc, open, dashed) or with the inducer (+ATc at 300 ng/mL, closed, solid). Data are averages of three replicates (±S.E.M) and representative of two independent experiments.

## 3 Conclusions

The results summarized in this study contribute to the development of ATP synthase inhibitors from the SQA class of substances. Like **BDQ**, SQAs target the mycobacterial ATP synthase but differ from **BDQ** in terms of their chemical structure, binding site, and mechanism of action. SQAs are of therapeutic interest because they are unaffected by certain known BDQ resistance mechanisms.^36^ The clinical value of targeting *Mtb*’s ATP synthase was proven with several TB treatment regimens, including the recent example BPaLM (BDQ, pretomanid, linezolid, and moxifloxacin).^37^ We optimized and simplified the synthesis of SQAs with a Friedel–Crafts acylation that does not require the use of AlCl_3_ as Lewis acid and still proceeds efficiently. This procedure avoids the disposal of environmentally harmful aluminum salts.

The panel of SQAs described here were characterized both on the isolated target and in whole-cell assays against pathogenic mycobacteria. With regard to the structure–activity relationships of the SQAs, various observations can be summarized, although the results from whole-cell assays and enzyme inhibition assays do not always correlate. Some derivatives, such as **PRP006**, **PRP010**, **PRP011**, **PRP014**, **PRP018**, **PRP019**, and **PRP027**, show ATP synthase inhibition in ATP-driven IMV acidification assays, but no pronounced growth inhibition against mycobacteria. This behavior is not unusual, as permeability or bacterial metabolism can reduce efficacy in whole-cell assays. Regarding the SQAs active in the whole-cell assay, the following structure–activity relationships were observed: In the picolyl amine part of **SQ31f**/**SQ6ab**, the pyridine could be replaced by other heteroaromatics. Imidazole (**PRP017**) and thiazole (**PRP020**, **PRP021**, **PRP022**, **PRP023**, **PRP024**) proved to be particularly potent against *Mtb*. The 1,3-benzodioxol-5-amine substituent of **SQ6ab** could be replaced by various chemical structures while maintaining activity. The 2,2-dimethylbenzodioxol-5-amine substituent proved to be a suitable substitution to maintain activity (**PRP022**). It is also possible to replace the two oxygen atoms of the acetal structure with methylene groups while maintaining antimycobacterial activity (**PRP023**, **PRP024**). In addition, an aniline substituent could also be introduced at this point in the molecule (**PRP026**). Compared to the reference compounds **SQ31f** and **SQ6ab**, the diamino-substituted SQAs **PRP020** and **PRP024** showed improved activity against *Mtb* with MIC_95_ values of 1–2 μM. Both compounds share an (aminomethyl)­thiazole substituent. In contrast to the anti-*Mtb* activity of di- and monoamino-substituted SQAs, only the monoamino-substituted analogues were effective against *Mav*, with **PRP003** being the most effective substance. **PRP003** also has an (aminomethyl)­thiazole residue. A key finding of the study is the microsomal stability of **PRP020**, which is superior to the known lead compounds **SQ31f** and **SQ6ab**. This development is important for further investigation of SQAs, as both **SQ31f** and **SQ6ab** have poor bioavailability in mice due to hepatic degradation. Another crucial aspect of the study is the improved cytotoxicity and absence of antiproliferative effects of **PRP020**. The known lead compounds **SQ31f** and **SQ6ab** exhibit substantial antiproliferative activity at concentrations of 100 μM, while **PRP020** and several other compounds reported here do not show cytotoxicity even at 1000 μM, the highest concentration tested. This improvement in tolerability with mammalian cell lines is an important optimization of SQAs. Overall, this work demonstrates the high value of the (aminomethyl)­thiazole-substituted SQAs. It also justifies further medicinal-chemical development of SQAs for the treatment of TB and other mycobacterial infections.

## 4 Experimental Section

### 4.1 *M. smegmatis* Growth, IMV Preparation, and Acidification Assays

Assays were performed as described previously.^29^ Briefly, the *Msmeg* strains GMC_MSM1 (wild type with a 3 × FLAG affinity tag the C terminus of the β subunits of the ATP synthase) and GMC_MSM2 (identical to GMC_MSM1 but with a 3 × FLAG affinity tag truncating the α subunits of the ATP synthase at Ser518)^30^ were grown on LB agar plates with 50 μg/mL hygromycin for 2 days at 37 °C. A colony from the plate was transferred to a 25 mL preculture of 7H9 medium supplemented with TDS (10 g/L tryptone, 2 g/L dextrose, 0.8 g/L NaCl, 50 μg/mL hygromycin B, and 0.05% Tween 80). The strains were grown for 48 h in the dark at 37 °C, shaking at 180 rpm before being used to inoculate a 6 L culture, and growing at 30 °C and 180 rpm for 48 h. Cells were harvested by centrifugation for 20 min at 6500*g* and 4 °C. These strains are available from the Biodefense and Emerging Infections (BEI) Resources (www.beiresources.org) as Items #NR-59698 and #NR-59699.

Cell pellets were resuspended using a Caframo model BCD2002 compact Digital Overhead stirrer at 900 rpm in 25 mL of lysis buffer (50 mM Tris–HCl [pH 7.5], 150 mM NaCl, 5 mM MgSO_4_, 5 mM benzamidine hydrochloride, and 5 mM 6-aminocaproic acid) per 1 L of starting cell culture. The cell suspension was filtered through Miracloth (Millipore), and phenylmethylsulfonyl fluoride (PMSF) in ethanol was added to the suspension to achieve a final concentration of 1 mM from a 100 mM stock. Cells were lysed by passing them three times through an Avestin homogenizer operating at 20,000 to 25,000 psi. Insoluble debris was removed by centrifugation for 30 min at 39,000*g* and 4 °C, and the membrane fraction from the cells was collected by centrifugation for 1 h at ∼200,000*g* and 4 °C using a Beckmann Ti70 ultracentrifuge rotor. To form IMVs, membranes were resuspended with a Dounce homogenizer in 2.5 mL (GMC_MSM1) or 2.7 (GMC_MSM2) of resuspension buffer (50 mM Tris–HCl [pH 7.5], 150 mM NaCl, 5 mM MgSO_4_, 5 mM benzamidine hydrochloride, 5 mM 6-aminocaproic acid, and 20% [v/v] glycerol) per 1 L of original cell culture. The IMVs were divided into aliquots and stored at −80 °C. The concentration of total protein in the IMV suspension was determined by the bicinchoninic acid assay (Pierce) without solubilizing the IMVs and found to be 14 mg/mL for GMC_MSM1 and 11 mg/mL for GMC_MSM2.

IMV acidification assays were performed in a 96-well plate (BRANDplates pureGrade 96-well black microplates). Each well contained 80 μL 2× ACMA assay buffer (20 mM Hepes-KOH [pH 7.5], 200 mM KCl, and 10 mM MgCl_2_), 23.35 μL of Milli-Q water, 0.25 μL of ACMA dye from a 2 mM stock in ethanol, 10 μL of IMV, and 3.2 μL of test compound in 2% DMSO, or 3.2 μL of DMSO alone. ACMA fluorescence was followed with a BioTek Synergy Neo2Multimode Assay Microplate reader (Agilent Technologies) at 25 °C. The excitation and emission wavelengths were set to 410 and 480 nm, respectively. Fluorescence was monitored for 2 min to determine a baseline, at which point 40 μL of an energy source was added to each well using the instrument’s automated injector. Fluorescence was monitored for 15 min before 3.2 μL of 100 μM nigericin in 1% (v/v) ethanol was added to each well with a multichannel pipettor. The samples were then mixed with a different multichannel pipettor, and fluorescence signal recovery was monitored for an additional 5 min before ending the experiment. The fluorescence recovery was calculated by subtracting the average of the last 10 data points before nigericin injection from the average of the last 10 data points after nigericin injection. Relative activity was reported as the fluorescence recovery normalized by the mean fluorescence recovery without an inhibitor:

$$\mathrm{Fluorescence}\;\mathrm{Recovery}=\mathrm{Average}\;{\mathrm{RFU}}_{\mathrm{post}-\mathrm{nigericin}}-\mathrm{Average}\;{\mathrm{RFU}}_{\mathrm{pre}-\mathrm{nigericin}}$$

$$\mathrm{Relative}\;\mathrm{Activity}=\frac{\mathrm{Fluorescence}\;{\mathrm{Recovery}}_{\mathrm{Inhibitor}}}{\mathrm{Mean}\;\mathrm{Fluorescence}\;{\mathrm{Recovery}}_{\mathrm{DMSO}}}$$

In ATP-driven acidification assays, IMVs from the GMC_MSM2 strain were added to the master solution, and 40 μL of 8 mM disodium ATP in 16 mM Tris was injected without adjusting the pH (pH ∼ 7), resulting in a final concentration of 2 mM ATP/well. The fluorescence gain was set to 100.

For succinate-driven acidification assays, IMVs from the GMC_MSM1 strain were diluted 8-fold in resuspension buffer before use, and 40 μL of 20 mM sodium succinate in water was injected into the wells, resulting in a final concentration of 5 mM succinate per well. The fluorescence gain was set to 90.

### 4.2 Whole-Cell Assays

#### MIC Determination against *M. smegmatis* mc^2^ 155 and *M. abscessus* ATCC19977 pTEC27

MIC values were determined by the broth microdilution method. 96-well flat-bottom tissue culture plates (Sarstedt, 83.3924.500) were used. In the third well of each row, two times the desired highest concentration of each compound was added in 7H9 medium supplemented with 10% ADS (albumin, dextrose, and saline) and 0.05% Tween 80. Each compound was diluted 2-fold in a nine-point serial dilution. The concentration of the starting inoculum was 5 × 10^5^ CFU/mL. The starting inoculum was diluted from a preculture at the mid log phase (OD_600_ 0.3 to 0.8) and an OD_600_ of 0.1 was correlated to 1 × 10^8^ CFU/mL. The plates were sealed with parafilm, placed in a container with moist tissue, and incubated for 3 days (*Msmeg*) or 4 days (*Mabs*) at 37 °C. Each plate had eight negative controls (1% dimethyl sulfoxide) and eight positive controls (100 μM amikacin). After incubation the plates were monitored by OD measurement at 550 nm (BMG LABTECH FLUOstar Optima). The *Mabs* plates were additionally evaluated by fluorescence measurement and by measurement (λ_ex_ = 544 nm λ_em_ = 590 nm), and care was taken to ensure consistent values; the results of the OD measurement are shown in the manuscript.

##### Data Analysis

Every assay plate contained eight wells with dimethyl sulfoxide (1%) as negative control, which corresponds to 100% bacterial growth and eight wells with amikacin (100 μM) as positive control in which 100% inhibition of bacterial growth was reached. Controls were used to monitor the assay quality through determination of the *Z*′ score. The *Z*′ factor was calculated as follows:

$$Z^{’}=1-\frac{3\left({\mathrm{SD}}_{\mathrm{amikacin}}+{\mathrm{SD}}_{\mathrm{DMSO}}\right)}{M_{\mathrm{amicacin}}-M_{\mathrm{DMSO}}}$$

SD = standard deviation; M = mean.

The percentage of growth inhibition was calculated by the equation:

$$\%\mathrm{growth}\;\mathrm{inhibition}=-100\%\times \frac{\mathrm{signal}\left(\mathrm{sample}\right)-\mathrm{signal}\left(\mathrm{DMSO}\right)}{\mathrm{signal}\left(\mathrm{DMSO}\right)-\mathrm{signal}\left(\mathrm{amikacin}\right)}$$

#### MIC Determination against *Mycobacterium tuberculosis* H37Rv (ATCC 27294, *atpE-* and *Rv0689* mutants) and *Mycobacterium avium* (ATCC 700898)

*Mtb* H37Rv (ATCC 27294, *atpE-* and *Rv0689* mutants) and *Mav* (ATCC 700898) were cultured in 7H9 complete medium (BD Difco; Becton Dickinson (BD)) supplemented with oleic acid–albumin–dextrose–catalase (OADC, 10%; BD) in the absence of glycerol and Tween 80 until OD 0.4. Cultures were centrifuged (1900*g*, 10 min). Supernatants were discarded, and bacteria were washed twice with PBS and subsequently thoroughly resuspended (5 times) in 7H9 medium (10% OADC) by use of a syringe and a 26-gauge syringe needle. Compound efficacy was assessed using a broth microdilution assay, based on a previously described protocol^35^ with a few modifications. Briefly, 2-fold serial dilutions of each compound were prepared starting at a concentration of 64 μM. Wells were inoculated with *Mtb* (2 × 10^5^ CFU) or *Mav* (1 × 10^4^ CFU) per well in a final volume of 100 μL, using clear, U-bottom 96-well microtiter plates to evaluate antitubercular activity. Plates were left to incubate at 37 °C, in ziplock bags within humidified sealed boxes for 7 days (*Mtb*) or 3 days (*Mav*), prior to visual examination of bacterial growth using an inverted mirror device. 30 μL of a 0.02% (w/v) aqueous solution of resazurin (Cayman Chemical, Ann Arbor, USA) was then added to each well and the plates left to incubate at 37 °C for 4 h. Reduction of resazurin to resorufin, indicative of cell viability, was measured with a fluorescence plate reader (Synergy2, Agilent) at wavelengths 540 nm­(ex)/590­(em) nm. Fluorescence values were converted to percentage growth relative to DMSO-treated cells (100% growth). Final DMSO concentrations did not exceed 1% (v/v). The obtained values were normalized to fluorescence values of the solvent control (DMSO)-treated bacteria set to 100%), and MIC_95_ of each compound was determined. MIC_95_ was defined as the minimum concentration of the compound required to achieve a reduction in fluorescence by 95%.

Diarylquinoline resistant mutants were evolved from *Mtb* H37Rv lab strain (ATCC 27294) under sublethal drug exposure to either BDQ or clofazimine (CFZ) as recently described.^35^ Briefly, H37Rv was exposed to subinhibitory concentrations of BDQ or CFZ and plated on antibiotic supplemented 7H10 plates. Single colonies were selected and grown in standard media (7H9, 10% OADC, 0.5% glycerol, and 0.25% Tween 80) and frozen at −80 °C. The bacteria were thawed and regrown in standard media from which new aliquots were made and whole genome sequencing (WGS) was performed. The mutants utilized in these experiments did not carry any additional variants (as compared to the wild-type ancestor) besides the BDQ-associated variant, either atpE D28G, A62P, E61D, or Rv0678 Q51R or R38stop. Sequences were deposited in the National Library of Medicine - National Center for Biotechnology Information (project number PRJNA1304451).

### 4.3 Toxicity Assays

#### Cell Culture

16HBE14σ cells were obtained from Sigma-Aldrich (Sigma-Aldrich, #SCC150) and cultivated in MEM Eagle (PAN-Biotech, P04-08500) medium, both of which were supplemented with 10% fetal calf serum (FCS) (Anprotec, #AC-SM-0027) at 37 °C, 5% CO_2_. J774A.1 cells were obtained from ATCC and cultivated in Dulbecco’s modified Eagle’s medium (DMEM) (Anprotec, #AC-LM-0012) supplemented with 10% FCS. None of the cell lines used are listed in the database of commonly misidentified cell lines maintained by ICLAC. STR profiling is performed regularly. All cells are proven to be mycoplasma-free quarterly.

#### Cell Viability Assay—CellTiter-Blue Assay

16HBE14σ The CellTiter-Blue assay (Promega, Mannheim, Germany) was used to evaluate potential cell toxicity and calculate the IC_50_ values of the tested squaramides. Therefore, cells were seeded into 96-well plates at densities of 10,000 cells/well for 16HBE14σ and 15,000 cells/well for J77A.1. After a 24 h settling period, cells were treated with squaramides at final concentrations of 10 or 100 μM. Following a 22 h incubation at 37 °C (5% CO_2_), the CellTiter-Blue reagent was added at a 1:5 ratio. Cells were further incubated for 2 h under the same conditions. Fluorescence was measured using a SpectraFluor Plus plate reader (Tecan, Männedorf, Switzerland) with excitation at 550 nm and emission at 595 nm.

To determine potential IC_50_ values, 2000 cells/well (16HBE14σ) or 5000 cells/well (J774A.1) were seeded into a 96-well plate each using a multichannel pipet. After a 24 h settling period, cells were treated with squaramides as indicated. After an incubation time of 70 h at 37 °C (5% CO_2_), relative proliferation was calculated the following way:

$$\mathrm{relative}\;\mathrm{cytoxicity}=\frac{x-\mathrm{zero}\;\mathrm{value}}{\mathrm{control}-\mathrm{zero}\;\mathrm{value}}$$

### 4.4 Metabolic Stability in Mouse Liver Microsomes

For the evaluation of phase I metabolic stability, the compounds (1 μM) were incubated with 0.5 mg/mL pooled mouse liver microsomes (Xenotech, Kansas City, USA), 2 mM NADPH, and 10 mM MgCl_2_ at 37 °C for 120 min on a microplate shaker (Eppendorf, Hamburg, Germany). The metabolic stability of testosterone, verapamil, and ketoconazole was determined in parallel to confirm the enzymatic activity of mouse liver microsomes. The incubation was stopped after defined time points by precipitation of aliquots of enzymes with two volumes of cold internal standard solution (15 nM diphenhydramine in 10% methanol/acetonitrile). Samples were stored on ice until the end of the incubation, and precipitated protein was removed by centrifugation (15 min, 4 °C, 4000*g*). The remaining test compound at the different time points was analyzed by HPLC-MS/MS (Vanquish Flex coupled to a TSQ Altis Plus, Thermo Fisher, Dreieich, Germany) and used to determine half-life (*t*_1/2_).

### 4.5 Drug Susceptibility Testing in Bacterial Strains with Regulated *Mtb* ATP Synthase

All reagents were sourced commercially (Sigma-Aldrich, Research Products International, BD Difco, Thermo Fisher, Roche, MedChemExpress) and were used without further purification. Compound stocks at 10 mM were prepared in DMSO. Stocks of anhydrotetracycline (ATc), ciprofloxacin, and rifampicin were prepared in DMSO. The antibiotics Zeocin (zeo) and hygromycin (hyg) were added at 25 and 50 μg/mL where indicated. All *Mtb* strains were grown in Middlebrook 7H9 broth supplemented with 0.5% (w/v) bovine serum albumin, 0.2% (w/v) dextrose, 0.085% (w/v) sodium chloride, 0.2% (v/v) glycerol, and 0.05% (v/v) tyloxapol. All *Msmeg* strains were grown in Brain Heart Infusion broth supplemented with 0.05% (v/v) Tween80. Anhydrotetracycline (ATc, Sigma-Aldrich) was used at 300 and 600 ng/mL as per strain. Bacterial strains were cultivated at 37 °C, 5% CO_2_, and 80% relative humidity unless otherwise specified. The effect of a given compound was estimated as % growth = 100 × [(data – max inhibition control)/(max growth control – max inhibition control)].

Construction of bacterial strains with regulated *Mtb* ATP synthase levels will be described elsewhere (Grover et al., manuscript in preparation). For drug susceptibility assays, WT *Mtb* and the derivative expressing 38% ATP synthase (*atpDC-38%*) were cultivated with ATc until mid to log phase (OD_580_ = 0.6 to 1.0). Cells were harvested, washed, and resuspended in 30 mL of Middlebrook 7H9 broth without and with inducer ATc to a final OD_580_ of ∼0.05 and incubated at 37 °C for up to 48 h. Next, 50 μL aliquots of a single-cell suspension (OD_580_ ∼ 0.01) were used to seed black/clear, flat-bottom 384-well microplates (GrenierBio One) containing a nanoliter volume of predispensed compounds. The *Msmeg*-expressing *Mtb* ATP synthase (AtpSynOE) and WT were cultivated in BHI Broth until mid to log phase (OD_580_ = 0.6 to 1.0). Cells were resuspended to an OD_580_ ≈ 0.02 in 30 mL of BHI without or with inducer ATc and incubated at 37 °C, 120 rpm, until OD_580_ ≈ 0.4 to 0.8. Next, 100 μL aliquots of a single-cell suspension (OD_580_ ∼ 0.05) were used to seed 96-well plates (Costar) containing nanoliter volume of predispensed compounds. DMSO concentrations were normalized to 1% across all wells. Plates were incubated at 37 °C for 9 to 14 days for *Mtb* and 3 to 5 days for *Msmeg*. A minimum of 10 doses, each diluted by 2-fold, were assessed. Rifampicin (*Mtb*) or ciprofloxacin (*Msmeg*) at 2 μg/mL was used as control for 100% inhibition. *Msmeg* was resuspended in multiwell plates by pipetting. Growth measurements were performed using SpectraMax M2e or SpectraMax iD3. MIC_50_ values were determined using the variable-slope four-parameter nonlinear least-squares regression model in GraphPad Prism software package (version 10.4).

### 4.6 Chemistry

#### 4.5.1 Materials and Instruments

Solvents were used after distillation and storing over 4 Å molecular sieves. Deuterated solvents for NMR spectroscopy were purchased from Eurisotop (Saarbrücken, Delaware). All further reaction chemicals were purchased either from Sigma-Aldrich (Hamburg, Delaware), TCI Chemicals (Tokyo, Japan), or BLD Pharmatech Ltd. (Shanghai, China) and used as received. All reactions were monitored by thin-layer chromatography (TLC) on silica gel plates using Merck TLC silica gel 60 on aluminum sheets with fluorescent indicator F254. APCI-MS (atmospheric pressure chemical ionization) was performed using an expression CMS mass spectrometer (Advion Inc., Ithaca, New York, USA), with both ASAP (atmospheric solids analysis probe) sampling and with the help of the Plate Express TLC-plate extractor. ESI measurements have been conducted on the same expression CMS mass spectrometer with an ESI ionization module and direct injection sampling. HRMS was carried out using an LTQ Orbitrap XL mass spectrometer (Thermo Fisher Scientific Inc., Waltham, Massachusetts, USA). Flash chromatography was performed with a puriFlash 430 instrument (Interchim, Montluçon, France). Columns were packed in either 8 g (*v* = 10 mL/min), 45 g (*v* = 30 mL/min), or 90 g (*v* = 40 mL/min) cartridges with 40–63 μm normal phase silica gel produced by Carl Roth. Column loading was performed with the dry load method. The maximum compound load per column was 5% (m/m) of the silica gel quantity. NMR spectra were recorded on an Agilent Technologies 400 MHz VNMRS, Agilent Technologies 600 MHz shielded VNMRS, and Jeol 600 MHz - JNM-ECZL600G spectrometers at 25 °C. The chemical shifts of ^1^H NMR and ^13^C NMR spectra are referenced on the solvent residual signals of CDCl_3_ (δ_H_ = 7.26 ppm; δ_C_ = 77.36 ppm) or DMSO-*d*_6_ (δ_H_ = 2.48 ppm; δ_C_ = 40.01 ppm). Spectra have been cut, baseline and phase corrected, and analyzed utilizing MestreNova 11.0 software (Mestrelab Research, S.L., Spain). Melting points were determined with Electrothermal IA 9100 by Fisher Bioblock Scientific (Brussels, Belgium). All described final compounds were confirmed to be of >95% purity determined by HPLC analysis using a Shimadzu instrument (Shimadzu, Kyoto, Japan) with a CBM-40 control unit, a DGU-403 degassing unit, two LC-40D chromatography pumps, an SIL-40C autosampler unit, a CTO-40C column oven, and an SPD M40 PDA UV detector. The standard method for purity determination utilized an Agilent Poroshell 120, EC-C18, 3.0 × 50 mm, 2.7 μm analytical column at a flow rate of 1.2 mL/min at room temperature. The 6 min gradient started at 5% and increased to 95% acetonitrile in water and was used in all cases for this column. If the peak width was too large, the gradient was adjusted to 10 min. For some compounds, the purity determination was done using Agilent Polaris 5 C18-A 250 × 4.6 mm analytical column at a flow rate of 1.2 mL/min at room temperature. The 15 min gradient started at 5% and increased to 95% acetonitrile in water and was used in all cases for this column. All solvents used are HPLC-grade purity. UV absorbance at 254 nm was measured, and the purity was derived from the integrated intensity signal. In case of solubility problems of the sample, HPLC-grade DMSO was added.

#### 4.5.2 Synthetic Procedures

##### Synthesis of Squaric Acid Dichloride^38^

3,4-Dihydroxycyclobut-3-ene-1,2-dione (5.00g, 43.86 mmol) was placed in a flame-dried round-bottom flask followed by addition of 2.1 equiv thionyl chloride (10.96 g, 6.69 mL, 92.11 mmol) and 10 drops of *N*,*N*-dimethylformamide. The mixture was stirred for 12 h at 80 °C followed by evaporation of excessive thionyl chloride. The resulting precipitate was washed with hot heptane. The solid was dried under reduced pressure. 3,4-Dichlorocyclobut-3-ene-1,2-dione was then used without further purification (see respective synthesis documentation in the Supporting Information).

##### Monoarylation of Squaric Acid Dichloride^25^

Freshly prepared 3,4-dichlorocyclobut-3-ene-1,2-dione (1.00 g, 6.63 mmol) was dissolved in dry toluene (80 mL) followed by addition of *N*-phenylmorpholine (1.08 g, 6.63 mmol). The reaction was heated to 85 °C for 4 h followed be addition of methanol (1.35 mL, 33.15 mmol) and *N*,*N*-diisopropylethylamine (2.55 mL, 13.26 mmol) and stirring at RT for 1 h. The solvents were removed under reduced pressure, and the resulting mixture was purified by a silica gel-packed flash chromatography column with eluent heptane/ethyl acetate (1/0 to 1/2), which gave 3-methoxy-4-(4-morpholinophenyl)­cyclobut-3-ene-1,2-dione (Intermediate 1 in S.I.) as a yellow solid in 0.79 g (44%, 0.29 mmol) yield.

##### General Procedure **1** Synthesis of SQA Monoamino-Substituted Squaramides^19^

One equivalent of 3-methoxy-4-(4-morpholinophenyl)­cyclobut-3-ene-1,2-dione was dissolved in methanol followed by addition of one equivalent amine and corresponding equivalents DIPEA if a hydrochloride was used. The solution was stirred overnight at room temperature. The resulting precipitate (**SQ31f** and **PRP001**-**004** in S.I.) was centrifuged, washed with isopropanol-heptane, and vacuum-dried (see respective synthesis documentation in the Supporting Information).

##### General Procedure **2** Synthesis of Monoamino-Substituted Squaric Acid Methylesters^20^

One equivalent of commercially available 3,4-dimethoxycyclobut-3-ene-1,2-dione was dissolved in methanol, and one equivalent corresponding amine was added. The solution was stirred overnight at room temperature. The resulting precipitate (Intermediate 2–14 in S.I.) was centrifuged, washed with isopropanol-heptane, and vacuum-dried (see respective synthesis documentation in the Supporting Information).

##### General Procedure **3** Synthesis of Diamino-Substituted Squaramides^20^

One equivalent Intermediate 2–14 was dissolved in methanol, and one equivalent of corresponding amine was added. If the amine was present as a hydrochloride, equimolar amounts of DIPEA were added. The solution was stirred overnight at room temperature. The resulting precipitate (SQ6ab and **PRP005**-**031** in S.I.) was centrifuged, washed with isopropanol-heptane, and vacuum-dried (see respective synthesis documentation in the Supporting Information).

##### General Procedure 4 *N*-Methylation of Primary Amines^39^

One equivalent amine was dissolved in methanol with 10 equiv paraformaldehyde. Five equivalents of sodium methoxide was added, and the mixture was stirred for 24 h at room temperature. Then, three equivalents of sodium borohydride was added to the solution and stirred for 3 h at 45 °C. The solvent was removed under reduced pressure, and the residue was dissolved in ethyl acetate and subsequently washed with water and brine. After evaporation of the solvent, the *N*-methylated amine (Intermediate 15 and 16 in S.I.) was used without further purification (see respective synthesis documentation in the Supporting Information).

All synthetic protocols and reaction schemes can be found in the Supporting Information with associated spectra, chromatograms, and additional data.

## Supplementary Material





## Notes

Structures and synthetic protocols of intermediates and final compounds, activity of SQA derivatives against clinical mycobacterial strains, toxicity of inactive SQA derivatives, additional graphs for relative proliferation, NMR spectra, and HPLC-purity chromatograms (PDF)
Molecular formula strings, clogP, MICs of SQAs against clinical mycobacterial strains, and microsomal *t*_1/2_ and clearance (CSV)

## Abbreviations

ABT: aminobenzotriazole
ACMA: fluorophore 9-amino-6-chloro-2-methoxyacridine
ATc: anhydrotetracycline
ATP: adenosine triphosphate
BDQ: bedaquiline
BPaLM: bedaquiline, pretomanid, linezolid, and moxifloxacin
DMF: *N*,*N*-dimethylformamide
DIPEA: *N*,*N*-diisopropylethylamine
hERG: human ether-à-go-go-related gene
FDA: US Food and Drug Administration
IMVs: inverted membrane vesicles
Mav: *Mycobacterium avium*
Mabs: *Mycobacterium abscessus*
MeOH: methanol
MDR: multidrug-resistant
MIC: minimal inhibitory concentration
Msmeg: *Mycobacterium smegmatis*
Mtb: *Mycobacterium tuberculosis*
NTM: nontuberculous mycobacteria
OD: optical density
PMF: proton motive force
RT: room temperature
SQA: squaramide
TB: tuberculosis
TLC: thin-layer chromatography
WT: wild type
XDR: extensively drug-resistant.
