# Supplementary material for: Design, Synthesis, and Biological Evaluation of Mono- and Diamino-Substituted Squaramide Derivatives as Potent Inhibitors of Mycobacterial Adenosine Triphosphate (ATP) Synthase
Source: J Med Chem. 2025 Nov 24;68(23):25274–89. doi: 10.1021/acs.jmedchem.5c02284 (PMC12703743; doi:10.1021/acs.jmedchem.5c02284)
Supplement: Supplementary file 1 [file jm5c02284_si_001.pdf]

Supplementary Information

Design, synthesis and biological evaluation of mono- and diamino substituted squaramide derivatives as potent inhibitors of mycobacterial adenosine triphosphate (ATP) synthase

Paul R. Palme[1], Shipra Grover[2], Rana Abdelaziz[3], Lea Mann[1], Andreas M. Kany[4,5], Lina Ouologuem[6], Karin Bartel[6], Lindsay Sonnenkalb[7], Norbert Reiling[8,11], Anna K. H. Hirsch[4,5,9], Dirk Schnappinger[2], John L. Rubinstein[3,10], Peter Imming[1]*, Adrian Richter[1]*

[1]Martin-Luther-Universität Halle-Wittenberg (Pharmaceutical Chemistry, Institute of Pharmacy, Halle (Saale) 06120, Germany)

[2]Weill Cornell Medical College (Department of Microbiology and Immunology, New York NY 10065, USA)

[3]The Hospital for Sick Children (Molecular Medicine Program, Toronto ON M5G1H3, Canada)

[4]Helmholtz Institute for Pharmaceutical Research Saarland (HIPS)–Helmholtz Centre for Infection Research (HZI), Saarbrücken 66123, Germany

[5]PharmaScienceHub, Saarbrücken 66123, Germany

[6]Ludwig Maximilian University of Munich (Department of Pharmacy, Faculty of Chemistry and Pharmacy, Munich 81377, Germany)

[7]Research Center Borstel, Leibniz Lung Center (Molecular and Experimental Mycobacteriology, Borstel 23845, Germany)

[8]Research Center Borstel, Leibniz Lung Center (Microbial Interface Biology, Borstel 23845, Germany)

[9]Saarland University (Department of Pharmacy, Campus E8.1, Saarbrücken 66123, Germany)

[10]The University of Toronto (Departments of Biochemistry and Medical Biophysics, Toronto ON M5G 1L7, Canada)

[11]German Center for Infection Research (DZIF, Partner Site Hamburg-Lübeck-Borstel-Riems, Borstel 23845, Germany)

*Corresponding author:

* Prof. Dr. Peter Imming, Pharmazeutische Chemie, Institut für Pharmazie, Kurt-Mothes-Straße 3, 06120 Halle (Saale), Germany

Email: peter.imming@pharmazie.uni-halle.de

* Dr., Adrian Richter, Pharmazeutische Chemie, Institut für Pharmazie, Kurt-Mothes-Straße 3, 06120 Halle (Saale), Germany

Email: adrian.richter@pharmazie.uni-halle.de

Table of Contents

1. **Structures and synthetic protocols**
    a. Intermediates 1-16 S3-S8
    b. Monoamino substituted SQAs S9-S12
    c. Diamino substituted SQAs S12-S28
2. **Activity of SQA derivatives against various mycobacterial strains** S29
3. **Additional cytotoxicity data of SQA derivatives** S29
4. **Reaction schemes for Synthetic procedures** S30-S31
5. **References** S31
6. **NMR spectra** S32-S82
7. **HPLC-purity chromatograms** S83-S91

1. Structures and synthetic protocols

a. Intermediates 1-16

3-Methoxy-4-(4-morpholinophenyl)cyclobut-3-ene-1,2-dione (Intermediate 1)

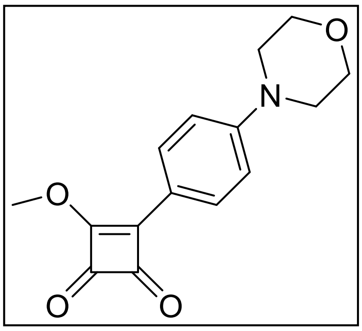

According to the procedure "Synthesis of squaric acid dichloride" (see manuscript) freshly prepared 3,4-dichlorocyclobut-3-ene-1,2-dione (1.00 g, 6.63 mmol) was dissolved in dry toluene (80 mL) followed by addition of *N*-phenylmorpholine (1.08 g, 6.63 mmol). The reaction was heated to 85 °C for 4 h followed be addition of methanol (1.35 mL, 33.15 mmol) and *N,N*-diisopropylethylamine (2.55 mL, 13.26 mmol) and stirring at RT for one hour. The solvents were removed under reduced pressure and the resulting mixture was purified by a silica gel-packed flash chromatography column with eluent heptane/ethyl acetate (1/0 to 1/2), which gave **Intermediate 1** as a yellow solid in 0.79 g (44 %, 2.9 mmol) yield.

^{1}H-NMR (600 MHz, DMSO-d_6) δ 7.78 – 7.75 (m, 2H), 7.10 – 7.06 (m, 2H), 4.47 (s, 3H), 3.74 – 3.70 (m, 4H), 3.34 – 3.30 (m, 4H);

MS (APCI, MeOH): *m/z* $[M+H]^+$ calcd for $C_{15}H_{15}NO_4$: 274.1, found: 274.4.

3-Methoxy-4-((4-morpholinophenyl)amino)cyclobut-3-ene-1,2-dione (Intermediate 2) [1]

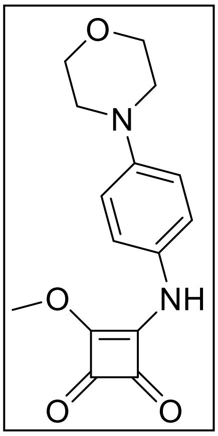

3,4-Dimethoxycyclobut-3-ene-1,2-dione (0.43 g, 3.00 mmol) was dissolved in methanol (50 mL) followed by addition of 4-morpholino-aniline (0.53 g, 3.00 mmol). The reaction was carried out as described in General Procedure 2, which gave **Intermediate 2** as a pale grey solid in 0.69 g (80 %, 2.41 mmol) yield.

^{1}H-NMR (400 MHz, DMSO-d_6) δ 10.56 (s, 1H), 7.19 (s, 2H), 6.94 – 6.85 (m, 2H), 4.34 (s, 3H), 3.75 – 3.66 (m, 4H), 3.10 – 2.99 (m, 4H).

HRMS (ESI, DMSO): *m/z* $[M+H]^+$ calcd for $C_{15}H_{16}N_2O_4$: 289.1183, found: 289.1177.

3-Methoxy-4-((pyridin-2-ylmethyl)amino)cyclobut-3-ene-1,2-dione (Intermediate 3) [2]

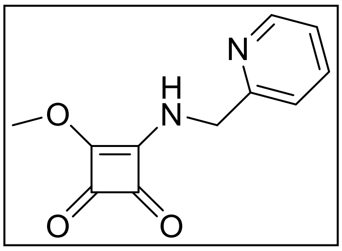

3,4-Dimethoxycyclobut-3-ene-1,2-dione (0.14 g, 1.00 mmol) was dissolved in methanol (30 mL) followed by dropwise addition of 2-(aminomethyl)pyridine (0.11 g, 103 µL, 1.00 mmol) and triethylamine (0.20 g, 280 µL, 2.00 mmol). The reaction was carried out as described in General Procedure 2, which gave **Intermediate 3** as a yellow solid in 0.13 g (60 %, 0.60 mmol) yield.

^{1}H-NMR (402 MHz, DMSO-d_6) δ 9.21 (t, *J* = 6.9 Hz, 0.5H), 9.02 (t, *J* = 6.3 Hz, 0.5H), 8.51 (d, *J* = 4.9 Hz, 1H), 7.76 (td, *J* = 7.7, 1.8 Hz, 1H), 7.33 (d, *J* = 7.9 Hz, 1H), 7.27 (dd, *J* = 7.6, 4.9 Hz, 1H), 4.76 (d, *J* = 6.3 Hz, 1H), 4.54 (d, *J* = 6.4 Hz, 1H), 4.26 (s, 1.5H), 4.21 (s, 1.5H).

HRMS (ESI, DMSO): *m/z* $[M+H]^+$ calcd for $C_{11}H_{10}N_2O_3$: 219.0764, found: 219.0763.

3-(Benzo[*d*][1,3]dioxol-5-ylamino)-4-methoxycyclobut-3-ene-1,2-dione (Intermediate 4) [3]

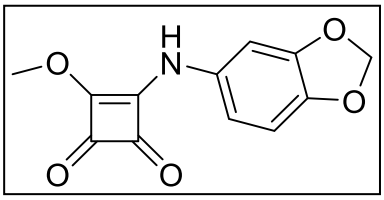

3,4-Dimethoxycyclobut-3-ene-1,2-dione (1.42 g, 10.00 mmol) was dissolved in methanol (100 mL) followed by addition of benzo[*d*][1,3]dioxol-5-amine (1.37 g, 10.00 mmol). The reaction was carried out as described in General Procedure 2, which gave **Intermediate 4** as a grey solid in 2.26 g (91 %, 9.14 mmol) yield.

^{1}H-NMR (402 MHz, DMSO-d_6) δ 10.59 (s, 1H), 6.95 (s, 1H), 6.84 (d, *J* = 8.3 Hz, 1H), 6.79 – 6.67 (m, 1H), 5.97 (s, 2H), 4.33 (s, 3H);

HRMS (ESI, HRMS): *m/z* $[M+H]^+$ calcd for $C_{12}H_9N_4O_4$: 248.0553, found: 248.0553.

3-((2,2-Dimethylbenzo[d][1,3]dioxol-5-yl)amino)-4-methoxycyclobut-3-ene-1,2-dione (Intermediate 5)

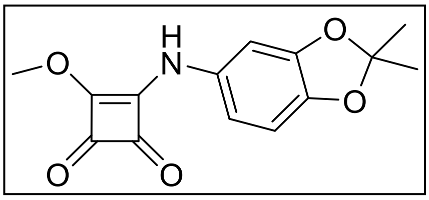

3,4-Dimethoxycyclobut-3-ene-1,2-dione (0.14 g, 1.00 mmol) was dissolved in methanol (50 mL) followed by addition of 2,2-dimethylbenzo[*d*][1,3]dioxol-5-amine (0.16 g, 1.00 mmol). The reaction was carried out as described in General Procedure 2, which gave **Intermediate 5** as a brown solid in 0.22 g (80 %, 0.80 mmol) yield.

^{1}H-NMR (402 MHz, DMSO-d_6) δ 10.57 (s, 1H), 6.88 (s, 1H), 6.76 (d, J = 8.3 Hz, 1H), 6.74 – 6.67 (m, 1H), 4.33 (s, 3H), 1.60 (s, 6H);

MS (APCI, MeOH): m/z $[M+H]^+$ calcd for $C_{14}H_{13}NO_5$: 276.1, found: 276.2.

3-((2,3-Dihydro-1H-inden-5-yl)amino)-4-methoxycyclobut-3-ene-1,2-dione (Intermediate 6) [1]

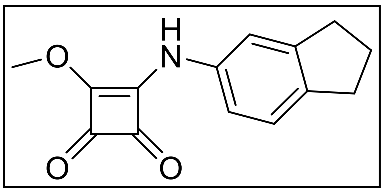

3,4-Dimethoxycyclobut-3-ene-1,2-dione (0.14 g, 1.00 mmol) was dissolved in methanol (50 mL) followed by addition of 2,3-dihydro-1*H*-inden-5-amine (0.13 g, 1.00 mmol). The reaction was carried out as described in General Procedure 2, which gave **Intermediate 6** as a grey solid in 0.23 g (94 %, 0.94 mmol) yield.

^{1}H-NMR (402 MHz, DMSO-d_6) δ 10.61 (s, 1H), 7.23 – 7.10 (m, 2H), 7.10 – 6.99 (m, 1H), 4.32 (s, 3H), 2.78 (dt, J = 10.7, 7.4 Hz, 4H), 1.97 (p, J = 7.4 Hz, 2H);

MS (APCI, MeOH): m/z $[M+H]^+$ calcd for $C_{14}H_{13}NO_3$: 244.1, found: 244.3.

3-Methoxy-4-((5,6,7,8-tetrahydronaphthalen-2-yl)amino) cyclobut-3-ene-1,2-dione (Intermediate 7)

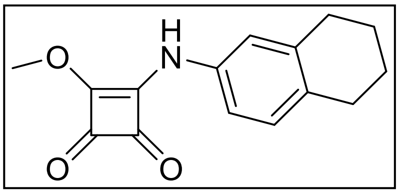

3,4-Dimethoxycyclobut-3-ene-1,2-dione (0.14 g, 1.00 mmol) was dissolved in methanol (50 mL) followed by addition of 5,6,7,8-tetrahydronaphthalen-2-amine (0.15 g, 1.00 mmol). The reaction was carried out as described in General Procedure 2, which gave **Intermediate 7** as a grey solid in 0.24 g (92 %, 0.92 mmol) yield.

^{1}H-NMR (402 MHz, DMSO-d_6) δ 10.01 (s, 1H), 7.25 – 7.05 (m, 2H), 6.95 (t, J = 8.9 Hz, 1H), 3.39 (s, 3H), 2.62 (dt, J = 12.2, 5.6 Hz, 4H), 1.66 (p, J = 3.2 Hz, 4H);

MS (APCI, MeOH): m/z $[M+H]^+$ calcd for $C_{15}H_{15}NO_3$: 258.1, found: 258.2.

3-((1,3-Dihydroisobenzofuran-5-yl)amino)-4-methoxycyclobut-3-ene-1,2-dione (Intermediate 8)

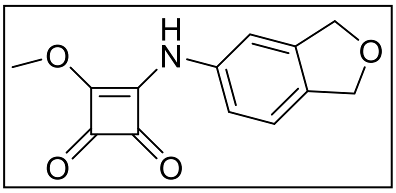

3,4-Dimethoxycyclobut-3-ene-1,2-dione (0.14 g, 1.00 mmol) was dissolved in methanol (50 mL) followed by addition of 1,3-dihydroisobenzofuran-5-amine (0.13 g, 1.00 mmol). The reaction was carried out as described in General Procedure 2, which gave **Intermediate 8** as a grey solid in 0.20 g (81 %, 0.81 mmol) yield.

^{1}H-NMR (402 MHz, DMSO-*d*$_6$) δ 10.75 (s, 1H), 7.36 – 7.13 (m, 3H), 4.94 (dq, *J* = 4.1, 1.9 Hz, 4H), 4.35 (s, 3H), 3.29 (s, 6H);

MS (APCI, MeOH): *m/z* $[M+H]^+$ calcd for $C_{15}H_{15}NO_3$: 246.1, found: 246.3.

3-((4-Chloro-3-fluorophenyl)amino)-4-methoxycyclobut-3-ene-1,2-dione (Intermediate 9)

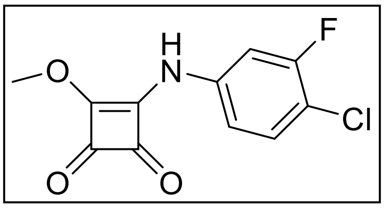

3,4-Dimethoxycyclobut-3-ene-1,2-dione (0.14 g, 1.00 mmol) was dissolved in methanol (50 mL) followed by addition of 4-chloro-3-fluoroaniline (0.15 g, 1.00 mmol). The reaction was carried out as described in General Procedure 2, which gave **Intermediate 9** as a grey solid in 0.19 g (76 %, 0.76 mmol) yield.

^{1}H-NMR (402 MHz, DMSO-*d*$_6$) δ 10.90 (s, 1H), 7.53 (t, *J* = 8.6 Hz, 1H), 7.42 (dd, *J* = 11.4, 2.4 Hz, 1H), 7.18 (dd, *J* = 9.0, 2.6 Hz, 1H), 4.36 (s, 3H);

MS (APCI, MeOH): *m/z* $[M+H]^+$ calcd for $C_{11}H_7ClFNO_3$: 256.0, found: 256.2.

3-((3-Bromo-4-chlorophenyl)amino)-4-methoxycyclobut-3-ene-1,2-dione (Intermediate 10)

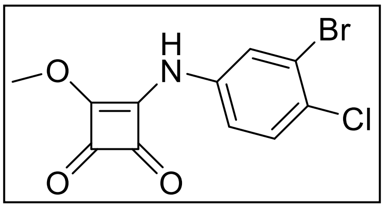

3,4-Dimethoxycyclobut-3-ene-1,2-dione (0.14 g, 1.00 mmol) was dissolved in methanol (50 mL) followed by addition of 3-bromo-4-chloroaniline (0.21 g, 1.00 mmol). The reaction was carried out as described in General Procedure 2, which gave **Intermediate 10** as a grey solid in 0.25 g (79 %, 0.79 mmol) yield.

^{1}H-NMR (402 MHz, DMSO-*d*$_6$) δ 10.85 (s, 1H), 7.77 (d, *J* = 2.6 Hz, 1H), 7.57 (d, *J* = 8.8 Hz, 1H), 7.35 (dd, *J* = 8.8, 2.7 Hz, 1H), 4.36 (s, 3H);

MS (APCI, MeOH): *m/z* $[M+H]^+$ calcd for $C_{11}H_7BrClNO_3$: 315.9, found: 316.1.

3-((3,4-Dichlorophenyl)(methyl)amino)-4-methoxycyclobut-3-ene-1,2-dione (Intermediate 11)

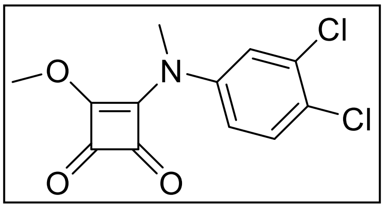

3,4-Dimethoxycyclobut-3-ene-1,2-dione (0.14 g, 1.00 mmol) was dissolved in methanol (50 mL) followed by addition of 3,4-dichloro-*N*-methylaniline (0.18 g, 1.00 mmol). The reaction was carried out as described in

General Procedure 2, which gave **Intermediate 11** as a grey solid in 0.11 g (61 %, 0.61 mmol) yield.

^{1}H-NMR (402 MHz, DMSO-d_6) δ 7.67 – 7.58 (m, 2H), 7.31 (dd, *J* = 8.7, 2.7 Hz, 1H), 4.27 (s, 3H), 3.57 (s, 3H);

MS (APCI, MeOH): *m/z* $[M+H]^+$ calcd for $C_{12}H_9Cl_2NO_3$: 286.0, found: 286.1.

3-((3,4-Difluorophenyl)(methyl)amino)-4-methoxycyclobut-3-ene-1,2-dione (Intermediate 12)

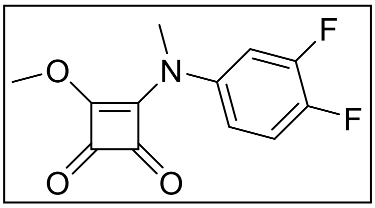

3,4-Dimethoxycyclobut-3-ene-1,2-dione (0.14 g, 1.00 mmol) was dissolved in methanol (50 mL) followed by addition of 3,4-difluoro-*N*-methylaniline (0.18 g, 1.00 mmol). The reaction was carried out as described in General Procedure 2, which gave **Intermediate 12** as a grey solid in 0.18 g (70 %, 0.70 mmol) yield.

^{1}H-NMR (402 MHz, DMSO-d_6) δ 7.55 – 7.41 (m, 2H), 7.18 (m, 1H), 4.26 (s, 3H), 3.56 (s, 3H);

MS (APCI, MeOH): *m/z* $[M+H]^+$ calcd for $C_{12}H_9F_2NO_3$: 254.1, found: 254.3.

***N*-Methylbenzo[*d*][1,3]dioxol-5-amine (Intermediate 13) [4]**

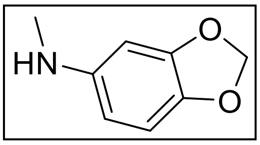

Benzo[*d*][1,3]dioxol-5-amine (0.21 g, 1.5 mmol) was dissolved in methanol (30 mL) followed by addition of paraformaldehyde (0.45 g, 15.00 mmol) and sodium methoxide (0.41 g, 7.5 mmol). After 24 h stirring at room temperature sodium borohydride (0.17 g, 4.5 mmol) was added and the reaction and the reaction was stirred for 3 h at 45 °C. The solvent was removed under reduced pressure and the residue was dissolved in ethyl acetate and subsequently washed with water and brine. Final evaporation of the solvent gave **Intermediate 13** as a grey solid in 0.21 g (93 %, 1.40 mmol) yield.

^{1}H-NMR (402 MHz, Chloroform-*d*) δ 6.67 (d, *J* = 8.3 Hz, 1H), 6.34 (d, *J* = 2.3 Hz, 1H), 6.16 (dd, *J* = 8.3, 2.4 Hz, 1H), 5.85 (s, 2H), 4.17 (s, 1H), 2.79 (s, 3H);

MS (APCI, MeOH): *m/z* $[M+H]^+$ calcd for $C_8H_9NO_2$: 152.1, found: 152.4.

***N*,2,2-Trimethylbenzo[*d*][1,3]dioxol-5-amine (Intermediate 14)**

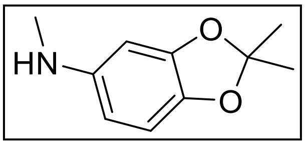

2,2-Dimethylbenzo[d][1,3]dioxol-5-amine (0.25 g, 1.5 mmol) was dissolved in methanol (30 mL) followed by addition of paraformaldehyde (0.45 g, 15.00 mmol)

and sodium methoxide (0.41 g, 7.5 mmol). After 24 h stirring at room temperature sodium borohydride (0.17 g, 4.5 mmol) was added and the reaction was stirred for 3 h at 45 °C. The solvent was removed under reduced pressure and the residue was dissolved in ethyl acetate and subsequently washed with water and brine. Final evaporation of the solvent gave **Intermediate 14** as a grey solid in 0.24 g (89 %, 1.34 mmol) yield.

^{1}**H-NMR** (402 MHz, Chloroform-*d*) δ 6.56 (d, *J* = 8.2 Hz, 1H), 6.15 (d, *J* = 2.3 Hz, 1H), 5.99 (dd, *J* = 8.3, 2.4 Hz, 1H), 3.13 (s, 1H), 2.76 (s, 3H), 1.62 (s, 6H);

MS (APCI, MeOH): *m/z* $[M+H]^+$ calcd for $C_{10}H_{13}NO_2$: 180.1, found: 180.2.

3-(Benzo[*d*][1,3]dioxol-5-yl(methyl)amino)-4-methoxycyclobut-3-ene-1,2-dione (Intermediate 15)

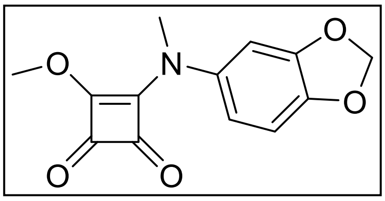

3,4-Dimethoxycyclobut-3-ene-1,2-dione (0.83 g, 5.84 mmol) was dissolved in methanol (100 mL) followed by addition of **Intermediate 13** (0.88 g, 5.84 mmol). The reaction was carried out as described in General Procedure 2, which gave **Intermediate 15** as a grey solid in 0.89 g (58 %, 3.39 mmol) yield.

^{1}**H-NMR** (402 MHz, DMSO-*d*$_6$) δ 6.98 (d, *J* = 2.2 Hz, 1H), 6.88 (d, *J* = 8.3 Hz, 1H), 6.78 (dd, *J* = 8.3, 2.3 Hz, 1H), 6.03 (s, 2H), 4.22 (s, 3H), 3.52 (s, 3H);

MS (APCI, MeOH): *m/z* $[M+H]^+$ calcd for $C_{13}H_{11}NO_5$: 262.1, found: 262.2.

3-((2,2-Dimethylbenzo[*d*][1,3]dioxol-5-yl)(methyl)amino)-4-methoxycyclobut-3-ene-1,2-dione (Intermediate 16)

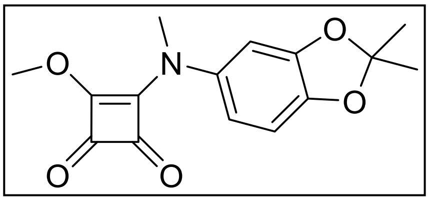

3,4-Dimethoxycyclobut-3-ene-1,2-dione (0.19 g, 1.34 mmol) was dissolved in methanol (50 mL) followed by addition of **Intermediate 14** (0.24 g, 1.34 mmol). The reaction was carried out as described in General Procedure 2, which gave **Intermediate 16** as a brown solid in 0.24 g (63 %, 0.85 mmol) yield.

^{1}**H-NMR** (402 MHz, DMSO-*d*$_6$) δ 6.90 (d, *J* = 2.2 Hz, 1H), 6.80 (d, *J* = 8.3 Hz, 1H), 6.76 – 6.71 (m, 1H), 4.23 (s, 3H), 3.52 (s, 3H), 1.63 (s, 6H);

MS (APCI, MeOH): *m/z* $[M+H]^+$ calcd for $C_{15}H_{15}NO_5$: 290.1, found: 290.3.

b. Monoamino substituted SQAs

3-(4-Morpholinophenyl)-4-((pyridin-2-ylmethyl)-amino)cyclobut-3-ene-1,2-dione (SQ31f) [5]

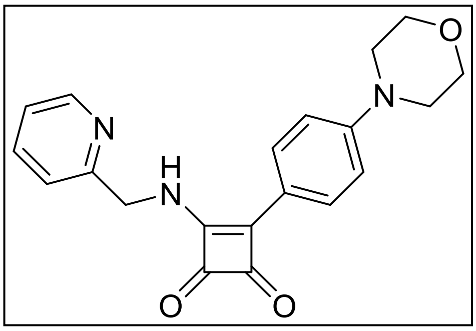

Intermediate 1 (0.27 g, 1.00 mmol) was dissolved in methanol (100 mL) followed by addition of pyridin-2-ylmethanamine (0.11 g, 0.10 mL, 1.00 mmol). The reaction was carried out as described in General Procedure 1, which gave **SQ31f** as a yellow solid in 0.29 g (84 %, 0.84 mmol) yield.

^{1}H-NMR (400 MHz, DMSO-d_6) δ 9.36 (t, *J* = 6.3 Hz, 1H), 8.53 (ddd, *J* = 4.9, 1.8, 0.9 Hz, 1H), 7.98 – 7.89 (m, 2H), 7.79 (td, *J* = 7.7, 1.8 Hz, 1H), 7.41 (dt, *J* = 7.8, 1.0 Hz, 1H), 7.30 (ddd, *J* = 7.6, 4.8, 1.1 Hz, 1H), 7.08 – 6.99 (m, 2H), 4.99 (d, *J* = 6.2 Hz, 2H), 3.78 – 3.68 (m, 4H), 3.27 (t, *J* = 4.9 Hz, 4H);

^{13}C-NMR (101 MHz, DMSO-d_6) δ 192.22, 189.43, 178.51, 163.32, 157.85, 152.75, 149.68, 137.48, 128.41, 123.10, 122.01, 119.77, 114.34, 66.30, 49.25, 47.38;

MP: 203-204 °C

HRMS (ESI, HRMS): *m/z* [M+H]$^+$ calcd for $C_{20}H_{19}N_3O_3$: 350.1499, found: 350.1497;

HPLC purity: 98 %.

3-(4-Morpholinophenyl)-4-((2-(pyridin-2-yl)ethyl)amino)cyclobut-3-ene-1,2-dione (PRP001)

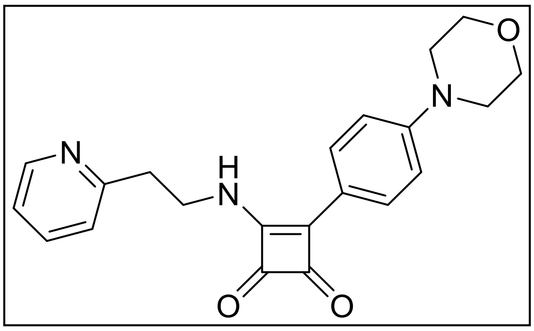

Intermediate 1 (0.27 g, 1.00 mmol) was dissolved in methanol (100 mL) followed by addition of 2-(pyridin-2-yl)ethan-1-amine (0.12 g, 0.12 mL, 1.00 mmol). The reaction was carried out as described in General Procedure 1, which gave **PRP001** as a yellow solid in 0.18 g (76 %, 0.76 mmol) yield.

^{1}H-NMR (500 MHz, DMSO-d_6) δ 8.93 (t, *J* = 6.0 Hz, 1H), 8.48 (dt, *J* = 4.6, 1.4 Hz, 1H), 7.91 – 7.84 (m, 2H), 7.70 (td, *J* = 7.6, 1.9 Hz, 1H), 7.28 (d, *J* = 7.8 Hz, 1H), 7.21 (ddd, *J* = 7.5, 4.9, 1.1 Hz, 1H),

7.08 – 7.01 (m, 2H), 4.05 (q, *J* = 6.9 Hz, 2H), 3.77 – 3.70 (m, 4H), 3.27 (t, *J* = 4.9 Hz, 4H), 3.10 (t, *J* = 7.3 Hz, 2H);

^{13}C-NMR (126 MHz, DMSO-*d*$_6$) δ 192.02, 189.19, 178.04, 162.87, 158.60, 152.66, 149.58, 136.99, 128.19, 123.78, 122.16, 119.83, 114.36, 66.32, 47.41, 44.34, 39.21;

MP: decomposition observed ≥ 200 °C;

HRMS (ESI, HRMS): *m/z* [M+H]$^+$ calcd for $C_{21}H_{21}N_3O_3$: 364.1656, found: 364.1652;

HPLC purity: 97 %.

3-(((1H-Pyrrol-2-yl)methyl)amino)-4-(4-morpholinophenyl)cyclobut-3-ene-1,2-dione (PRP002)

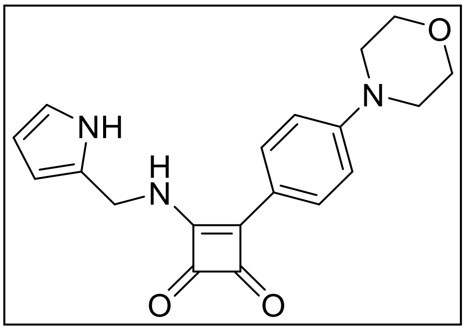

Intermediate 1 (0.27 g, 1.00 mmol) was dissolved in methanol (100 mL) followed by addition of 2-(pyridin-2-yl)ethan-1-amine hydrochloride (0.13 g, 1.00 mmol) and *N*,*N*-diisopropylethylamine (0.13 g, 0.18 mL, 1.00 mmol). The reaction was carried out as described in General Procedure 1, which gave **PRP002** as a brown solid in 0.24 g (70 %, 0.70 mmol) yield.

^{1}H-NMR (600 MHz, DMSO-*d*$_6$) δ 10.77 (s, 1H), 9.22 (s, 1H), 7.94 – 7.89 (m, 2H), 7.04 – 6.98 (m, 2H), 6.69 (q, *J* = 2.4 Hz, 1H), 5.96 (ddt, *J* = 13.9, 5.7, 2.7 Hz, 2H), 4.86 (s, 2H), 3.73 – 3.68 (m, 4H), 3.24 (t, *J* = 4.9 Hz, 4H);

^{13}C-NMR (151 MHz, DMSO-*d*$_6$) δ 192.40, 189.23, 177.58, 162.83, 152.62, 128.34, 128.24, 119.89, 118.48, 114.28, 107.87, 107.09, 66.30, 47.38, 41.41;

MP: decomposition observed ≥ at 230 °C;

HRMS (ESI, HRMS): *m/z* [M+H]$^+$ calcd for $C_{19}H_{19}N_3O_3$: 338.1499, found: 338.1498;

HPLC purity: 97 %.

3-(4-Morpholinophenyl)-4-((thiazol-4-ylmethyl)amino)cyclobut-3-ene-1,2-dione (PRP003)

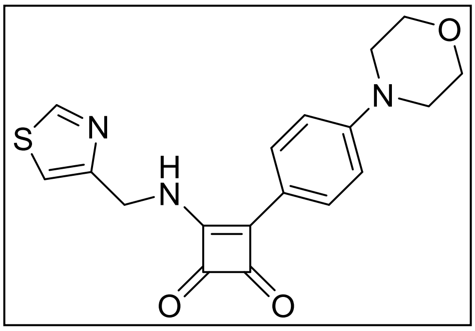

Intermediate 1 (0.27 g, 1.00 mmol) was dissolved in methanol (100 mL) followed by addition of thiazol-4-ylmethanamine hydrochloride (0.15 g, 1.00 mmol) and *N*,*N*-diisopropylethylamine (0.13 g, 0.18 mL, 1.00 mmol). The reaction was carried out as described in General Procedure 1, which gave **PRP003** as a yellow solid in 0.30 g (85 %, 0.85 mmol) yield.

^{1}H-NMR (600 MHz, DMSO-*d*$_6$) δ 9.39 (s, 1H), 9.07 (d, *J* = 2.0 Hz, 1H), 7.98 – 7.87 (m, 2H), 7.61 (d, *J* = 1.9 Hz, 1H), 7.07 – 6.98 (m, 2H), 5.01 (s, 2H), 3.77 – 3.66 (m, 4H), 3.31 – 3.19 (m, 4H);

^{13}C-NMR (151 MHz, DMSO-*d*$_6$) δ 192.10, 189.39, 178.18, 163.40, 155.22, 154.36, 152.74, 128.42, 119.71, 116.91, 114.30, 66.29, 47.37, 44.05;

MP: decomposition observed ≥ 246 °C;

HRMS (ESI, HRMS): *m/z* [M+H]$^+$ calcd for $C_{18}H_{17}N_3O_3S$: 356.1063, found: 356.1062;

HPLC purity: 99 %.

3-((Benzo[*d*][1,3]dioxol-5-ylmethyl)amino)-4-(4-morpholinophenyl)cyclobut-3-ene-1,2-dione (PRP004)

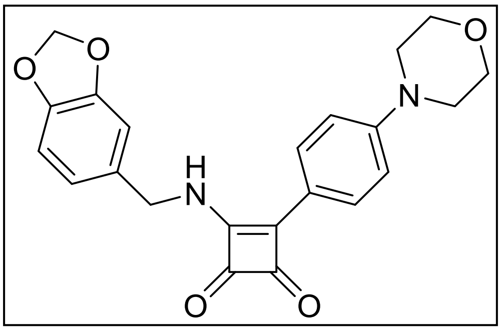

Intermediate 1 (0.27 g, 1.00 mmol) was dissolved in methanol (100 mL) followed by addition of benzo[*d*][1,3]dioxol-5-ylmethanamine (0.15 g, 0.12 mL, 1.00 mmol). The reaction was carried out as described in General Procedure 1, which gave **PRP004** as a grey solid in 0.24 g (61 %, 0.61 mmol) yield.

^{1}H-NMR (500 MHz, DMSO-*d*$_6$) δ 9.27 (t, *J* = 6.2 Hz, 1H), 7.95 – 7.88 (m, 2H), 7.06 – 7.02 (m, 2H), 6.94 (d, *J* = 1.7 Hz, 1H), 6.89 (d, *J* = 7.9 Hz, 1H), 6.84 (dd, *J* = 8.0, 1.7 Hz, 1H), 5.99 (s, 2H), 4.78 (d, *J* = 6.2 Hz, 2H), 3.76 – 3.70 (m, 4H), 3.27 (t, *J* = 4.9 Hz, 4H);

^{13}C-NMR (126 MHz, DMSO-*d*$_6$) δ 192.31, 189.16, 177.62, 163.42, 152.75, 147.90, 147.09, 132.72, 128.37, 121.53, 119.75, 114.32, 108.74, 108.66, 101.47, 66.30, 47.62, 47.37;

MP: decomposition observed ≥ 269 °C;

HRMS (ESI, HRMS): *m/z* $[M+H]^+$ calcd for $C_{22}H_{20}N_2O_5$: 393.1445, found: 393.1442;

HPLC purity: 98 %.

C. Diamino substituted SQAs

3-(Benzo[*d*][1,3]dioxol-5-ylamino)-4-((pyridin-2-yl-methyl)amino)cyclobut-3-ene-1,2-dione (SQ6ab) [1]

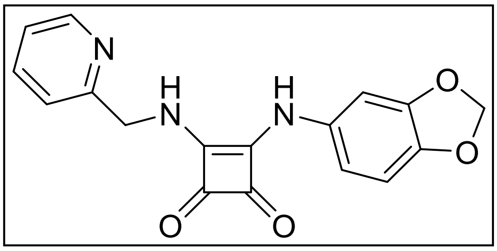

Intermediate 4 (0.25 g, 1.00 mmol) was dissolved in methanol (100 mL) followed by addition of pyridin-2-ylmethanamine (0.11 g, 0.10 mL, 1.00 mmol). The reaction was carried out as described in General Procedure 3, which gave **SQ6ab** as a light brown solid in 0.31 g (95 %, 0.95 mmol) yield.

^{1}H-NMR (600 MHz, DMSO-d_6) δ 9.74 (s, 1H), 8.63 – 8.52 (m, 1H), 8.09 (s, 1H), 7.81 (td, *J* = 7.7, 1.8 Hz, 1H), 7.41 (d, *J* = 7.8 Hz, 1H), 7.33 (ddd, *J* = 7.5, 4.8, 1.1 Hz, 1H), 7.23 (s, 1H), 6.86 (d, *J* = 8.3 Hz, 1H), 6.73 (dd, *J* = 8.3, 2.3 Hz, 1H), 5.98 (s, 2H), 4.91 (d, *J* = 6.2 Hz, 2H);

^{13}C-NMR (151 MHz, DMSO-d_6) δ 183.92, 181.11, 169.19, 164.06, 157.45, 149.63, 148.31, 143.42, 137.65, 134.07, 123.20, 122.16, 111.30, 108.93, 101.63, 101.05, 48.92;

MP: 216 - 219 °C

HRMS (ESI, HRMS): *m/z* $[M+H]^+$ calcd for $C_{17}H_{13}N_3O_4$: 324.0979, found: 324.0978;

HPLC purity: 96 %.

3-((4-Morpholinophenyl)amino)-4-((pyrazin-2-ylmethyl)amino)cyclobut-3-ene-1,2-dione (PRP005)

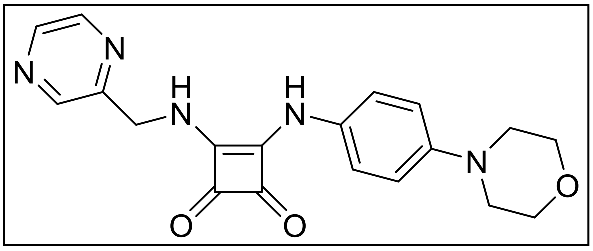

Intermediate 2 (0.29 g, 1.00 mmol) was dissolved in methanol (100 mL) followed by addition of pyrazin-2-ylmethanamine (0.11 g, 0.10 mL, 1.00 mmol). The reaction was carried out as described in General Procedure 3, which gave **PRP005** as a light brown solid in 0.28 g (77 %, 0.77 mmol) yield.

^{1}H-NMR (400 MHz, DMSO-*d*$_6$) δ 9.64 (s, 1H), 8.83 – 8.47 (m, 3H), 8.00 (s, 1H), 7.28 (d, *J* = 8.5 Hz, 2H), 6.97 – 6.83 (m, 2H), 4.98 (d, *J* = 4.8 Hz, 2H), 3.71 (t, *J* = 4.8 Hz, 4H), 3.03 (t, *J* = 4.8 Hz, 4H);

^{13}C-NMR (101 MHz, DMSO-*d*$_6$) δ 183.73, 181.43, 169.12, 164.39, 153.47, 147.65, 144.61, 144.23, 144.00, 131.57, 119.87, 116.49, 66.54, 49.33, 46.71;

MP: decomposition observed ≥ 279 °C;

HRMS (ESI, HRMS): *m/z* [M+H]$^+$ calcd for $C_{19}H_{19}N_5O_3$: 366.1561, found: 366.1556;

HPLC purity: 95 %.

3-(((1*H*-Imidazol-2-yl)methyl)amino)-4-((4-morpholinophenyl)amino)cyclobut-3-ene-1,2-dione (PRP006)

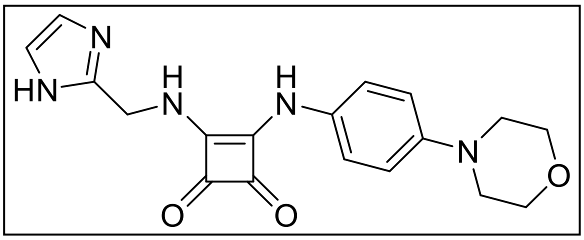

Intermediate 2 (0.29 g, 1.00 mmol) was dissolved in methanol (100 mL) followed by addition of (1*H*-imidazol-2-yl)methanamine dihydrochloride (0.17 g, 1.00 mmol) and *N*,*N*-diisopropylethylamine (0.26 g, 0.36 mL, 2.00 mmol). The reaction was carried out as described in General Procedure 3, which gave **PRP006** as a yellow solid in 0.23 g (66 %, 0.66 mmol) yield.

^{1}H-NMR (600 MHz, DMSO-*d*$_6$) δ 12.09 (s, 1H), 9.58 (s, 1H), 7.89 (s, 1H), 7.29 (d, *J* = 8.6 Hz, 2H), 7.00 (s, 2H), 6.92 (d, *J* = 8.7 Hz, 2H), 4.82 (s, 2H), 3.71 (t, *J* = 4.8 Hz, 4H), 3.03 (t, *J* = 4.8 Hz, 4H);

^{13}C-NMR (151 MHz, DMSO-*d*$_6$) δ 183.69, 181.08, 168.76, 164.14, 147.57, 144.33, 131.76, 120.05, 119.66, 116.55, 116.44, 66.55, 49.37, 41.51;

MP: decomposition observed ≥ 312 °C;

HRMS (ESI, HRMS): *m/z* [M+H]$^+$ calcd for $C_{18}H_{19}N_5O_3$: 354.1561, found: 354.1562;

HPLC purity: 96 %.

3-((2-(1*H*-Imidazol-4-yl)ethyl)amino)-4-((4-morpholinophenyl)amino)cyclobut-3-ene-1,2-dione (PRP007)

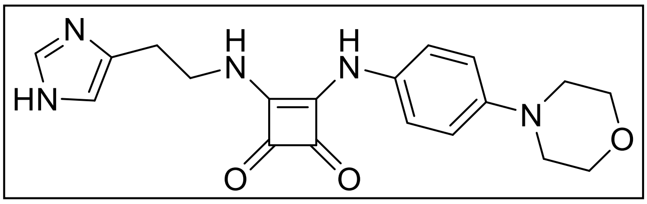

Intermediate 2 (0.29 g, 1.00 mmol) was dissolved in methanol (100 mL) followed by addition of 2-(1*H*-imidazol-4-yl)ethan-1-amine dihydrochloride (0.18 g, 1.00 mmol) and *N*,*N*-diisopropylethylamine (0.26 g, 0.36 mL, 2.00 mmol). The reaction was carried out as described in General Procedure 3, which gave **PRP007** as a yellow solid in 0.23 g (57 %, 0.57 mmol) yield.

^{1}H-NMR (600 MHz, DMSO-*d*$_6$) δ 11.92 (s, 1H), 9.55 (s, 1H), 7.59 (s, 1H), 7.51 (s, 1H), 7.26 (d, *J* = 8.4 Hz, 2H), 6.90 (d, *J* = 8.7 Hz, 2H), 6.86 (s, 1H), 3.84 (d, *J* = 6.8 Hz, 2H), 3.71 (t, *J* = 4.8 Hz, 4H), 3.03 (t, *J* = 4.8 Hz, 4H), 2.79 (t, *J* = 6.7 Hz, 2H);

^{13}C-NMR (151 MHz, DMSO-*d*$_6$) δ 183.73, 180.91, 169.10, 163.99, 147.52, 135.31, 131.71, 120.02, 119.67, 116.51, 116.44, 66.55, 49.37, 43.86, 29.12;

MP: decomposition observed ≥ 297 °C;

HRMS (ESI, HRMS): *m/z* [M+H]$^+$ calcd for $C_{19}H_{21}N_5O_3$: 368.1717, found: 368.1710;

HPLC purity: 97 %.

3-(((1H-Benzo[d]imidazol-2-yl)methyl)amino)-4-((4-morpholinophenyl)amino)cyclobut-3-ene-1,2-dione (PRP008)

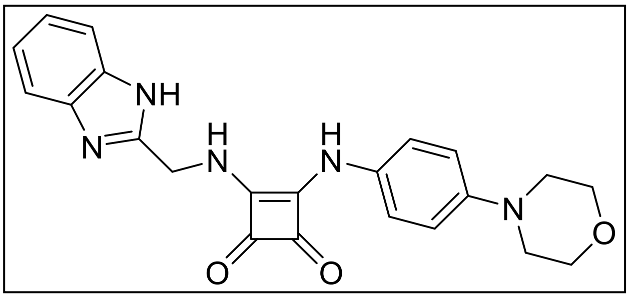

Intermediate 2 (0.29 g, 1.00 mmol) was dissolved in methanol (100 mL) followed by addition of (1*H*-benzo[d]imidazol-2-yl)methanamine dihydrochloride (0.22 g, 1.00 mmol) *N*,*N*-diisopropylethylamine (0.26 g, 0.36 mL, 2.00 mmol). The reaction was carried out as described in General Procedure 3, which gave **PRP008** as a grey solid in 0.30 g (74 %, 0.74 mmol) yield.

^{1}H-NMR (400 MHz, DMSO-*d*$_6$) δ 12.48 (s, 1H), 9.74 (s, 1H), 8.18 – 8.02 (m, 1H), 7.59 (d, J = 7.4 Hz, 1H), 7.47 (d, J = 7.3 Hz, 1H), 7.35 – 7.28 (m, 2H), 7.21 – 7.12 (m, 2H), 6.96 – 6.88 (m, 2H), 5.11 – 5.01 (m, 2H), 3.71 (dd, J = 6.0, 3.5 Hz, 4H), 3.04 (dd, J = 5.8, 3.8 Hz, 4H);

^{13}C-NMR (101 MHz, DMSO-*d*$_6$) δ 183.72, 181.39, 168.96, 164.34, 151.71, 147.63, 143.36, 134.83, 131.66, 122.71, 121.83, 119.76, 118.93, 116.55, 111.84, 66.55, 49.34, 42.15;

MP: decomposition observed ≥ 314 °C;

HRMS (ESI, HRMS): *m/z* [M+H]$^+$ calcd for $C_{22}H_{21}N_5O_3$: 404.1717, found: 404.1716;

HPLC purity: 95 %.

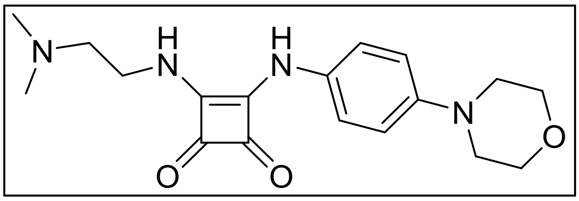

3-((2-(Dimethylamino)ethyl)amino)-4-((4-morpholinophenyl)amino)cyclobut-3-ene-1,2-dione (PRP009)

Intermediate 2 (0.29 g, 1.00 mmol) was dissolved in methanol (100 mL) followed by addition of N^1,N^1-dimethylethane-1,2-diamine (0.09 g, 0.11 mL, 1.00 mmol). The reaction was carried out as described in General Procedure 3, which gave **PRP009** as a grey solid in 0.30 g (87 %, 0.87 mmol) yield.

^{1}H-NMR (400 MHz, DMSO-d_6) δ 9.63 (s, 1H), 7.54 (s, 1H), 7.29 (d, *J* = 8.5 Hz, 2H), 6.96 – 6.85 (m, 2H), 3.80 – 3.61 (m, 6H), 3.03 (dd, *J* = 5.8, 3.8 Hz, 4H), 2.42 (t, *J* = 5.8 Hz, 2H), 2.18 (s, 6H);

^{13}C-NMR (101 MHz, DMSO-d_6) δ 183.81, 180.79, 169.03, 163.90, 147.49, 131.84, 119.61, 116.55, 66.55, 59.50, 49.39, 45.49, 41.76;

MP: decomposition observed ≥ 283 °C;

HRMS (ESI, HRMS): *m/z* $[M+H]^+$ calcd for $C_{18}H_{24}N_4O_3$: 345.1921, found: 345.1917;

HPLC purity: 98 %.

3-((4-(1H-Imidazol-1-yl)phenyl)amino)-4-((pyridin-2 ylmethyl)amino)cyclobut-3-ene-1,2-dione (PRP010)

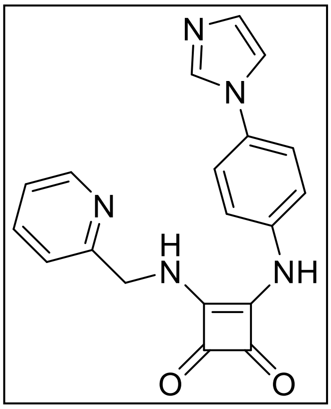

Intermediate 3 (0.22 g, 1.00 mmol) was dissolved in methanol (100 mL) followed by addition of 4-(1*H*-imidazol-1-yl)aniline (0.16 g, 1.00 mmol). The reaction was carried out as described in General Procedure 3, which gave **PRP010** as a light grey solid in 0.32 g (94 %, 0.94 mmol) yield.

^{1}H-NMR (402 MHz, DMSO-d_6) δ 9.96 (s, 1H), 8.57 (dt, *J* = 4.7, 1.4 Hz, 1H), 8.23 (s, 1H), 8.16 (t, *J* = 1.1 Hz, 1H), 7.81 (td, *J* = 7.7, 1.8 Hz, 1H), 7.65 (t, *J* = 1.4 Hz, 1H), 7.62 – 7.46 (m, 4H), 7.44 – 7.38 (m, 1H), 7.32 (ddd, *J* = 7.6, 4.8, 1.1 Hz, 1H), 7.06 (t, *J* = 1.1 Hz, 1H), 4.92 (d, *J* = 4.5 Hz, 2H);

^{13}C-NMR (101 MHz, DMSO-d_6) δ 184.54, 181.03, 169.64, 163.91, 157.34, 149.63, 138.23, 137.66, 135.82, 132.29, 130.13, 123.22, 122.18, 121.89, 119.54, 118.45, 48.93;

MP: decomposition observed ≥ 243 °C;

HRMS (ESI, HRMS): *m/z* $[M+H]^+$ calcd for $C_{19}H_{15}N_5O_2$: 346.1299, found: 346.1303;

HPLC purity: 96 %.

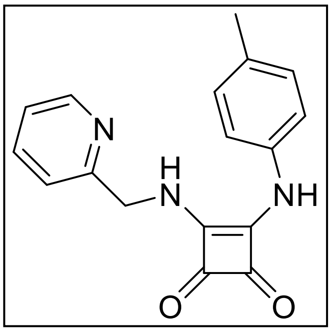

3-((Pyridin-2-ylmethyl)amino)-4-(p-tolylamino)cyclobut-3-ene-1,2-dione (PRP011)

Intermediate 3 (0.22 g, 1.00 mmol) was dissolved in methanol (100 mL) followed by addition of *p*-toluidine (0.11 g, 1.00 mmol). The reaction was carried out as described in General Procedure 3, which gave **PRP011** as a light grey solid in 0.29 g (98 %, 0.98 mmol) yield.

^{1}H-NMR (402 MHz, DMSO-d_6) δ 9.72 (s, 1H), 8.63 – 8.52 (m, 1H), 8.10 (s, 1H), 7.80 (td, *J* = 7.7, 1.8 Hz, 1H), 7.40 (d, *J* = 7.7 Hz, 1H), 7.36 – 7.25 (m, 3H), 7.11 (d, *J* = 8.1 Hz, 2H), 4.89 (d, *J* = 6.0 Hz, 2H), 2.22 (s, 3H);

^{13}C-NMR (101 MHz, DMSO-d_6) δ 181.07, 173.49, 169.34, 164.29, 157.45, 149.62, 137.64, 136.96, 132.18, 130.15, 123.19, 122.15, 118.58, 48.90, 20.75;

MP: decomposition observed ≥ 235 °C;

HRMS (ESI, HRMS): *m/z* $[M+H]^+$ calcd for $C_{17}H_{15}N_3O_2$: 294.1237, found: 294.1234;

HPLC purity: 98 %.

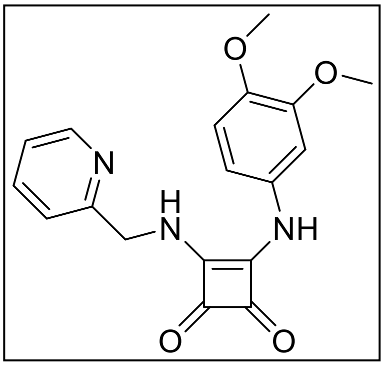

3-((3,4-Dimethoxyphenyl)amino)-4-((pyridin-2-ylmethyl)amino)cyclobut-3-ene-1,2-dione (PRP012)

Intermediate 3 (0.22 g, 1.00 mmol) was dissolved in methanol (100 mL) followed by addition of 3,4-dimethoxyaniline (0.15 g, 1.00 mmol). The reaction was carried out as described in General Procedure 3, which gave **PRP012** as a light brown solid in 0.26 g (75 %, 0.75 mmol) yield.

^{1}H-NMR (402 MHz, DMSO-d_6) δ 9.75 (s, 1H), 8.61 – 8.50 (m, 1H), 8.10 (s, 1H), 7.79 (td, *J* = 7.7, 1.8 Hz, 1H), 7.39 (d, *J* = 7.8 Hz, 1H), 7.31 (dd, *J* = 7.5, 5.0 Hz, 1H), 7.28 – 7.18 (m, 1H), 6.88 (d, *J* = 8.7 Hz, 1H), 6.79 (dd, *J* = 8.6, 2.5 Hz, 1H), 4.90 (s, 2H), 3.72 (s, 3H), 3.69 (s, 3H);

^{13}**C-NMR** (101 MHz, DMSO-d_6) δ 183.84, 181.29, 169.15, 164.16, 157.48, 149.79, 149.60, 145.19, 137.61, 133.11, 123.17, 122.14, 113.15, 110.10, 104.05, 56.28, 55.85, 48.90;

MP: decomposition observed ≥ 248 °C;

HRMS (ESI, HRMS): *m/z* $[M+H]^+$ calcd for $C_{18}H_{17}N_3O_4$: 340.1292, found: 340.1288;

HPLC purity: 95 %.

3-((Benzo[*d*][1,3]dioxol-5-ylmethyl)amino)-4-((pyridin-2-ylmethyl)amino)cyclobut-3-ene-1,2-dione (PRP013)

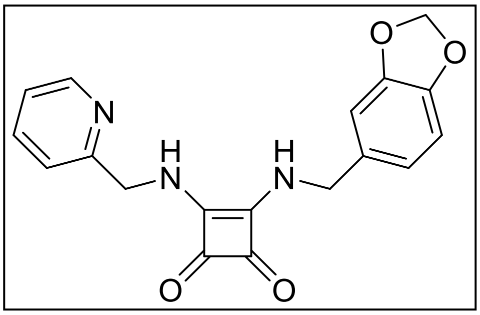

Intermediate 3 (0.22 g, 1.00 mmol) was dissolved in methanol (100 mL) followed by addition of benzo[*d*][1,3]dioxol-5-ylmethanamine (0.15 g, 0.13 mL, 1.00 mmol). The reaction was carried out as described in General Procedure 3, which gave **PRP013** as a yellow solid in 0.16 g (47 %, 0.47 mmol) yield.

^{1}**H-NMR** (600 MHz, DMSO-d_6) δ 8.53 (d, *J* = 4.8 Hz, 1H), 7.86 (s, 2H), 7.78 (td, *J* = 7.7, 1.7 Hz, 1H), 7.34 (d, *J* = 7.8 Hz, 1H), 7.32 – 7.27 (m, 1H), 6.92 – 6.84 (m, 2H), 6.80 (d, *J* = 8.0 Hz, 1H), 5.98 (d, *J* = 0.9 Hz, 2H), 4.81 (s, 2H), 4.60 (d, *J* = 6.0 Hz, 2H);

^{13}**C-NMR** (151 MHz, DMSO-d_6) δ 183.29, 183.05, 167.86, 157.93, 149.59, 147.90, 146.99, 137.54, 133.23, 123.07, 122.03, 121.42, 108.76, 108.57, 101.43, 48.70, 47.03, 40.53;

MP: 204 - 209 °C;

HRMS (ESI, HRMS): *m/z* $[M+H]^+$ calcd for $C_{18}H_{15}N_3O_4$: 338.1135, found: 338.1139;

HPLC purity: 98 %.

3-(((1*H*-Imidazol-2-yl)methyl)amino)-4-(benzo[*d*][1,3]dioxol-5-ylamino)cyclobut-3-ene-1,2-dione (PRP014)

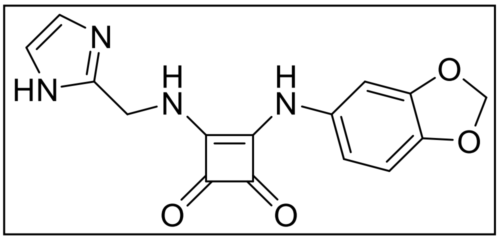

Intermediate 4 (0.25 g, 1.00 mmol) was dissolved in methanol (100 mL) followed by addition of (1*H*-imidazol-2-yl)methanamine dihydrochloride (0.17 g, 1.00 mmol) and *N*,*N*-diisopropylethylamine (0.26 g, 0.36 mL, 2.00 mmol). The reaction

was carried out as described in General Procedure 3, which gave **PRP014** as a brown solid in 0.26 g (83 %, 0.83 mmol) yield.

^{1}H-NMR (402 MHz, DMSO-*d*$_6$) δ 12.08 (s, 1H), 9.64 (s, 1H), 7.93 (s, 1H), 7.25 – 7.14 (m, 1H), 6.99 (s, 2H), 6.84 (d, *J* = 8.3 Hz, 1H), 6.71 (dd, *J* = 8.3, 2.3 Hz, 1H), 5.96 (s, 2H), 4.80 (s, 2H);

^{13}C-NMR (101 MHz, DMSO-*d*$_6$) δ 183.82, 181.02, 168.92, 165.39, 163.94, 148.30, 144.23, 143.36, 134.12, 111.17, 108.93, 101.61, 100.93, 41.57;

MP: decomposition observed ≥ 284 °C;

HRMS (ESI, HRMS): *m/z* [M+H]$^+$ calcd for $C_{15}H_{12}N_4O_4$: 313.0931, found: 313.0931;

HPLC purity: 96 %.

3-((2-(1*H*-Imidazol-4-yl)ethyl)amino)-4-(benzo[*d*][1,3]dioxol-5-ylamino)cyclobut-3-ene-1,2-dione (PRP015)

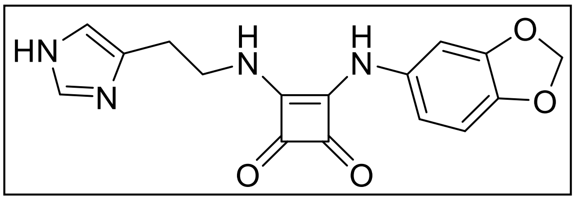

Intermediate 4 (0.25 g, 1.00 mmol) was dissolved in methanol (100 mL) followed by addition of 2-(1*H*-imidazol-4-yl)ethan-1-amine dihydrochloride (0.18 g, 1.00 mmol) and *N*,*N*-diisopropylethylamine (0.26 g, 0.36 mL, 2.00 mmol). The reaction was carried out as described in General Procedure 3, which gave **PRP015** as a green solid in 0.29 g (88 %, 0.88 mmol) yield.

^{1}H-NMR (600 MHz, DMSO-*d*$_6$) δ 11.88 (s, 1H), 9.59 (s, 1H), 7.61 – 7.45 (m, 2H), 7.18 (s, 1H), 6.88 – 6.79 (m, 2H), 6.67 (dd, *J* = 8.3, 2.2 Hz, 1H), 5.95 (s, 2H), 3.81 (q, *J* = 6.5 Hz, 2H), 2.77 (t, *J* = 6.7 Hz, 2H);

^{13}C-NMR (151 MHz, DMSO-*d*$_6$) δ 183.96, 180.88, 169.32, 165.51, 163.88, 148.37, 143.43, 135.40, 134.17, 116.81, 111.30, 108.99, 101.69, 101.09, 43.99, 29.18;

MP: decomposition observed ≥ 269 °C;

HRMS (ESI, HRMS): *m/z* [M+H]$^+$ calcd for $C_{16}H_{14}N_4O_4$: 327.1088, found: 327.1082;

HPLC purity: 95 %.

3-(((1*H*-Benzo[d]imidazol-2-yl)methyl)amino)-4-(benzo[*d*][1,3]dioxol-5-ylamino)cyclobut-3-ene-1,2-dione (PRP016)

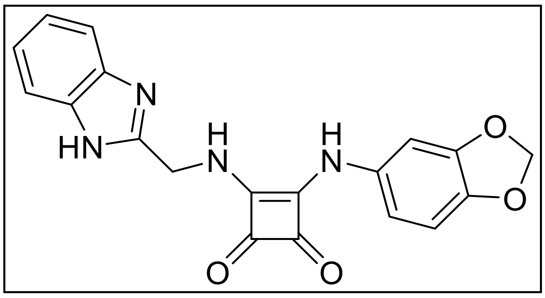

Intermediate 4 (0.25 g, 1.00 mmol) was dissolved in methanol (100 mL) followed by addition of (1H-benzo[d]imidazol-2-yl)methanamine dihydrochloride (0.22 g, 1.00 mmol) and *N*,*N*-diisopropylethylamine (0.26 g, 0.36 mL, 2.00 mmol). The reaction was carried out as described in General Procedure 3, which gave **PRP016** as a brown solid in 0.24 g (67 %, 0.67 mmol) yield.

^{1}H-NMR (402 MHz, DMSO-d_6) δ 12.47 (s, 1H), 9.79 (s, 1H), 8.12 (s, 1H), 7.52 (s, 2H), 7.22 (d, *J* = 2.3 Hz, 1H), 7.19 – 7.05 (m, 2H), 6.86 (d, *J* = 8.3 Hz, 1H), 6.73 (dd, *J* = 8.3, 2.3 Hz, 1H), 5.97 (s, 2H), 5.05 (d, *J* = 4.5 Hz, 2H);

^{13}C-NMR (101 MHz, DMSO-d_6) δ 183.87, 181.26, 169.05, 165.38, 164.15, 151.61, 148.33, 143.45, 134.03, 122.23, 118.68, 111.30, 108.96, 101.64, 101.02, 42.15;

MP: decomposition observed ≥ 288 °C;

HRMS (ESI, HRMS): *m/z* [M+H]$^+$ calcd for $C_{19}H_{14}N_4O_4$: 363.1088, found: 363.1085;

HPLC purity: 95 %.

3-(Benzo[*d*][1,3]dioxol-5-ylamino)-4-(((2-phenyl-1*H*-imidazol-4-yl)methyl)amino)cyclobut-3-ene-1,2-dione (PRP017)

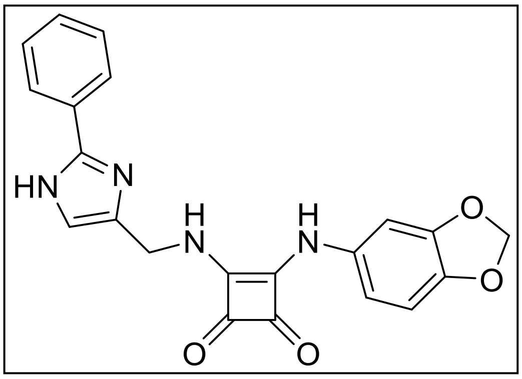

Intermediate 4 (0.25 g, 1.00 mmol) was dissolved in methanol (100 mL) followed by addition of (2-phenyl-1H-imidazol-4-yl)methanamine (0.17 g, 1.00 mmol). The reaction was carried out as described in General Procedure 3, which gave **PRP017** as a white solid in 0.32 g (82 %, 0.82 mmol) yield.

^{1}H-NMR (402 MHz, DMSO-d_6) δ 12.22 (s, 1H), 9.65 (s, 1H), 7.95 (s, 1H), 7.81 – 7.64 (m, 2H), 7.59 (d, *J* = 13.0 Hz, 1H), 7.31 (t, *J* = 7.6 Hz, 2H), 7.24 – 7.10 (m, 2H), 6.84 (d, *J* = 8.3 Hz, 1H), 6.71 (dd, *J* = 8.3, 2.3 Hz, 1H), 5.97 (s, 2H), 4.86 (s, 2H);

^{13}C-NMR (101 MHz, DMSO-d_6) δ 183.85, 181.09, 168.87, 164.00, 148.31, 144.87, 143.41, 140.59, 134.99, 134.03, 128.87, 126.55, 124.69, 113.72, 111.26, 108.93, 101.63, 100.99, 41.62;

MP: decomposition observed ≥ 273 °C;

HRMS (ESI, HRMS): *m/z* $[M+H]^+$ calcd for $C_{21}H_{16}N_4O_4$: 389.1244, found: 389.1239;

HPLC purity: 98 %.

3-(Benzo[*d*][1,3]dioxol-5-ylamino)-4-((oxazol-4-ylmethyl)amino)cyclobut-3-ene-1,2-dione (PRP018)

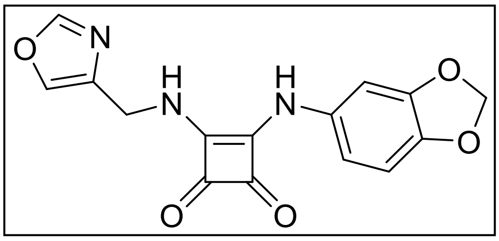

Intermediate 4 (0.25 g, 1.00 mmol) was dissolved in methanol (100 mL) followed by addition of oxazol-4-ylmethanamine hydrochloride (0.13 g, 1.00 mmol) and *N*,*N*-diisopropylethylamine (0.13 g, 0.18 mL, 1.00 mmol). The reaction was carried out as described in General Procedure 3, which gave **PRP018** as a white solid in 0.27 g (86 %, 0.86 mmol) yield.

^{1}H-NMR (502 MHz, DMSO-d_6) δ 9.57 (s, 1H), 8.39 (d, *J* = 1.0 Hz, 1H), 8.08 (d, *J* = 1.1 Hz, 1H), 7.88 (s, 1H), 7.29 – 7.11 (m, 1H), 6.85 (d, *J* = 8.3 Hz, 1H), 6.70 (dd, *J* = 8.3, 2.3 Hz, 1H), 5.98 (s, 2H), 4.71 (d, *J* = 5.9 Hz, 2H);

^{13}C-NMR (126 MHz, DMSO-d_6) δ 183.75, 181.06, 169.04, 164.04, 153.13, 148.31, 143.43, 137.35, 136.82, 133.99, 111.29, 108.93, 101.64, 101.00, 39.60;

MP: decomposition observed ≥ 266 °C;

HRMS (ESI, HRMS): *m/z* $[M+H]^+$ calcd for $C_{15}H_{11}N_3O_5$: 314.0772, found: 314.0769;

HPLC purity: 95 %.

3-(Benzo[*d*][1,3]dioxol-5-ylamino)-4-(((2-phenyloxazol-4-yl)methyl)amino)cyclobut-3-ene-1,2-dione (PRP019)

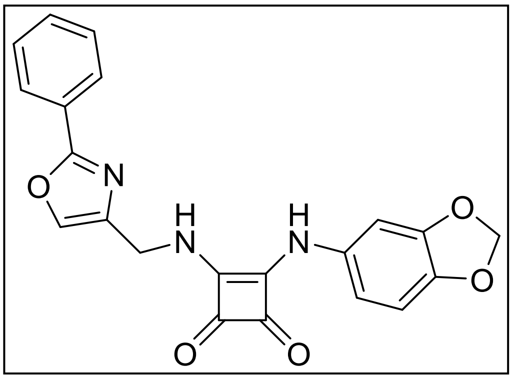

Intermediate 4 (0.25 g, 1.00 mmol) was dissolved in methanol (100 mL) followed by addition of (2-phenyloxazol-4-yl)methanamine hydrochloride (0.21 g, 1.00 mmol) and *N*,*N*-diisopropylethylamine (0.13 g, 0.18 mL, 1.00 mmol). The reaction was carried out as described in General Procedure 3, which gave **PRP019** as a white solid in 0.36 g (93 %, 0.93 mmol) yield.

^{1}H-NMR (600 MHz, DMSO-d_6) δ 9.59 (s, 1H), 8.18 (s, 1H), 7.97 (dd, *J* = 6.7, 3.0 Hz, 3H), 7.58 – 7.46 (m, 3H), 7.21 (s, 1H), 6.85 (d, *J* = 8.3 Hz, 1H), 6.71 (dd, *J* = 8.4, 2.2 Hz, 1H), 5.98 (s, 2H), 4.78 (d, *J* = 5.8 Hz, 2H);

^{13}C-NMR (151 MHz, DMSO-d_6) δ 183.79, 181.11, 169.02, 164.12, 161.58, 148.31, 143.45, 139.45, 137.33, 133.98, 131.27, 129.65, 127.10, 126.41, 111.33, 108.93, 101.64, 101.03, 40.54;

MP: decomposition observed ≥ 273 °C;

HRMS (ESI, HRMS): *m/z* $[M+H]^+$ calcd for $C_{21}H_{15}N_3O_5$: 390.1085, found: 390.1083;

HPLC purity: 99 %.

3-(Benzo[*d*][1,3]dioxol-5-ylamino)-4-((thiazol-4-ylmethyl)amino)cyclobut-3-ene-1,2-dione (PRP020)

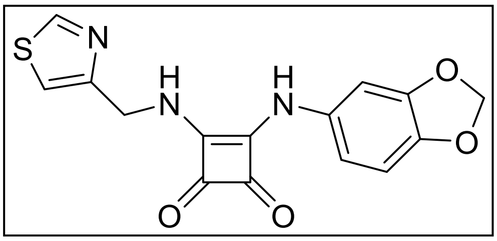

Intermediate 4 (0.25 g, 1.00 mmol) was dissolved in methanol (100 mL) followed by addition of thiazol-4-ylmethanamine dihydrochloride (0.19 g, 1.00 mmol) and *N*,*N*-diisopropylethylamine (0.26 g, 0.36 mL, 2.00 mmol). The reaction was carried out as described in General Procedure 3, which gave **PRP020** as a grey solid in 0.30 g (91 %, 0.91 mmol) yield.

^{1}H-NMR (502 MHz, DMSO-d_6) δ 9.61 (s, 1H), 9.13 (d, *J* = 1.9 Hz, 1H), 8.00 (s, 1H), 7.64 (dd, *J* = 1.9, 0.9 Hz, 1H), 7.21 (s, 1H), 6.85 (d, *J* = 8.3 Hz, 1H), 6.71 (dd, *J* = 8.3, 2.3 Hz, 1H), 5.98 (s, 2H), 4.92 (d, *J* = 6.1 Hz, 2H);

^{13}**C-NMR** (126 MHz, DMSO-d_6) δ 181.03, 169.02, 167.92, 164.03, 155.55, 154.13, 148.31, 143.42, 134.02, 116.97, 111.26, 108.93, 101.64, 101.01, 43.74;

MP: decomposition observed ≥ 257 °C;

HRMS (ESI, HRMS): *m/z* $[M+H]^+$ calcd for $C_{15}H_{11}N_3O_4S$: 330.0543, found: 330.0538;

HPLC purity: 96 %.

3-(Benzo[*d*][1,3]dioxol-5-ylamino)-4-(((2-phenylthiazol-4-yl)methyl)amino)cyclobut-3-ene-1,2-dione (PRP021)

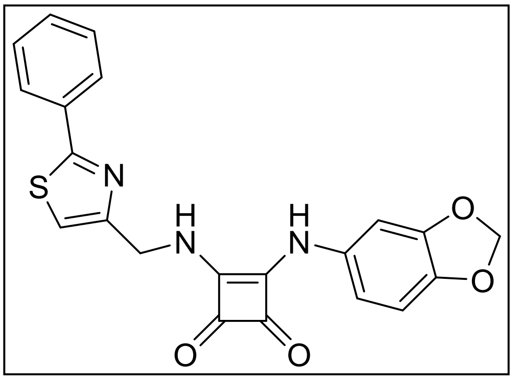

Intermediate 4 (0.25 g, 1.00 mmol) was dissolved in methanol (100 mL) followed by addition of (2-phenylthiazol-4-yl)methanamine dihydrochloride (0.26 g, 1.00 mmol) and *N*,*N*-diisopropylethylamine (0.26 g, 0.36 mL, 2.00 mmol). The reaction was carried out as described in General Procedure 3, which gave **PRP021** as a white solid in 0.37 g (90 %, 0.90 mmol) yield.

^{1}**H-NMR** (402 MHz, DMSO-d_6) δ 9.59 (s, 1H), 8.01 (s, 1H), 7.95 – 7.82 (m, 2H), 7.62 (s, 1H), 7.52 – 7.38 (m, 3H), 7.20 (s, 1H), 6.83 (d, *J* = 8.3 Hz, 1H), 6.70 (dd, *J* = 8.3, 2.3 Hz, 1H), 5.96 (s, 2H), 4.92 (d, *J* = 6.0 Hz, 2H);

^{13}**C-NMR** (101 MHz, DMSO-d_6) δ 183.81, 181.11, 169.03, 168.26, 164.10, 154.64, 148.29, 143.44, 133.97, 133.27, 130.83, 129.69, 126.55, 117.40, 111.35, 108.90, 101.63, 101.04, 44.01;

MP: 245 - 247 °C;

HRMS (ESI, HRMS): *m/z* $[M+H]^+$ calcd for $C_{21}H_{15}N_3O_4S$: 406.0856, found: 406.0855;

HPLC purity: 96 %.

3-((2,2-Dimethylbenzo[d][1,3]dioxol-5-yl)amino)-4-((thiazol-4-ylmethyl)amino)cyclobut-3-ene-1,2-dione (PRP022)

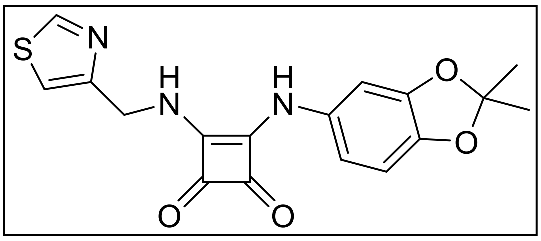

Intermediate 5 (0.26 g, 1.00 mmol) was dissolved in methanol (100 mL) followed by addition of thiazol-4-ylmethanamine dihydrochloride (0.19 g, 1.00 mmol) and *N*,*N*-diisopropylethylamine (0.26 g, 0.36 mL, 2.00 mmol). The reaction was carried out as described in General Procedure 3, which gave **PRP022** as a beige solid in 0.29 g (82 %, 0.82 mmol) yield.

^{1}H-NMR (600 MHz, DMSO-d_6) δ 10.08 – 9.98 (m, 1H), 9.11 (d, *J* = 1.9 Hz, 1H), 8.38 (s, 1H), 7.66 – 7.58 (m, 1H), 7.16 (s, 1H), 6.74 (d, *J* = 8.3 Hz, 1H), 6.70 (dd, *J* = 8.3, 2.3 Hz, 1H), 4.91 (d, *J* = 6.1 Hz, 2H), 1.60 (s, 6H);

^{13}C-NMR (151 MHz, DMSO-d_6) δ 183.54, 180.92, 169.10, 164.12, 155.43, 154.26, 147.84, 143.09, 133.77, 118.70, 116.80, 110.66, 108.63, 100.71, 43.79, 25.90;

MP: decomposition observed ≥ 237 °C;

HRMS (ESI, HRMS): *m/z* [M+H]$^+$ calcd for $C_{17}H_{15}N_3O_4S$: 358.0856, found: 358.0857;

HPLC purity: 97 %.

3-((2,3-Dihydro-1H-inden-5-yl)amino)-4-((thiazol-4-ylmethyl)amino)cyclobut-3-ene-1,2-dione (PRP023)

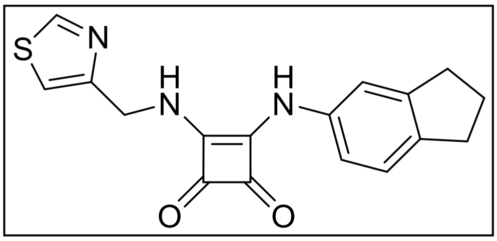

Intermediate 6 (0.24 g, 1.00 mmol) was dissolved in methanol (100 mL) followed by addition of thiazol-4-ylmethanamine dihydrochloride (0.19 g, 1.00 mmol) and *N*,*N*-diisopropylethylamine (0.26 g, 0.36 mL, 2.00 mmol). The reaction was carried out as described in General Procedure 3, which gave **PRP023** as a beige solid in 0.28 g (86 %, 0.86 mmol) yield.

^{1}H-NMR (600 MHz, DMSO-d_6) δ 9.81 (s, 1H), 9.11 (d, J = 2.0 Hz, 1H), 8.21 (s, 1H), 7.63 (d, J = 1.9 Hz, 1H), 7.31 (s, 1H), 7.13 (s, 2H), 4.92 (d, J = 6.0 Hz, 2H), 2.80 (t, J = 7.4 Hz, 2H), 2.76 (t, J = 7.4 Hz, 2H), 1.97 (p, J = 7.4 Hz, 2H).

^{13}C-NMR (151 MHz, dmso) δ 184.00, 180.96, 169.18, 164.37, 155.49, 154.22, 145.37, 138.53, 137.74, 125.13, 116.89, 116.70, 114.69, 43.76, 32.95, 32.08, 25.63;

MP: decomposition observed ≥ 242 °C;

HRMS (ESI, HRMS): *m/z* [M+H]$^+$ calcd for $C_{17}H_{15}N_3O_2S$: 326.0958, found: 326.0958;

HPLC purity: 95 %.

3-((5,6,7,8-Tetrahydronaphthalen-2-yl)amino)-4-((thiazol-4-ylmethyl)amino)cyclobut-3-ene-1,2-dione (PRP024)

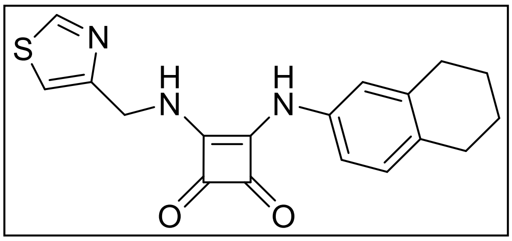

Intermediate 7 (0.26 g, 1.00 mmol) was dissolved in methanol (100 mL) followed by addition of thiazol-4-ylmethanamine dihydrochloride (0.19 g, 1.00 mmol) and *N*,*N*-diisopropylethylamine (0.26 g, 0.36 mL, 2.00 mmol). The reaction was carried out as described in General Procedure 3, which gave **PRP024** as a light brown solid in 0.30 g (89 %, 0.89 mmol) yield.

^{1}H-NMR (600 MHz, DMSO-d_6) δ 9.55 (s, 1H), 9.12 (d, *J* = 1.9 Hz, 1H), 7.98 (s, 1H), 7.63 (d, *J* = 1.9 Hz, 1H), 7.11 (dd, *J* = 8.1, 2.4 Hz, 1H), 7.07 – 7.02 (m, 1H), 6.97 (d, *J* = 8.3 Hz, 1H), 4.91 (s, 2H), 2.72 – 2.56 (m, 4H), 1.77 – 1.58 (m, 4H);

^{13}C-NMR (151 MHz, DMSO-d_6) δ 184.12, 181.06, 169.11, 164.34, 155.51, 154.18, 137.92, 136.73, 131.68, 130.08, 118.79, 116.93, 116.30, 43.72, 29.41, 28.59, 23.24, 23.06;

MP: decomposition observed ≥ 245 °C;

HRMS (ESI, HRMS): *m/z* [M+H]$^+$ calcd for $C_{18}H_{17}N_3O_2S$: 340.1114, found: 340.1112;

HPLC purity: 98 %.

3-((1,3-Dihydroisobenzofuran-5-yl)amino)-4-((thiazol-4-ylmethyl)amino)cyclobut-3-ene-1,2-dione (PRP025)

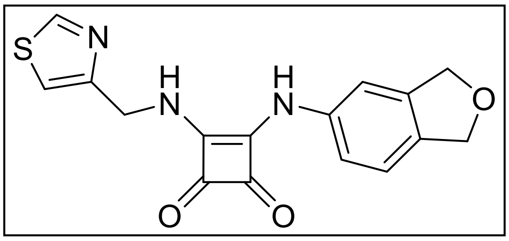

Intermediate 8 (0.24 g, 1.00 mmol) was dissolved in methanol (100 mL) followed by addition of thiazol-4-ylmethanamine dihydrochloride (0.19 g, 1.00 mmol) and *N*,*N*-diisopropylethylamine (0.26 g, 0.36 mL, 2.00 mmol). The reaction was carried out as described in General Procedure 3, which gave **PRP025** as a green solid in 0.21 g (64 %, 0.64 mmol) yield.

^{1}H-NMR (402 MHz, DMSO-d_6) δ 10.07 (s, 1H), 9.10 (d, *J* = 1.9 Hz, 1H), 8.37 (s, 1H), 7.62 (d, *J* = 1.9 Hz, 1H), 7.37 (s, 1H), 7.29 (dd, *J* = 8.1, 2.0 Hz, 1H), 7.21 (d, *J* = 8.1 Hz, 1H), 4.94 – 4.89 (m, 6H);

^{13}C-NMR (101 MHz, DMSO-d_6) δ 184.15, 180.88, 169.40, 164.22, 155.47, 154.17, 140.83, 138.91, 133.54, 122.23, 117.76, 116.87, 111.12, 72.95, 72.70, 43.79;

MP: decomposition observed ≥ 283 °C;

HRMS (ESI, HRMS): *m/z* $[M+Na]^+$ calcd for $C_{16}H_{13}N_3O_3S$: 350.0570, found: 350.0570;

HPLC purity: 98 %.

3-((4-Chloro-3-fluorophenyl)amino)-4-((thiazol-4-ylmethyl)amino)cyclobut-3-ene-1,2-dione (PRP026)

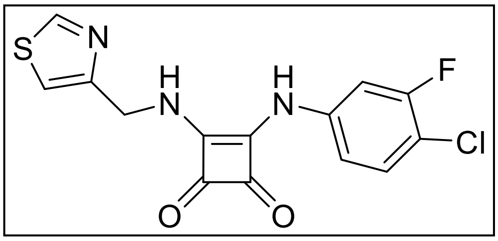

Intermediate 9 (0.26 g, 1.00 mmol) was dissolved in methanol (100 mL) followed by addition of thiazol-4-ylmethanamine dihydrochloride (0.19 g, 1.00 mmol) and *N*,*N*-diisopropylethylamine (0.26 g, 0.36 mL, 2.00 mmol). The reaction was carried out as described in General Procedure 3, which gave **PRP026** as a grey solid in 0.29 g (87 %, 0.87 mmol) yield.

^{1}H-NMR (402 MHz, DMSO-d_6) δ 10.61 – 10.32 (m, 1H), 9.09 (d, J = 1.9 Hz, 1H), 8.67 – 8.44 (m, 1H), 7.73 – 7.57 (m, 2H), 7.46 (t, J = 8.6 Hz, 1H), 7.15 (dd, J = 8.9, 2.5 Hz, 1H), 4.90 (d, J = 6.0 Hz, 2H);

^{13}C-NMR (101 MHz, DMSO-d_6) δ 184.48, 180.81, 169.83, 163.47, 157.88 (d, J = 244.5 Hz), 155.48, 153.99, 140.40 (d, J = 10.2 Hz), 131.48, 116.89, 115.31, 112.62 (d, J = 17.9 Hz), 106.81 (d, J = 25.8 Hz), 43.87;

^{19}F NMR (564 MHz, DMSO-d6) δ -114.02;

MP: decomposition observed ≥ 267 °C;

HRMS (ESI, HRMS): *m/z* $[M+Na]^+$ calcd for $C_{14}H_9ClFN_3O_2S$: 359.9980, found: 359.9979;

HPLC purity: 98 %.

3-((3-Bromo-4-chlorophenyl)amino)-4-((thiazol-4-ylmethyl)amino)cyclobut-3-ene-1,2-dione (PRP027)

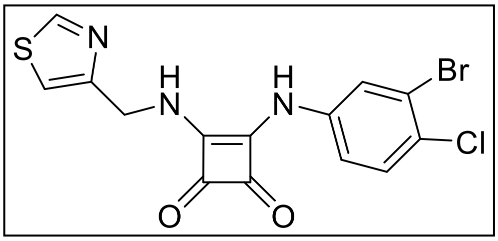

Intermediate 10 (0.32 g, 1.00 mmol) was dissolved in methanol (100 mL) followed by addition of thiazol-4-ylmethanamine dihydrochloride (0.19 g, 1.00 mmol) and *N*,*N*-diisopropylethylamine (0.26 g,

0.36 mL, 2.00 mmol). The reaction was carried out as described in General Procedure 3, which gave **PRP027** as a grey solid in 0.36 g (91 %, 0.91 mmol) yield.

^{1}H-NMR (^{1}H NMR (402 MHz, DMSO-d_6) δ 10.27 (s, 1H), 9.10 (d, *J* = 2.0 Hz, 1H), 8.43 (s, 1H), 7.93 (s, 1H), 7.62 (d, *J* = 1.9 Hz, 1H), 7.50 (d, *J* = 8.7 Hz, 1H), 7.34 (dd, *J* = 8.7, 2.7 Hz, 1H), 4.90 (d, *J* = 5.7 Hz, 2H);

^{13}C-NMR (101 MHz, DMSO-d_6) δ 184.57, 180.87, 169.76, 163.42, 155.51, 153.96, 139.71, 131.28, 126.48, 122.95, 122.28, 119.03, 116.94, 43.85;

MP: decomposition observed ≥ 283 °C;

HRMS (ESI, HRMS): *m/z* [M+H]$^+$ calcd for $C_{14}H_9BrClN_3O_2S$: 397.9360, found: 397.9365;

HPLC purity: 97 %.

3-(Benzo[*d*][1,3]dioxol-5-yl(methyl)amino)-4-((thiazol-4-ylmethyl)amino)cyclobut-3-ene-1,2-dione (PRP028)

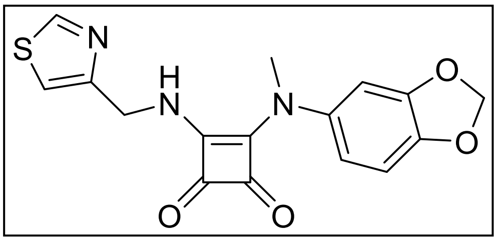

Intermediate 15 (0.26 g, 1.00 mmol) was dissolved in methanol (100 mL) followed by addition of thiazol-4-ylmethanamine dihydrochloride (0.19 g, 1.00 mmol) and *N*,*N*-diisopropylethylamine (0.26 g, 0.36 mL, 2.00 mmol). The reaction was carried out as described in General Procedure 3, which gave **PRP028** as a pale red solid in 0.30 g (80 %, 0.80 mmol) yield.

^{1}H-NMR (600 MHz, DMSO-d_6) δ 9.04 (d, *J* = 2.0 Hz, 1H), 7.46 (dd, *J* = 2.0, 1.0 Hz, 1H), 7.09 (s, 1H), 6.90 (d, *J* = 2.2 Hz, 1H), 6.87 (d, *J* = 8.3 Hz, 1H), 6.64 (dd, *J* = 8.3, 2.3 Hz, 1H), 6.01 (s, 2H), 4.80 (s, 2H), 3.58 (s, 3H);

^{13}C-NMR (151 MHz, DMSO-d_6) δ 184.62, 183.29, 168.22, 165.85, 155.42, 154.83, 148.25, 145.90, 136.60, 116.64, 115.83, 108.53, 104.98, 101.99, 44.03, 39.51;

MP: decomposition observed ≥ 218 °C;

HRMS (ESI, HRMS): *m/z* [M+H]$^+$ calcd for $C_{16}H_{13}N_3O_4S$: 344.0700, found: 344.0699;

HPLC purity: 97 %.

3-((2,2-Dimethylbenzo[*d*][1,3]dioxol-5-yl)(methyl)amino)-4-((thiazol-4-ylmethyl)amino)cyclobut-3-ene-1,2-dione (PRP029)

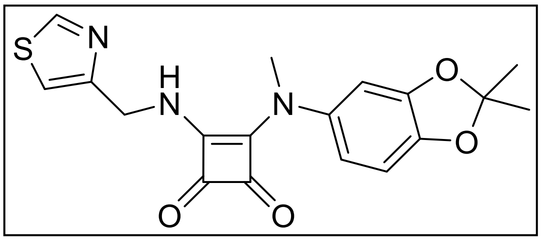

Intermediate 16 (0.29 g, 1.00 mmol) was dissolved in methanol (100 mL) followed by addition of thiazol-4-ylmethanamine dihydrochloride (0.19 g, 1.00 mmol) and *N*,*N*-diisopropylethylamine (0.26 g, 0.36 mL, 2.00 mmol). The reaction was carried out as described in General Procedure 3, which gave **PRP029** as a beige solid in 0.23 g (61 %, 0.61 mmol) yield.

^{1}H-NMR (600 MHz, DMSO-d_6) δ 9.03 (d, *J* = 1.9 Hz, 1H), 7.49 – 7.41 (m, 1H), 7.01 (s, 1H), 6.80 (d, *J* = 2.3 Hz, 1H), 6.77 (d, *J* = 8.2 Hz, 1H), 6.60 (dd, *J* = 8.2, 2.3 Hz, 1H), 4.80 (s, 2H), 3.57 (s, 3H), 1.62 (s, 6H);

^{13}C-NMR (151 MHz, DMSO-d_6) δ 184.52, 183.33, 168.14, 165.87, 155.37, 154.84, 147.79, 145.63, 136.15, 119.26, 116.22, 115.79, 108.28, 104.77, 44.05, 39.53, 26.01;

MP: 190 - 195 °C;

HRMS (ESI, HRMS): *m/z* [M+H]$^+$ calcd for $C_{18}H_{17}N_3O_4S$: 372.1013, found: 372.1011;

HPLC purity: 98 %.

3-((3,4-Dichlorophenyl)(methyl)amino)-4-((thiazol-4-ylmethyl)amino)cyclobut-3-ene-1,2-dione (PRP030)

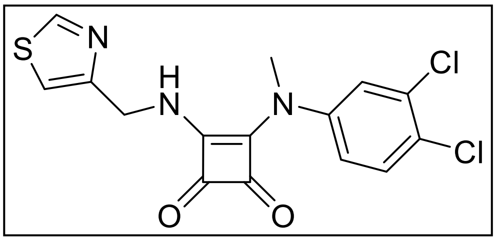

Intermediate 11 (0.29 g, 1.00 mmol) was dissolved in methanol (100 mL) followed by addition of thiazol-4-ylmethanamine dihydrochloride (0.19 g, 1.00 mmol) and *N*,*N*-diisopropylethylamine (0.26 g, 0.36 mL, 2.00 mmol). The reaction was carried out as described in General Procedure 3, which gave **PRP030** as a yellow solid in 0.31 g (85 %, 0.85 mmol) yield.

^{1}H-NMR (600 MHz, DMSO-d_6) δ 9.06 (d, *J* = 1.9 Hz, 1H), 7.76 – 7.67 (m, 1H), 7.57 (d, *J* = 8.8 Hz, 1H), 7.54 (d, *J* = 1.8 Hz, 1H), 7.37 (d, *J* = 2.6 Hz, 1H), 7.10 (dd, *J* = 8.7, 2.7 Hz, 1H), 4.87 (d, *J* = 5.8 Hz, 2H), 3.60 (s, 3H);

^{13}C-NMR (151 MHz, DMSO-*d*$_6$) δ 186.31, 183.36, 169.14, 164.85, 155.05, 154.97, 141.82, 132.00, 131.15, 127.22, 123.67, 121.88, 116.23, 44.02, 38.38;

MP: decomposition observed ≥ 238 °C;

HRMS (ESI, HRMS): *m/z* [M+H]$^+$ calcd for $C_{15}H_{11}Cl_2N_3O_2S$: 368.0022, found: 368.0026;

HPLC purity: 98 %.

3-((3,4-Difluorophenyl)(methyl)amino)-4-((thiazol-4-ylmethyl)amino)cyclobut-3-ene-1,2-dione (PRP031)

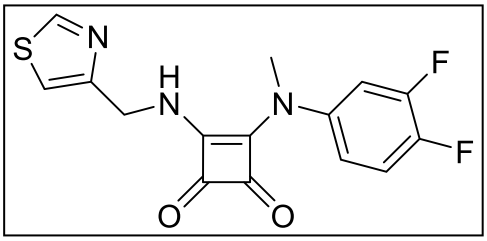

Intermediate 12 (0.25 g, 1.00 mmol) was dissolved in methanol (100 mL) followed by addition of thiazol-4-ylmethanamine dihydrochloride (0.19 g, 1.00 mmol) and *N*,*N*-diisopropylethylamine (0.26 g, 0.36 mL, 2.00 mmol). The reaction was carried out as described in General Procedure 3, which gave **PRP031** as a white solid in 0.30 g (88 %, 0.88 mmol) yield.

^{1}H-NMR (600 MHz, DMSO-*d*$_6$) δ 9.05 (d, *J* = 1.9 Hz, 1H), 7.51 (d, *J* = 2.0 Hz, 2H), 7.41 (dt, *J* = 10.5, 9.0 Hz, 1H), 7.30 (ddd, *J* = 12.1, 7.0, 2.8 Hz, 1H), 7.03 – 6.98 (m, 1H), 4.85 (s, 2H), 3.60 (s, 3H);

^{13}C-NMR (151 MHz, DMSO-*d*$_6$) δ 185.77, 183.26, 168.80, 165.30, 155.19, 154.91, 149.85 (dd, *J* = 245.9, 13.6 Hz), 147.58 (dd, *J* = 243.2, 12.7 Hz), 138.96 (d, *J* = 8.2 Hz), 119.03 (dd, *J* = 6.5, 3.2 Hz), 118.07 (d, *J* = 18.2 Hz), 116.09, 112.17 (d, *J* = 19.8 Hz), 44.01, 38.83;

^{19}F NMR (564 MHz, DMSO-*d*$_6$) δ -136.63 (dt, *J* = 22.3, 10.6 Hz), -142.92 (dddd, *J* = 22.0, 10.6, 7.1, 3.8 Hz);

MP: 214 - 218 °C;

HRMS (ESI, HRMS): *m/z* [M+H]$^+$ calcd for $C_{15}H_{11}F_2N_3O_2S$: 336.0613, found: 336.0611;

HPLC purity: 98 %.

2. Activity of SQA derivatives against clinical isolates

Table S1. Antimycobacterial activity of SQA derivatives against *Mav* SE01 and *Mtb* Lin4Haarlem

Compound	MIC$_{95}$(*Mav SE01*) [µM]	MIC$_{95}$(*Mtb Lin4Haarlem*) [µM]
**SQ31f**	4	n.d.
**PRP003**	4	n.d.
**SQ6ab**	n.d.	16
**PRP019**	n.d.	16
**PRP020**	64	4
**PRP021**	n.d.	16
**PRP022**	32	n.d.
**PRP023**	>64	n.d.
**PRP024**	>64	n.d.
**PRP025**	>64	n.d.
**PRP026**	>64	n.d.

3. Additional cytotoxicity data of SQA derivatives

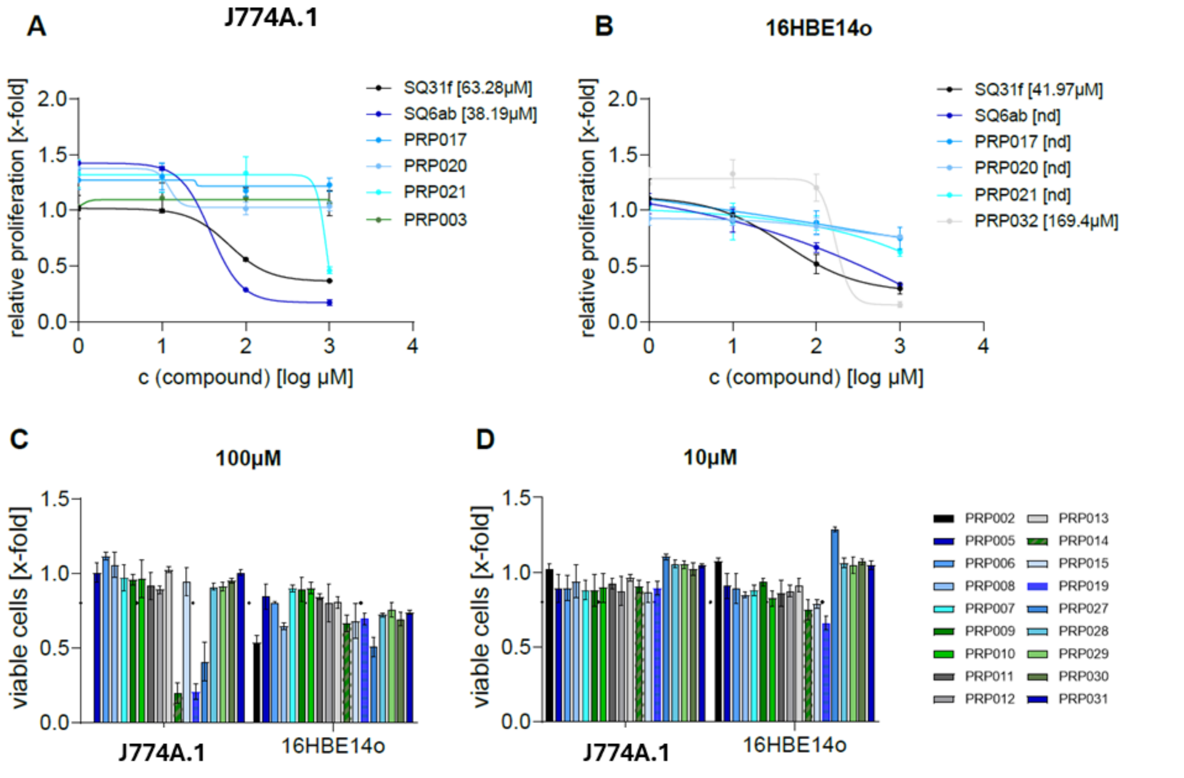

Figure S1. **A/B** Dose dependent antiproliferative effect of SQAs against murine monocytes (J774A.1 cells) and human bronchial epithelial cells (16HBE14σ) **C/D** Acute Toxicity against murine monocytes (J774A.1 cells) and human bronchial epithelial cells (16HBE14σ) at 10/100 µM.

4. Reaction schemes for Synthetic procedures

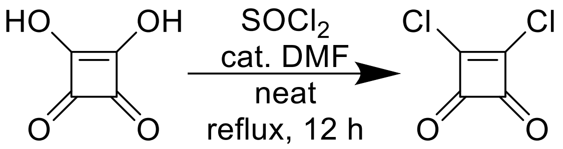

Scheme S1. Conversion of squaric acid to corresponding dichloride

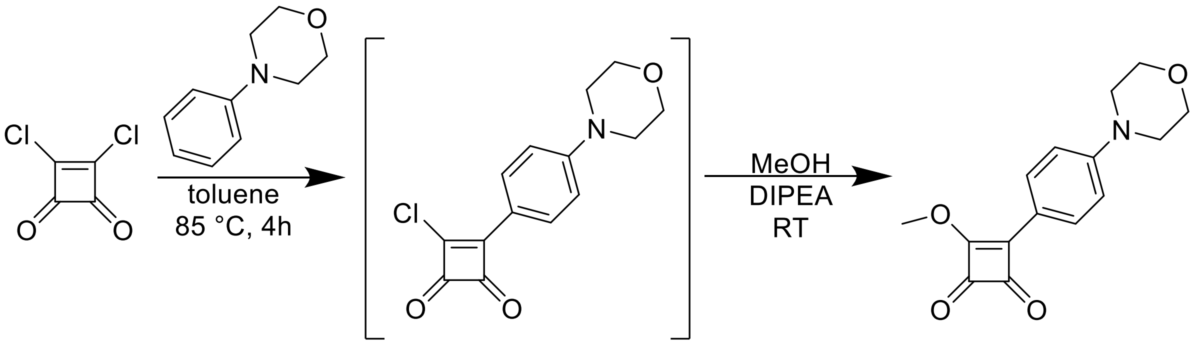

Scheme S2. Monoarylation of squaric acid dichloride

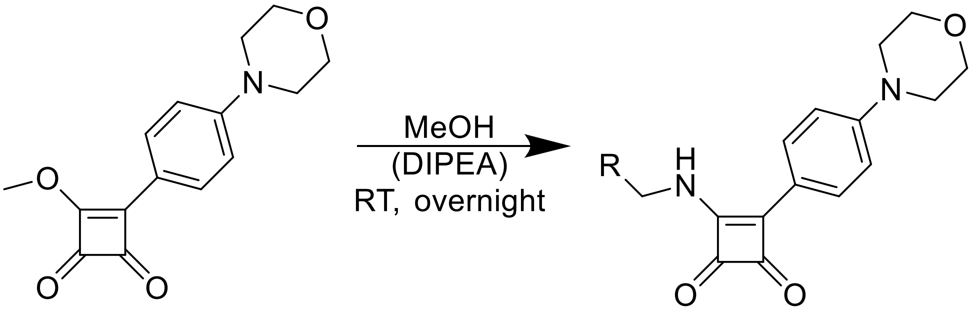

Scheme S3. General Procedure 1 Synthesis of SQA monoamino substituted squaramides:

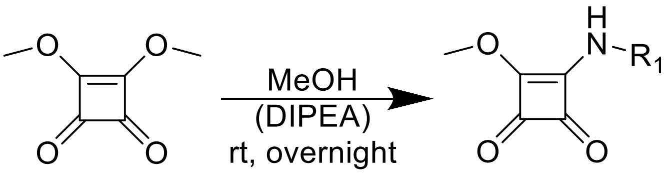

Scheme S4. General Procedure 2 Synthesis of monoamino substituted squaric acid methylesters

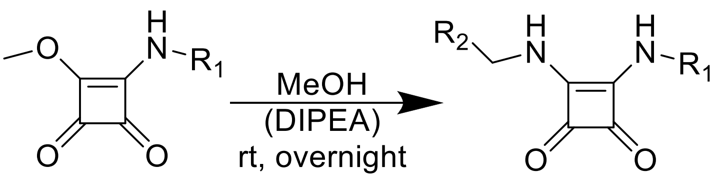

Scheme S5. General Procedure 3 Synthesis of diamino substituted squaramides

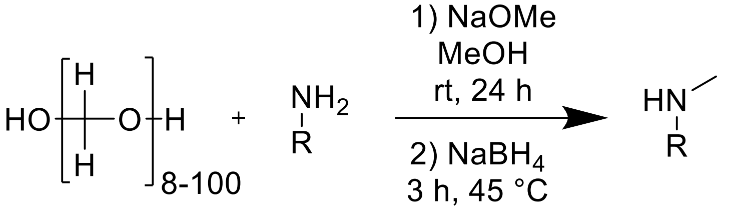

Scheme S6. General Procedure 4 *N*-Methylation of primary amines:[4]

5. References

(1) Li, P.; Wang, B.; Li, G.; Fu, L.; Zhang, D.; Lin, Z.; Huang, H.; Lu, Y. Design, synthesis and biological evaluation of diamino substituted cyclobut-3-ene-1,2-dione derivatives for the treatment of drug-resistant tuberculosis. *Eur J Med Chem* **2020**, *206*, 112538. DOI: 10.1016/j.ejmech.2020.112538.

(2) Chasak, J.; Oorts, L.; Dak, M.; Slachtova, V.; Bazgier, V.; Berka, K.; De Vooght, L.; Smiejkowska, N.; Calster, K. V.; Van Moll, L.; et al. Expanding the squaramide library as mycobacterial ATP synthase inhibitors: Innovative synthetic pathway and biological evaluation. *Bioorg Med Chem* **2023**, *95*, 117504. DOI: 10.1016/j.bmc.2023.117504.

(3) Palme, P. R.; Goddard, R.; Richter, A.; Imming, P.; Seidel, R. W. 3-[(Benzo-1,3-dioxol-5-yl)amino]-4-methoxy-cyclobut-3-ene-1,2-dione: polymorphism and twinning of a precursor to an antimycobacterial squaramide. *Acta Crystallogr C* **2024**, *80*, 375-+. DOI: 10.1107/S2053229624006211.

(4) Tabaru, K.; Fujihara, T.; Torii, K.; Suzuki, T.; Jing, Y.; Toyao, T.; Maeno, Z.; Shimizu, K. I.; Watanabe, T.; Sogawa, H.; et al. Exploring Catalytic Intermediates in Pd-Catalyzed Aerobic Oxidative Amination of 1,3-Dienes: Multiple Metal Interactions of the Palladium Nanoclusters. *J Am Chem Soc* **2024**, *146* (33), 22993-23003. DOI: 10.1021/jacs.4c02518.

(5) Tantry, S. J.; Markad, S. D.; Shinde, V.; Bhat, J.; Balakrishnan, G.; Gupta, A. K.; Ambady, A.; Raichurkar, A.; Kedari, C.; Sharma, S.; et al. Discovery of Imidazo[1,2-a]pyridine Ethers and Squaramides as Selective and Potent Inhibitors of Mycobacterial Adenosine Triphosphate (ATP) Synthesis. *J Med Chem* **2017**, *60* (4), 1379-1399. DOI: 10.1021/acs.jmedchem.6b01358.

6. NMR Spectra

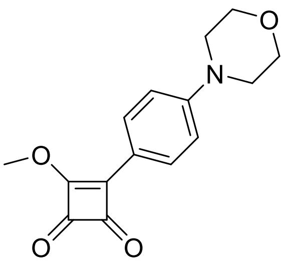

Intermediate 1

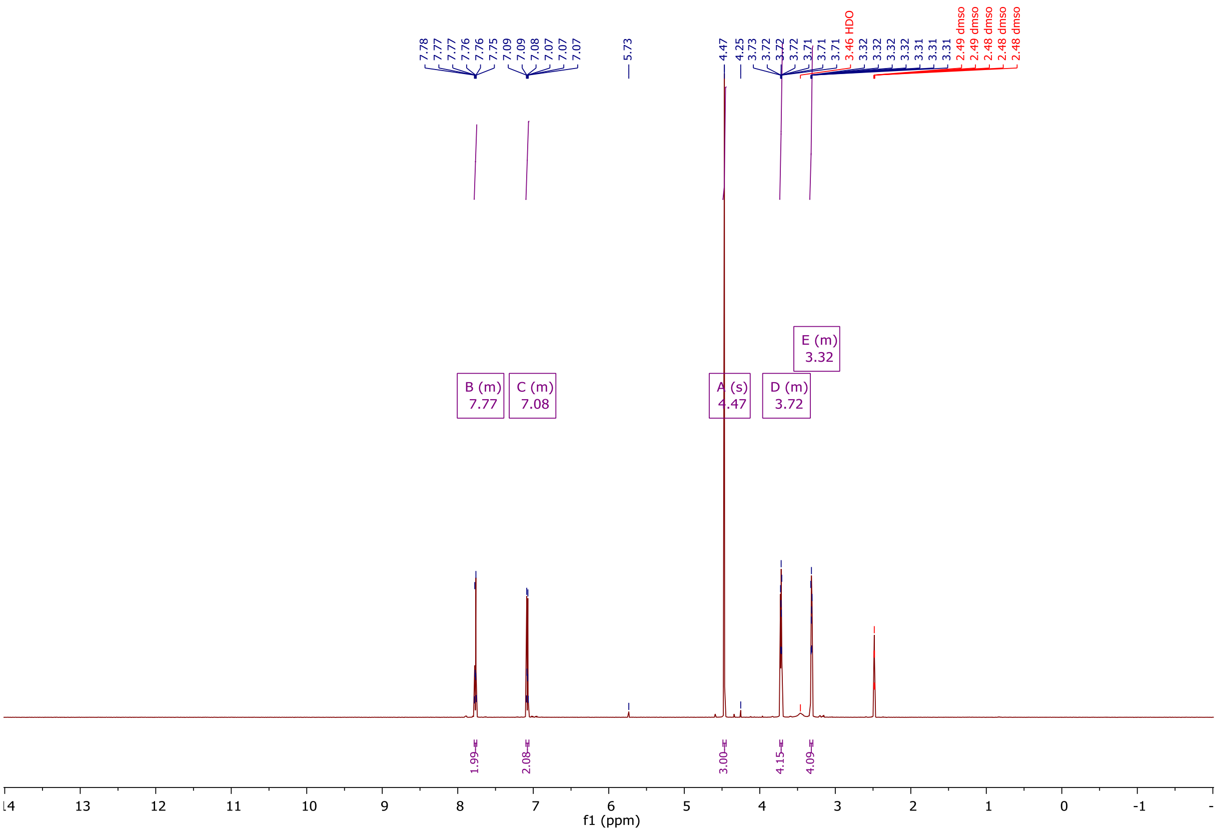

Bunge_797_PROTON_2025-03-14_01 — 2025-03-14 — Bunge_797 — VB041 —

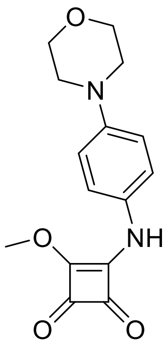

Intermediate 2

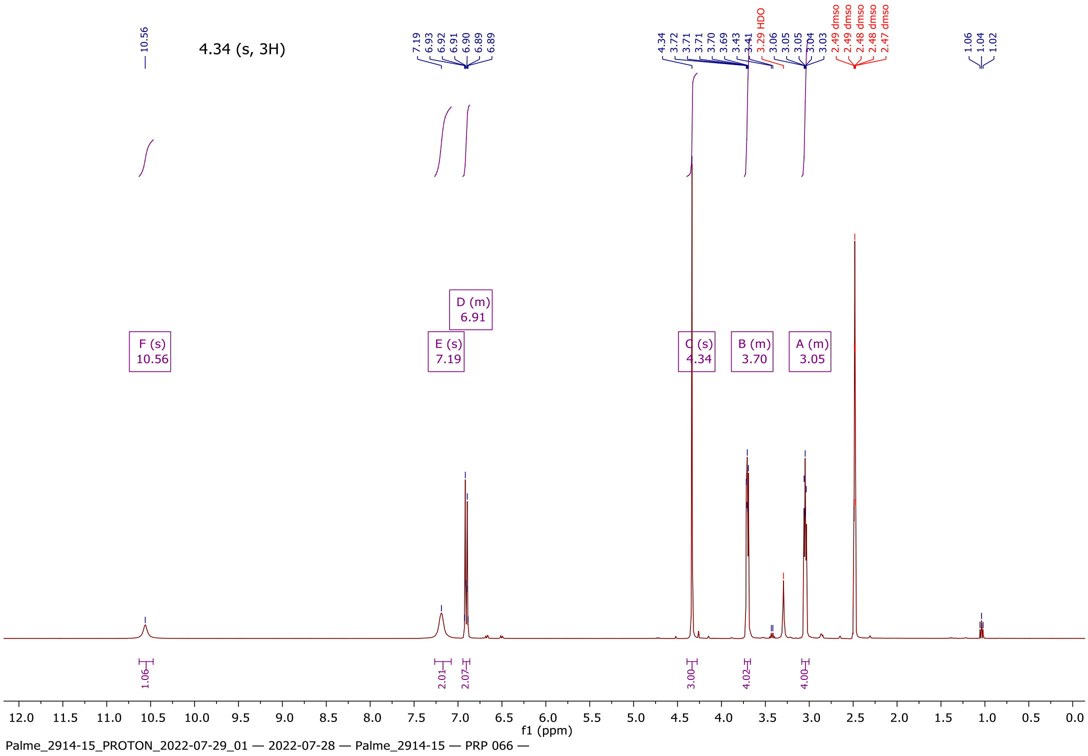

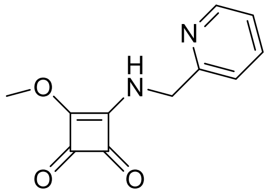

Intermediate 3

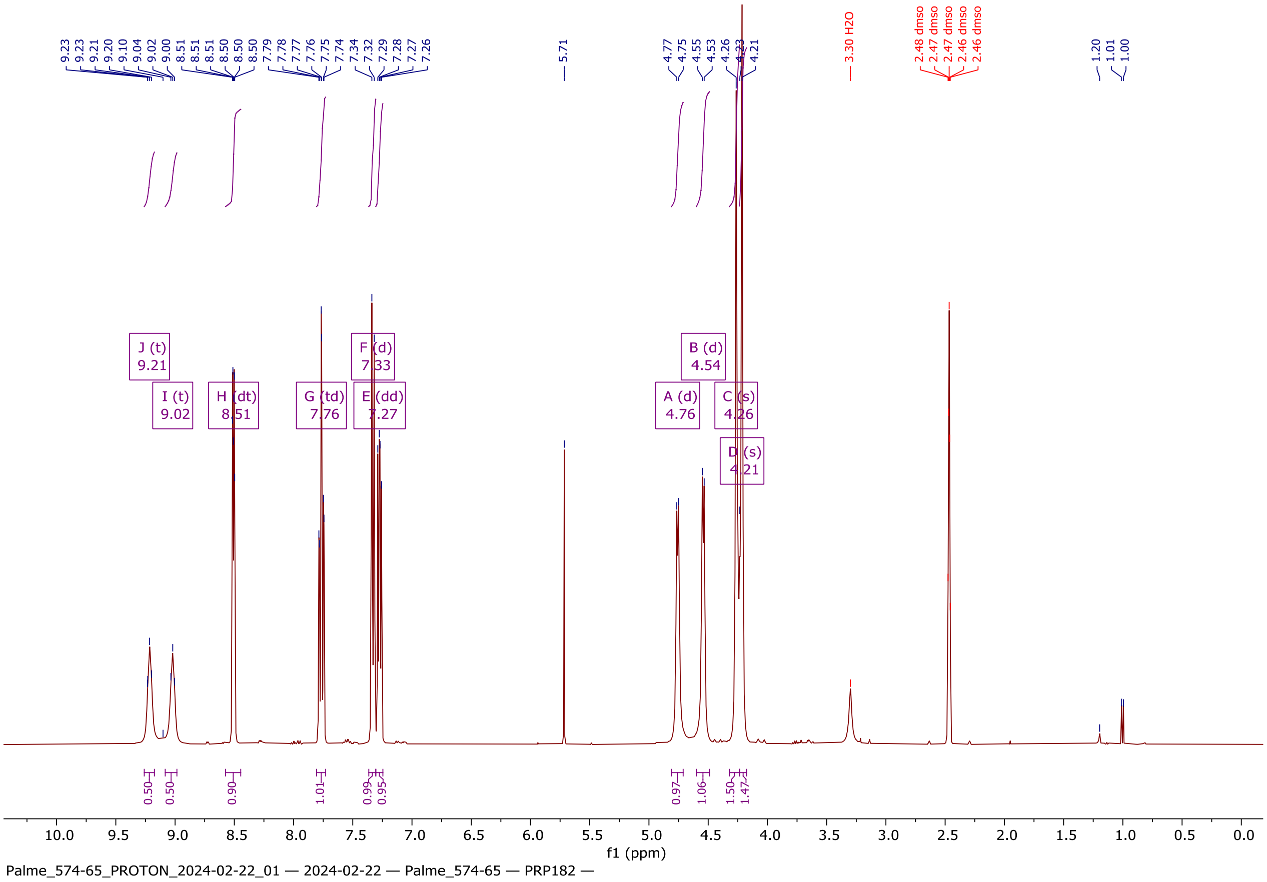

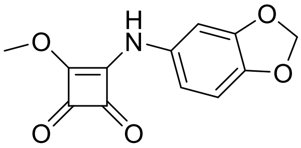

Intermediate 4

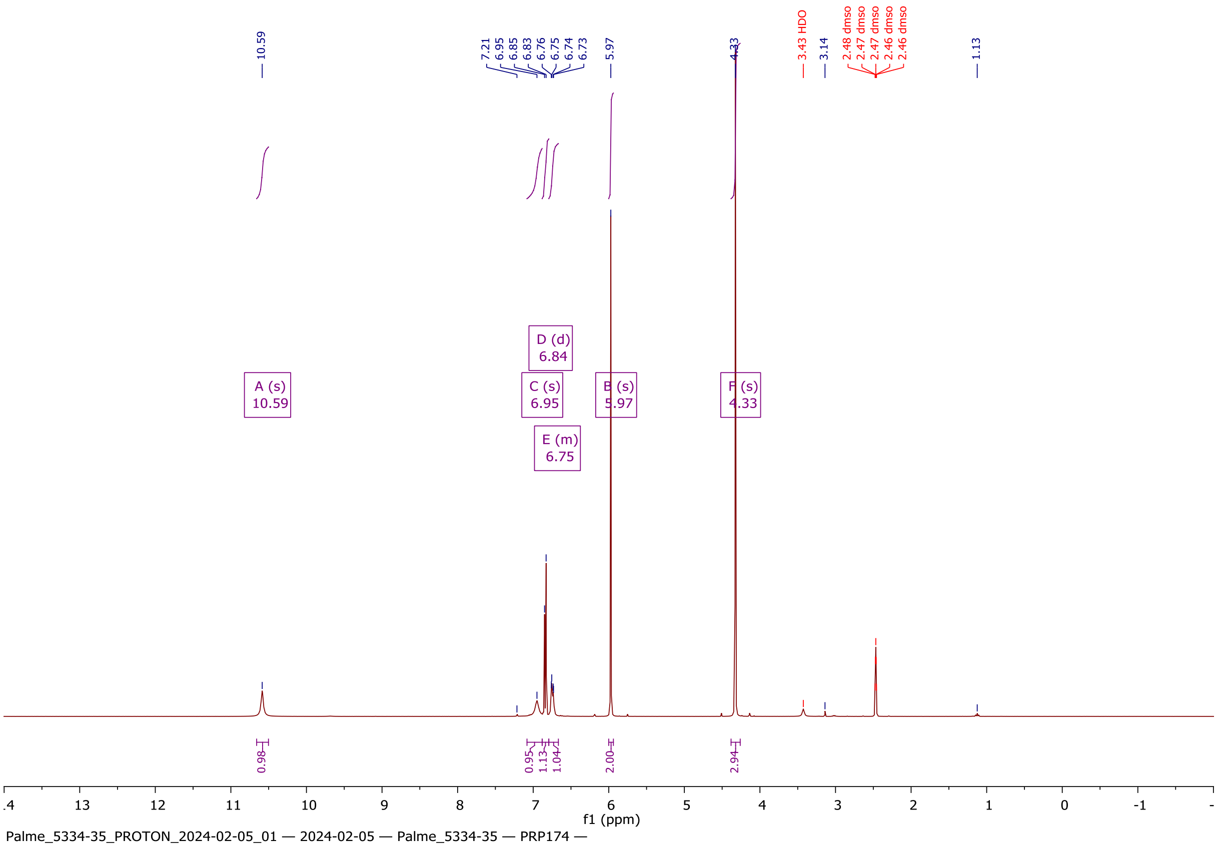

Palme_5334-35_PROTON_2024-02-05_01 — 2024-02-05 — Palme_5334-35 — PRP174 —

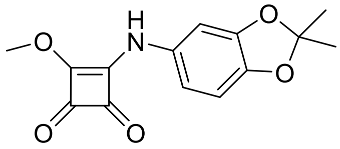

Intermediate 5

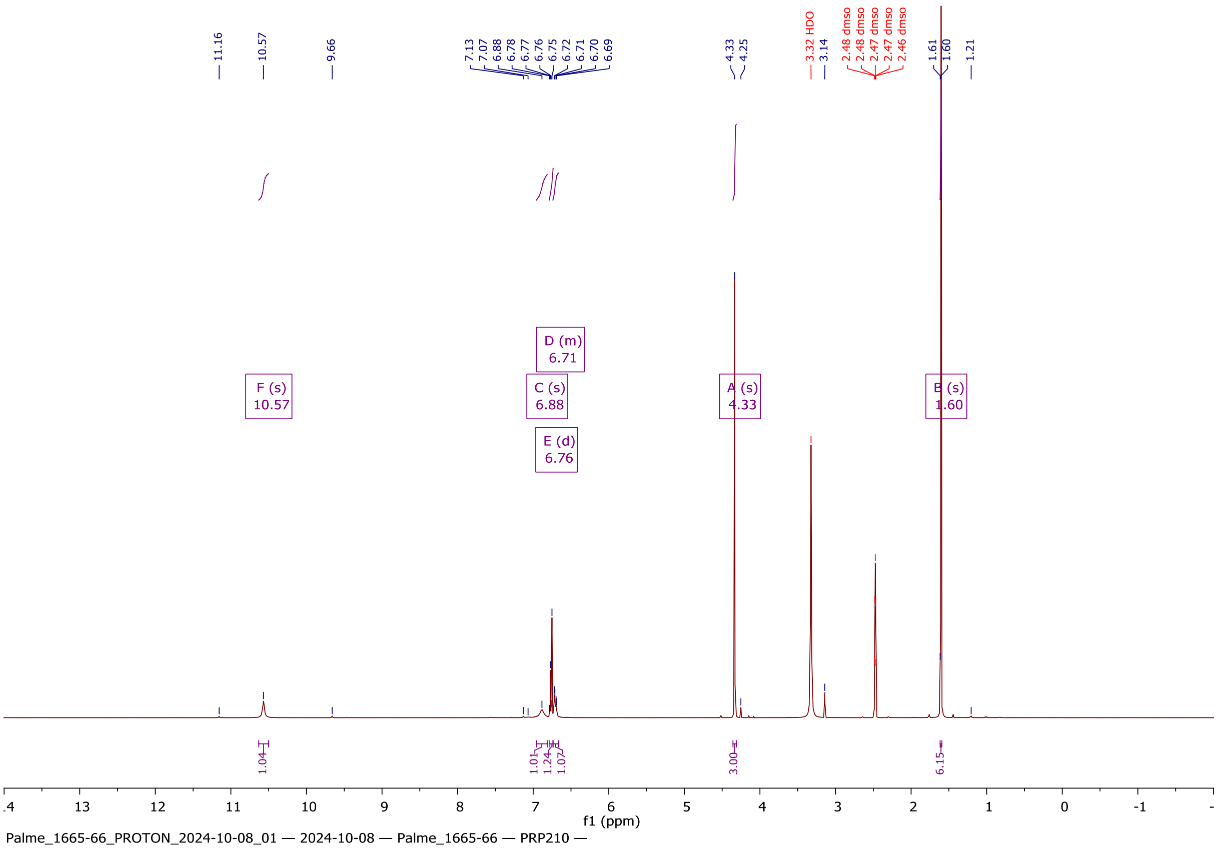

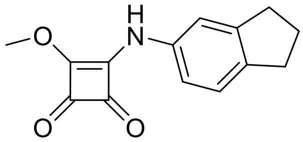

Intermediate 6

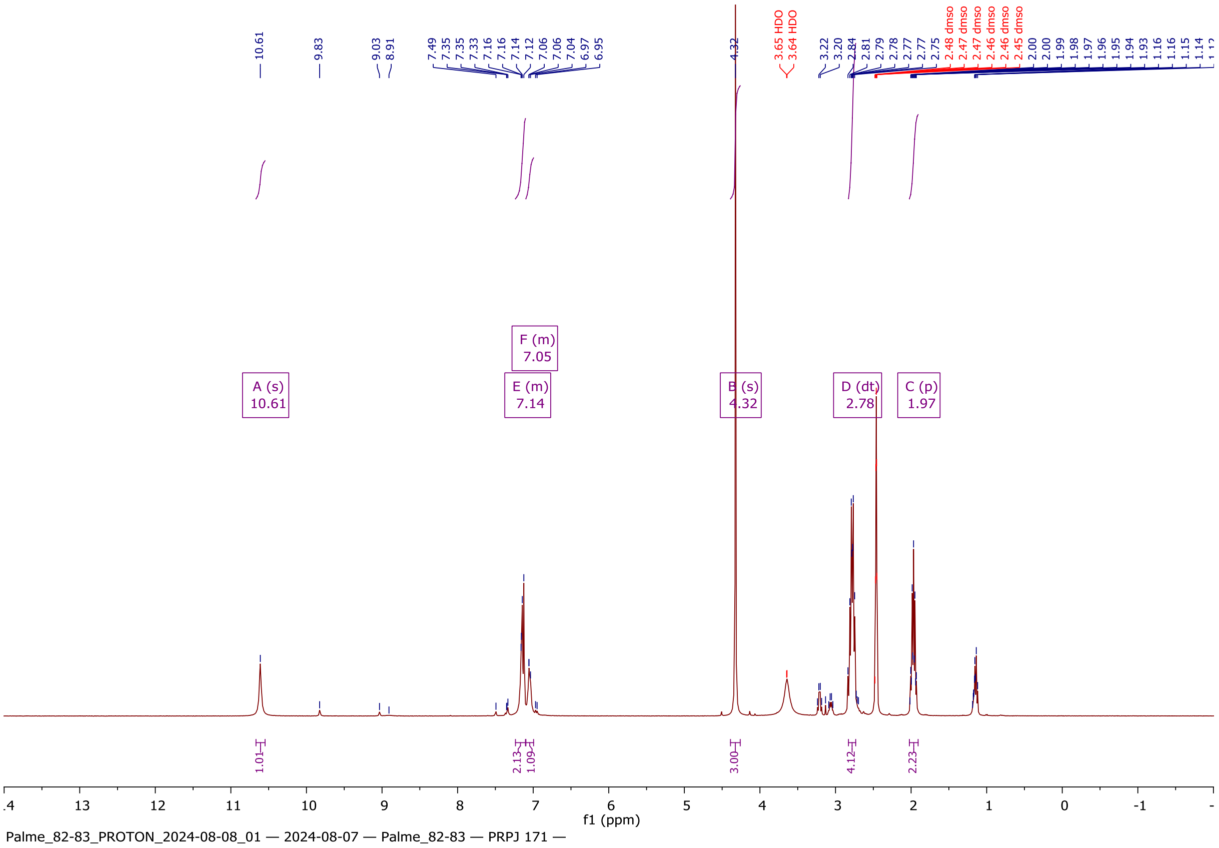

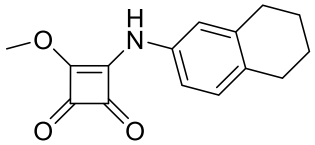

Intermediate 7

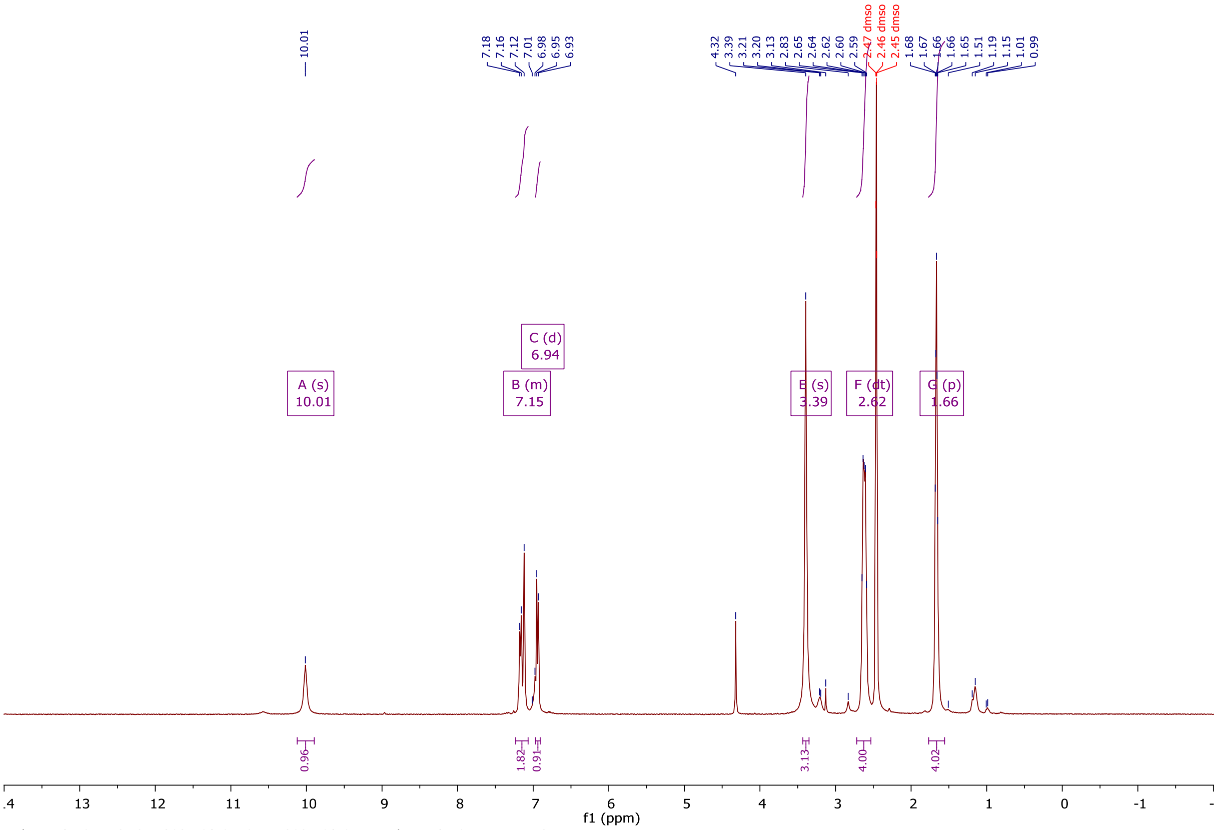

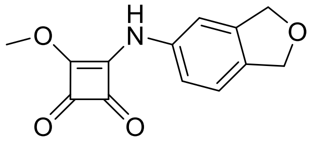

Intermediate 8

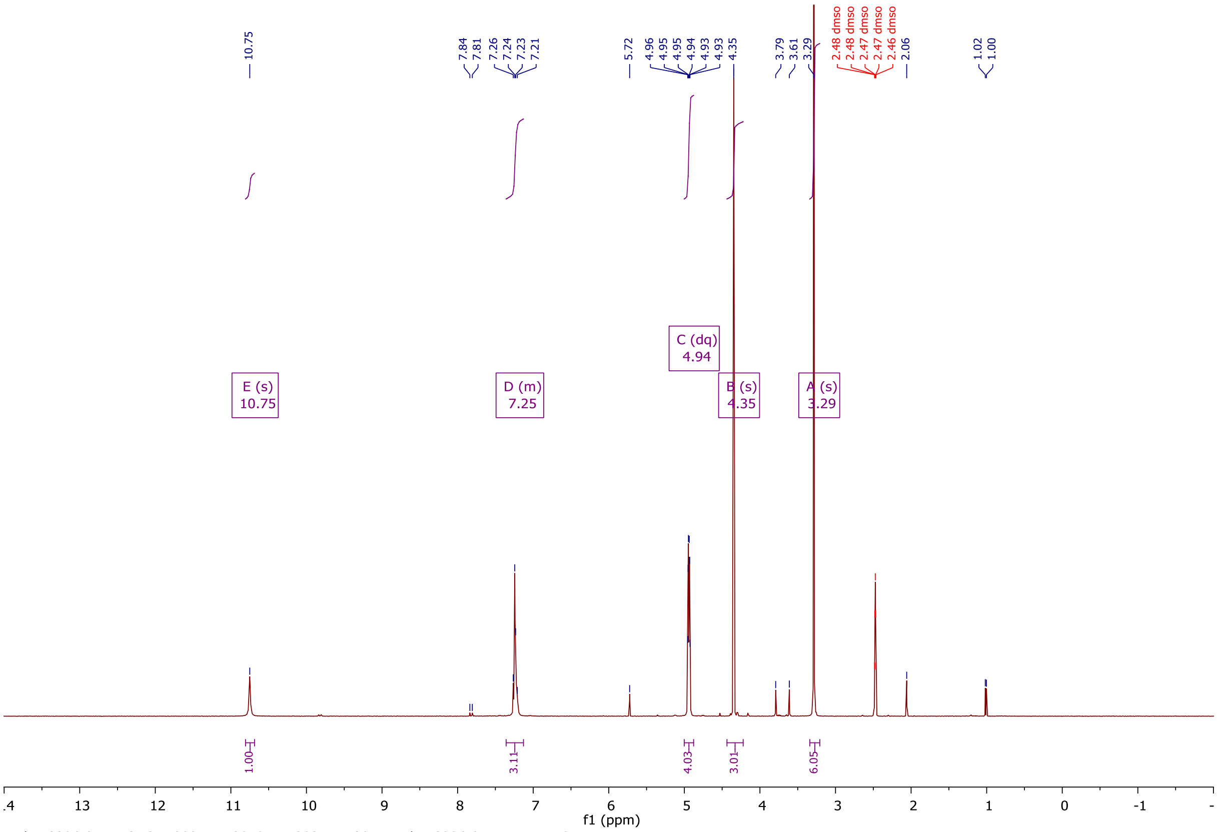

Dube_8206-07_PROTON_2024-11-20_01 — 2024-11-20 — Dube_8206-07 — PRPJ 170 —

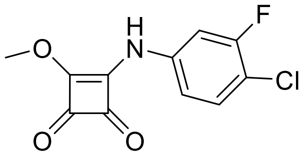

Intermediate 9

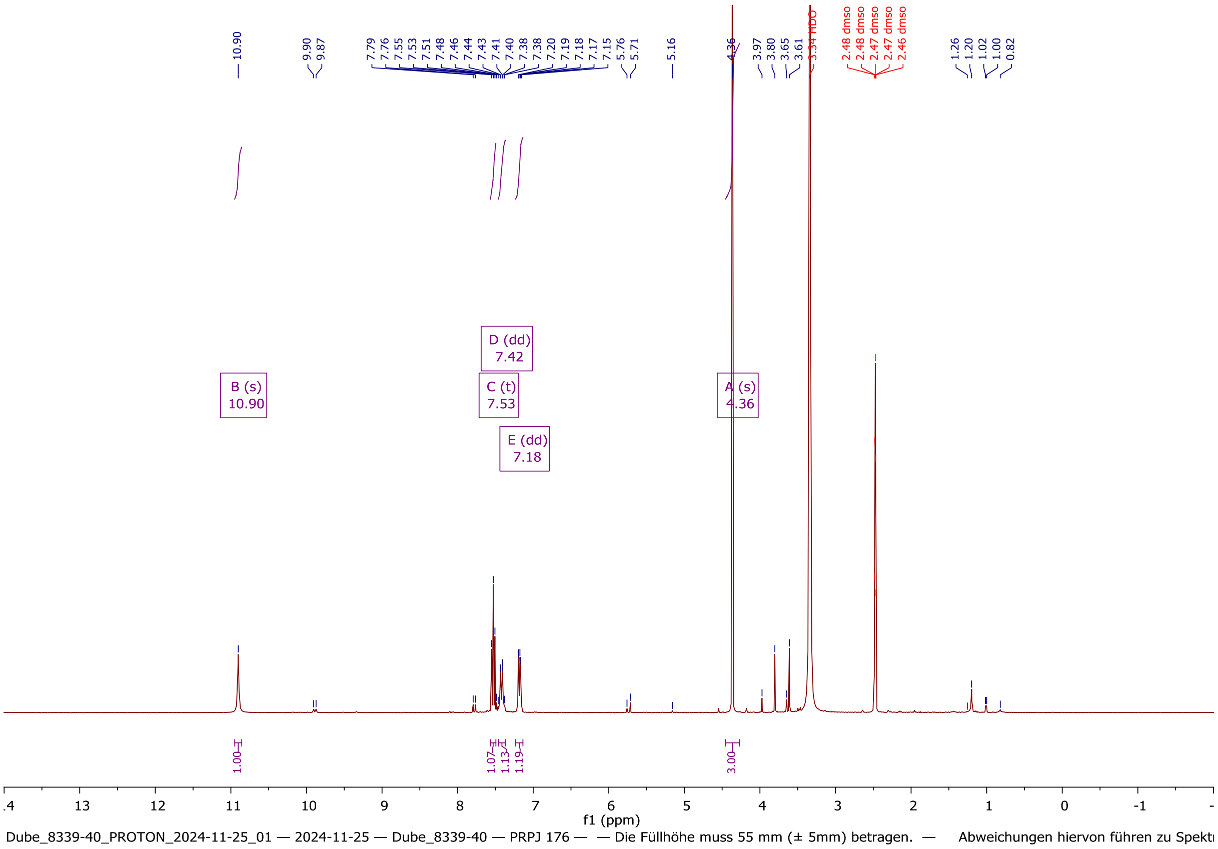

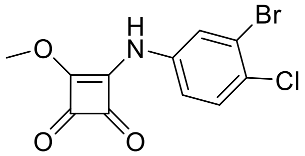

Intermediate 10

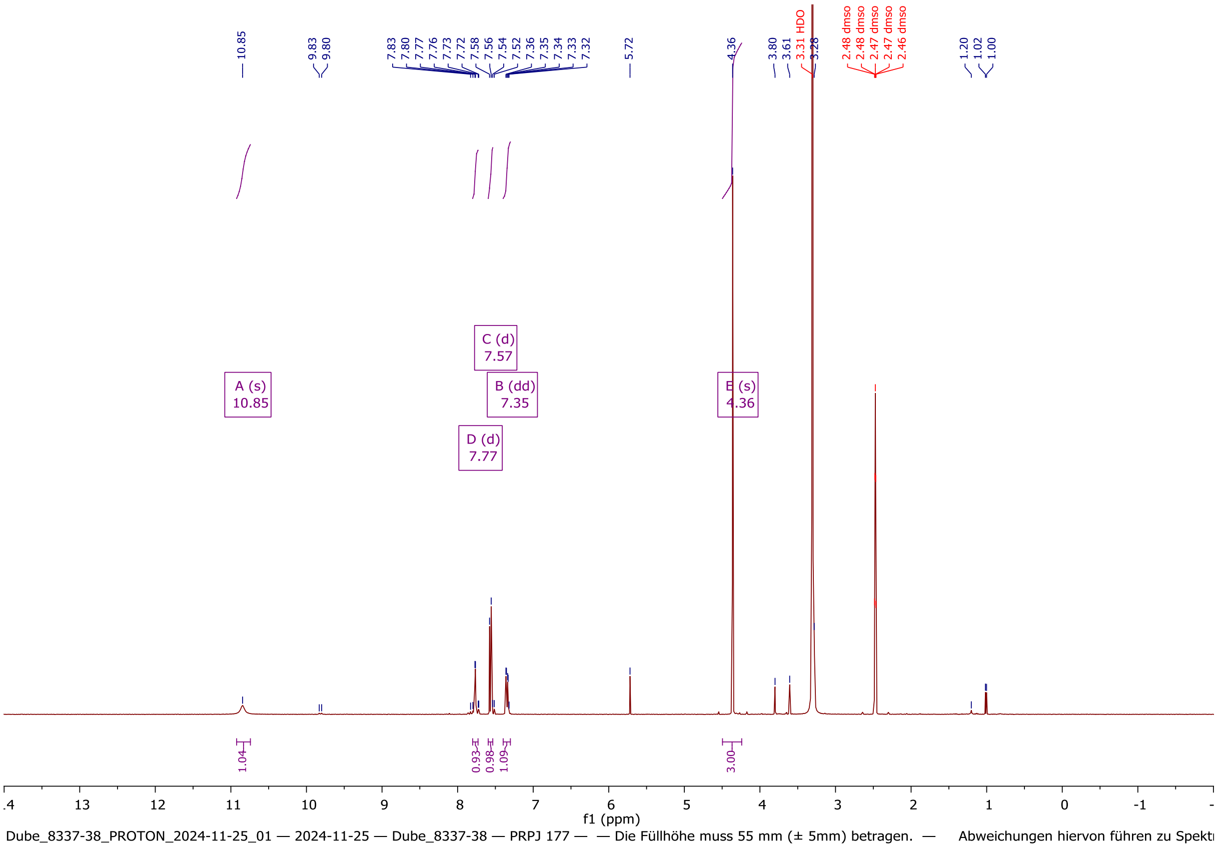

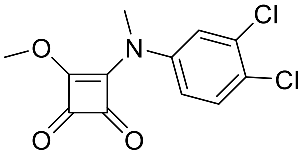

Intermediate 11

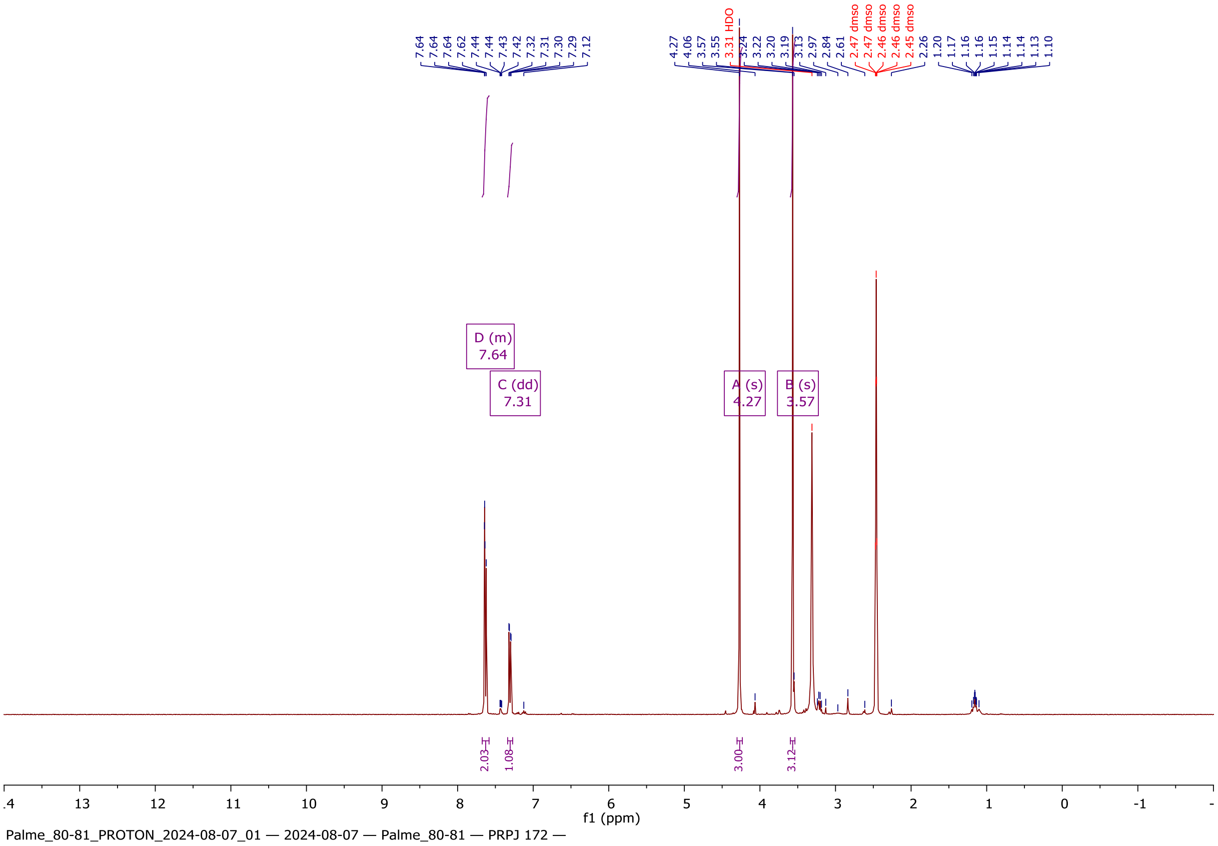

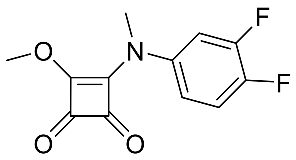

Intermediate 12

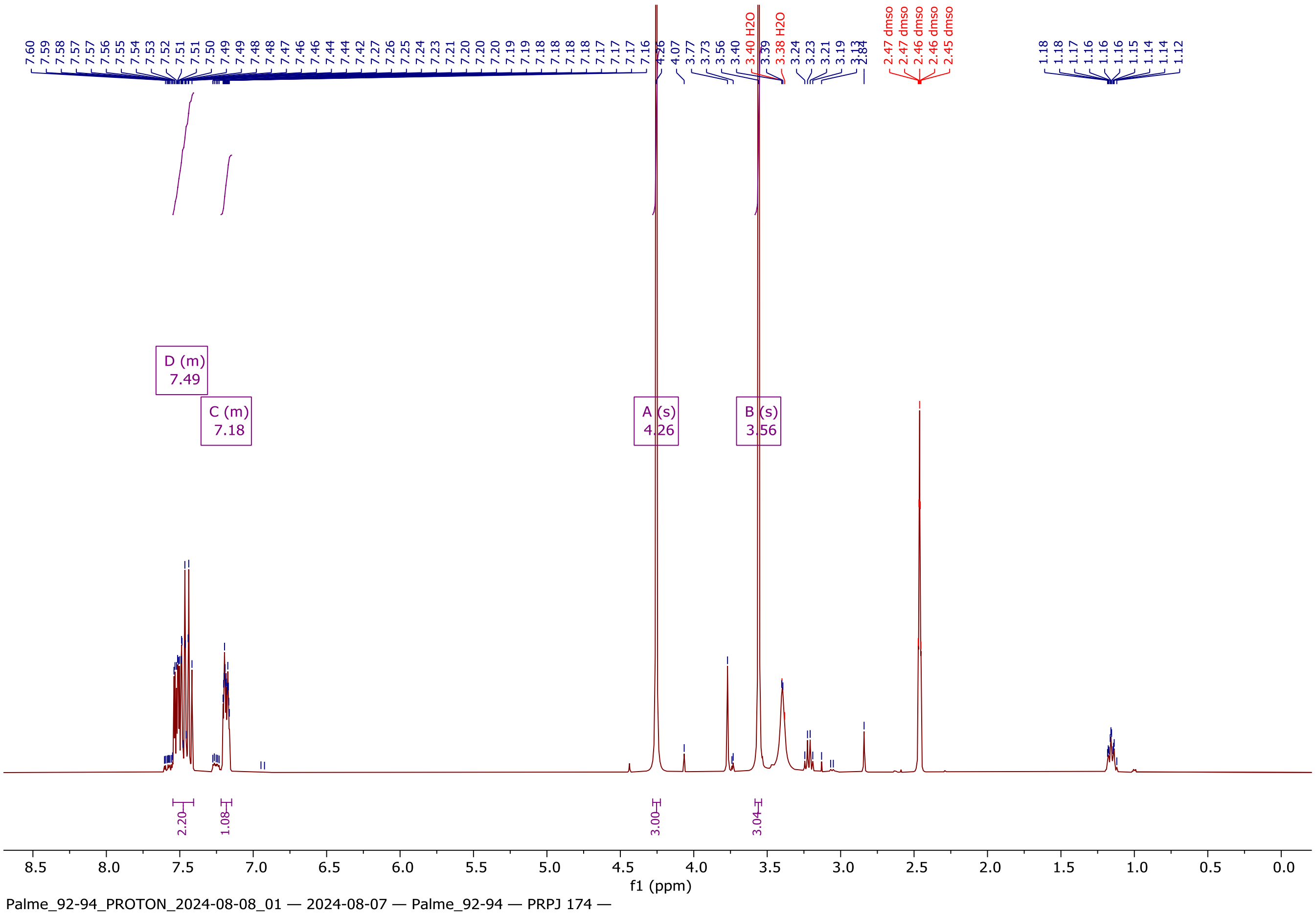

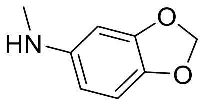

Intermediate 13

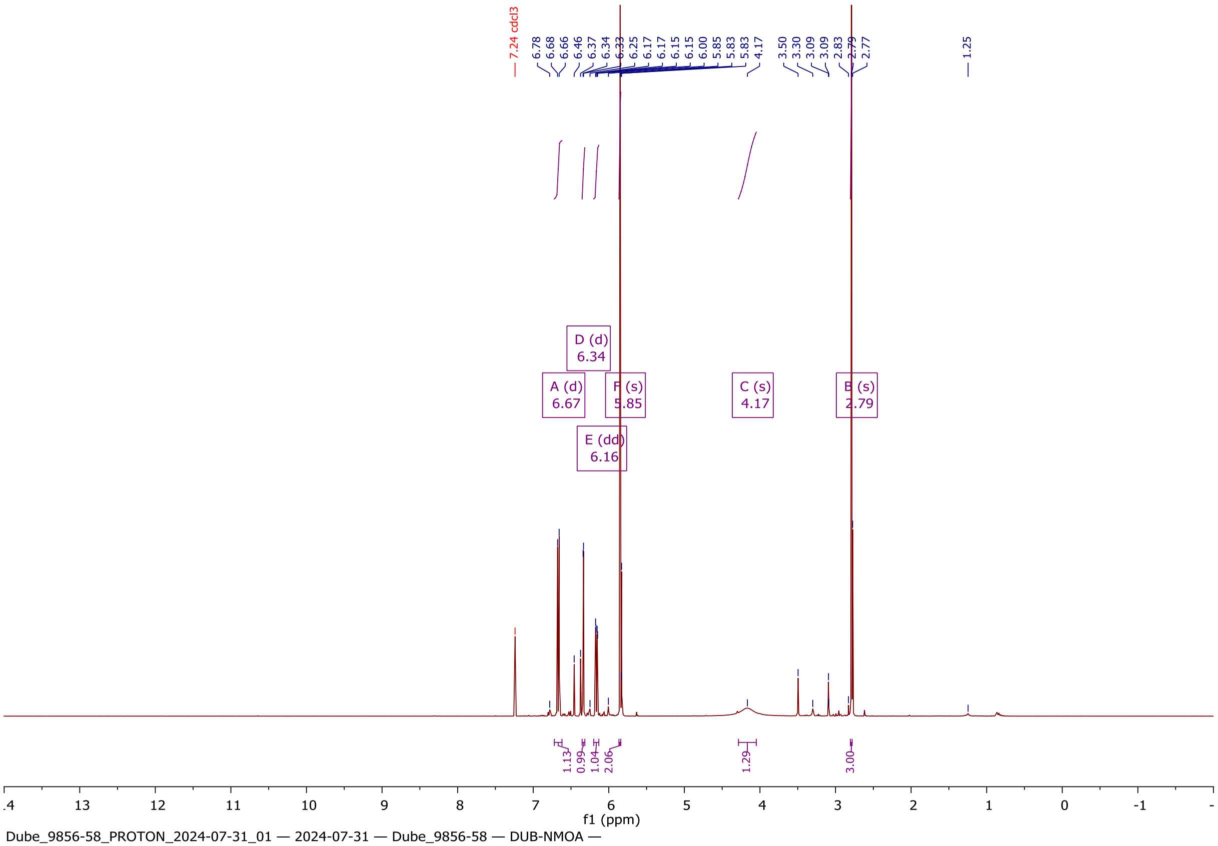

Dube_9856-58_PROTON_2024-07-31_01 — 2024-07-31 — Dube_9856-58 — DUB-NMOA —

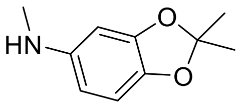

Intermediate 14

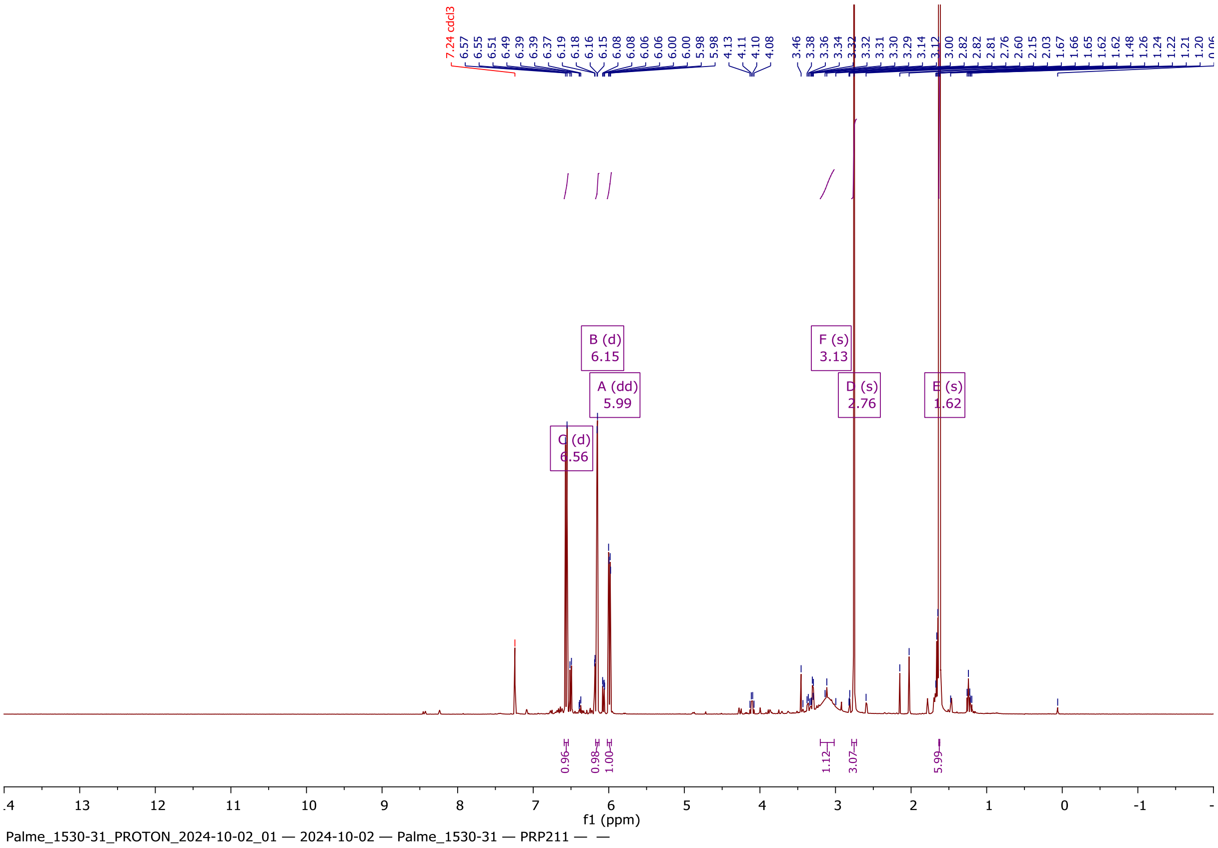

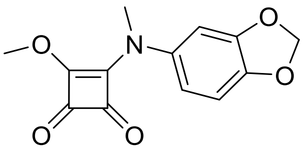

Intermediate 15

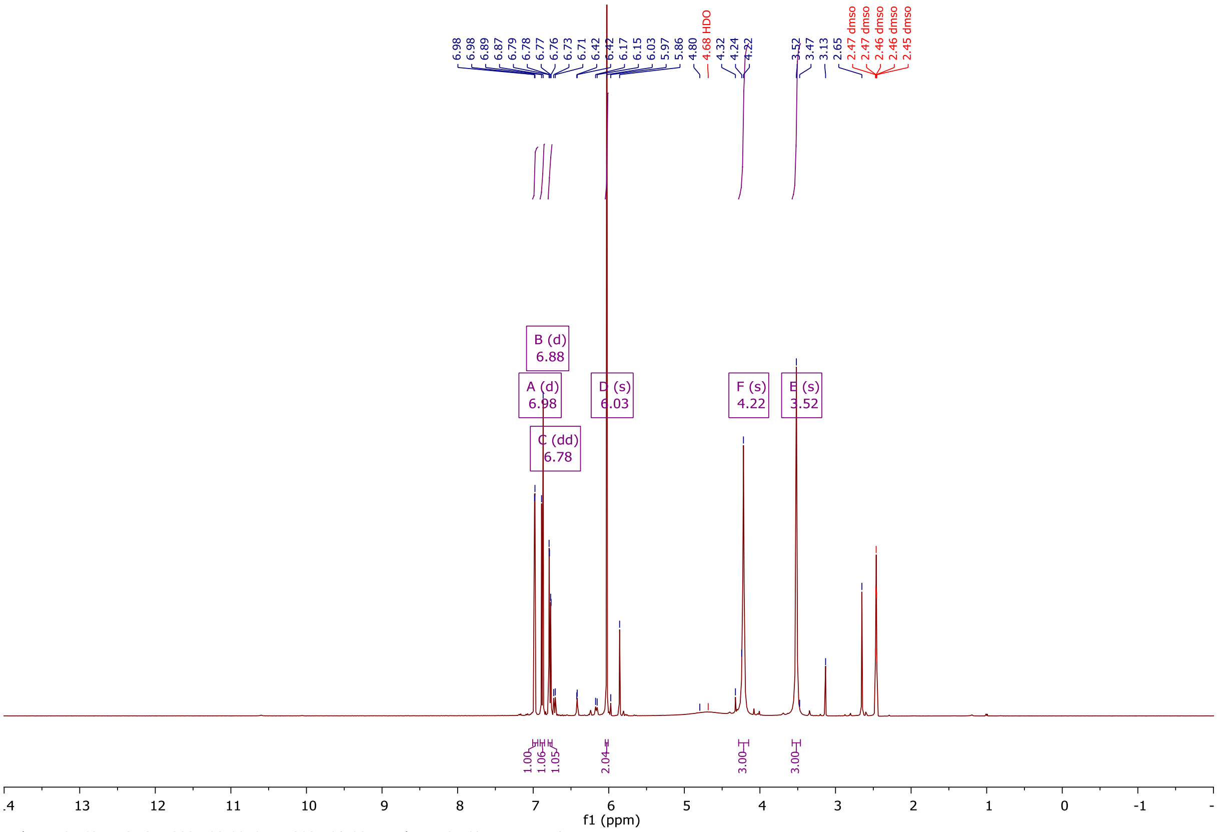

Palme_161-62_PROTON_2024-08-09_01 — 2024-08-09 — Palme_161-62 — PRPJ 178 —

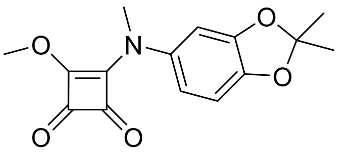

Intermediate 16

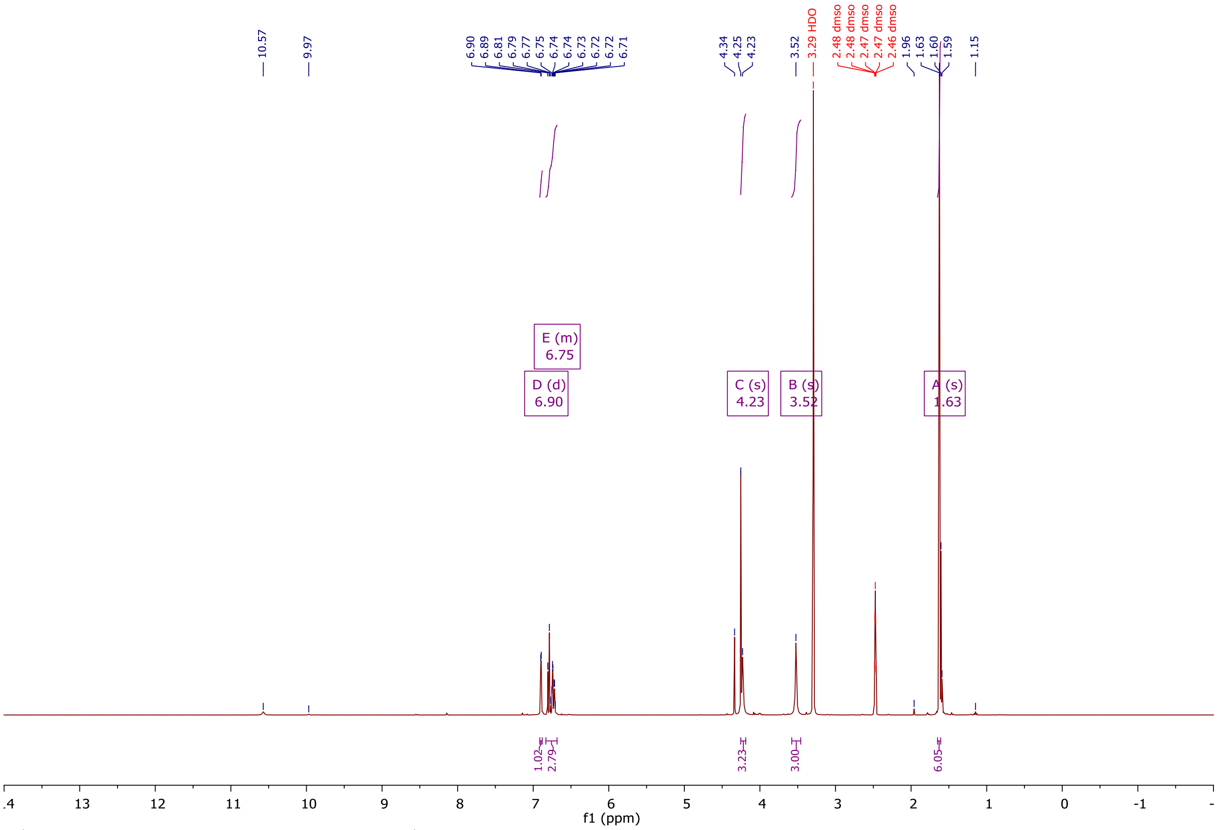

Palme_1694-95_PROTON_2024-10-09_01 — 2024-10-08 — Palme_1694-95 — PRP212 —

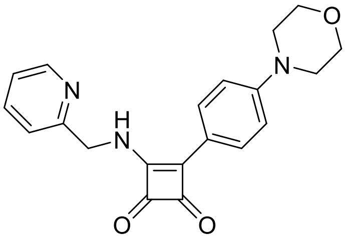

SQ31f

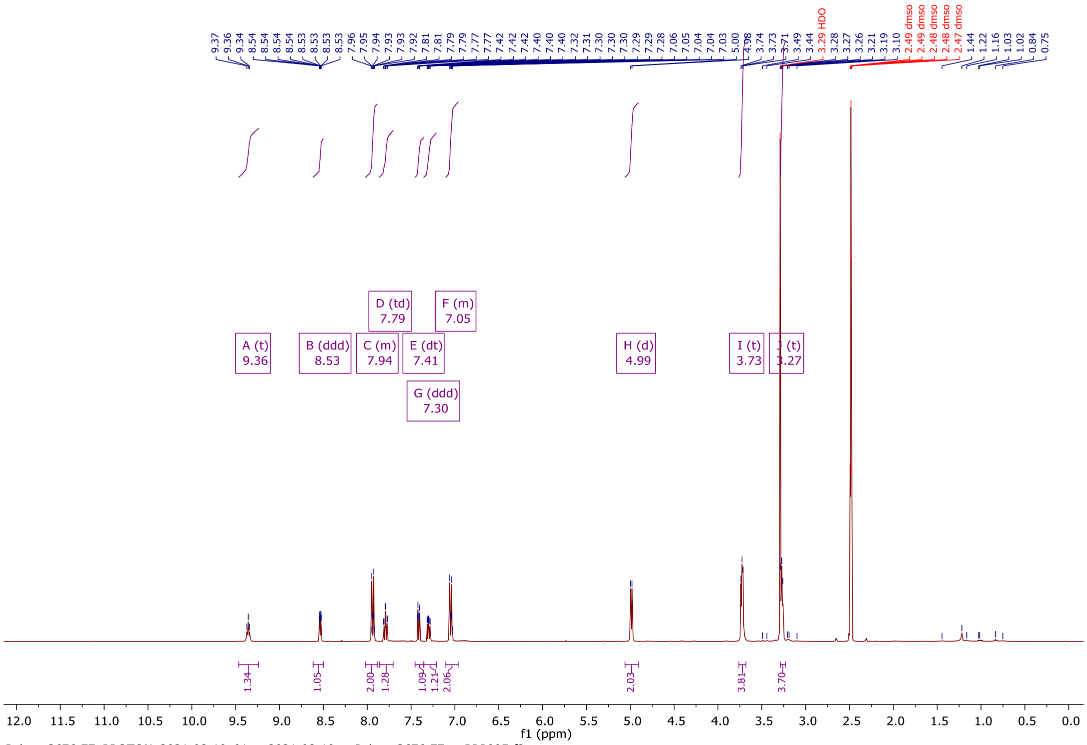

Palme_3676-77_PROTON_2021-08-10_01 — 2021-08-10 — Palme_3676-77 — PRP005-f2 —

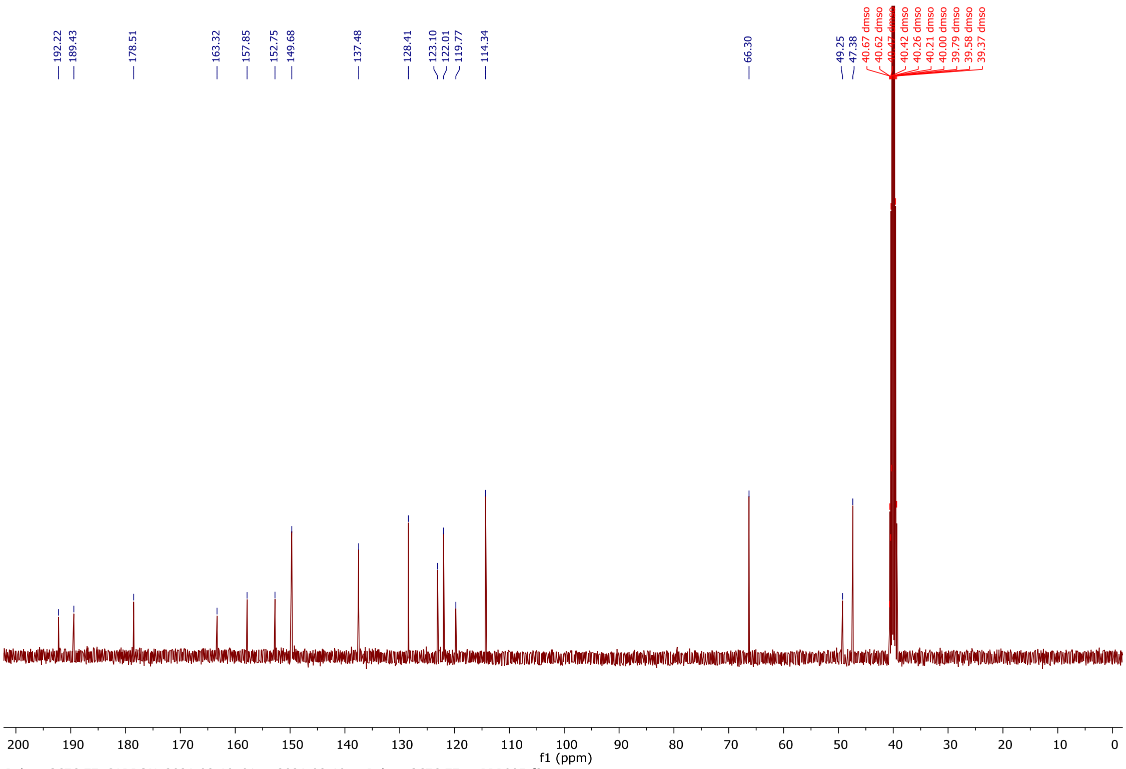

Palme_3676-77_CARBON_2021-08-10_01 — 2021-08-10 — Palme_3676-77 — PRP005-f2 —

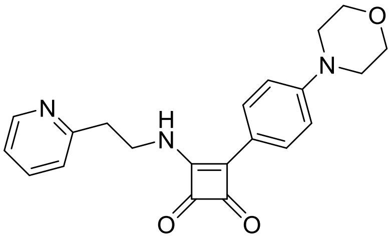

PRP001

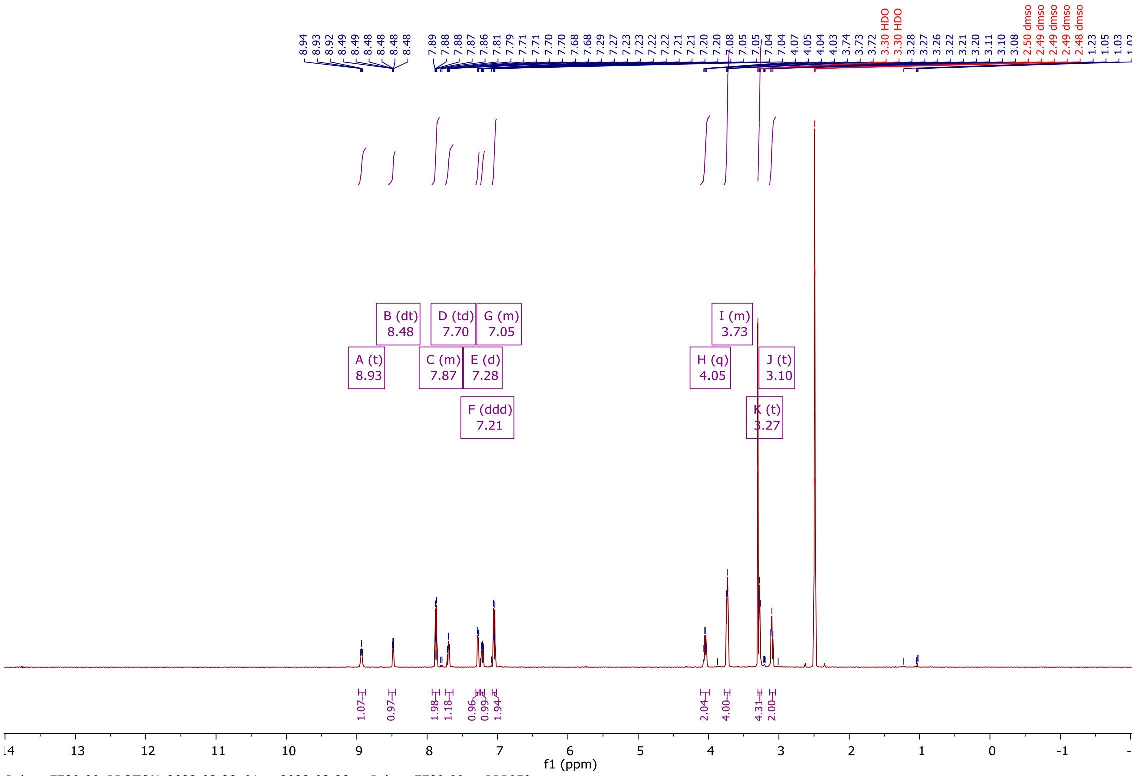

Palme_7789-90_PROTON_2022-03-28_01 — 2022-03-28 — Palme_7789-90 — PRP059 —

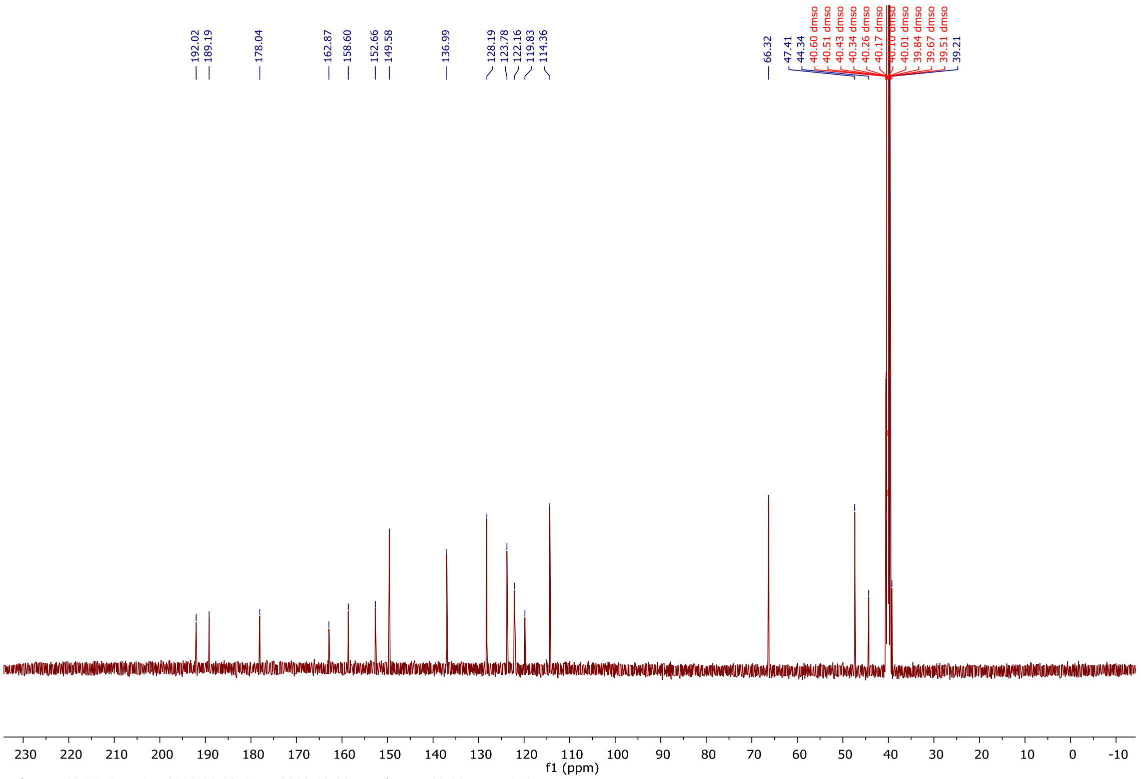

Palme_7789-90_CARBON_2022-03-28_01 — 2022-03-28 — Palme_7789-90 — PRP059 —

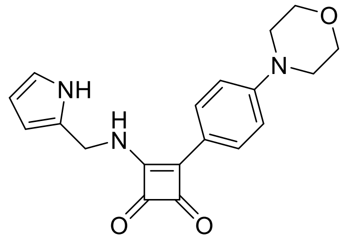

PRP002

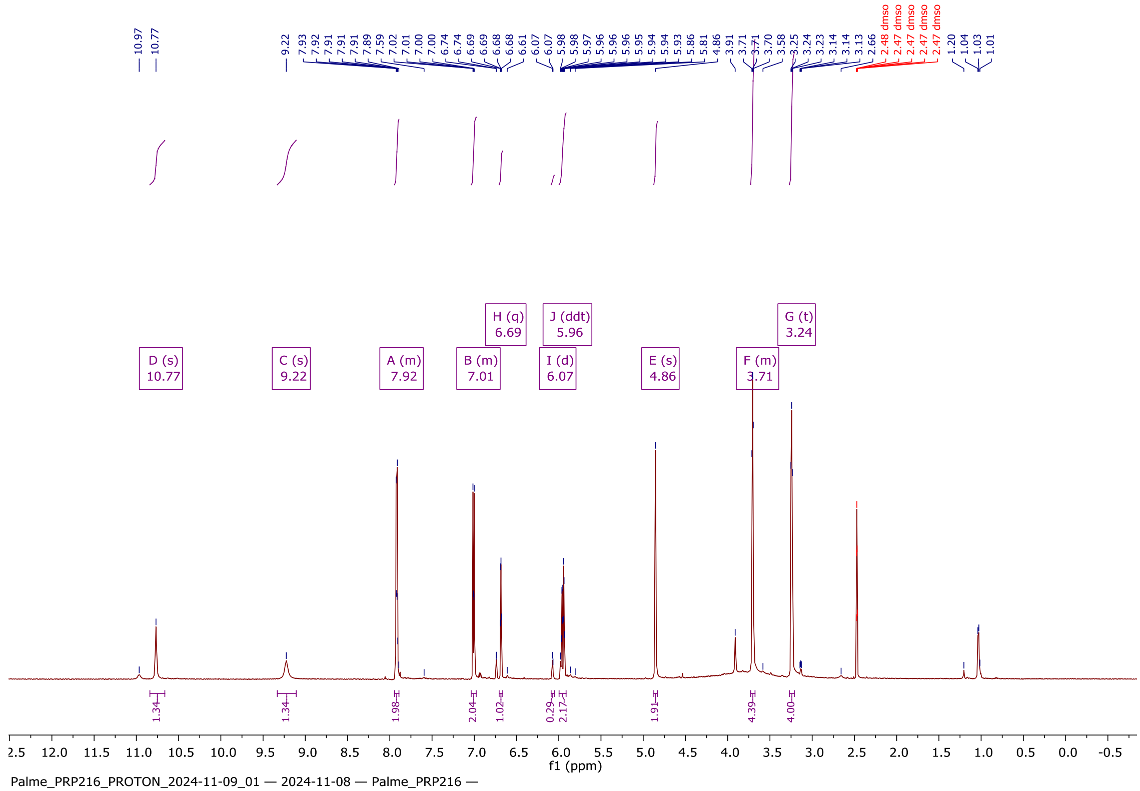

Palme_PRP216_PROTON_2024-11-09_01 — 2024-11-08 — Palme_PRP216 —

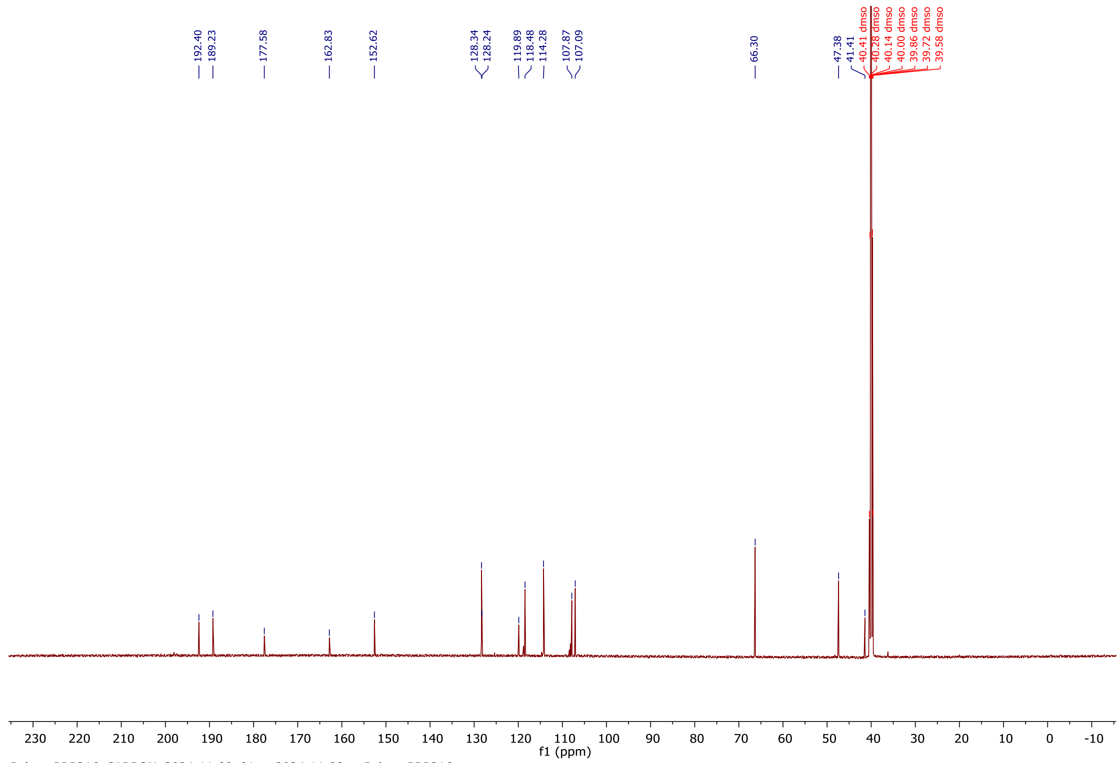

Palme_PRP216_CARBON_2024-11-09_01 — 2024-11-08 — Palme_PRP216 —

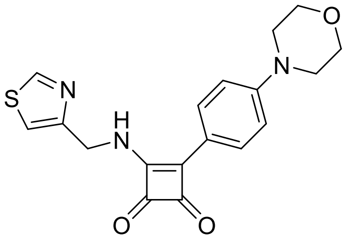

PRP003

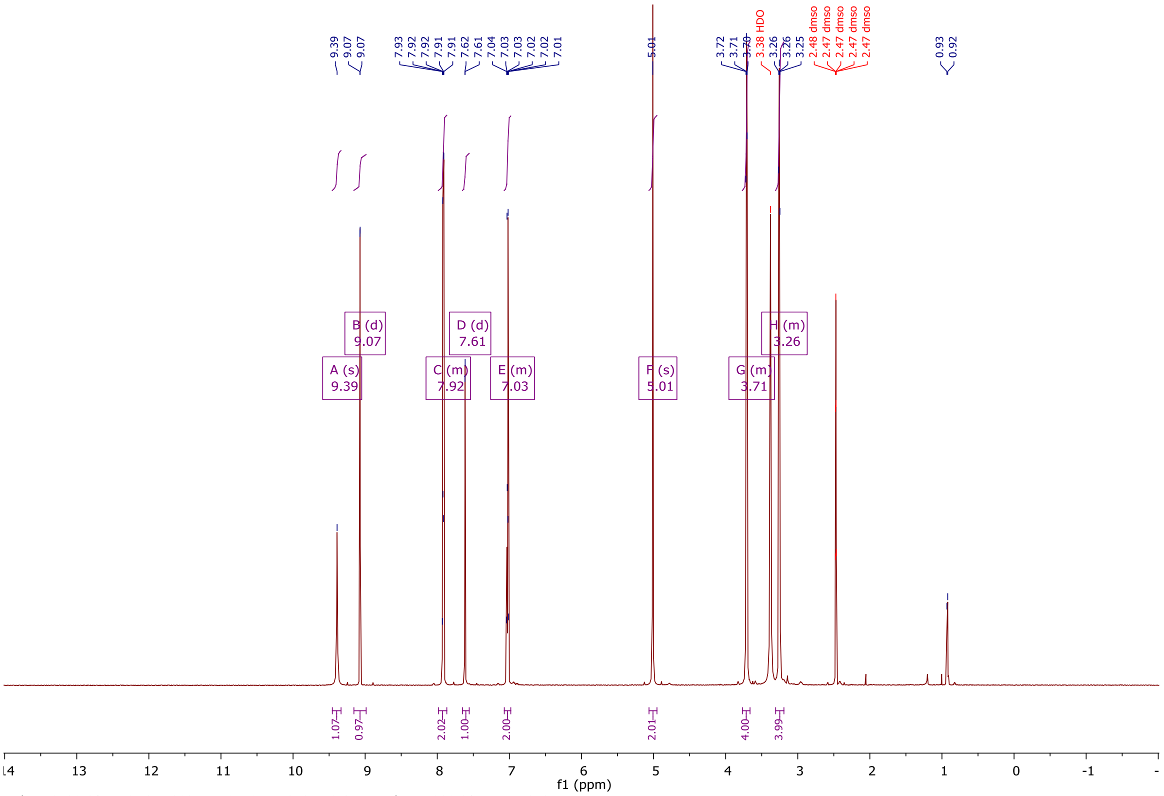

Palme_9920-38_PROTON_2024-11-01_04 — 2024-10-30 — Palme_9920-38 — PRPJ213 —

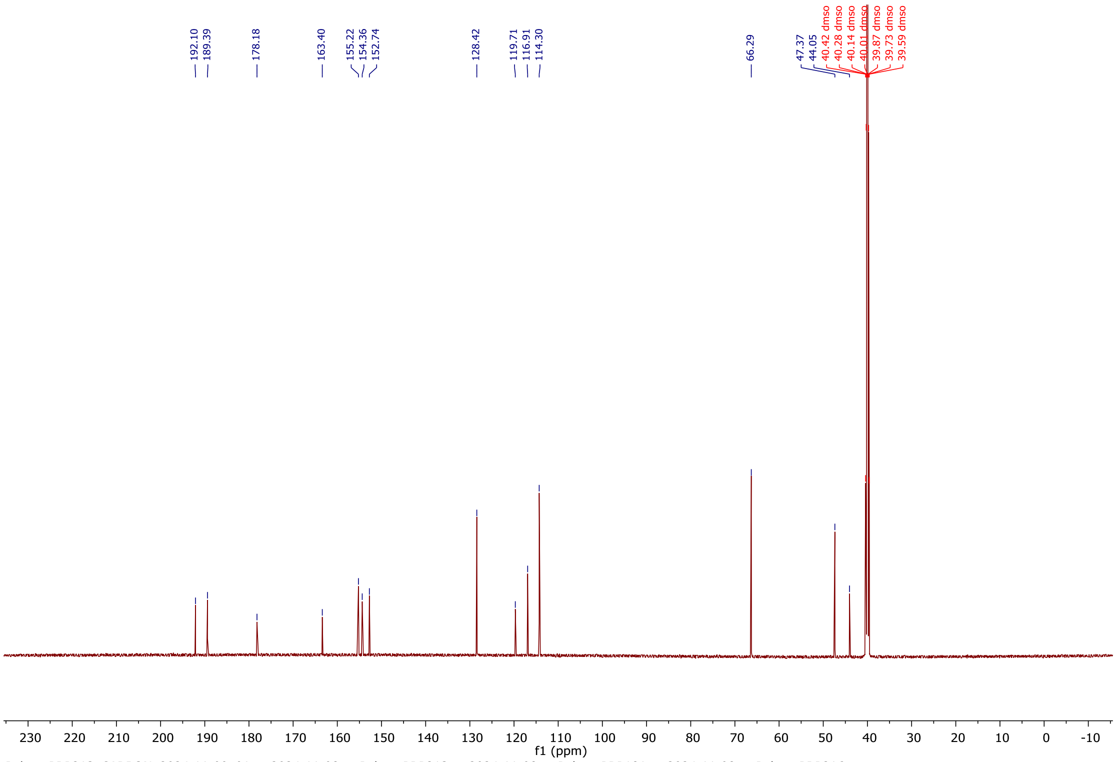

Palme_PRP213_CARBON_2024-11-09_01 — 2024-11-08 — Palme_PRP213 — 2024-11-08 — Palme_PRP181 — 2024-11-08 — Palme_PRP216 —

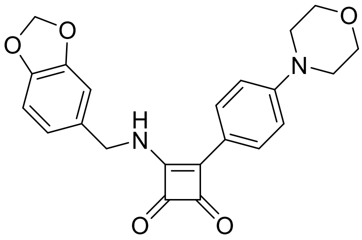

PRP004

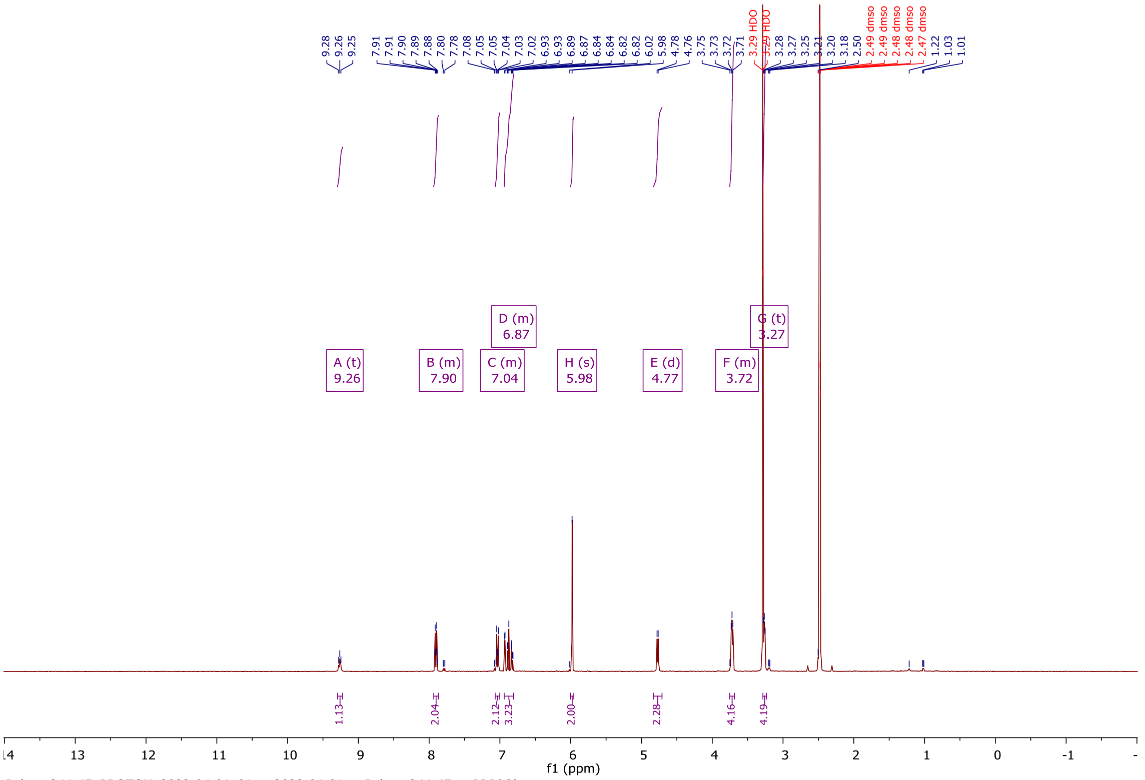

Palme_244-45_PROTON_2022-04-01_01 — 2022-04-01 — Palme_244-45 — PRP060 —

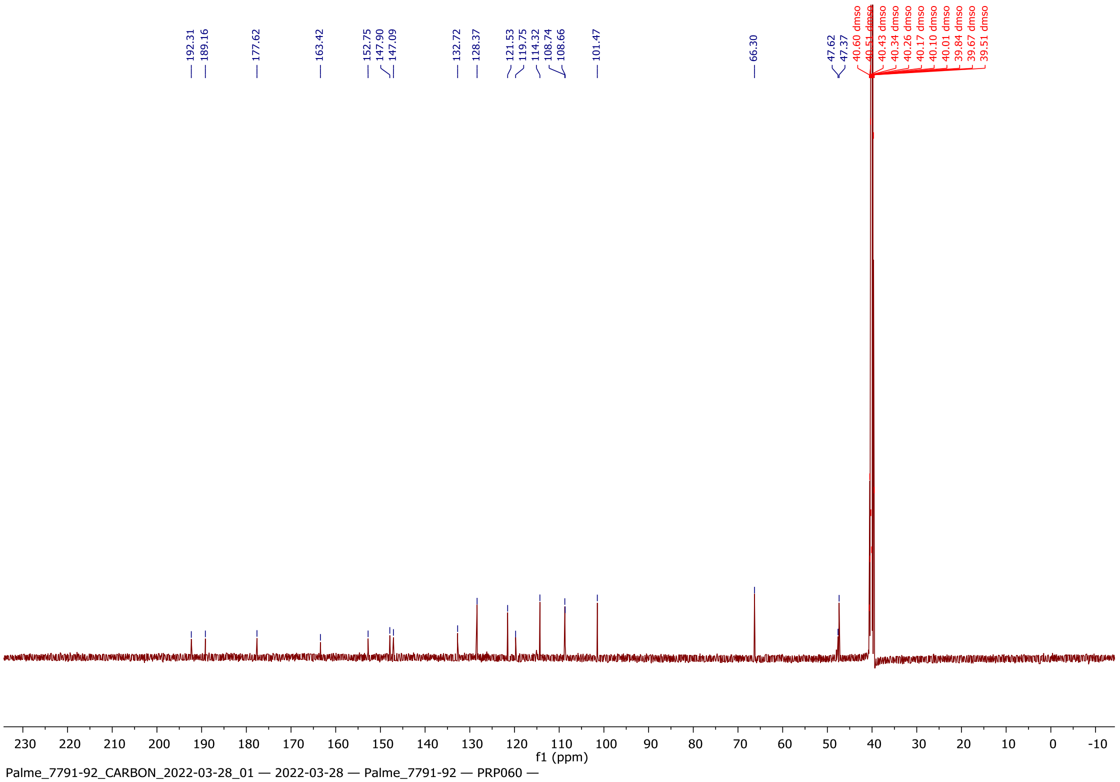

Palme_7791-92_CARBON_2022-03-28_01 — 2022-03-28 — Palme_7791-92 — PRP060 —

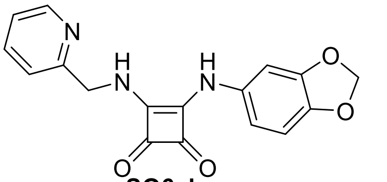

SQ6ab

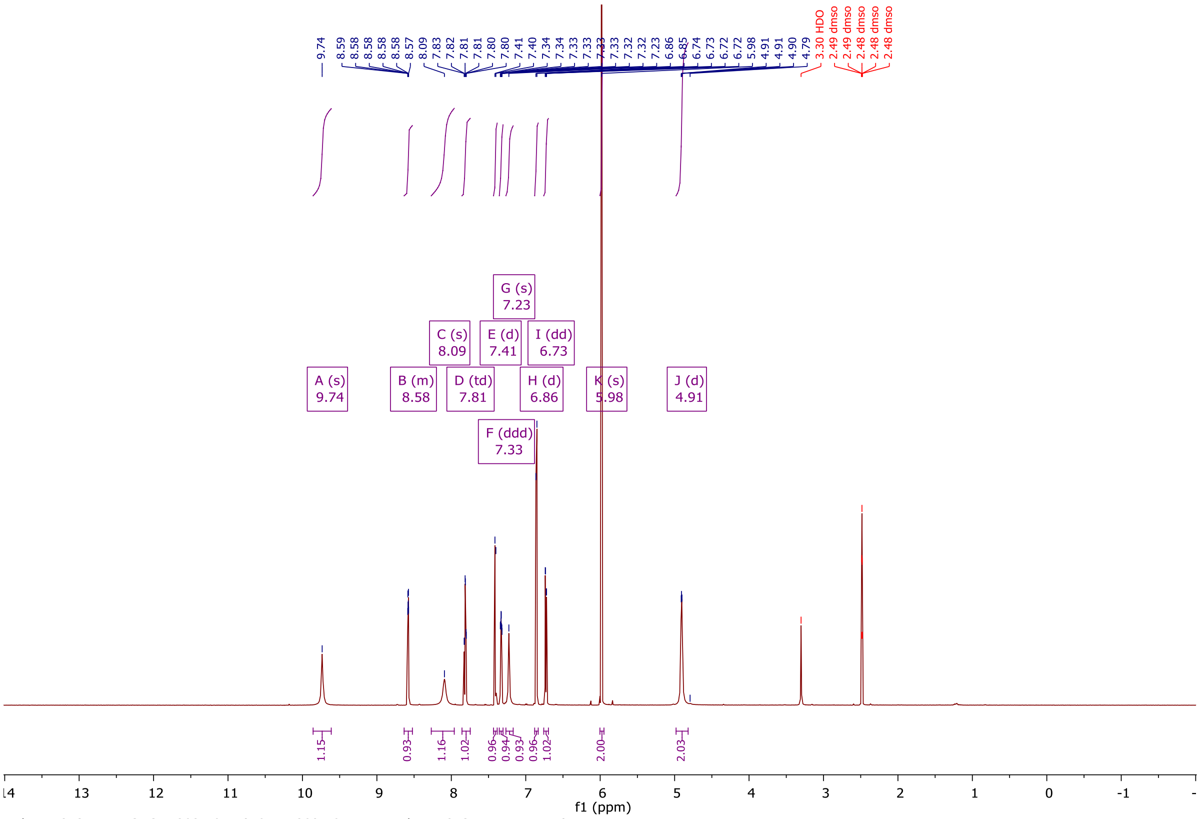

Palme_1253-54_PROTON_2025-04-18_01 — 2025-04-17 — Palme_1253-54 — PRPJ148 —

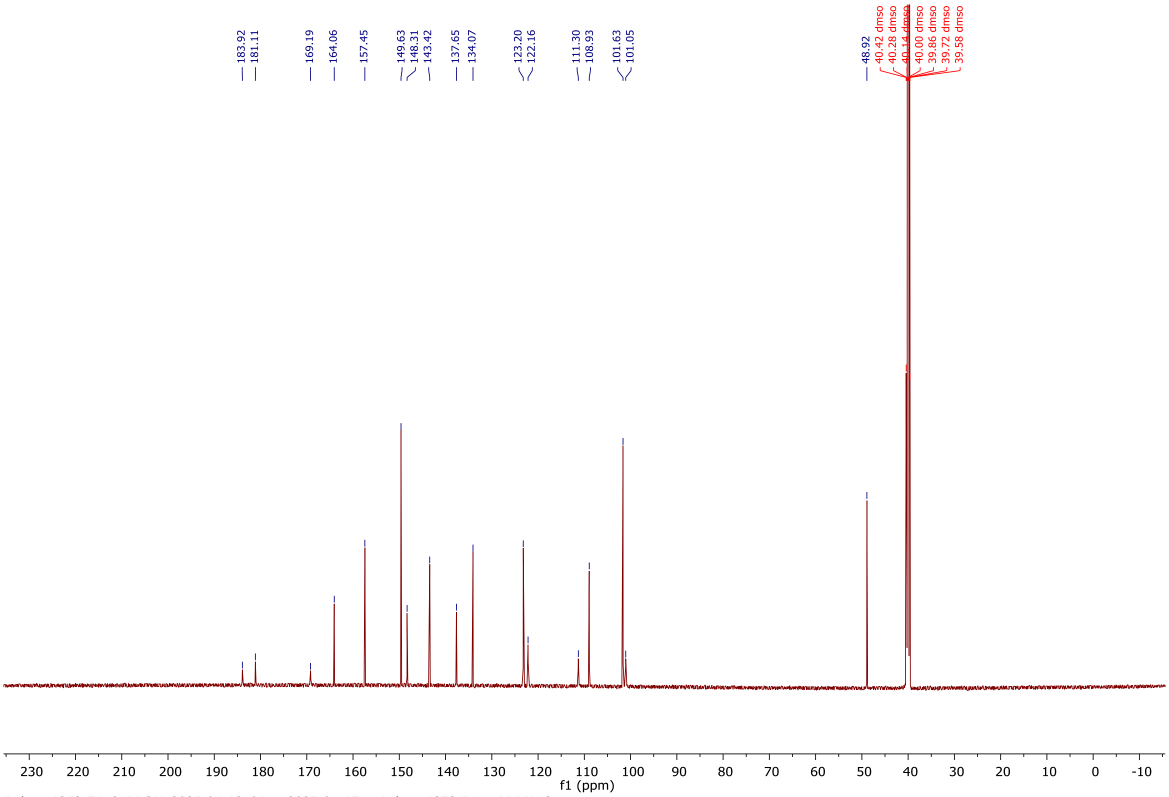

Palme_1253-54_CARBON_2025-04-18_01 — 2025-04-17 — Palme_1253-54 — PRPJ148 —

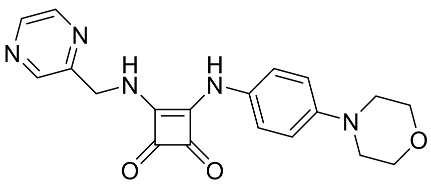

PRP005

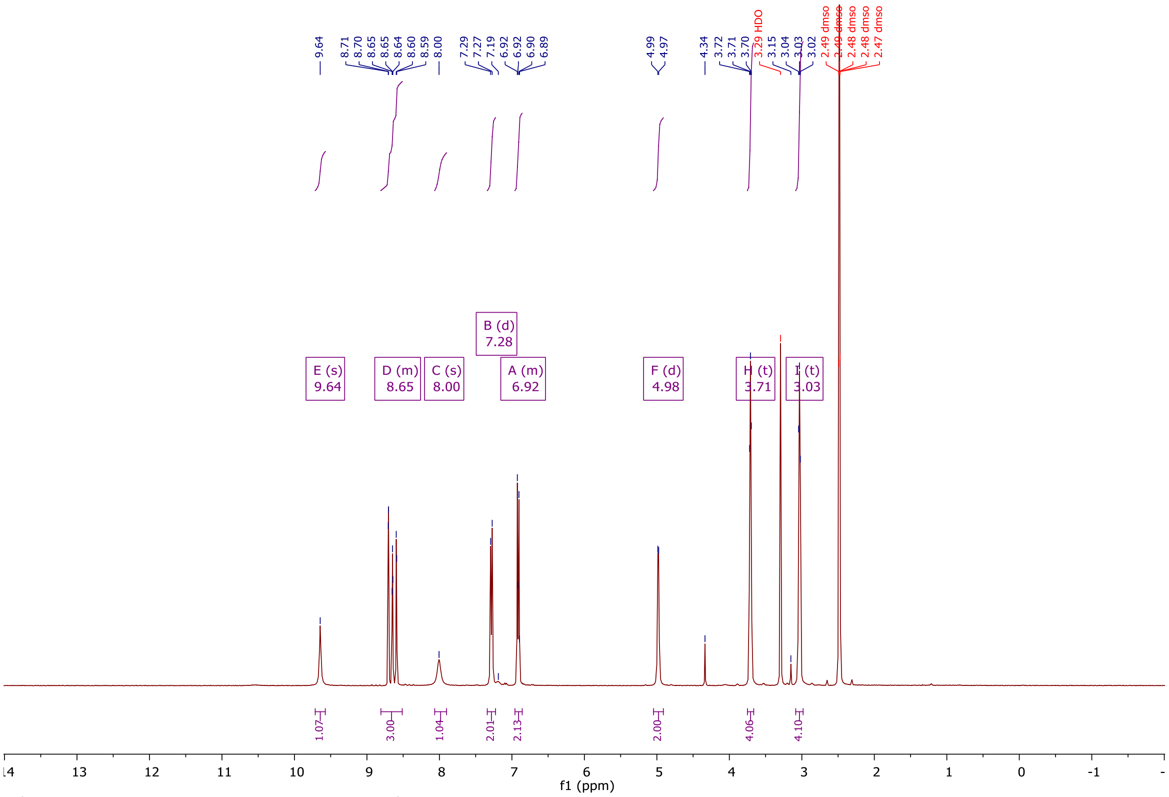

Palme_3275-77_PROTON_2022-08-11_01 — 2022-08-11 — Palme_3275-77 — PRP079 —

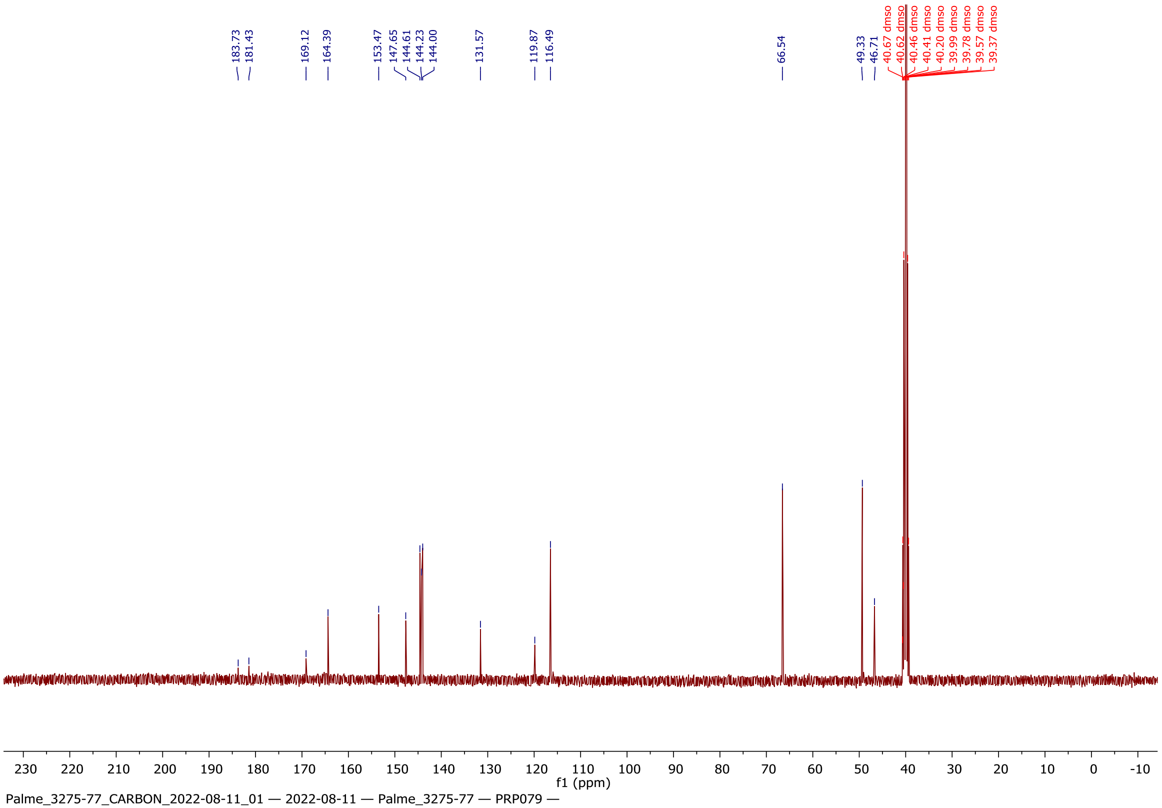

Palme_3275-77_CARBON_2022-08-11_01 — 2022-08-11 — Palme_3275-77 — PRP079 —

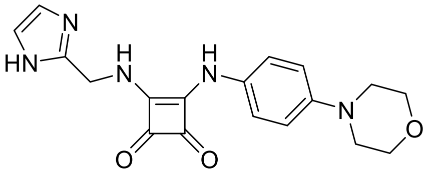

PRP006

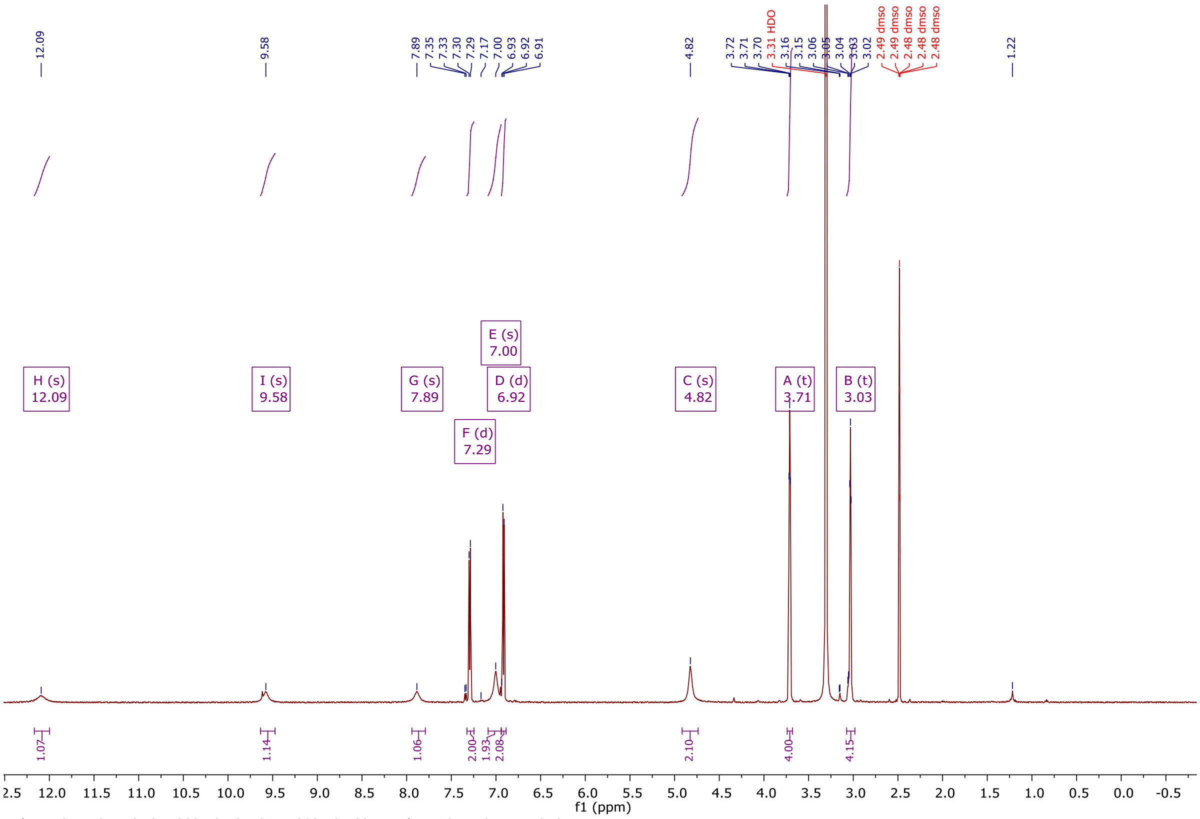

Palme_1057-59_PROTON_2025-04-04_01 — 2025-04-02 — Palme_1057-59 — PRP076 —

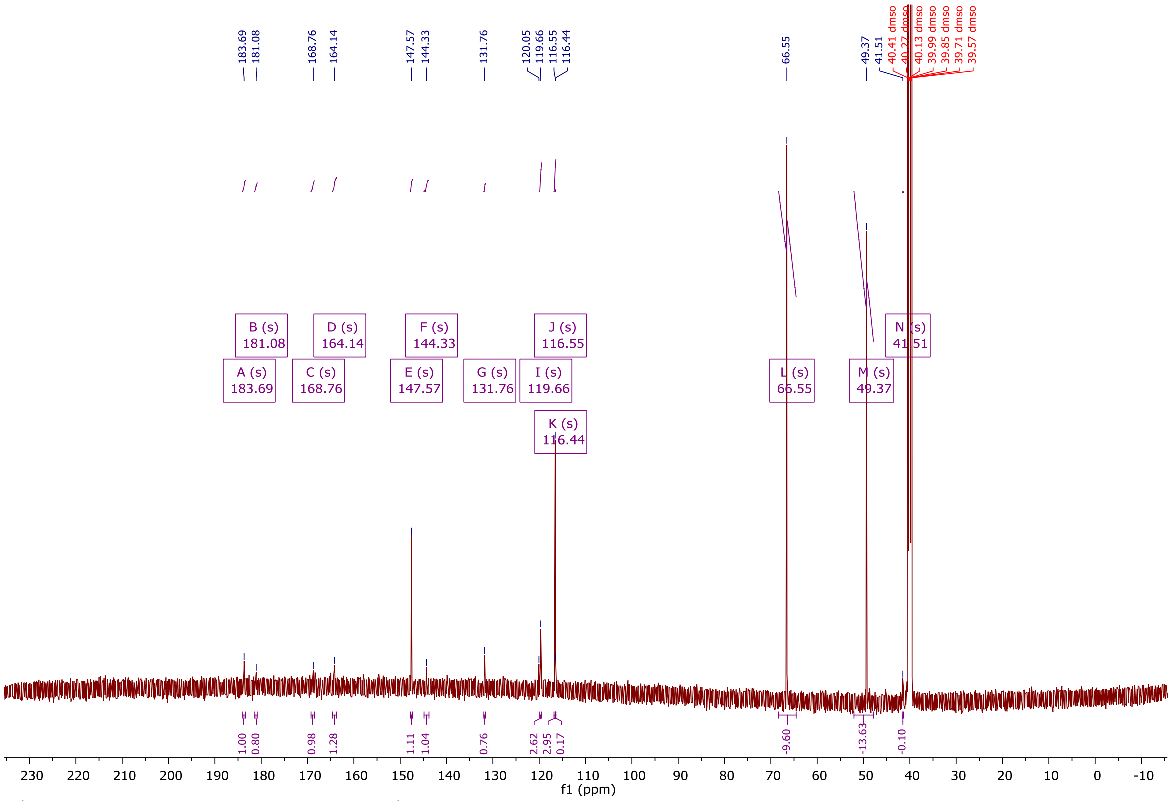

Palme_1057-59_CARBON_2025-04-03_01 — 2025-04-02 — Palme_1057-59 — PRP076 —

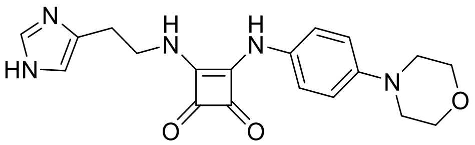

PRP007

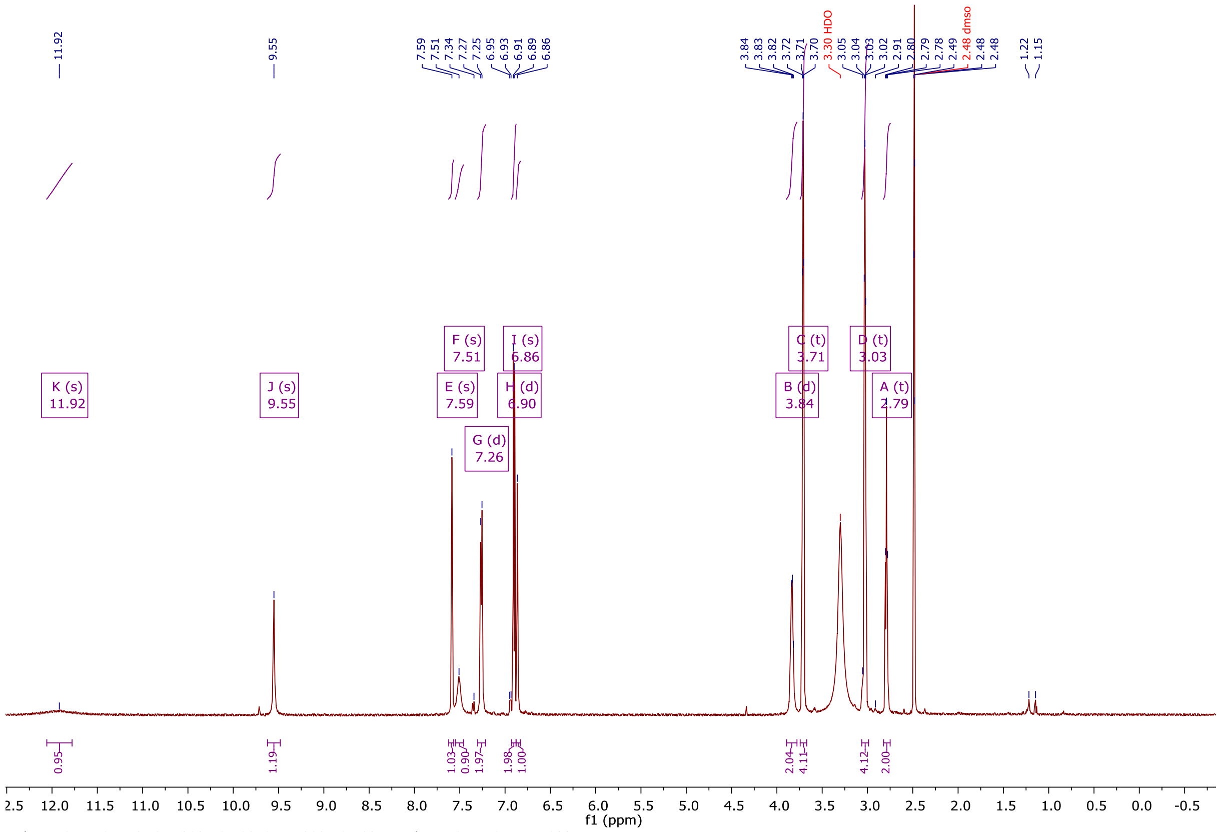

Palme_1054-56_PROTON_2025-04-03_01 — 2025-04-02 — Palme_1054-56 — PRP088 —

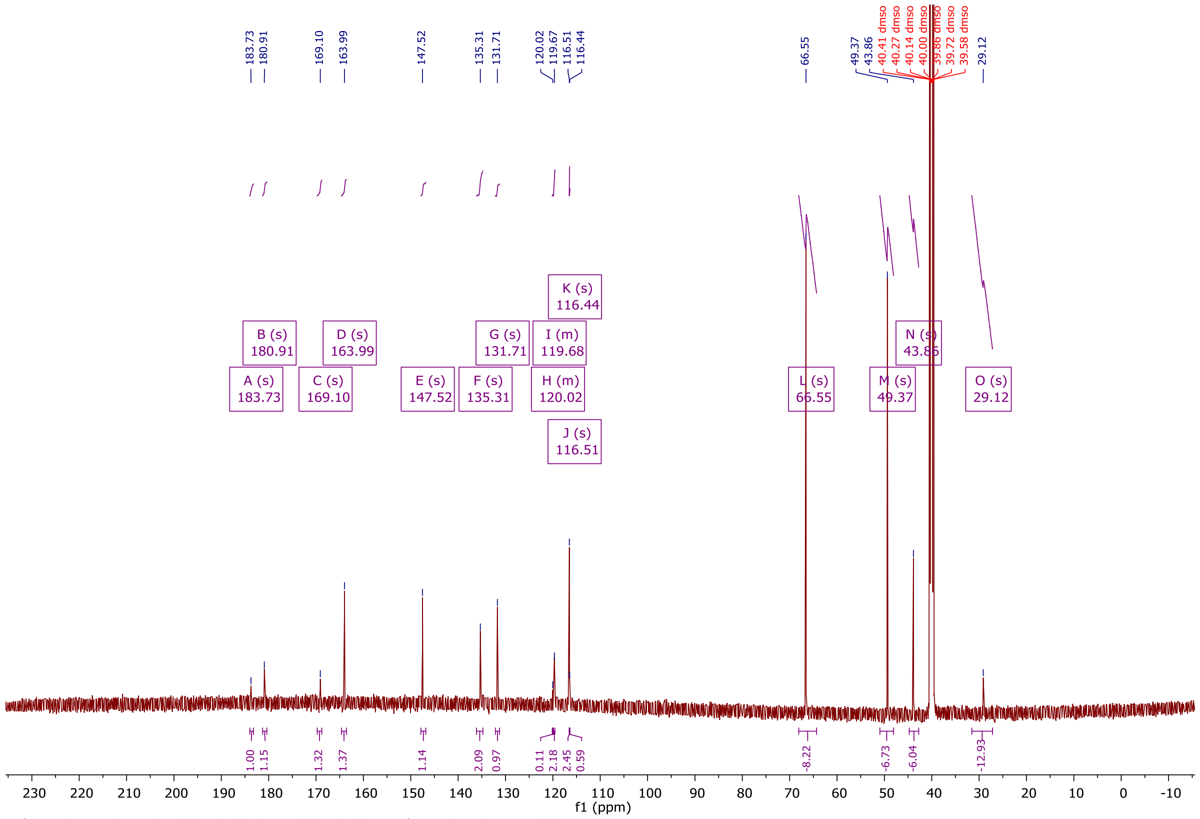

Palme_1054-56_CARBON_2025-04-03_01 — 2025-04-02 — Palme_1054-56 — PRP088 —

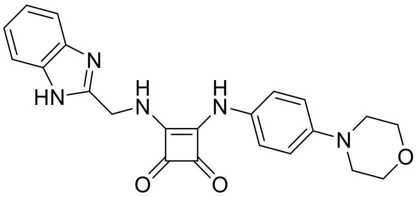

PRP008

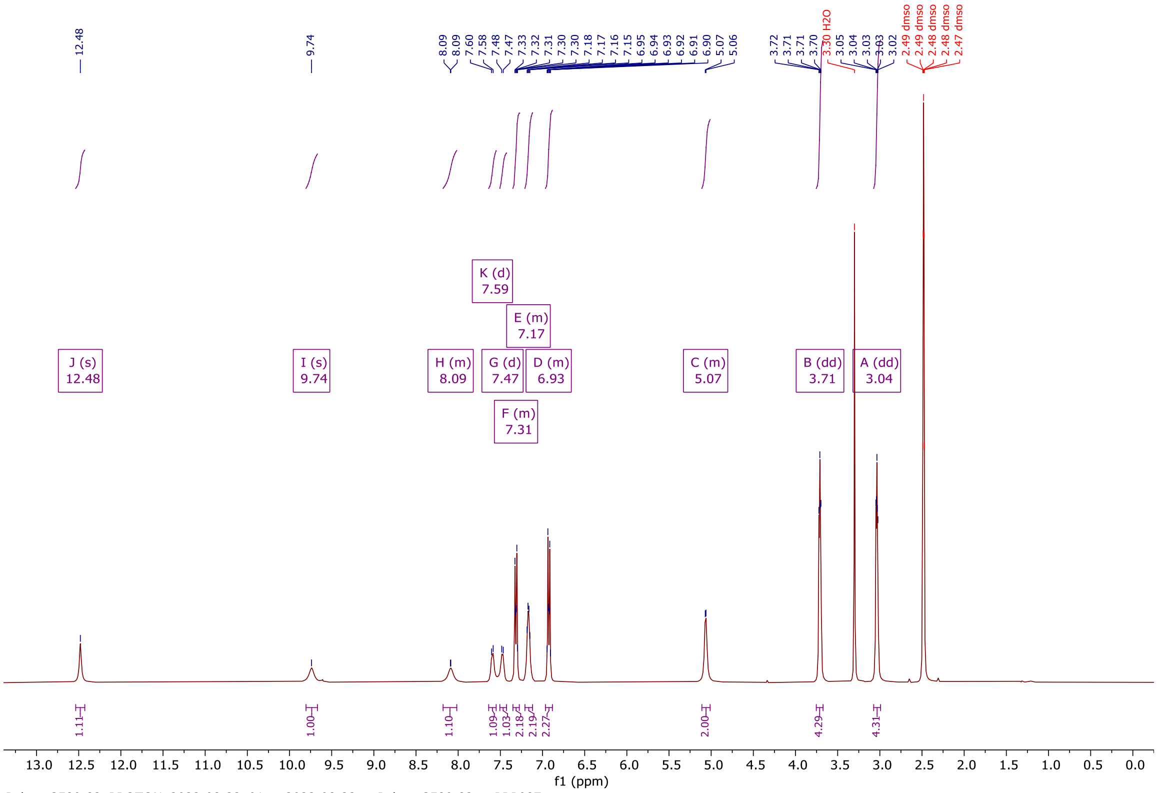

Palme_3580-82_PROTON_2022-08-22_01 — 2022-08-22 — Palme_3580-82 — PRP087 —

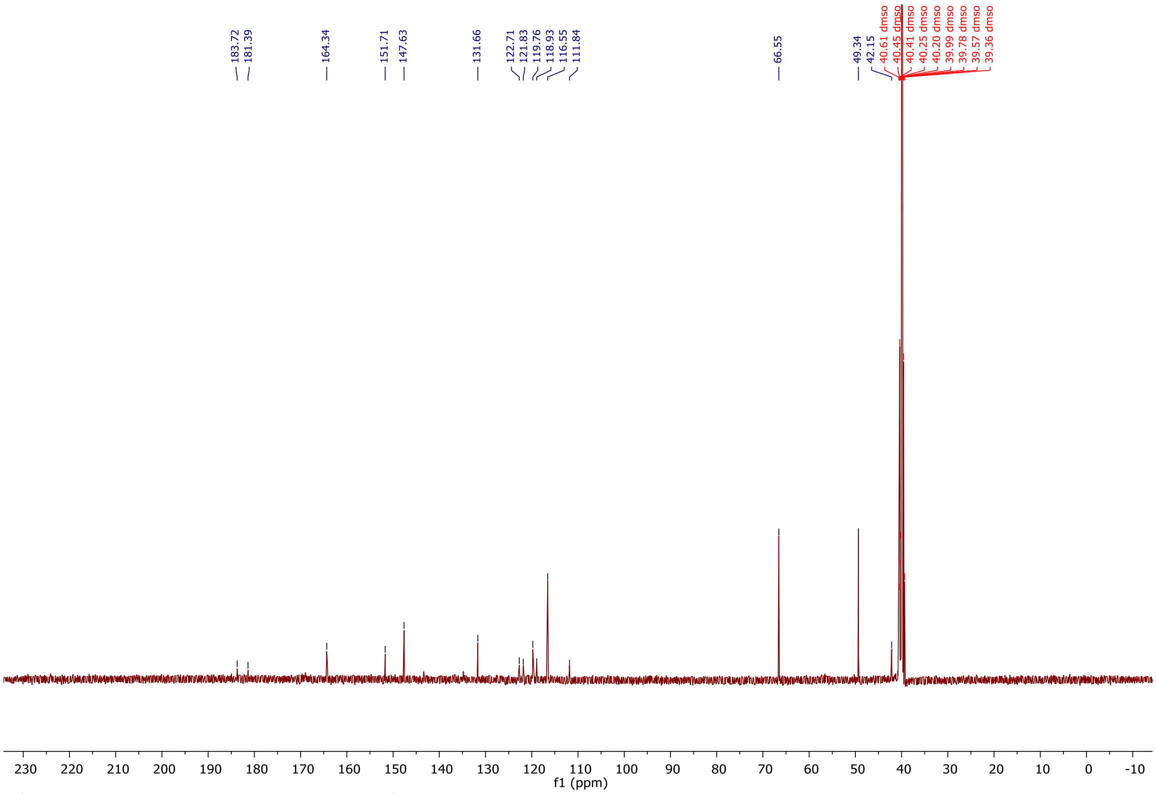

Palme_3580-82_CARBON_2022-08-22_01 — 2022-08-22 — Palme_3580-82 — PRP087 —

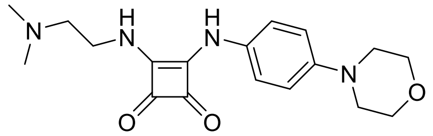

PRP009

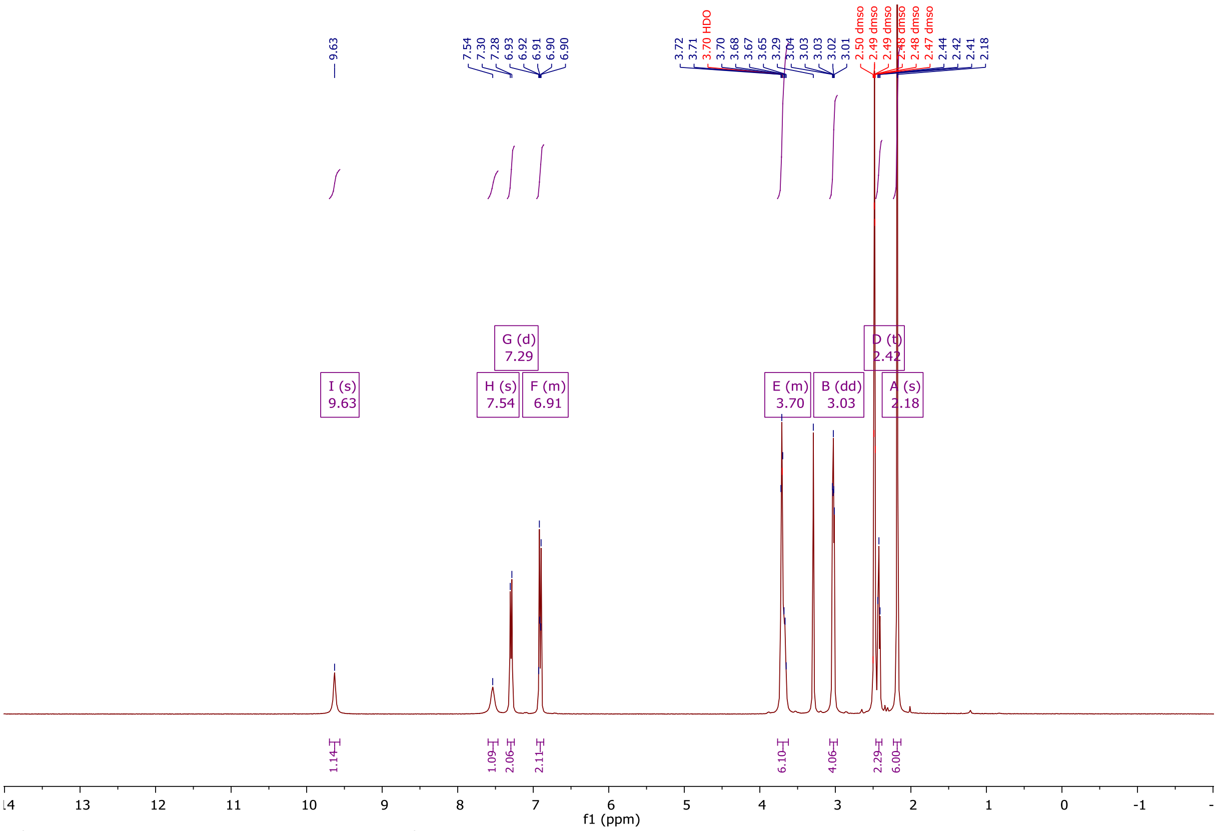

Palme_3583-85_PROTON_2022-08-22_01 — 2022-08-22 — Palme_3583-85 — PRP086 —

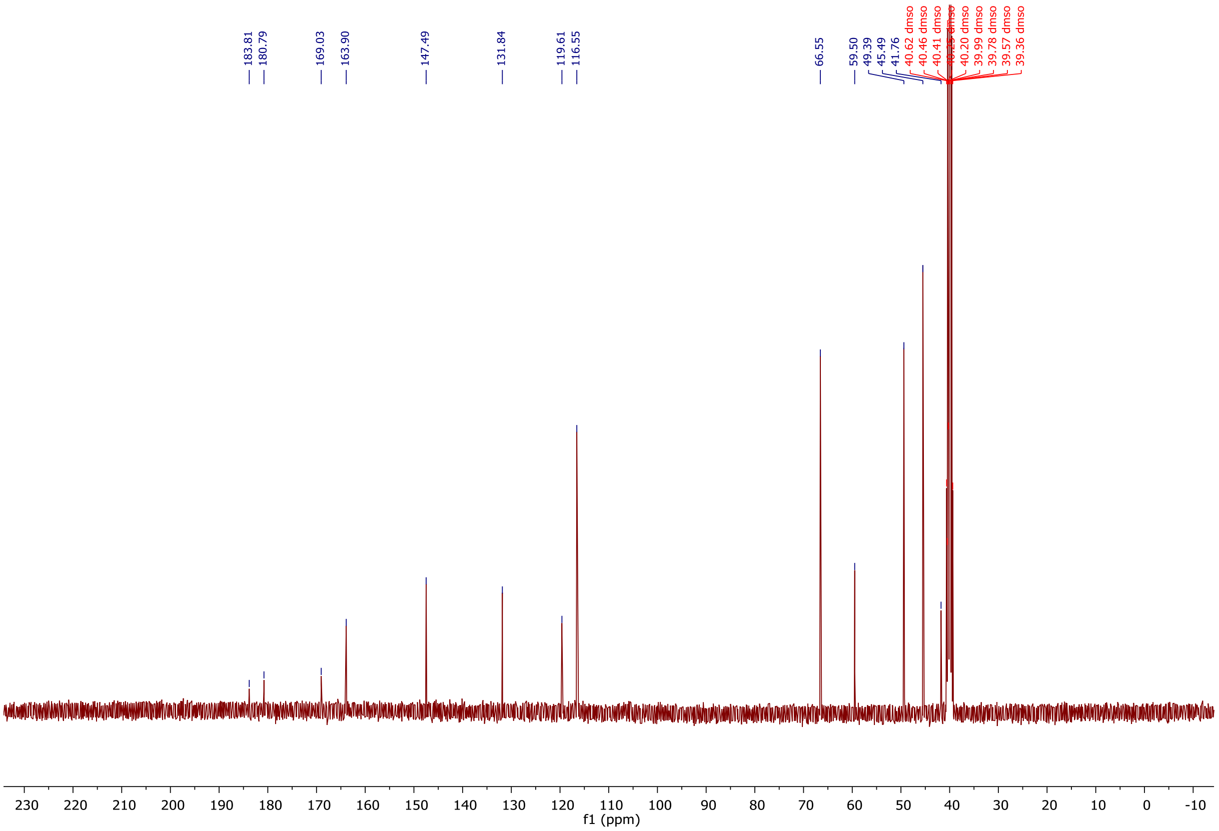

Palme_3583-85_CARBON_2022-08-22_01 — 2022-08-22 — Palme_3583-85 — PRP086 —

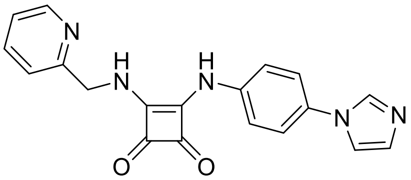

PRP010

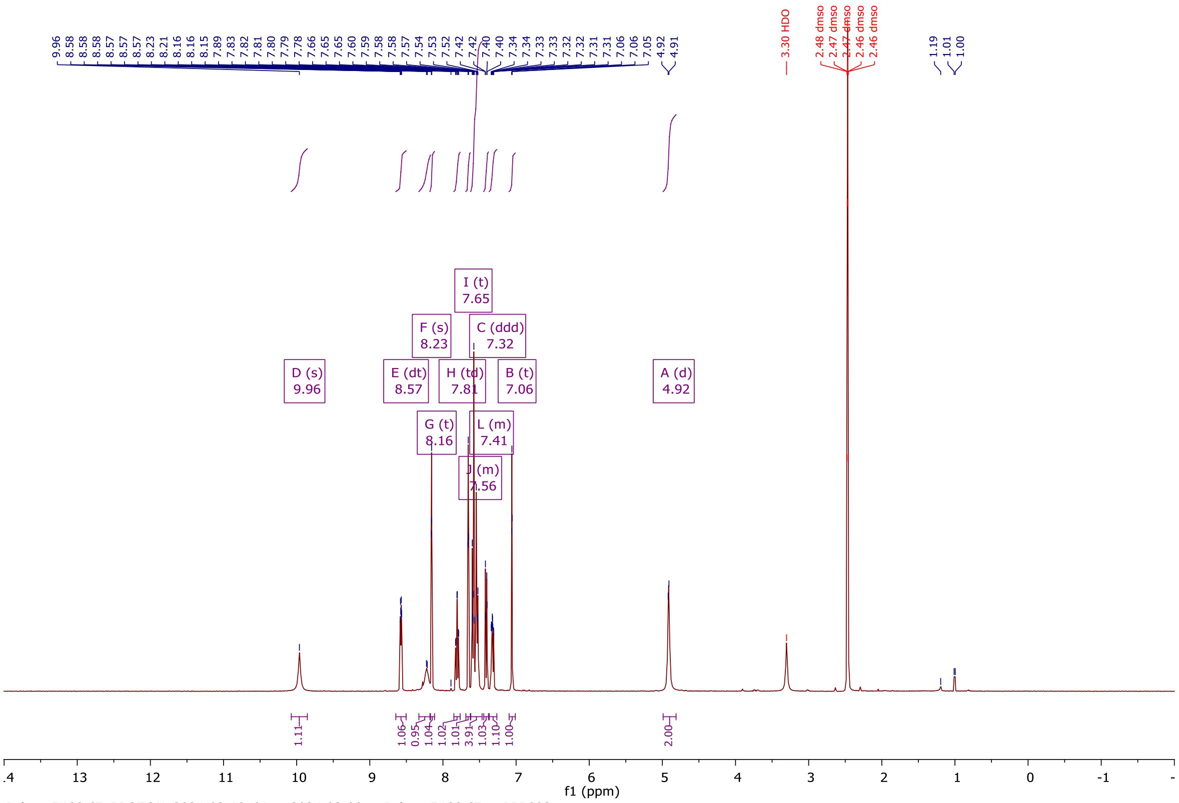

Palme_5466-67_PROTON_2024-02-10_01 — 2024-02-09 — Palme_5466-67 — PRP093 —

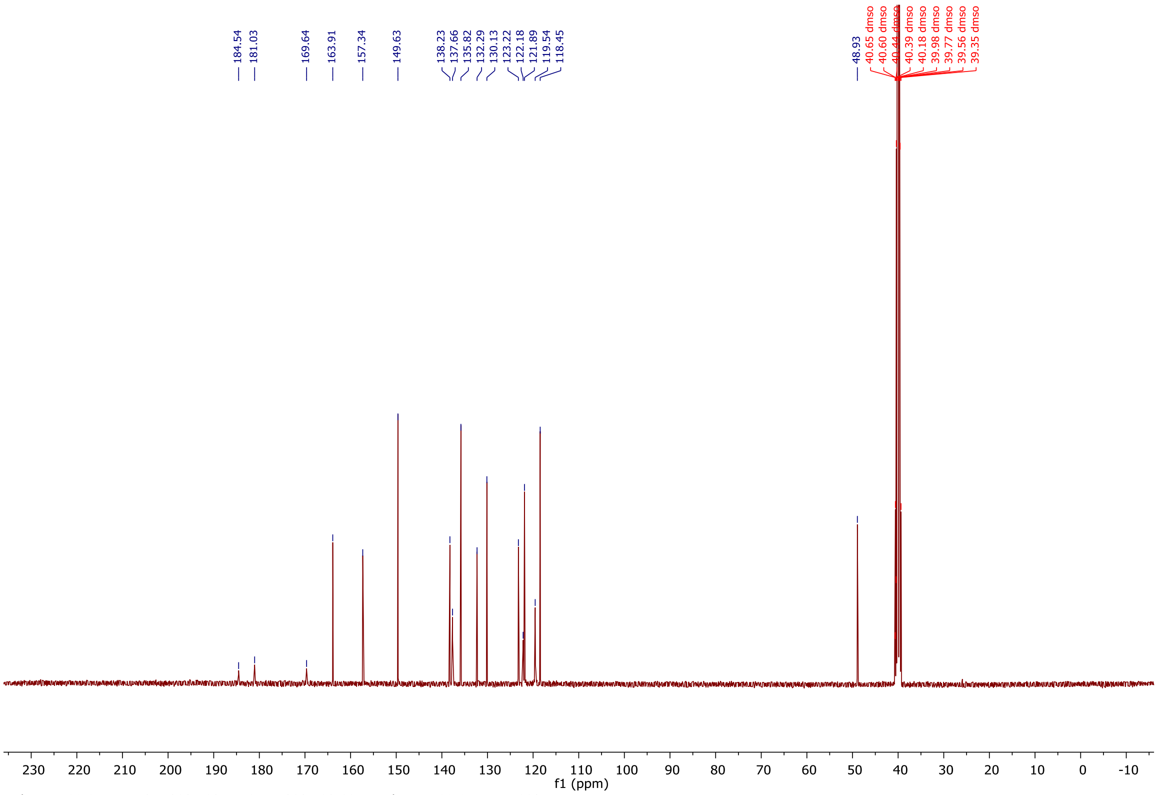

Palme_5466-67_CARBON_2024-02-11_01 — 2024-02-09 — Palme_5466-67 — PRP093 —

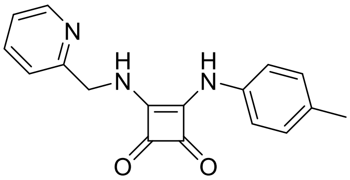

PRP011

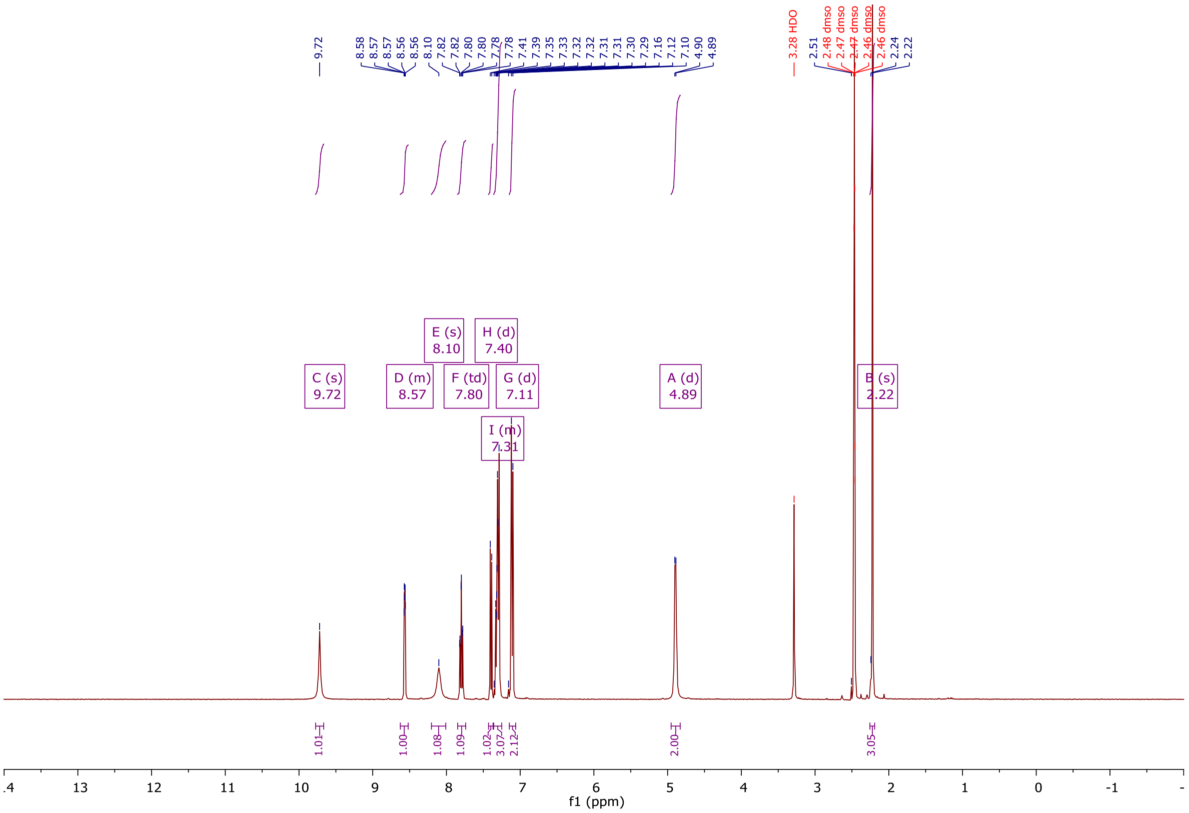

Seiser_3971-72_PROTON_2023-11-24_01 — 2023-11-23 — Seiser_3971-72 — PRPJ 147.2 Test —

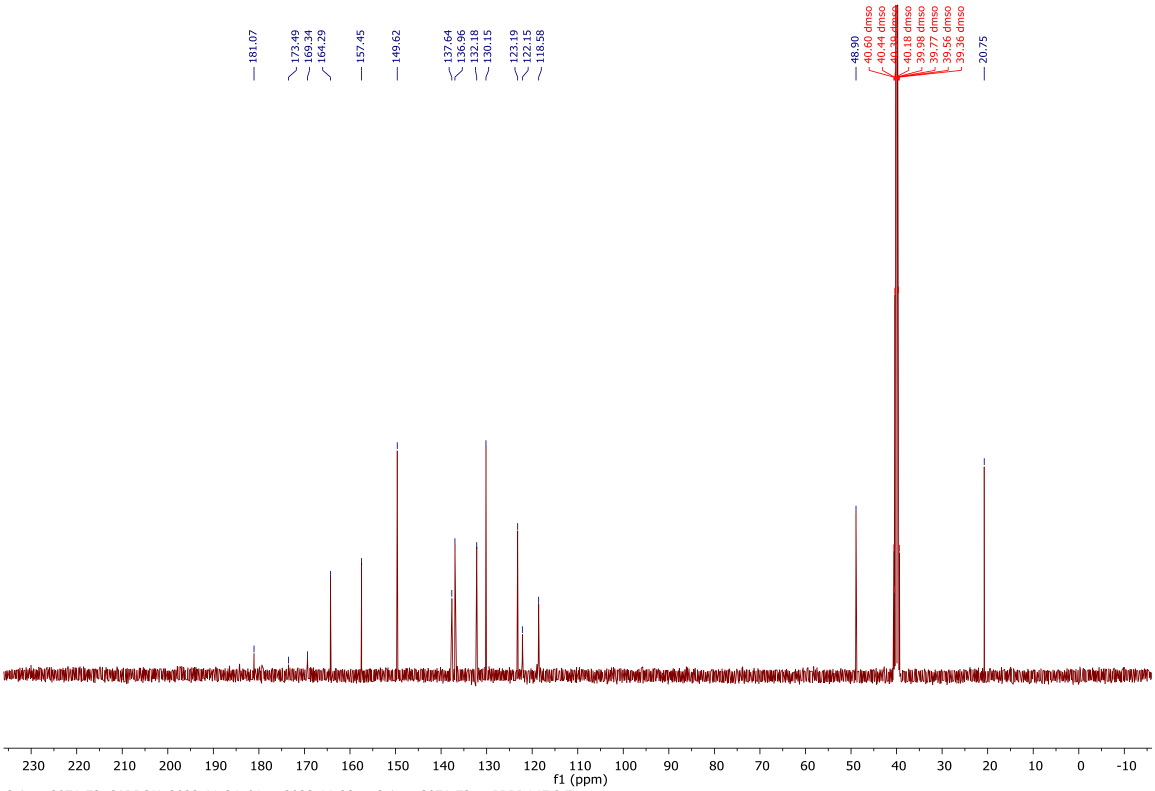

Seiser_3971-72_CARBON_2023-11-24_01 — 2023-11-23 — Seiser_3971-72 — PRPJ 147.2 Test —

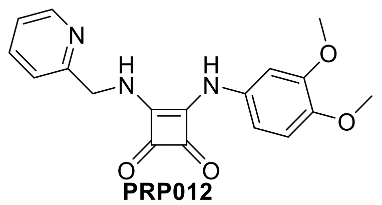

PRP012

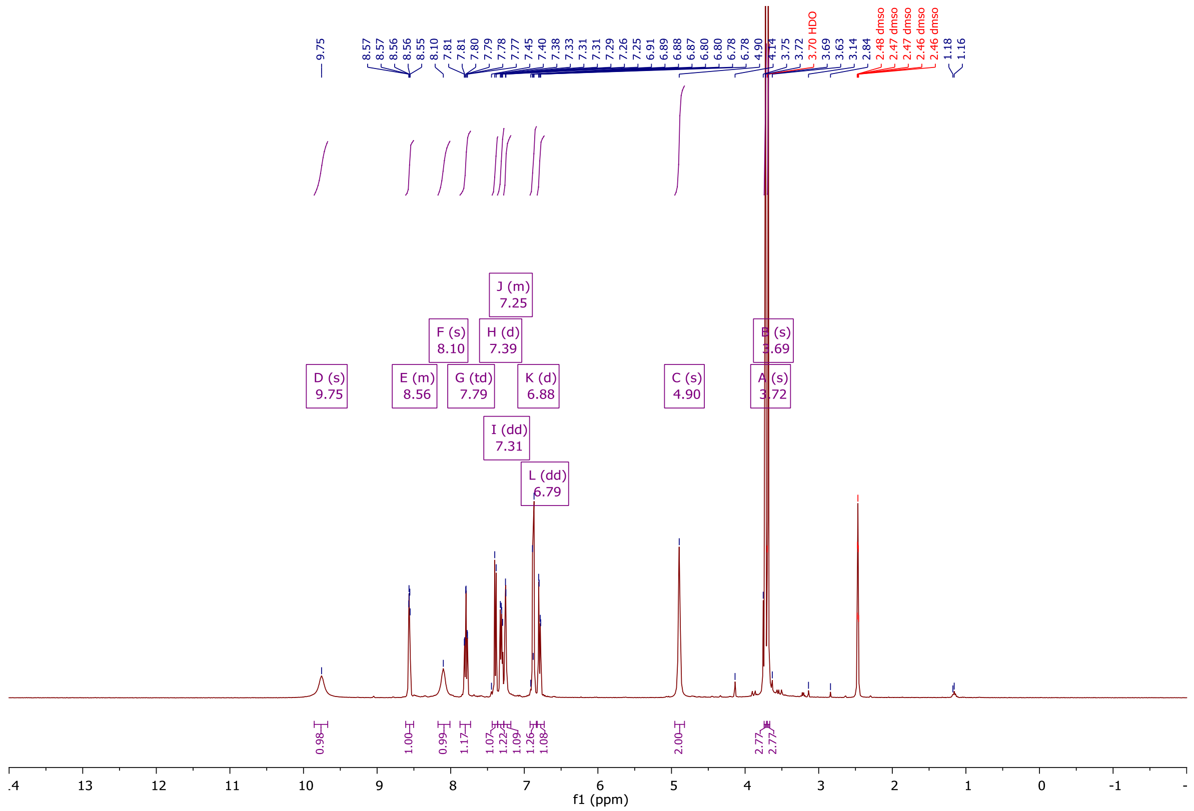

Seiser_4428-29_PROTON_2023-12-11_01 — 2023-12-11 — Seiser_4428-29 — PRPJ150.2 —

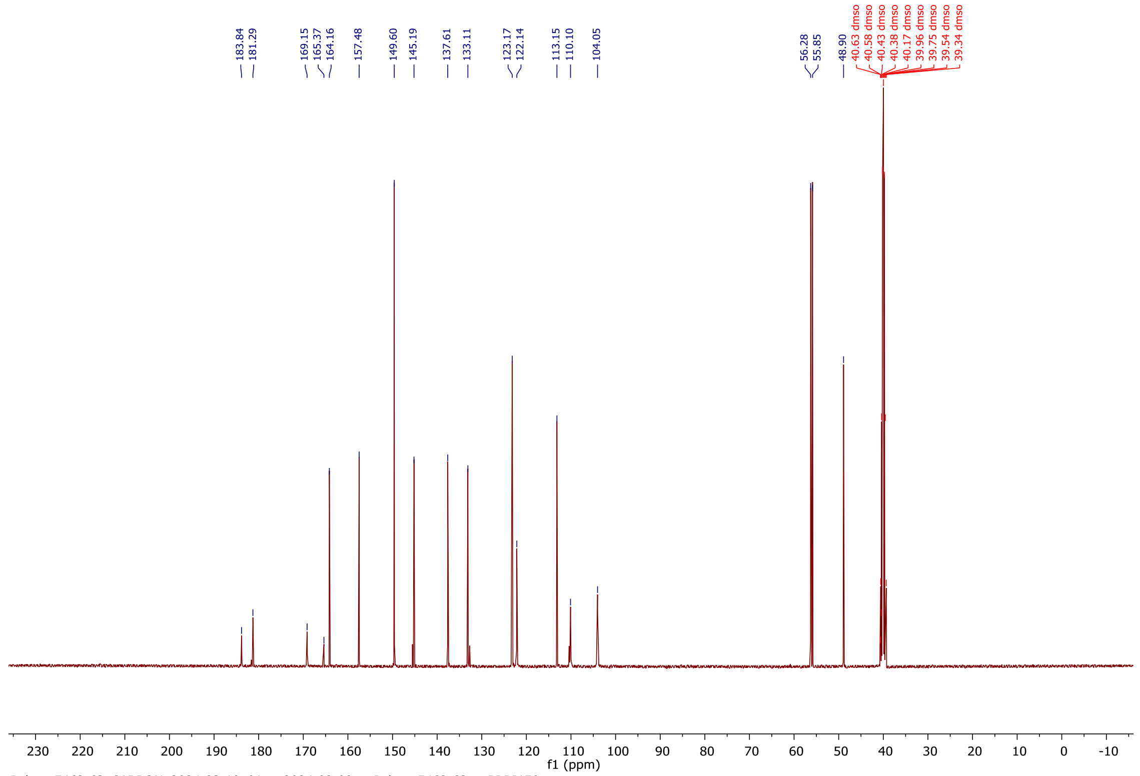

Palme_5462-63_CARBON_2024-02-10_01 — 2024-02-09 — Palme_5462-63 — PRPJ150 —

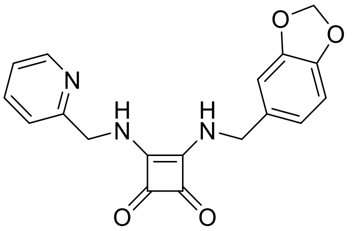

PRP013

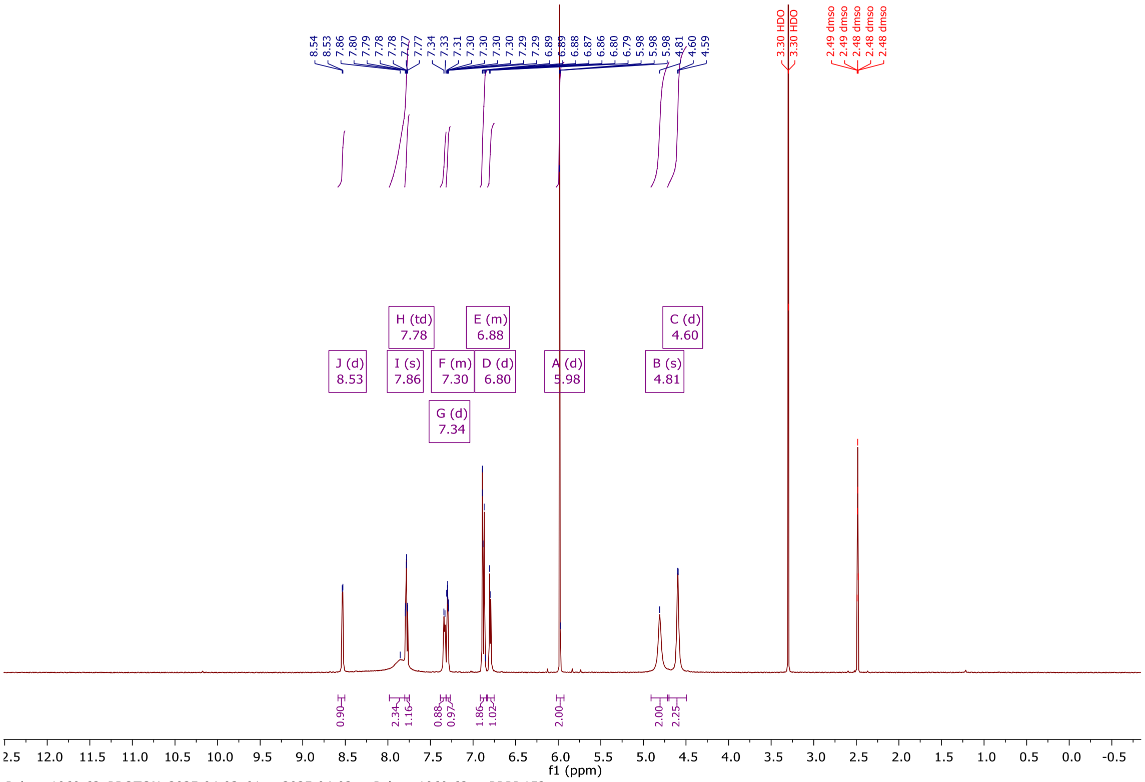

Palme_1060-62_PROTON_2025-04-03_01 — 2025-04-02 — Palme_1060-62 — PRPJ 152 —

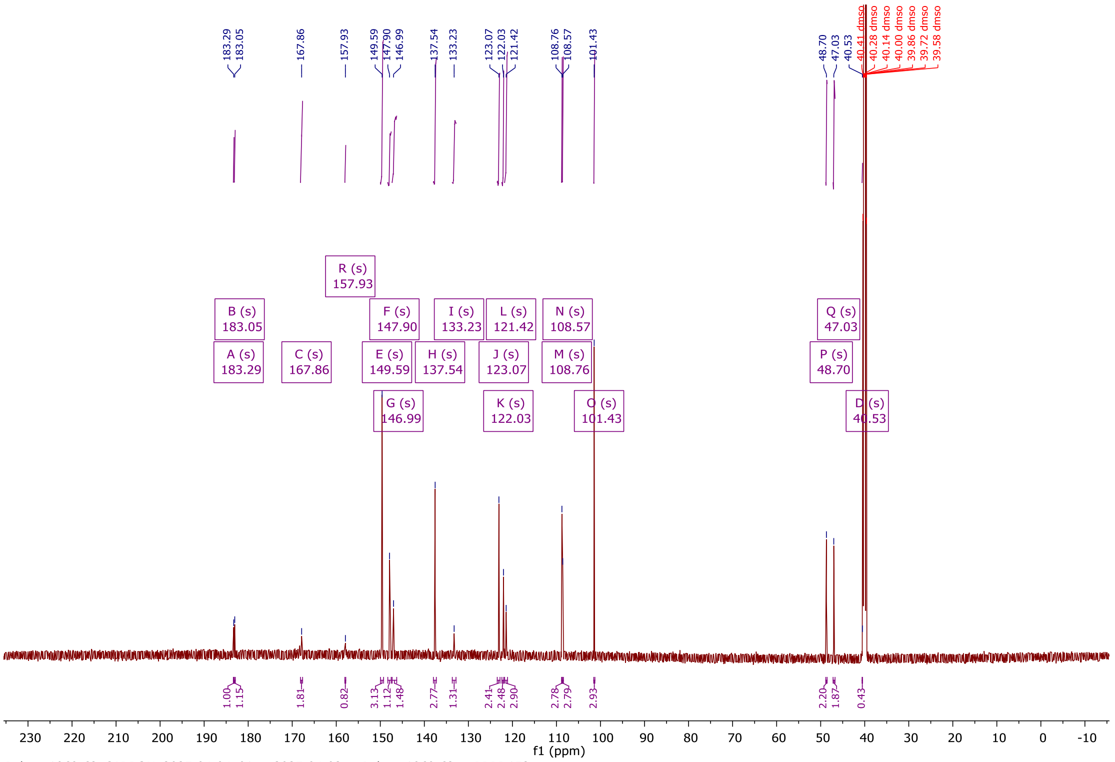

Palme_1060-62_CARBON_2025-04-04_01 — 2025-04-02 — Palme_1060-62 — PRPJ 152 —

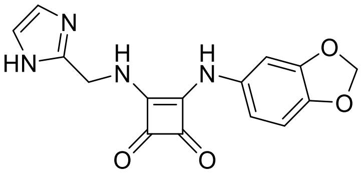

PRP014

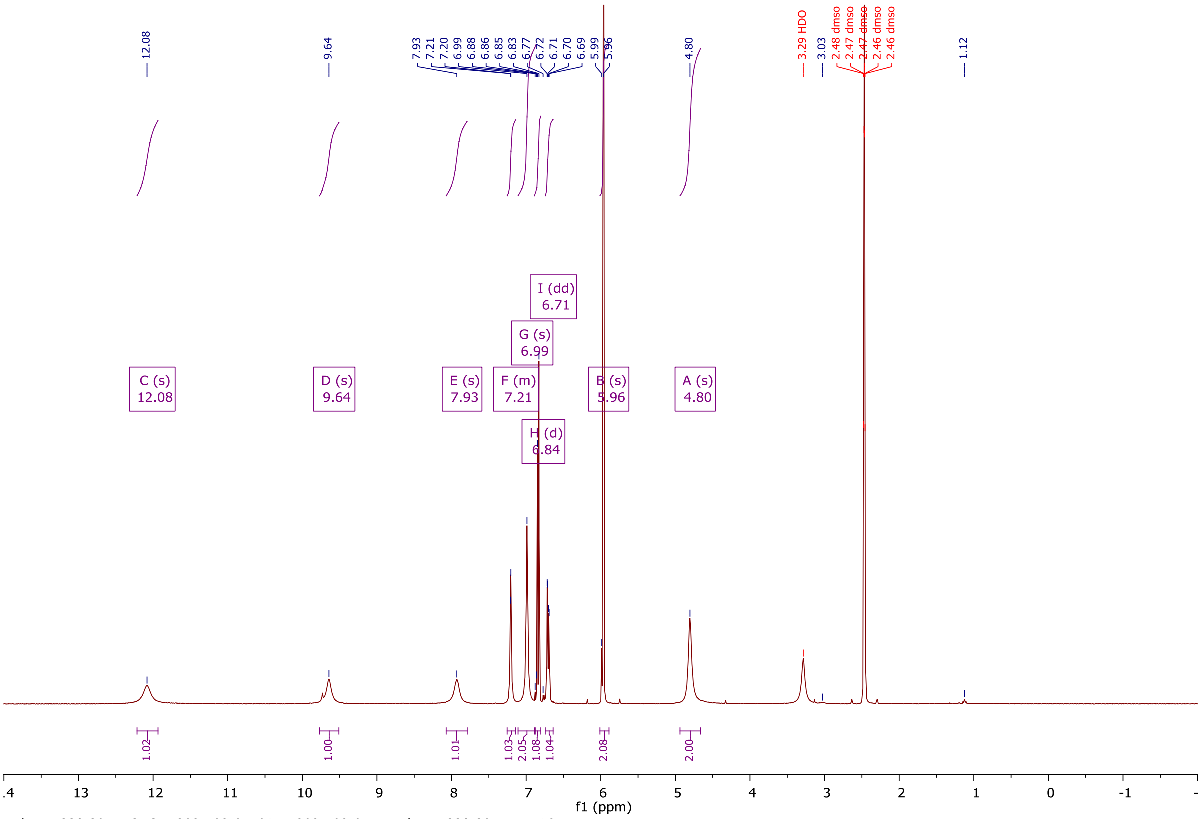

Palme_5338-39_PROTON_2024-02-05_01 — 2024-02-05 — Palme_5338-39 — PRP164 —

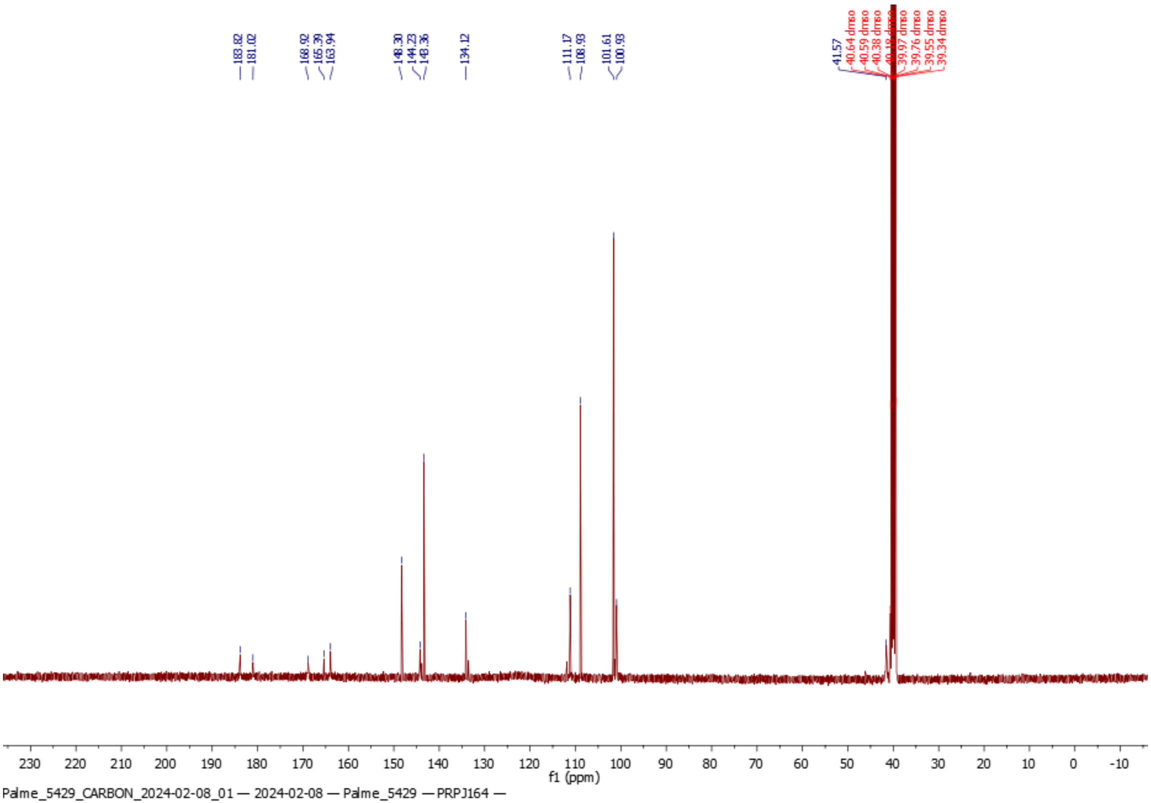

Palme_5429_CARBON_2024-02-08_01 — 2024-02-08 — Palme_5429 — PRPJ164 —

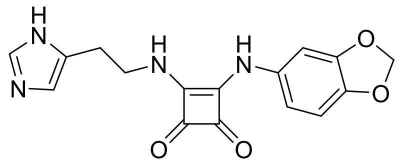

PRP015

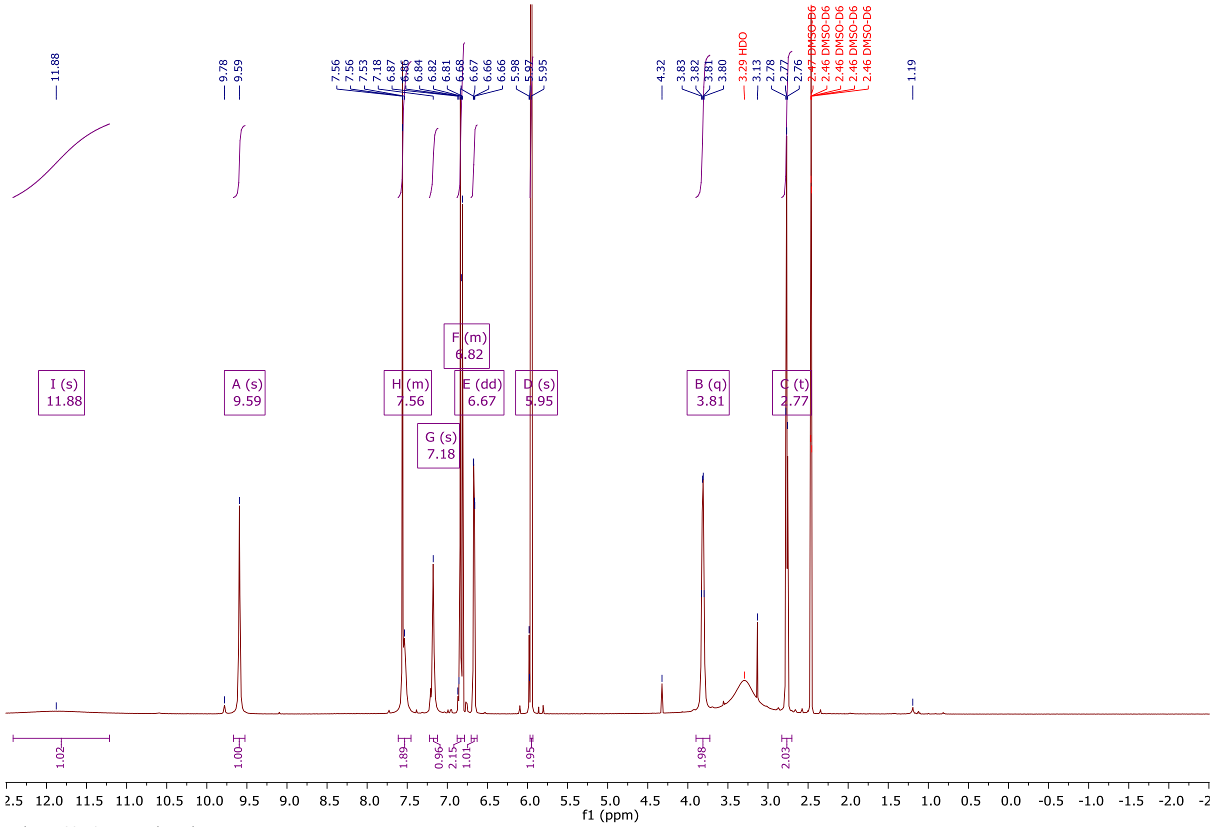

Palme_1095-97 — single_pulse

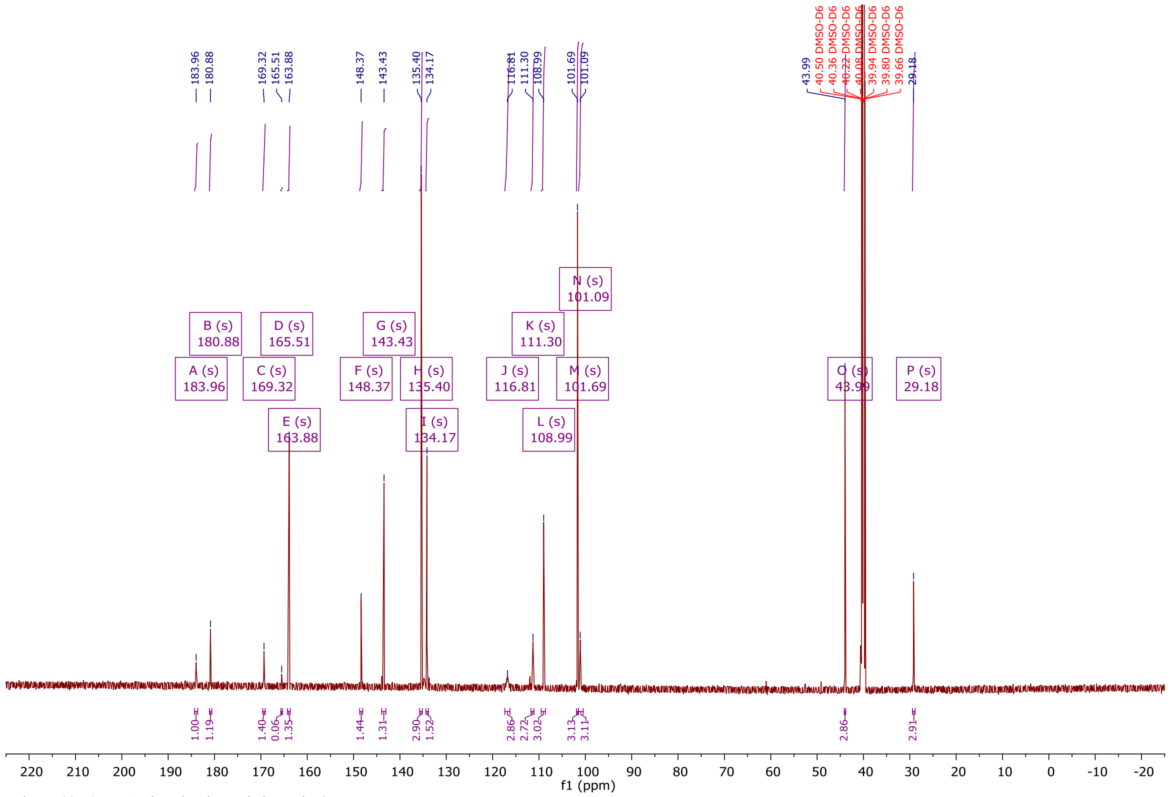

Palme_1095-97 — single pulse decoupled gated NOE

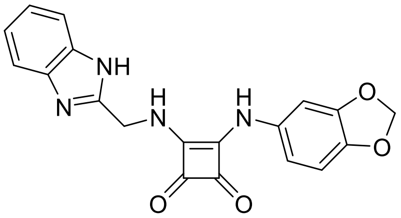

PRP016

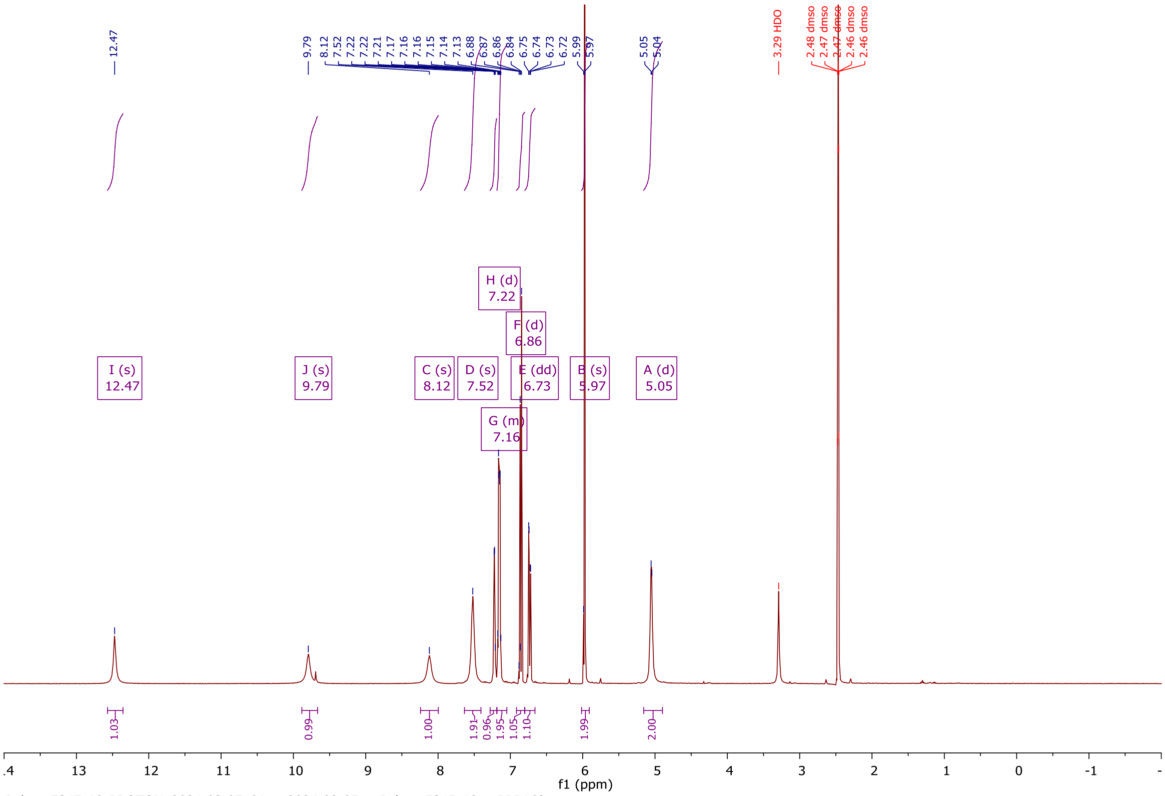

Palme_5345-46_PROTON_2024-02-05_01 — 2024-02-05 — Palme_5345-46 — PRP168 —

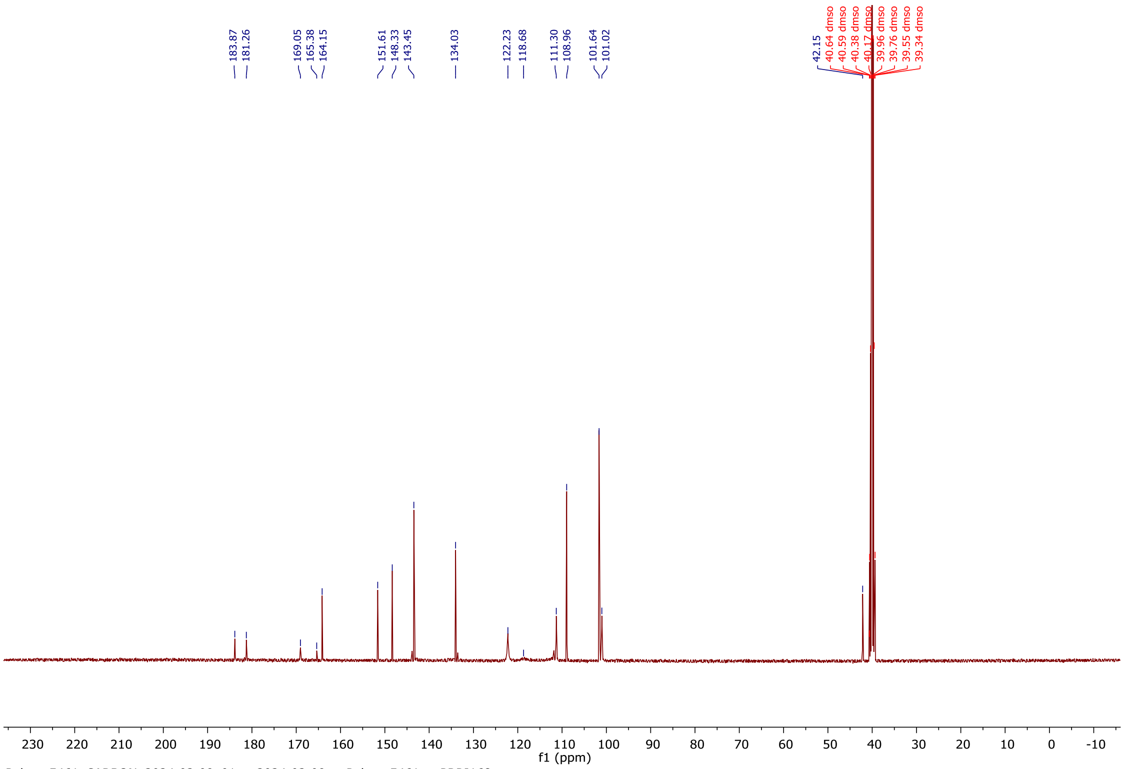

Palme_5461_CARBON_2024-02-09_01 — 2024-02-09 — Palme_5461 — PRPJ168 —

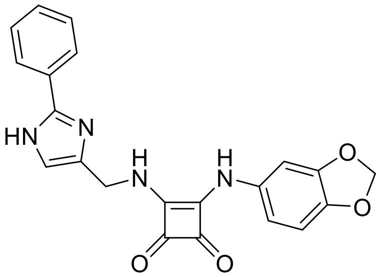

PRP017

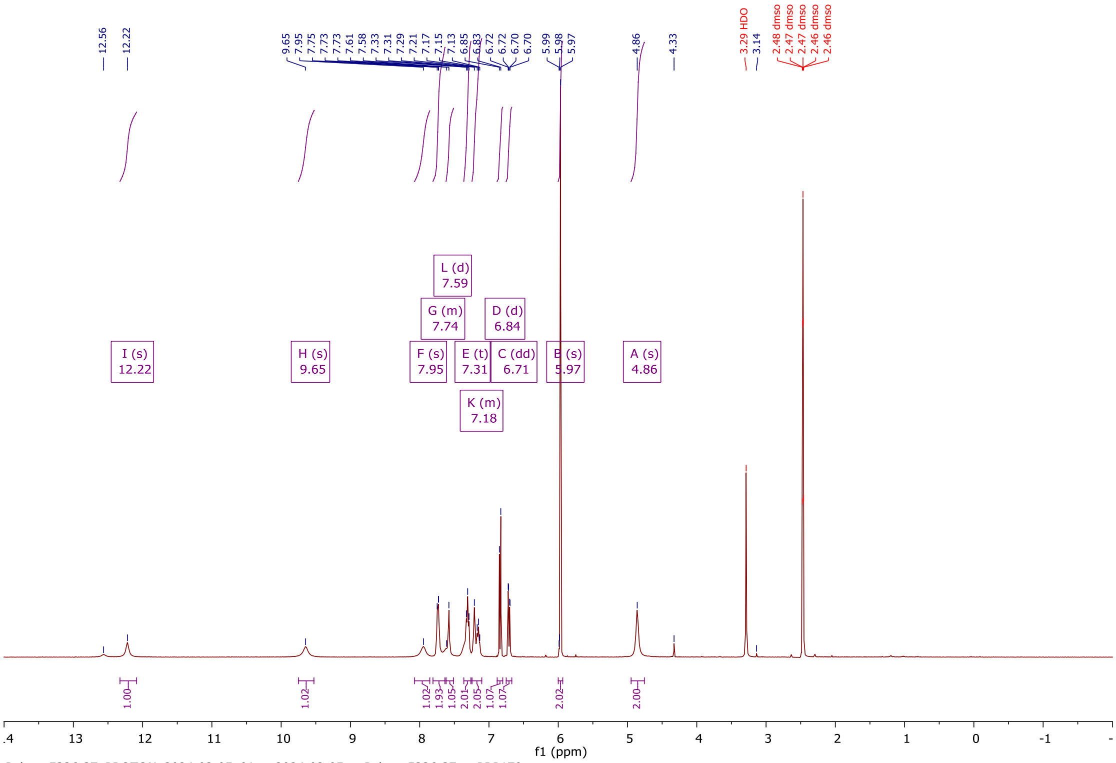

Palme_5336-37_PROTON_2024-02-05_01 — 2024-02-05 — Palme_5336-37 — PRP179 —

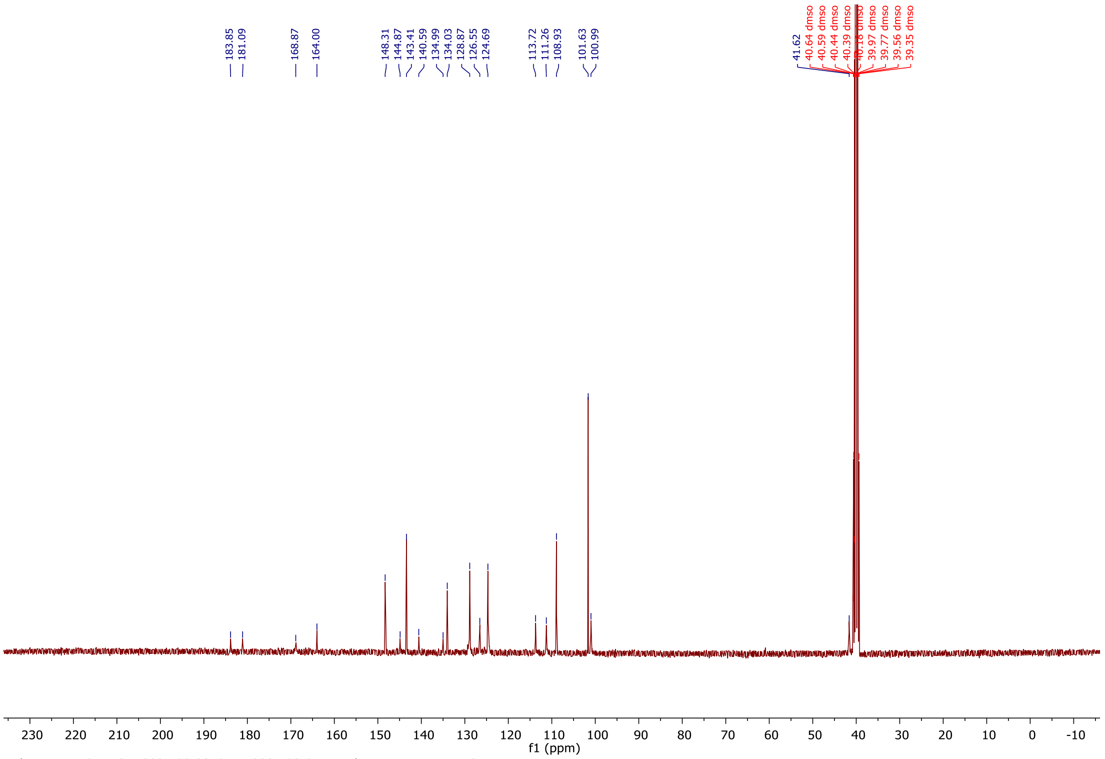

Palme_5414_CARBON_2024-02-08_01 — 2024-02-07 — Palme_5414 — PRP179 —

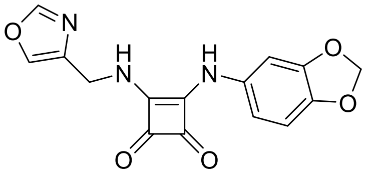

PRP018

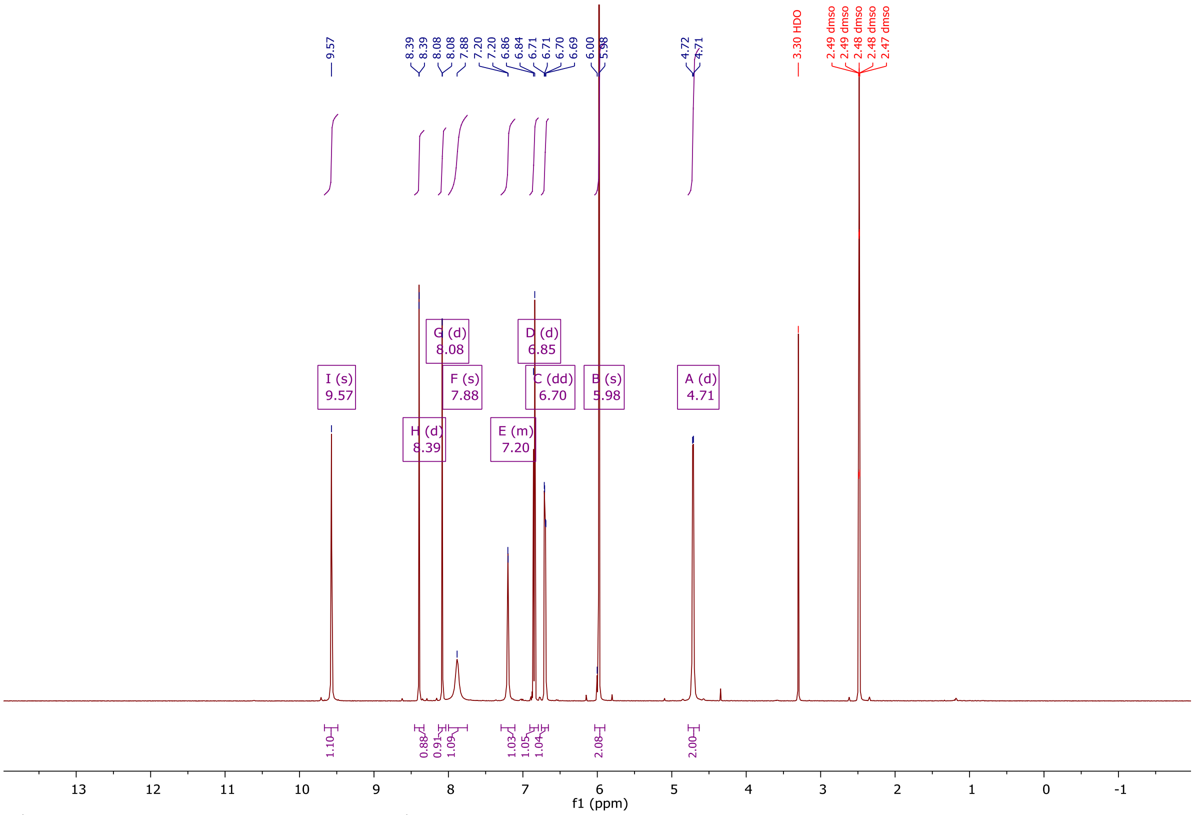

Palme_6432-33_PROTON_2023-12-12_01 — 2023-12-12 — Palme_6432-33 — PRP 178 —

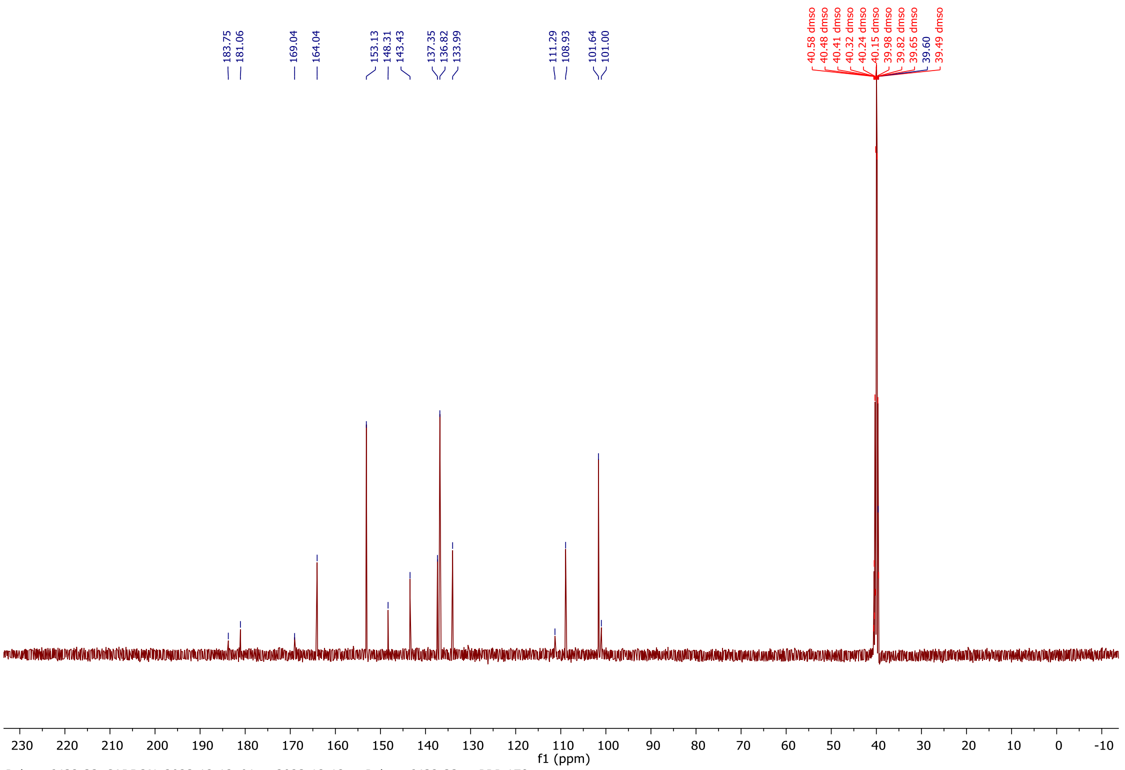

Palme_6432-33_CARBON_2023-12-12_01 — 2023-12-12 — Palme_6432-33 — PRP 178 —

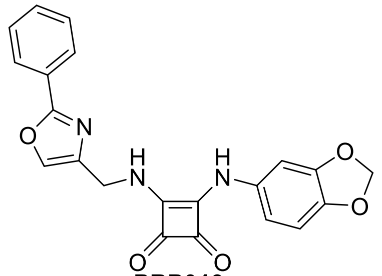

PRP019

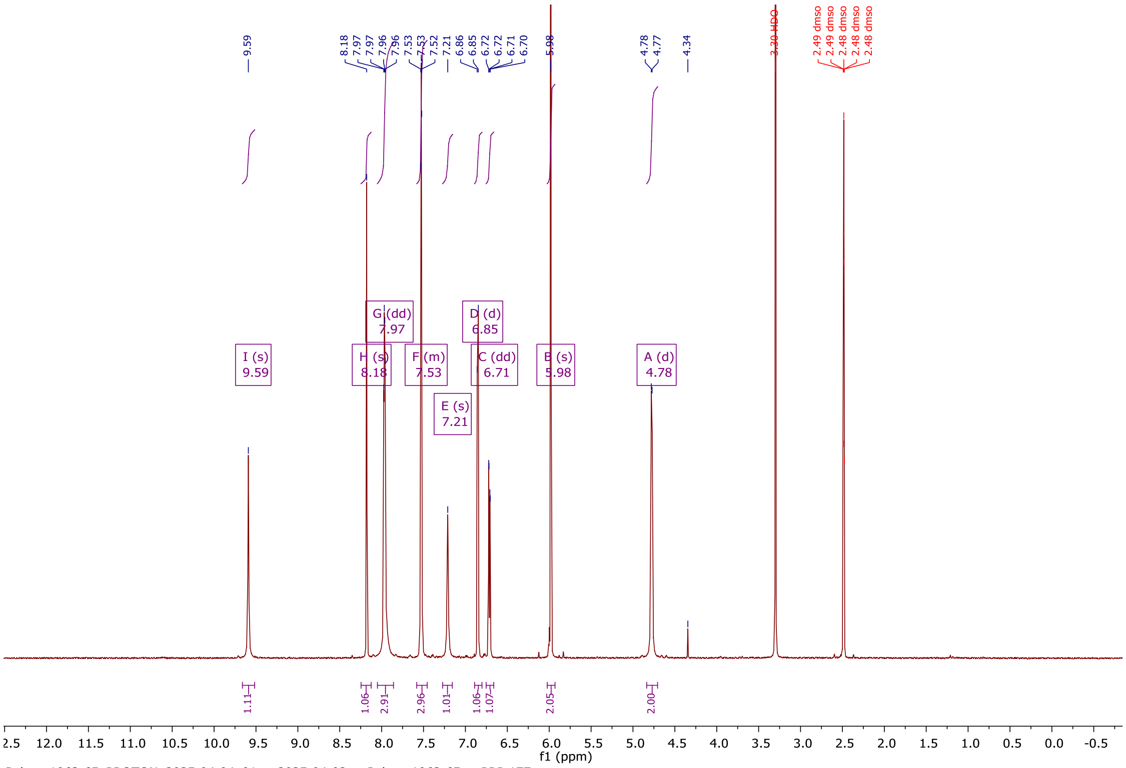

Palme_1063-65_PROTON_2025-04-04_01 — 2025-04-02 — Palme_1063-65 — PRP 177 —

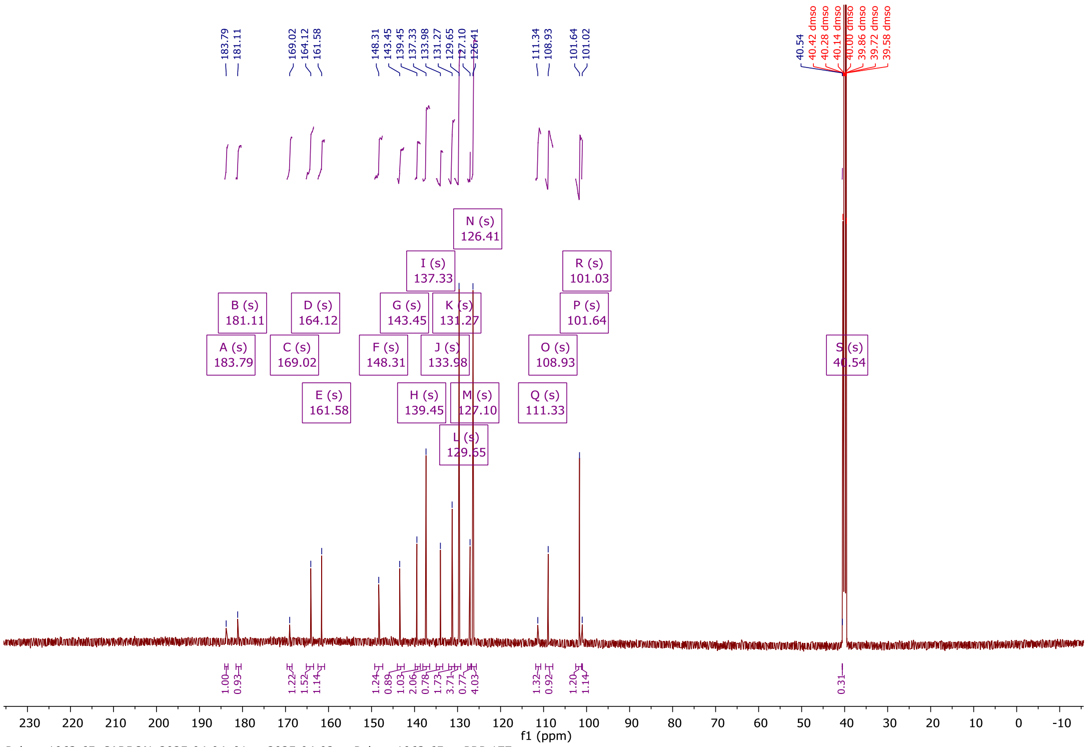

Palme_1063-65_CARBON_2025-04-04_01 — 2025-04-02 — Palme_1063-65 — PRP 177 —

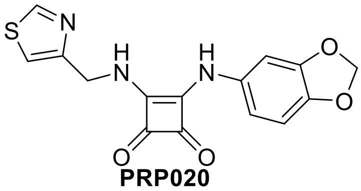

PRP020

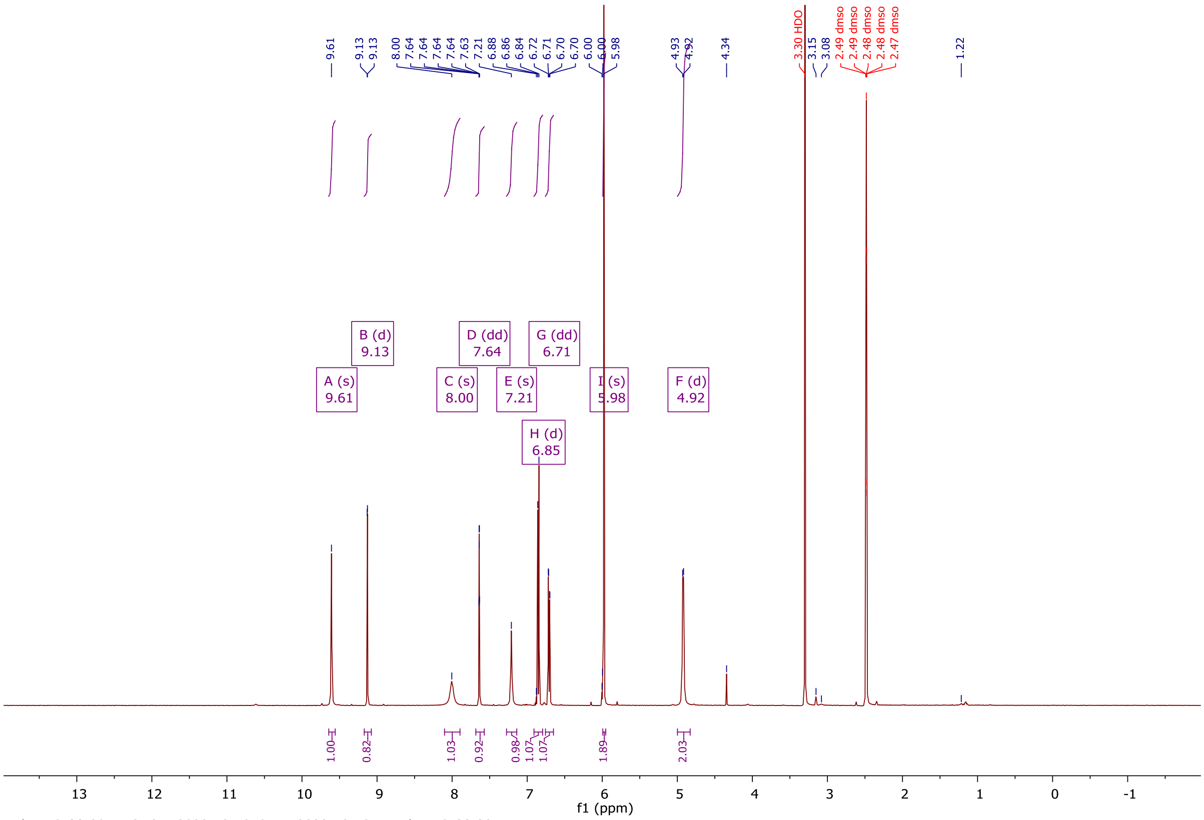

Palme_6438-39_PROTON_2023-12-12_01 — 2023-12-12 — Palme_6438-39 — PRP 175 —

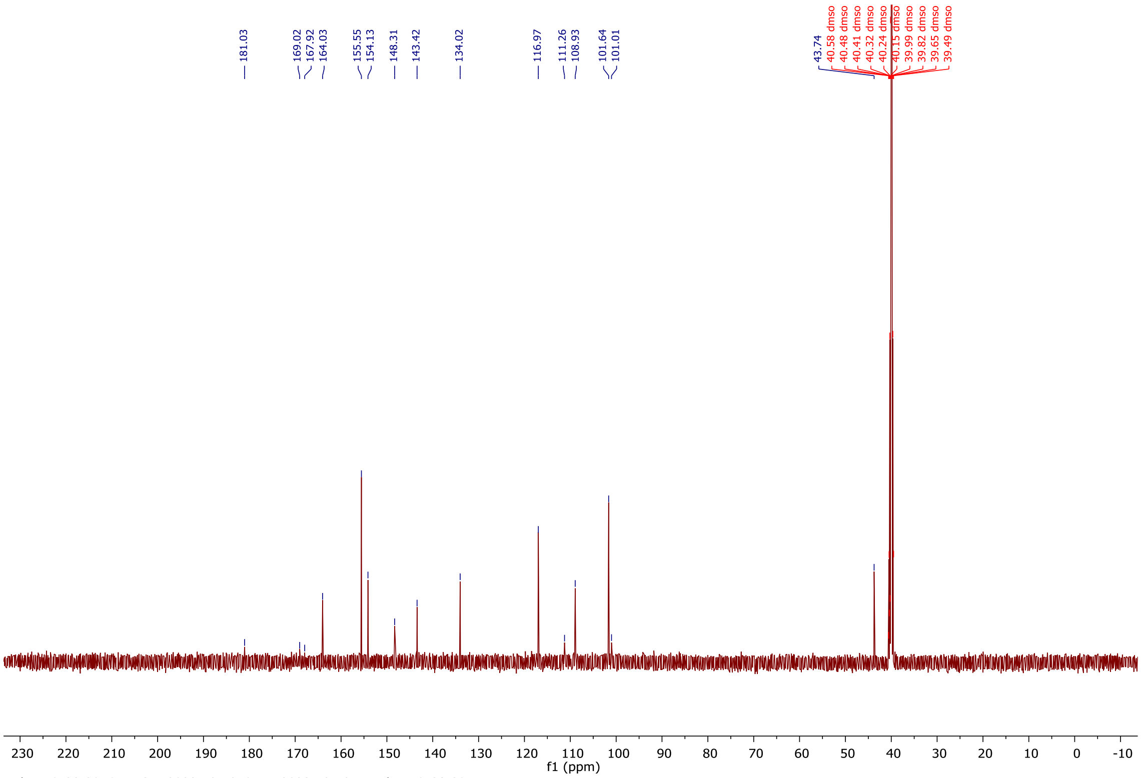

Palme_6438-39_CARBON_2023-12-12_01 — 2023-12-12 — Palme_6438-39 — PRP 175 —

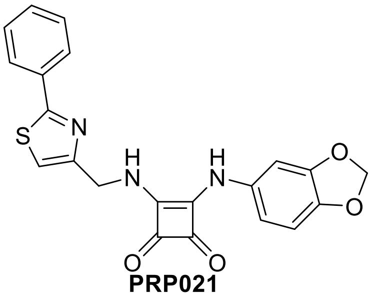

PRP021

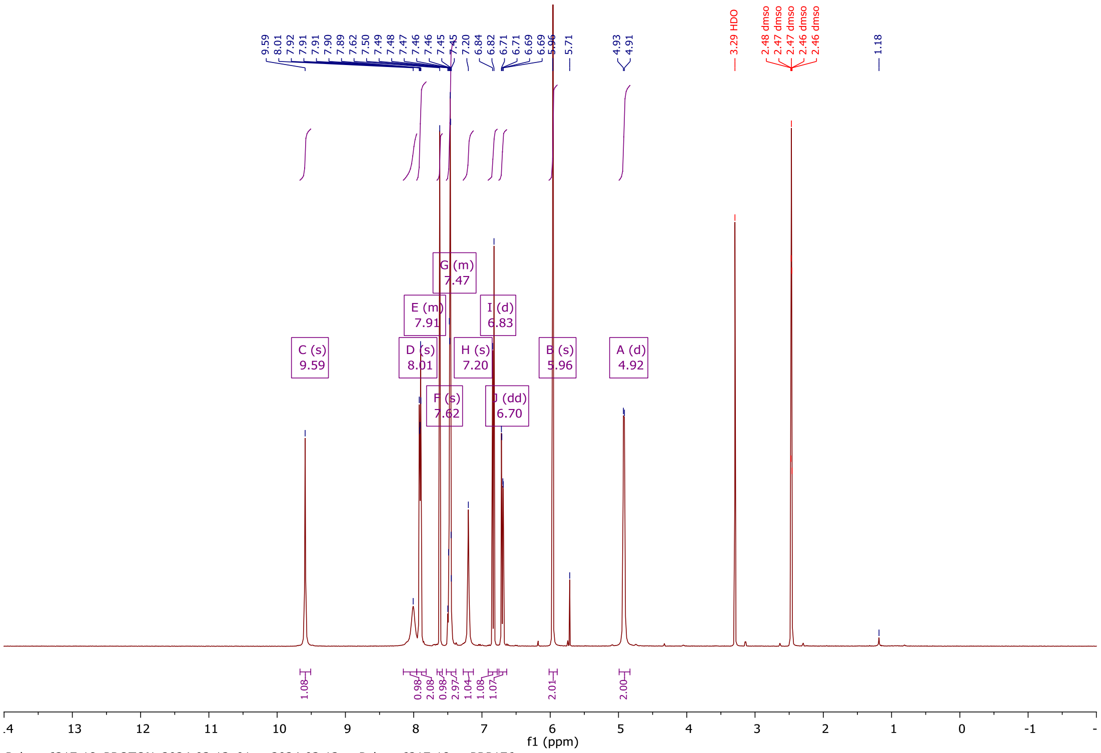

Palme_6217-18_PROTON_2024-03-13_01 — 2024-03-13 — Palme_6217-18 — PRP176 —

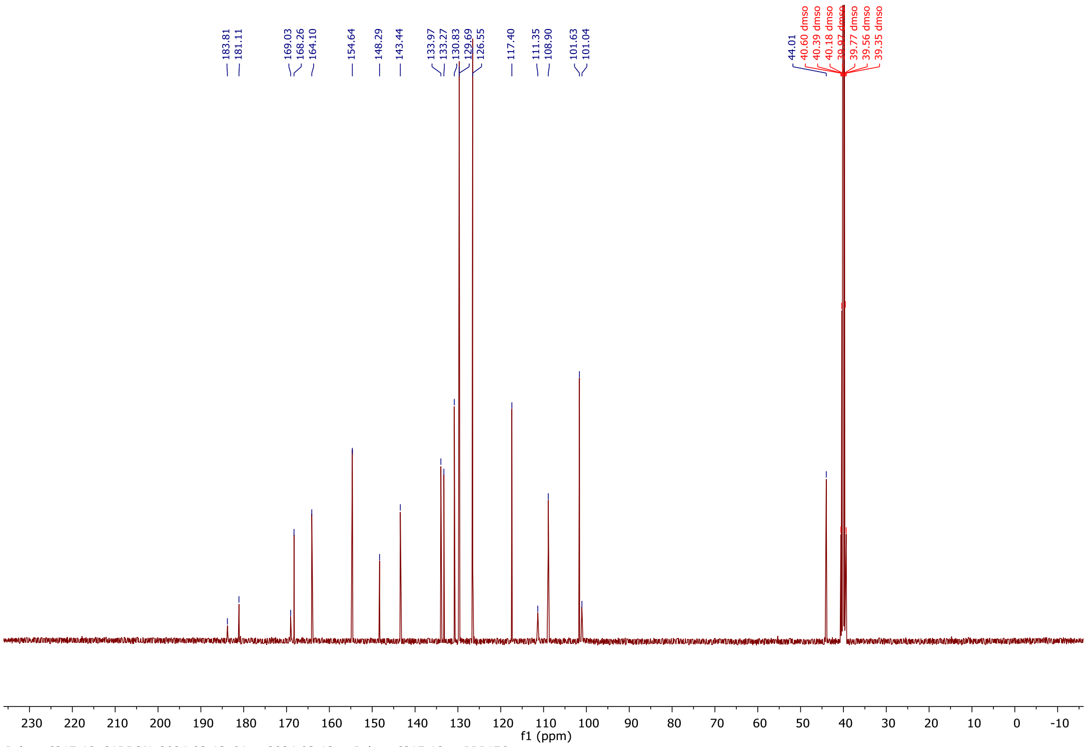

Palme_6217-18_CARBON_2024-03-13_01 — 2024-03-13 — Palme_6217-18 — PRP176 —

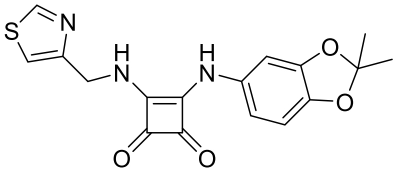

PRP022

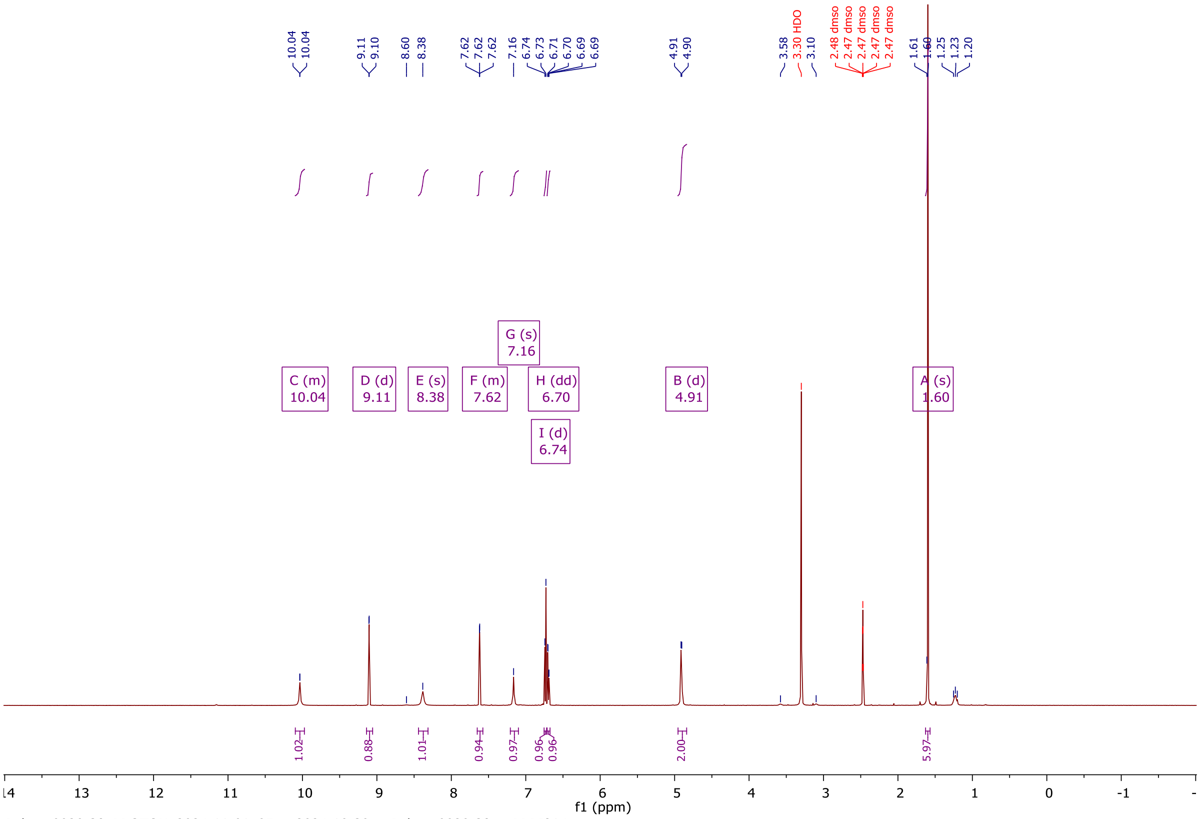

Palme_9920-38_PROTON_2024-11-01_05 — 2024-10-30 — Palme_9920-38 — PRPJ214 —

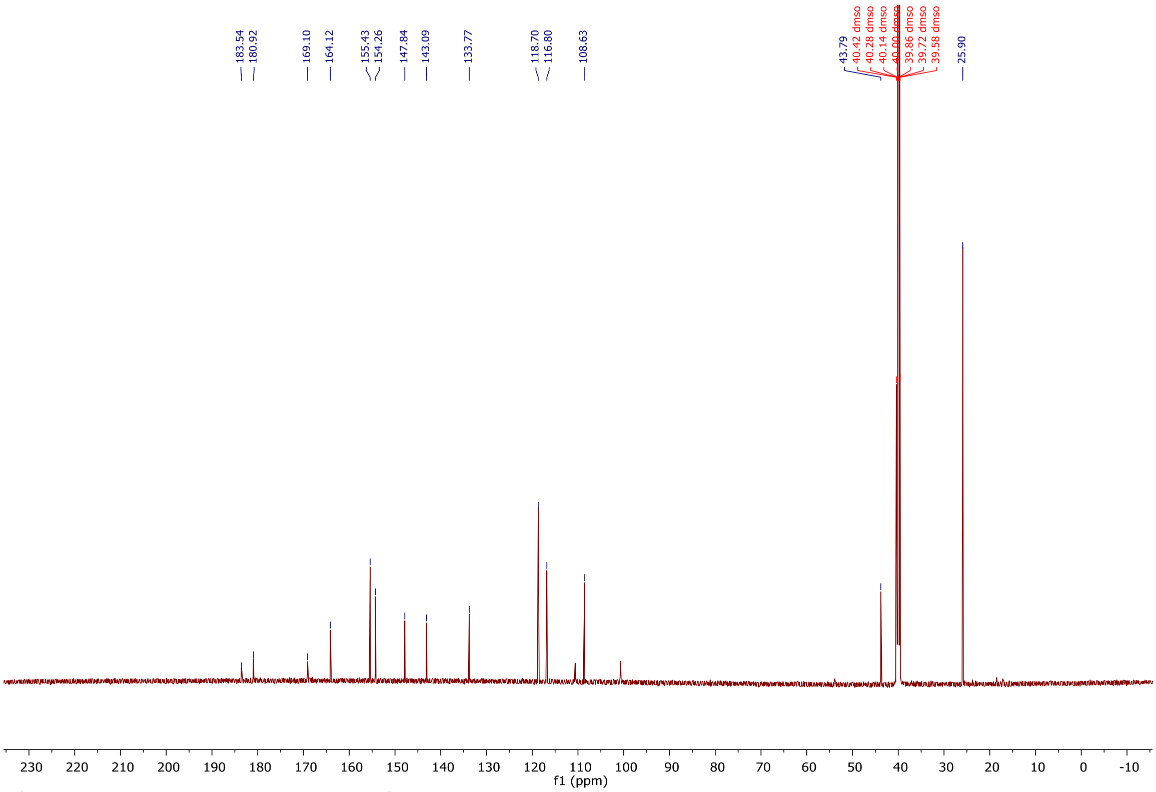

Palme_PRP214_CARBON_2024-11-10_01 — 2024-11-08 — Palme_PRP214 —

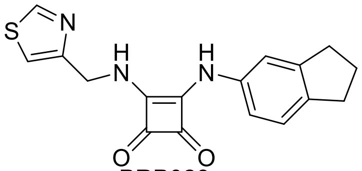

PRP023

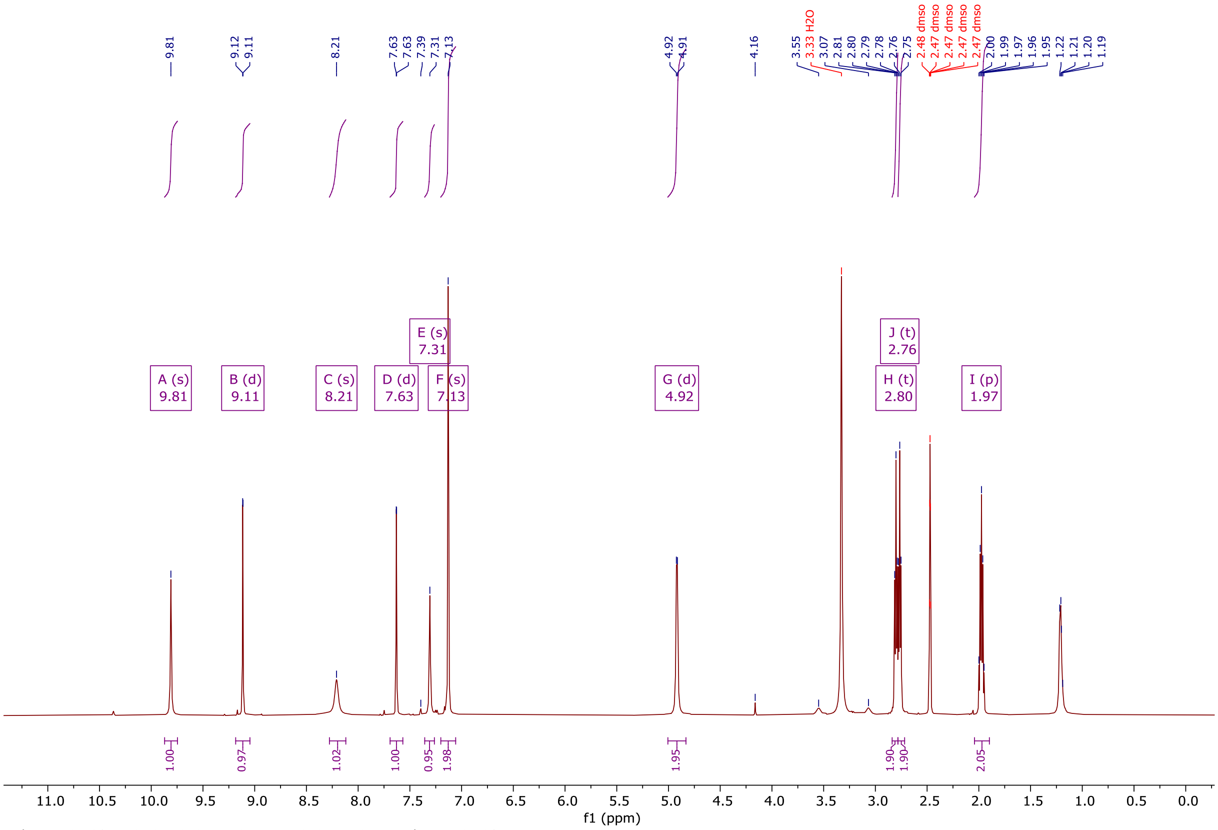

Palme_9920-38_PROTON_2024-10-30_01 — 2024-10-30 — Palme_9920-38 — PRPJ179 —

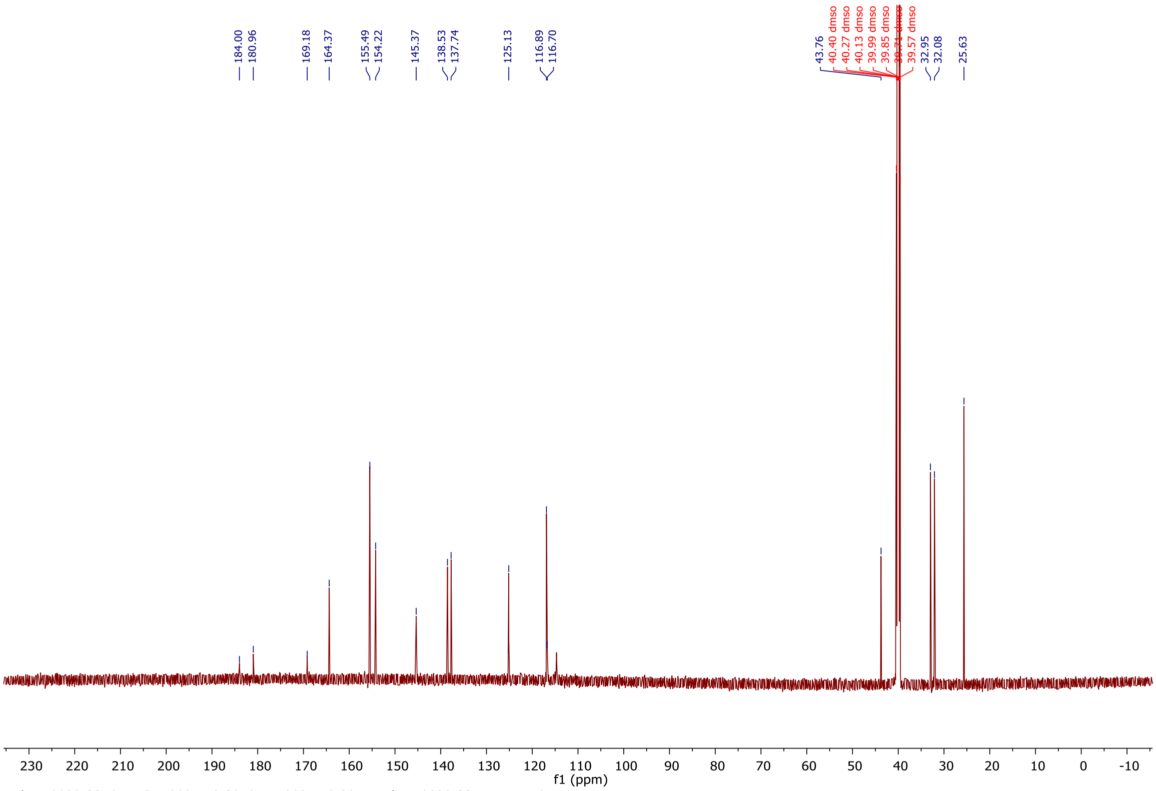

Palme_9920-38_CARBON_2024-10-30_01 — 2024-10-30 — Palme_9920-38 — PRPJ179 —

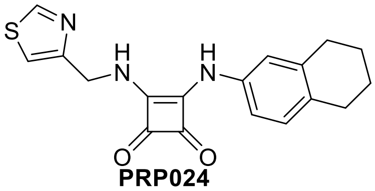

PRP024

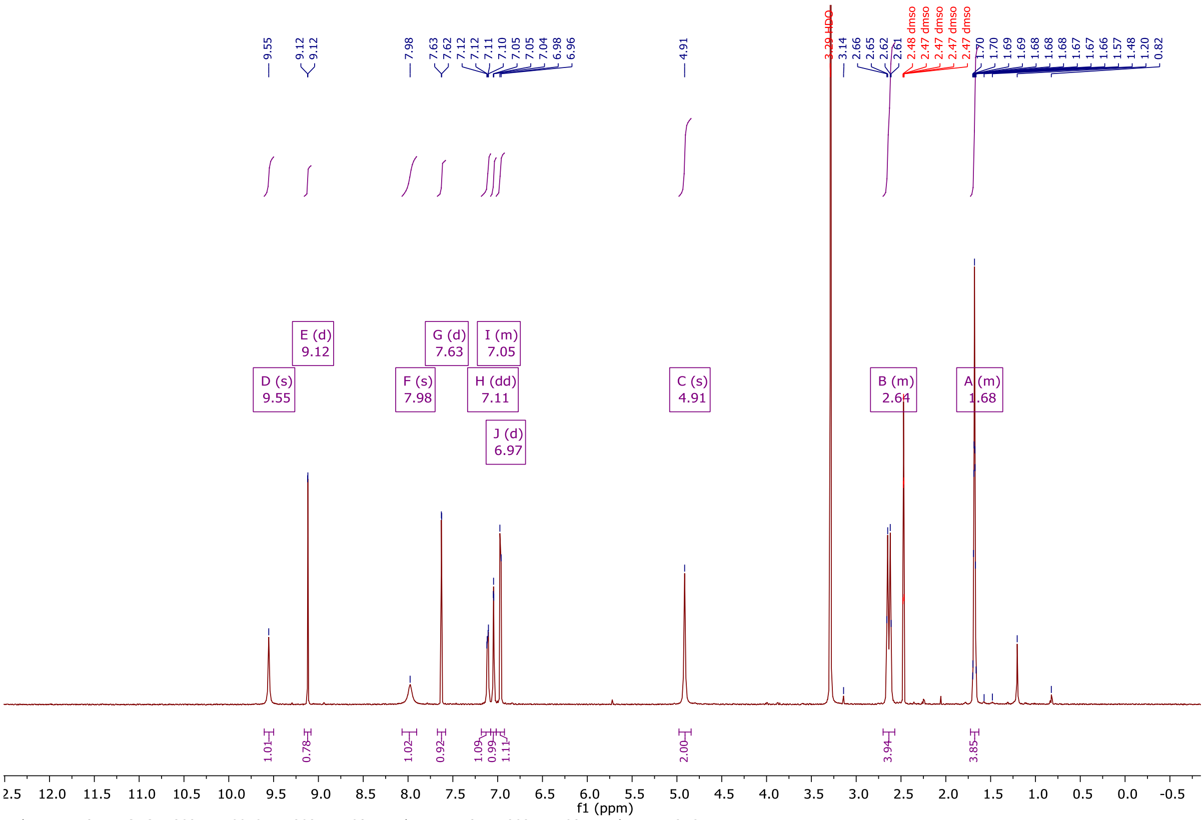

Palme_PRP181_PROTON_2024-11-09_01 — 2024-11-08 — Palme_PRP181 — 2024-11-08 — Palme_PRP216 —

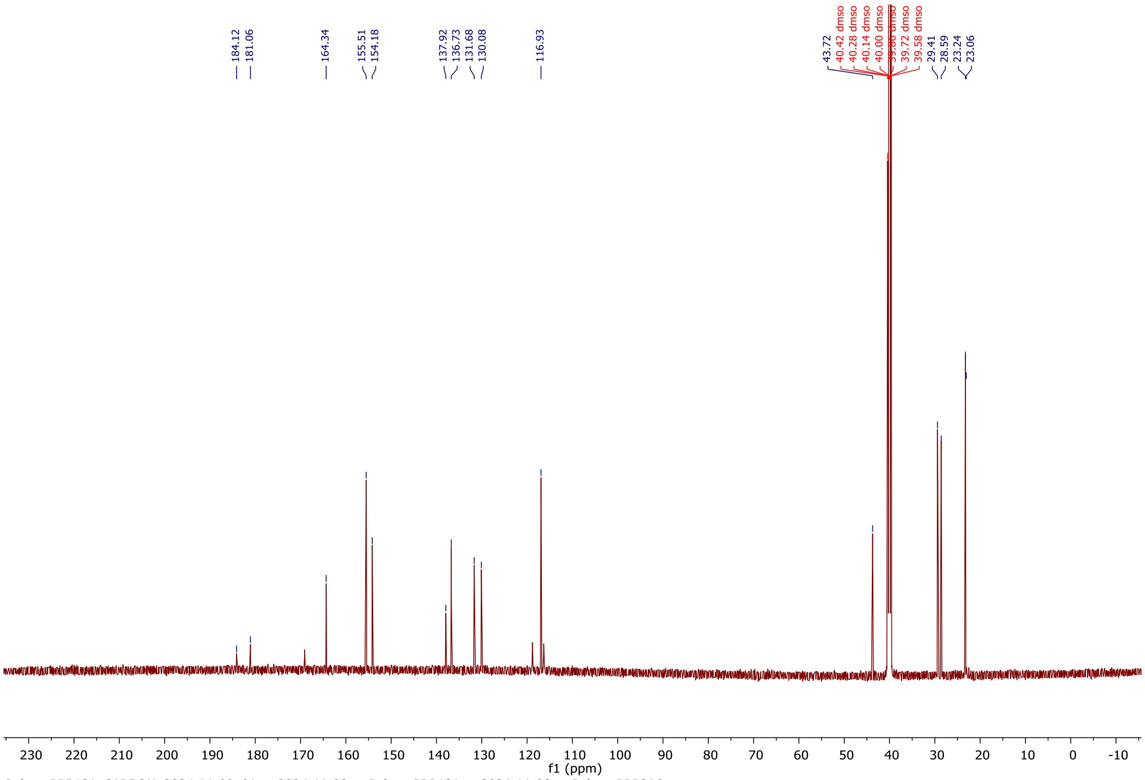

Palme_PRP181_CARBON_2024-11-09_01 — 2024-11-08 — Palme_PRP181 — 2024-11-08 — Palme_PRP216 —

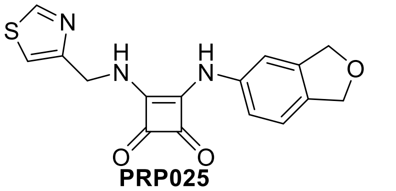

PRP025

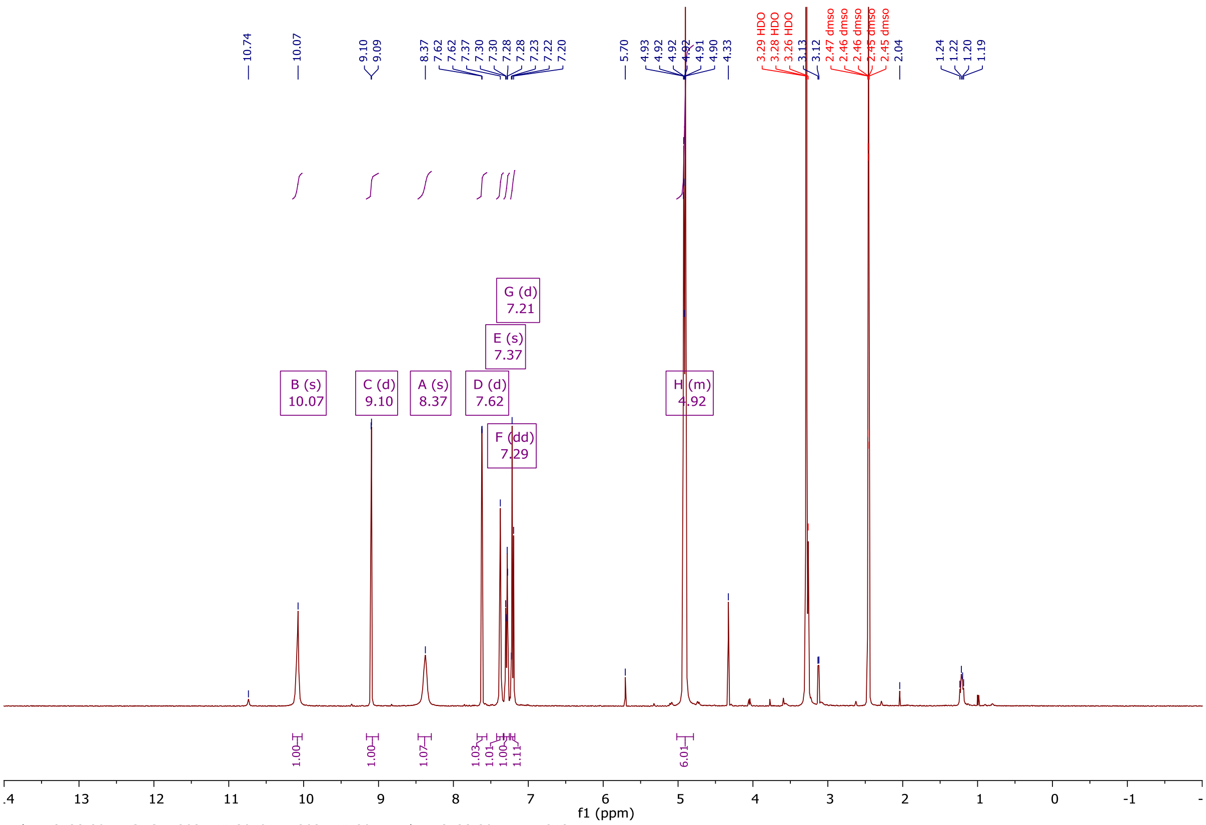

Palme_8528-29_PROTON_2024-11-30_01 — 2024-11-29 — Palme_8528-29 — PRP 218 —

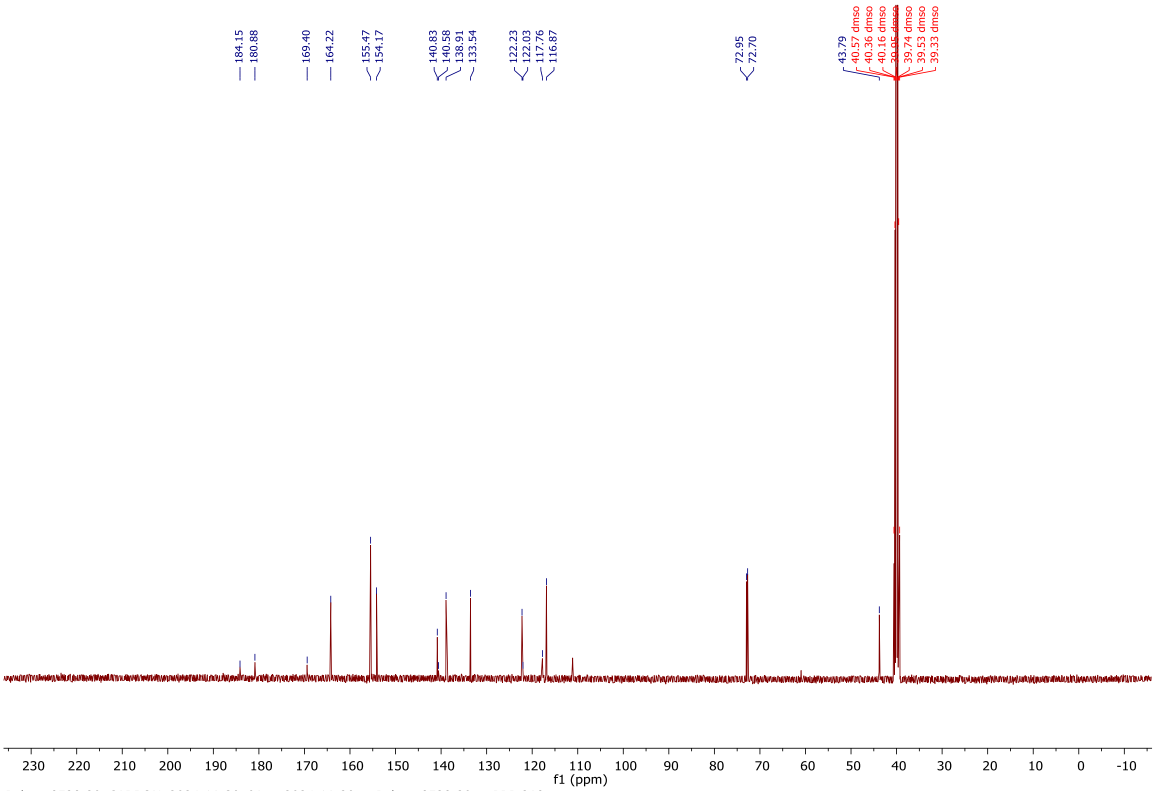

Palme_8528-29_CARBON_2024-11-30_01 — 2024-11-29 — Palme_8528-29 — PRP 218 —

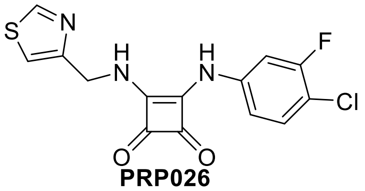

PRP026

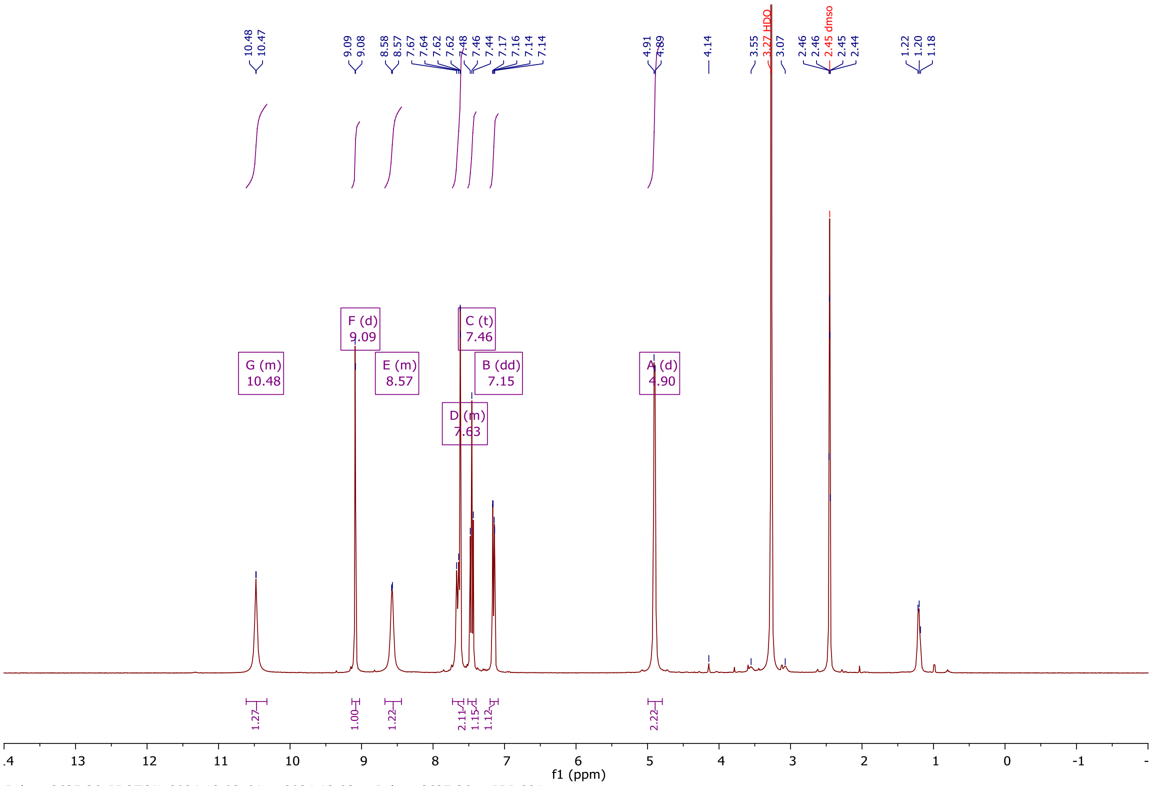

Palme_8635-36_PROTON_2024-12-03_01 — 2024-12-03 — Palme_8635-36 — PRP 221 —

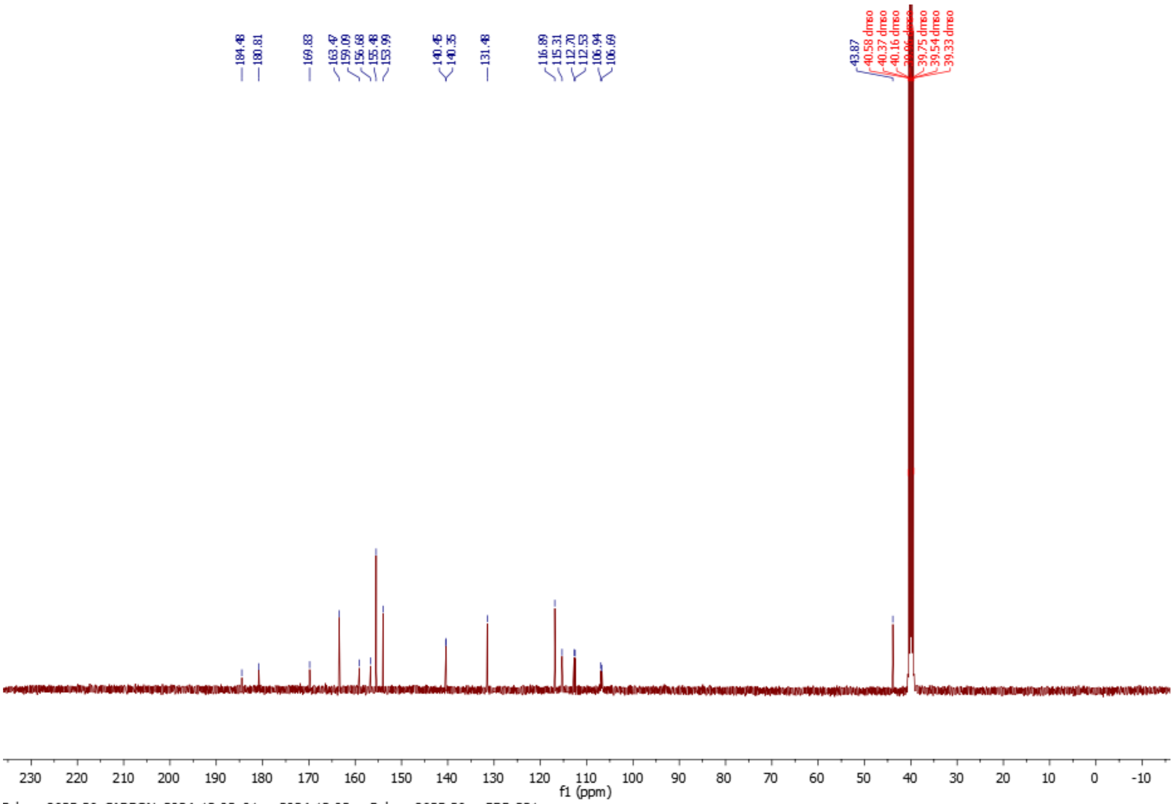

Palme_8635-36_CARBON_2024-12-03_01 — 2024-12-03 — Palme_8635-36 — PRP 221 —

Unknown NMR (564 MHz, DMSO-d_6) δ -114.02.

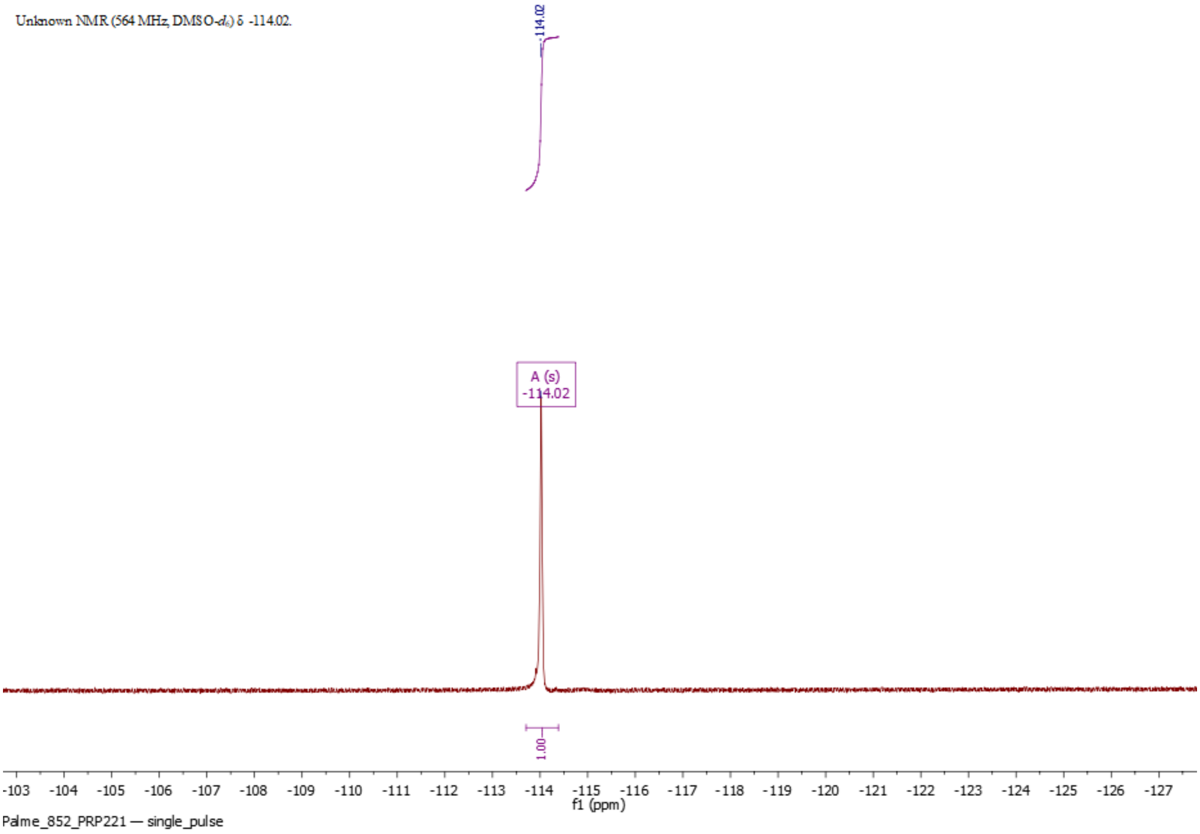

Palme_852_PRP221 — single_pulse

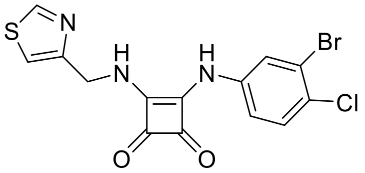

PRP027

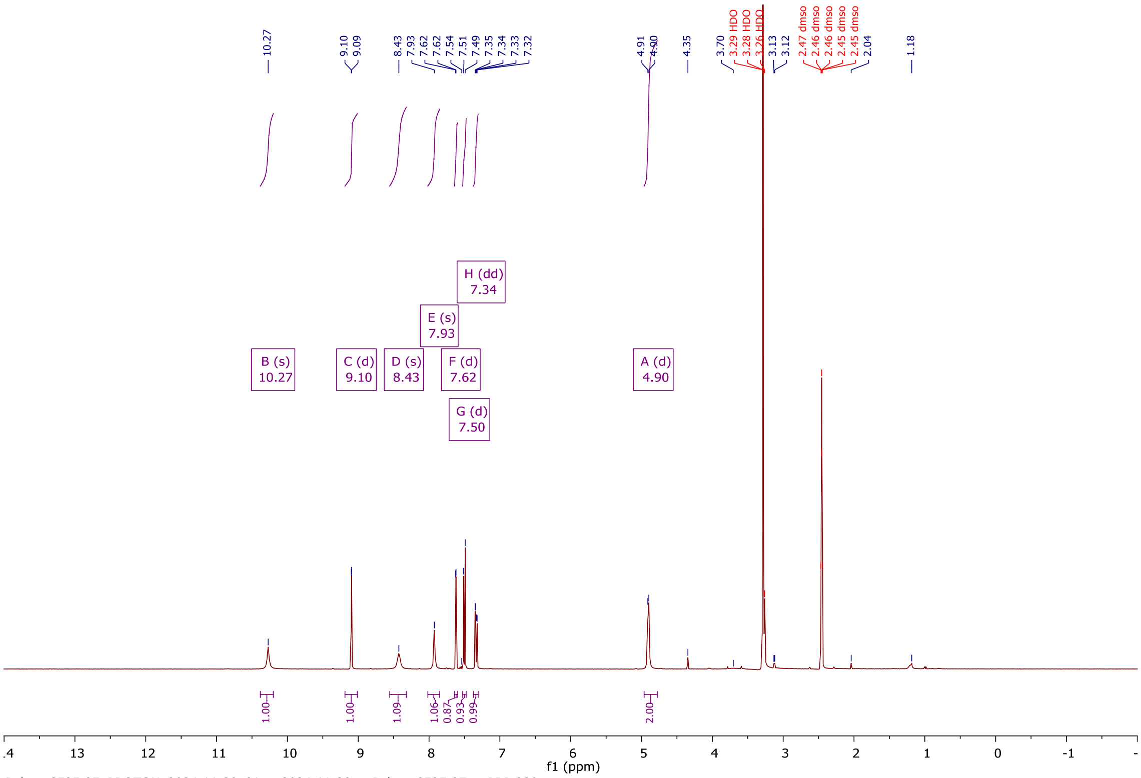

Palme_8525-27_PROTON_2024-11-29_01 — 2024-11-29 — Palme_8525-27 — PRP 220 —

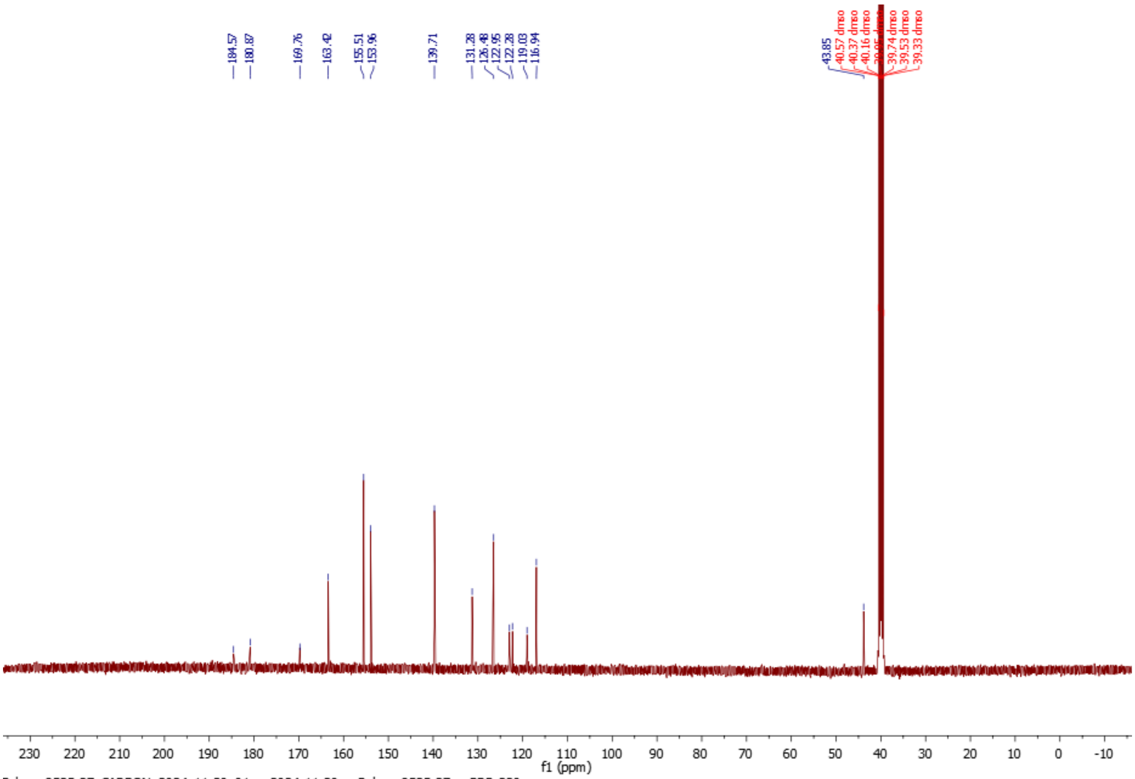

Palme_8525-27_CARBON_2024-11-30_01 — 2024-11-29 — Palme_8525-27 — PRP 220 —

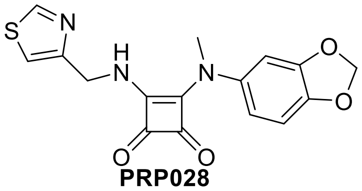

PRP028

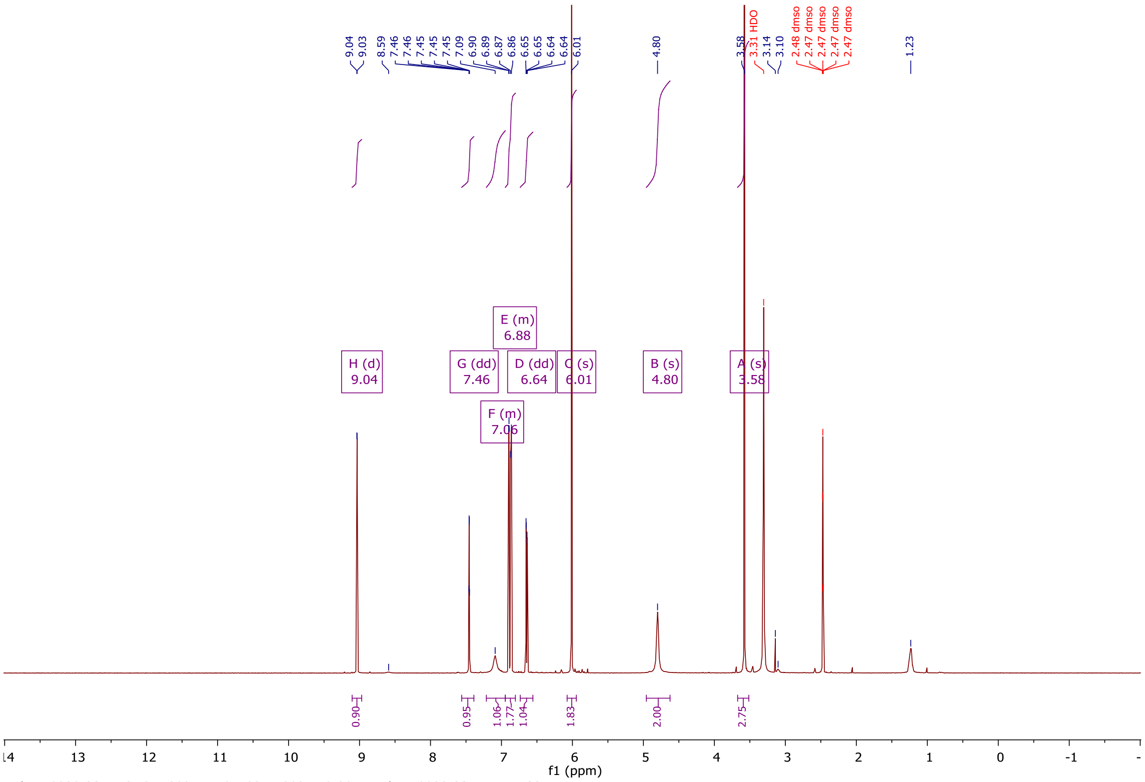

Palme_9920-38_PROTON_2024-11-01_03 — 2024-10-30 — Palme_9920-38 — PRPJ183 —

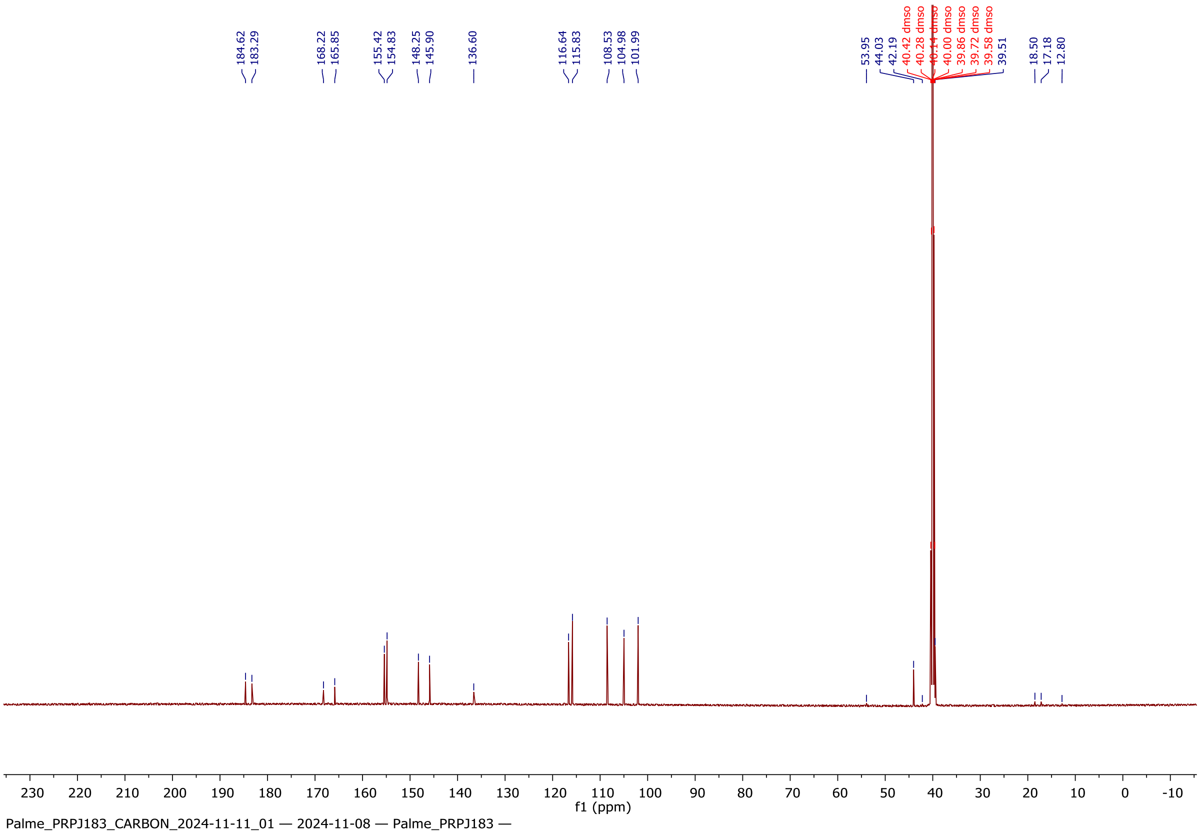

Palme_PRPJ183_CARBON_2024-11-11_01 — 2024-11-08 — Palme_PRPJ183 —

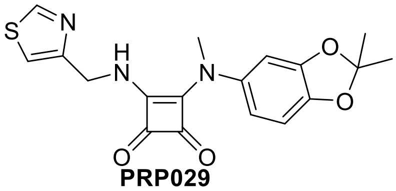

PRP029

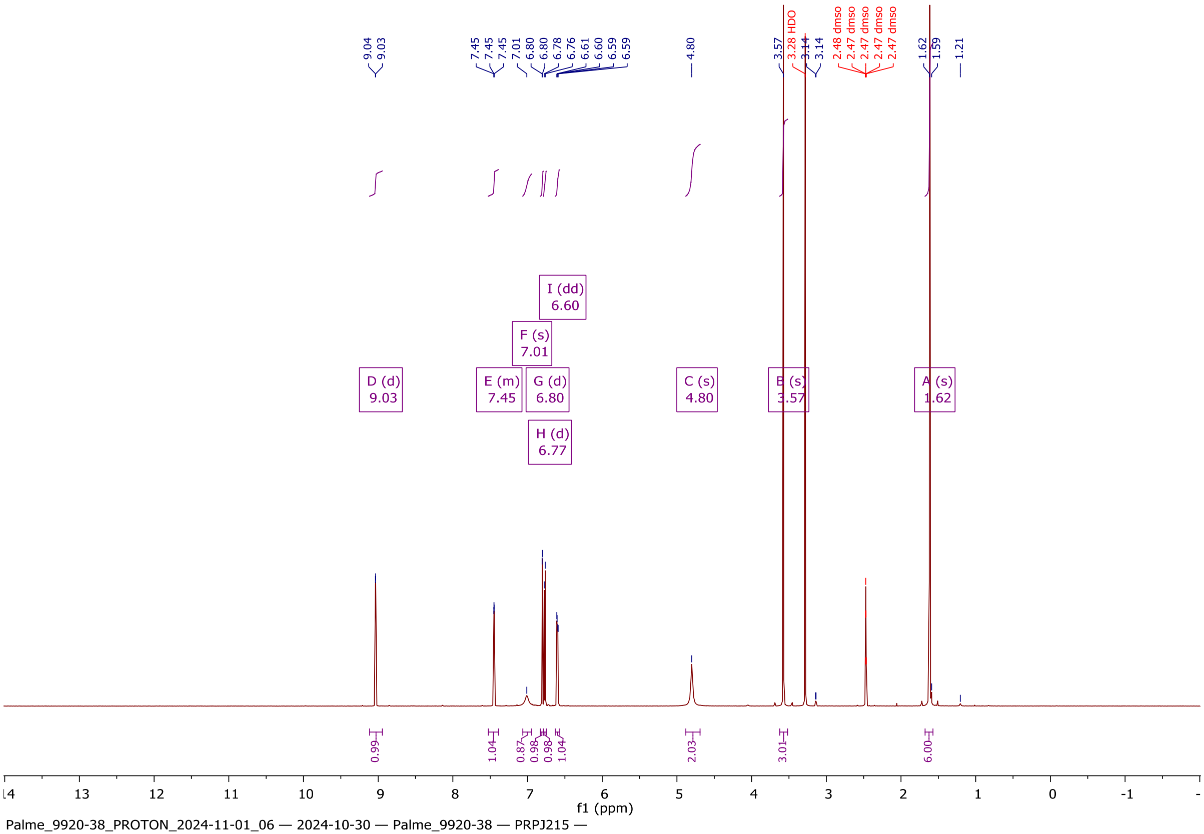

Palme_9920-38_PROTON_2024-11-01_06 — 2024-10-30 — Palme_9920-38 — PRPJ215 —

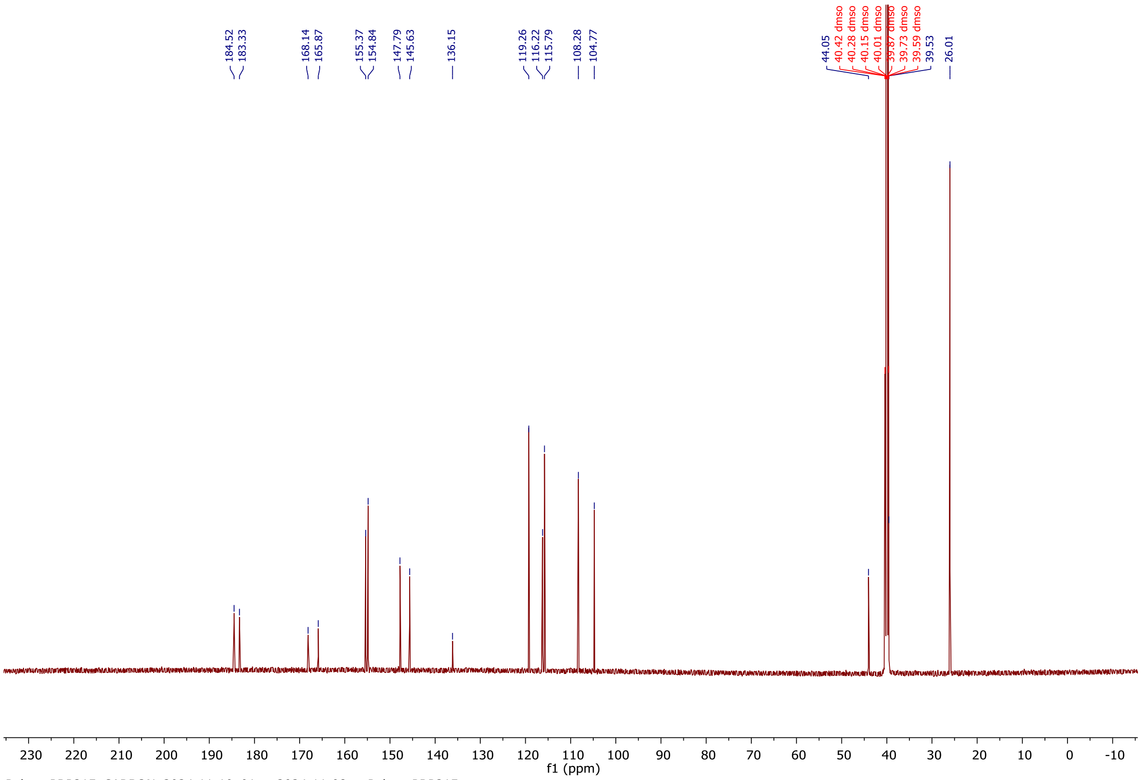

Palme_PRP215_CARBON_2024-11-10_01 — 2024-11-08 — Palme_PRP215 —

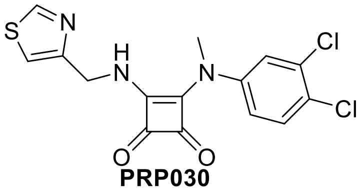

PRP030

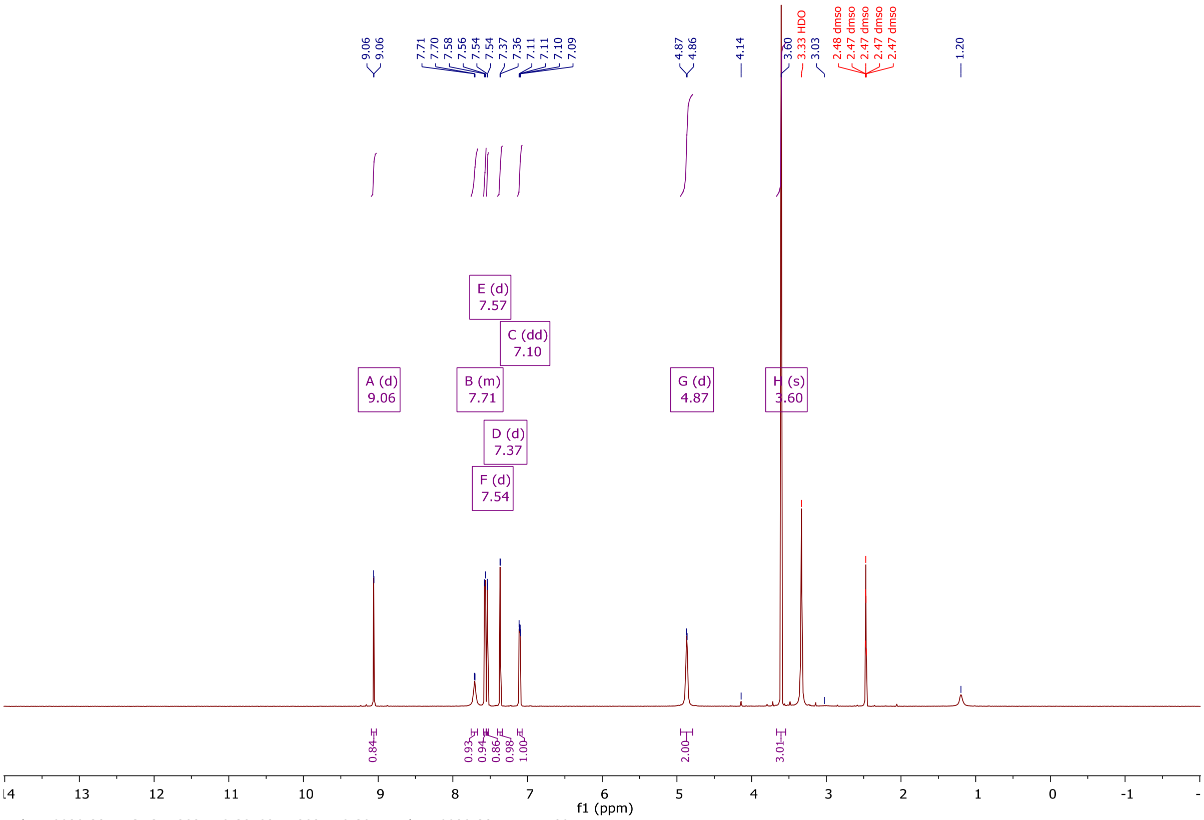

Palme_9920-38_PROTON_2024-10-30_02 — 2024-10-30 — Palme_9920-38 — PRPJ180 —

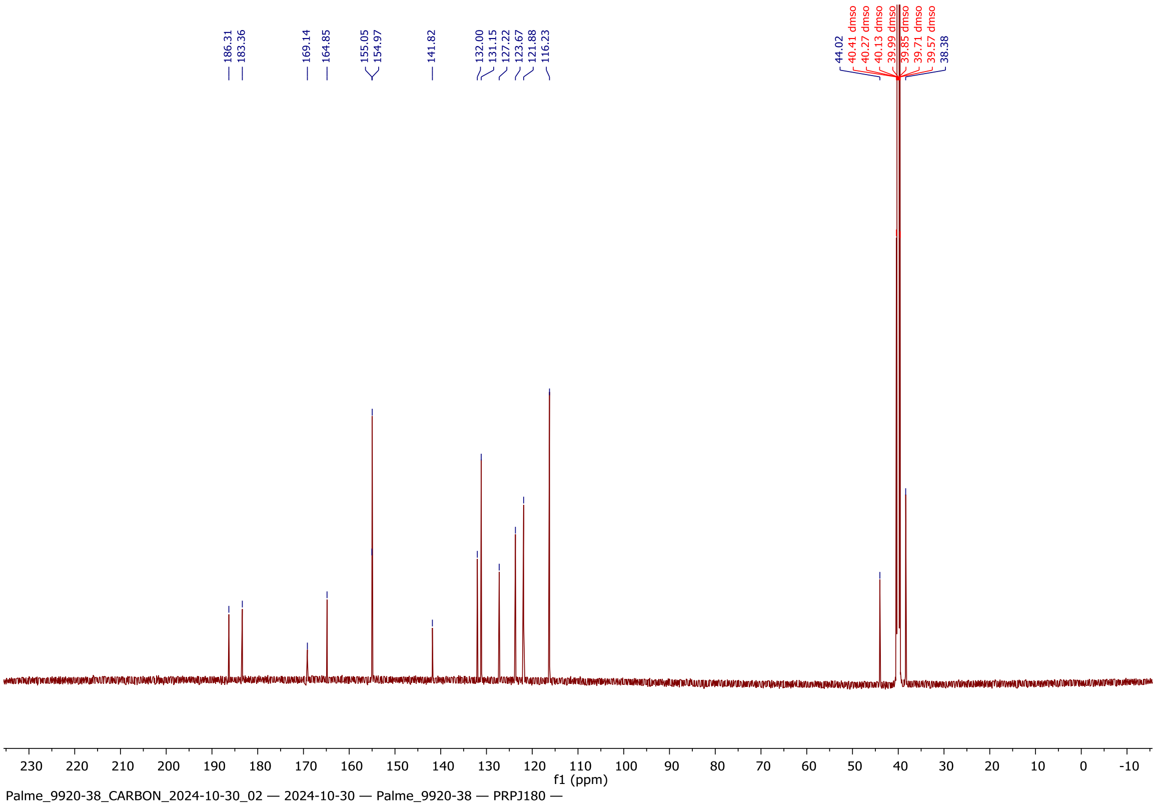

Palme_9920-38_CARBON_2024-10-30_02 — 2024-10-30 — Palme_9920-38 — PRPJ180 —

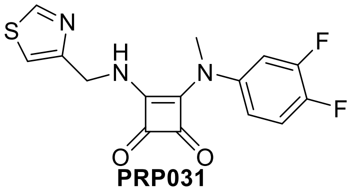

PRP031

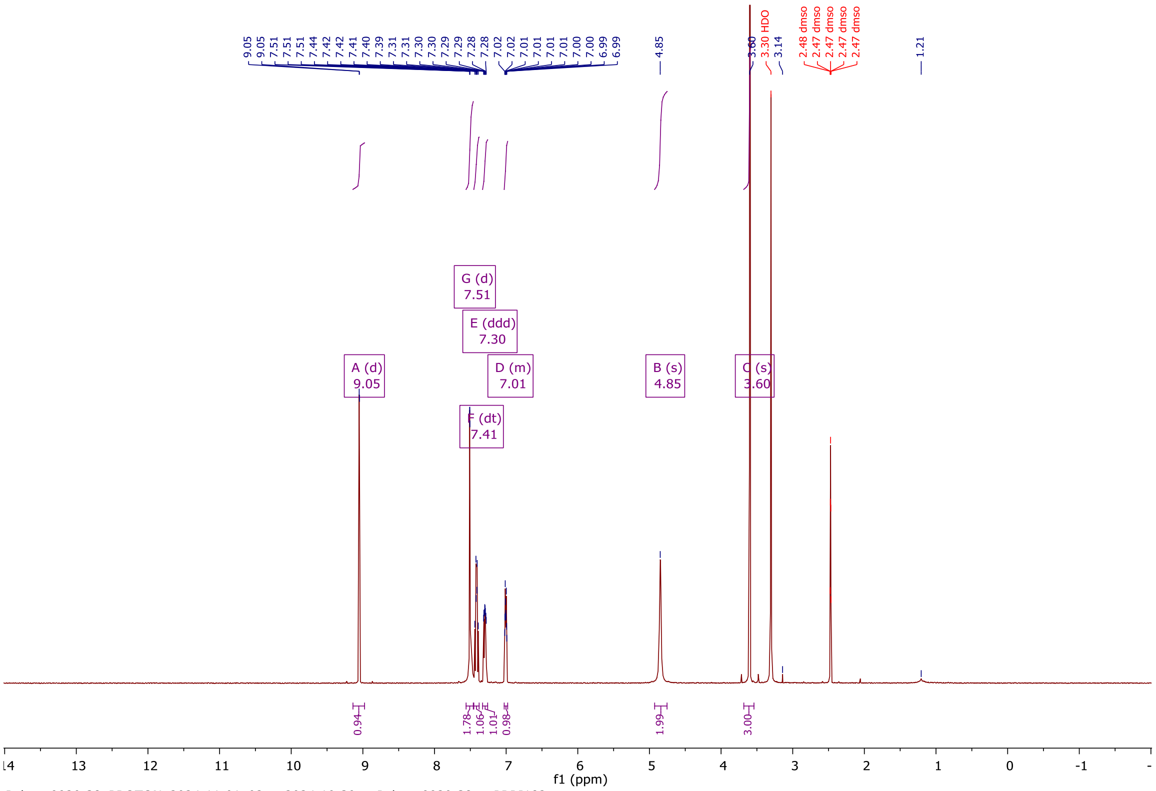

Palme_9920-38_PROTON_2024-11-01_02 — 2024-10-30 — Palme_9920-38 — PRPJ182 —

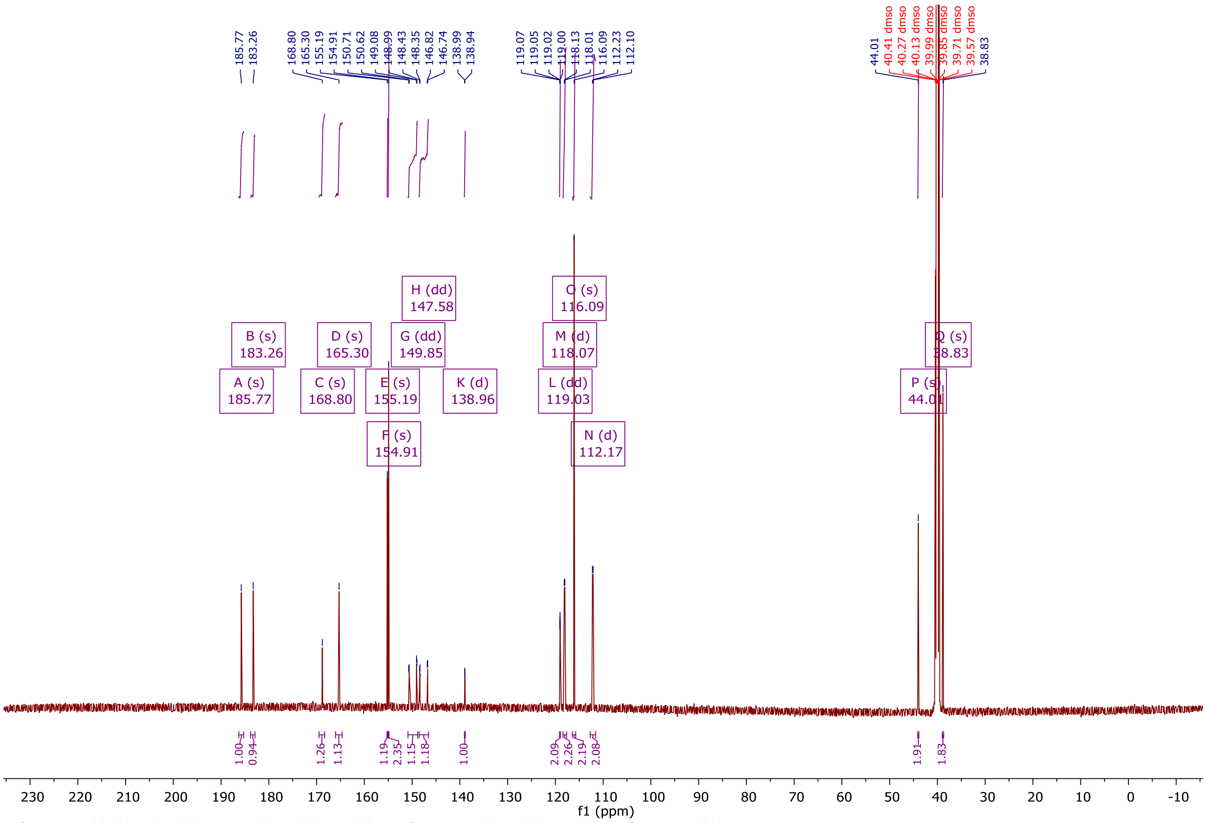

Palme_PRPJ182_CARBON_2024-11-11_01 — 2024-11-08 — Palme_PRPJ182 — 2024-11-08 — Palme_PRPJ183 —

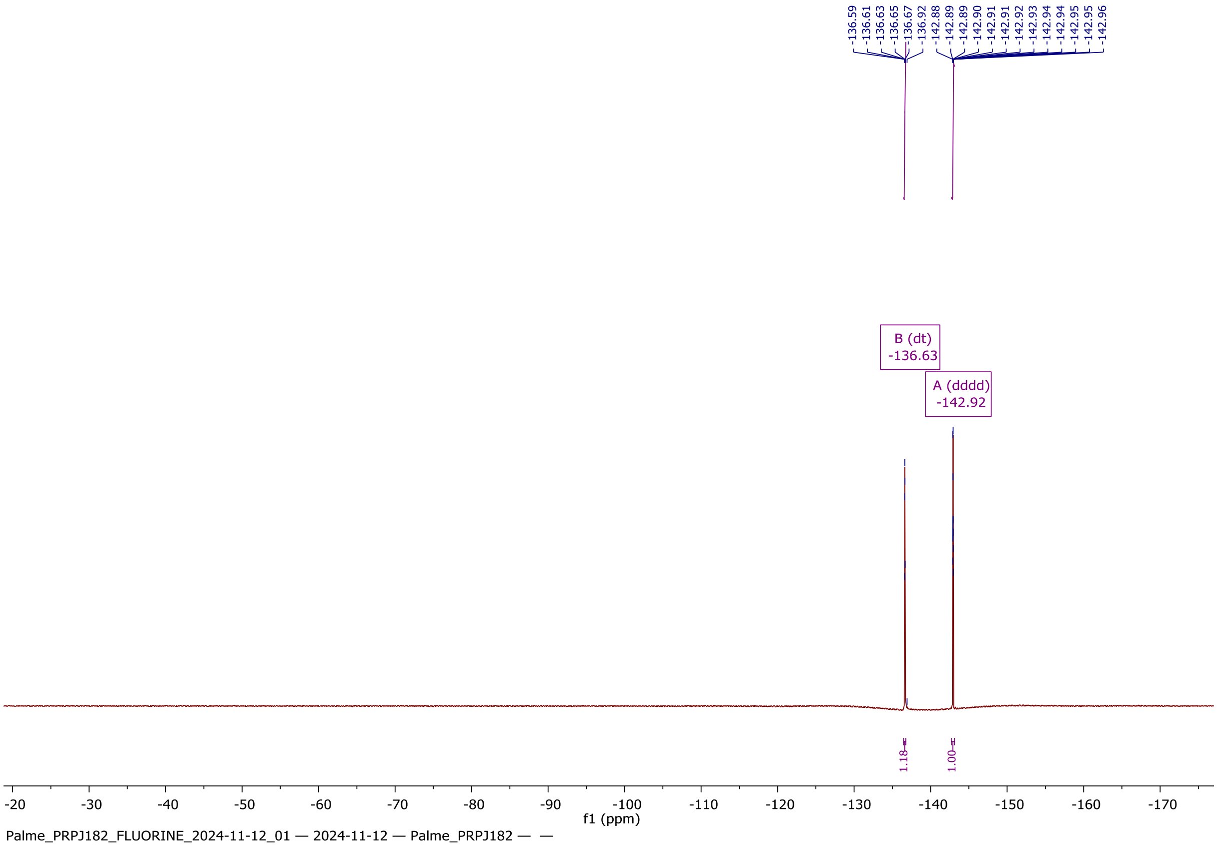
-136.59
-136.61
-136.63
-136.65
-136.67
-136.92
-142.88
-142.89
-142.89
-142.90
-142.91
-142.91
-142.92
-142.92
-142.93
-142.94
-142.94
-142.95
-142.95
-142.96
B (dt)
-136.63
A (dddd)
-142.92
1.18
1.00
-20 -30 -40 -50 -60 -70 -80 -90 -100 -110 -120 -130 -140 -150 -160 -170
f1 (ppm)
Palme_PRPJ182_FLUORINE_2024-11-12_01 — 2024-11-12 — Palme_PRPJ182 — —

6. HPLC chromatograms. (Note: For a Supplement or for the reviewers.)

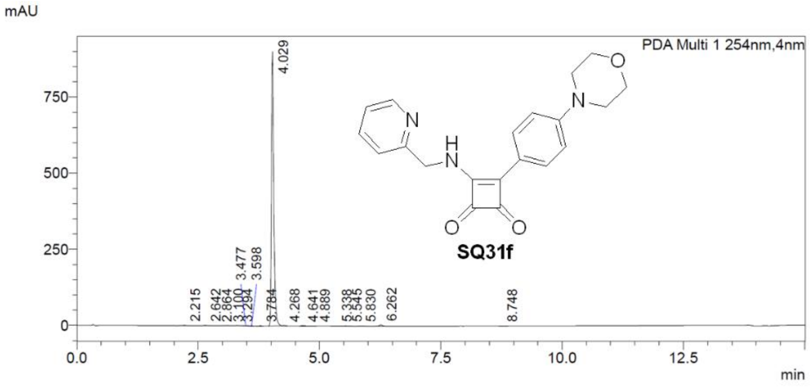

PDA Ch1 254nm

Peak#	Ret. Time	Area	Height	Area%
1	2.215	6826	1197	0.225
2	2.642	5772	994	0.190
3	2.864	1016	246	0.033
4	3.100	5175	889	0.171
5	3.294	3263	964	0.108
6	3.477	1714	420	0.056
7	3.598	202	103	0.007
8	3.784	1065	253	0.035
9	4.029	2977656	900606	98.145
10	4.268	1899	786	0.063
11	4.641	5402	1148	0.178
12	4.889	222	94	0.007
13	5.338	883	225	0.029
14	5.545	3803	754	0.125
15	5.830	1896	477	0.063
16	6.262	16654	4118	0.549
17	8.748	493	125	0.016
Total		3033939	913397	100.000

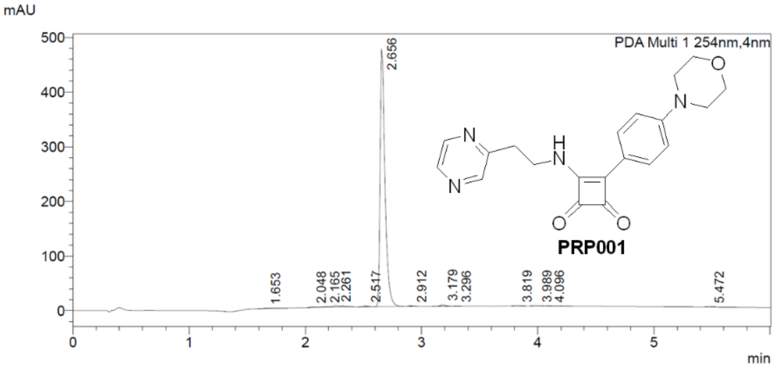

PDA Ch1 254 nm

Peak#	Ret. Time	Area	Height	Area%
1	1.653	2236	485	0.158
2	2.048	3225	1067	0.227
3	2.165	2066	909	0.146
4	2.261	13804	2740	0.972
5	2.517	4595	1744	0.324
6	2.656	1374895	471838	96.859
7	2.912	2791	1098	0.197
8	3.179	8456	2812	0.596
9	3.296	877	400	0.062
10	3.819	1420	468	0.100
11	3.989	1558	460	0.110
12	4.096	2831	519	0.199
13	5.472	722	270	0.051
Total		1419475	484810	100.000

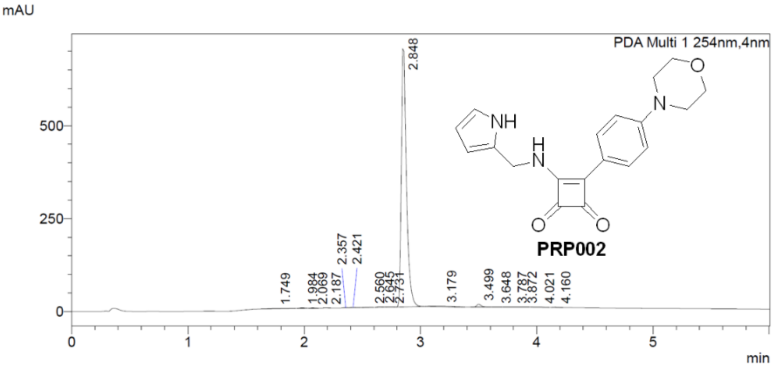

PDA Ch1 254 nm

Peak#	Ret. Time	Area	Height	Area%
1	1.749	4376	503	0.201
2	1.984	3279	1385	0.151
3	2.069	1838	816	0.085
4	2.187	1034	568	0.048
5	2.357	672	364	0.031
6	2.421	2311	843	0.106
7	2.560	652	174	0.030
8	2.645	2615	1115	0.120
9	2.731	1207	499	0.056
10	2.848	2110186	694482	97.138
11	3.179	17522	1441	0.807
12	3.499	22142	7560	1.019
13	3.648	763	322	0.035
14	3.787	459	127	0.021
15	3.872	563	168	0.026
16	4.021	1243	251	0.057
17	4.160	1491	597	0.069
Total		2172354	711216	100.000

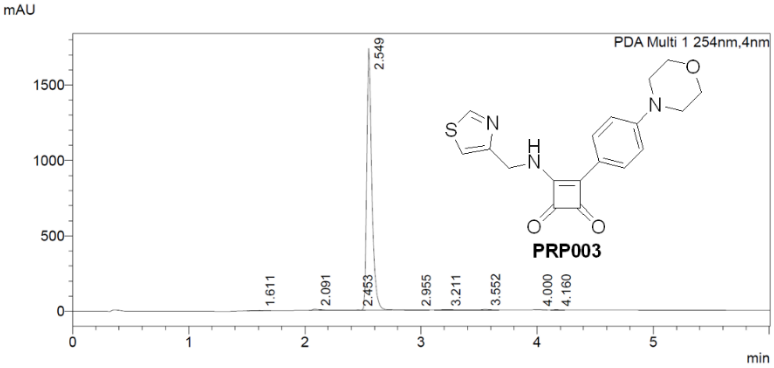

PDA Ch1 254 nm

Peak#	Ret. Time	Area	Height	Area%
1	1.611	4833	1243	0.091
2	2.091	30278	8041	0.569
3	2.453	1318	524	0.025
4	2.549	5252935	1735190	98.782
5	2.955	3512	656	0.066
6	3.211	10707	909	0.201
7	3.552	11829	4152	0.222
8	4.000	640	181	0.012
9	4.160	1650	629	0.031
Total		5317703	1751525	100.000

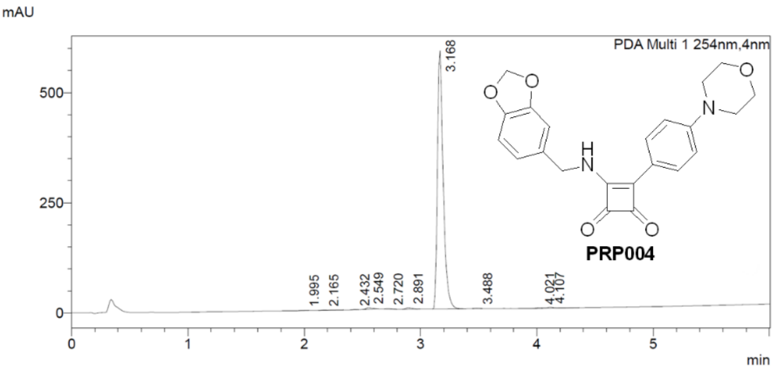

PDA Ch1 254 nm

Peak#	Ret. Time	Area	Height	Area%
1	1.995	1419	500	0.069
2	2.165	3175	501	0.154
3	2.432	1303	618	0.063
4	2.549	13274	3645	0.643
5	2.720	3426	1009	0.166
6	2.891	11763	3173	0.570
7	3.168	2013744	586236	97.569
8	3.488	1974	731	0.096
9	4.021	2922	777	0.142
10	4.107	10920	2063	0.529
Total		2063919	599253	100.000

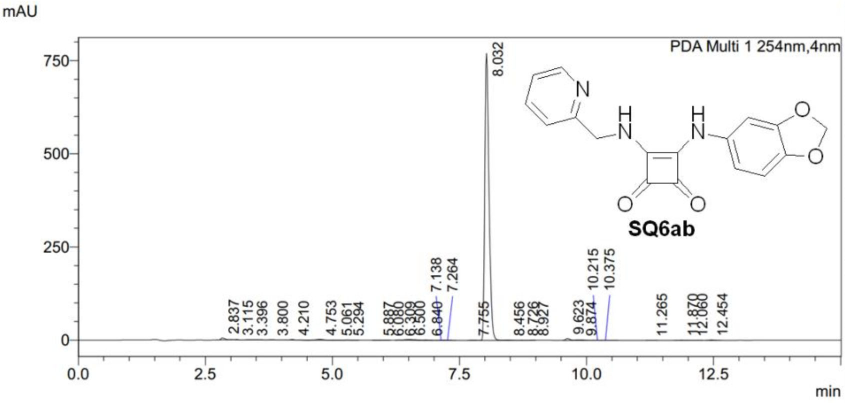

PDA Ch1 254nm

Peak#	Ret. Time	Area	Height	Area%
1	2.837	28816	5041	0.626
2	3.115	2994	1276	0.065
3	3.396	12383	555	0.269
4	3.800	8792	641	0.191
5	4.210	11085	1372	0.241
6	4.753	20905	2142	0.454
7	5.061	868	158	0.019
8	5.294	3032	542	0.066
9	5.887	1138	245	0.025
10	6.080	1343	107	0.029
11	6.309	3779	831	0.082
12	6.500	32141	1971	0.698
13	6.840	4722	735	0.103
14	7.138	1323	262	0.029
15	7.264	2154	268	0.047
16	7.755	4841	765	0.105
17	8.032	4402791	769491	95.622
18	8.456	1238	274	0.027
19	8.726	1719	260	0.037
20	8.927	1405	318	0.031
21	9.623	27127	4764	0.589
22	9.874	14408	1347	0.313
23	10.215	1381	185	0.030
24	10.375	2656	319	0.058
25	11.265	1101	186	0.024
26	11.870	2274	335	0.049
27	12.060	1932	237	0.042
28	12.454	6044	992	0.131
Total		4604391	795619	100.000

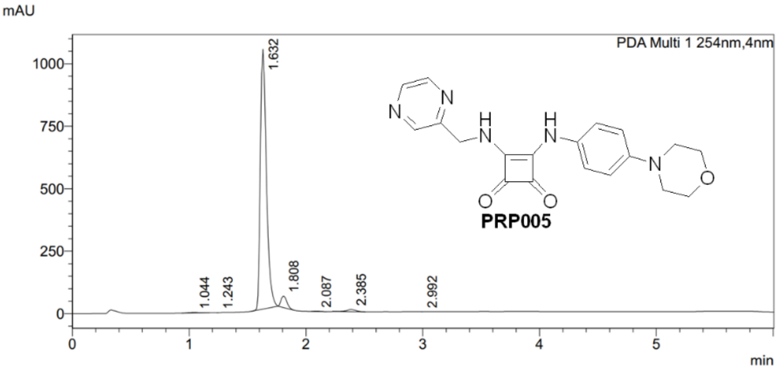

PDA Ch1 254nm

Peak#	Ret. Time	Area	Height	Area%
1	1.044	12448	1556	0.311
2	1.243	14	-22	0.000
3	1.632	3777638	1039374	94.467
4	1.808	158590	45398	3.966
5	2.087	2513	507	0.063
6	2.385	47676	8550	1.192
7	2.992	36	-57	0.001
Total		3998915	1095307	100.000

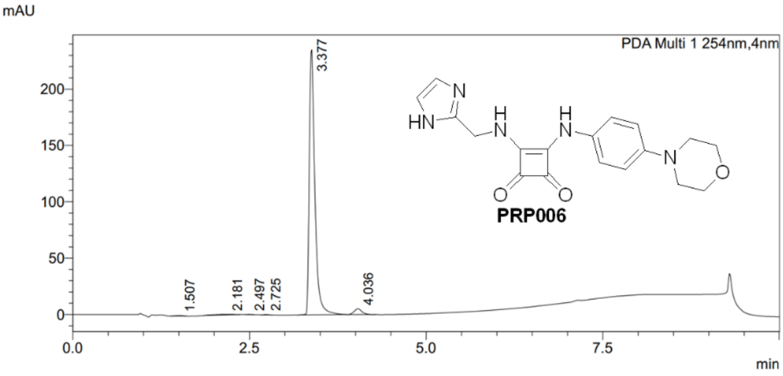

PDA Ch1 254nm

Peak#	Ret. Time	Area	Height	Area%
1	1.507	4841	665	0.358
2	2.181	12890	571	0.953
3	2.497	952	278	0.070
4	2.725	1482	304	0.110
5	3.377	1293609	234981	95.666
6	4.036	38438	5092	2.843
Total		1352212	241891	100.000

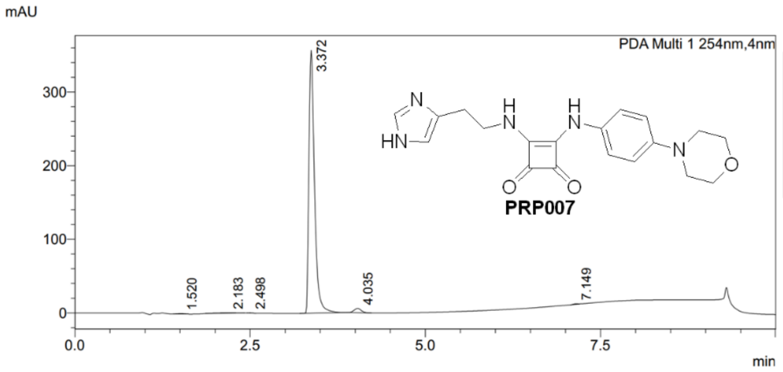

PDA Ch1 254nm

Peak#	Ret. Time	Area	Height	Area%
1	1.520	4378	610	0.213
2	2.183	12886	600	0.628
3	2.498	815	243	0.040
4	3.372	1989716	356718	96.941
5	4.035	41131	5775	2.004
6	7.149	3586	646	0.175
Total		2052512	364592	100.000

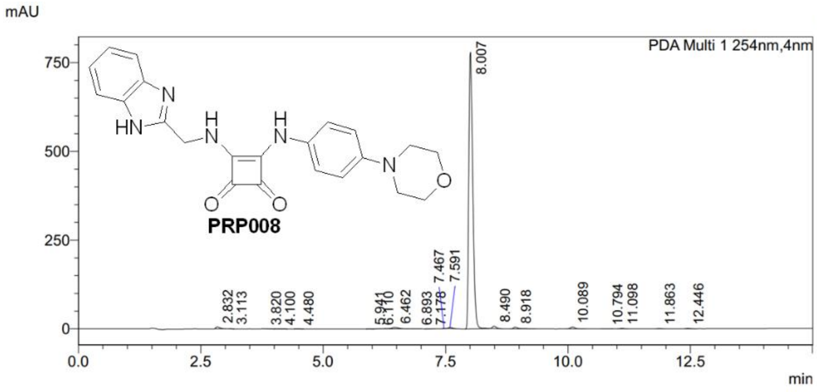

PDA Ch1 254nm

Peak#	Ret. Time	Area	Height	Area%
1	2.832	30023	5135	0.658
2	3.113	2633	1142	0.058
3	3.820	2160	380	0.047
4	4.100	2611	318	0.057
5	4.480	3179	645	0.070
6	5.941	1352	276	0.030
7	6.110	1001	189	0.022
8	6.462	41363	3804	0.906
9	6.893	1009	173	0.022
10	7.178	3340	449	0.073
11	7.467	2220	690	0.049
12	7.591	17679	3186	0.387
13	8.007	4333732	779010	94.937
14	8.490	38498	7108	0.843
15	8.918	36554	4780	0.801
16	10.089	32432	4990	0.710
17	10.794	1347	206	0.030
18	11.098	6530	1154	0.143
19	11.863	2797	528	0.061
20	12.446	4408	831	0.097
Total		4564869	814995	100.000

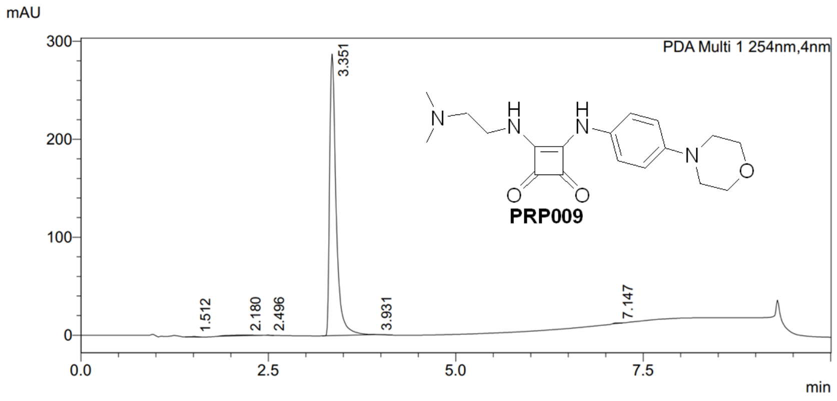

PDA Ch1 254nm

Peak#	Ret. Time	Area	Height	Area%
1	1.512	3667	530	0.208
2	2.180	19685	769	1.117
3	2.496	334	144	0.019
4	3.351	1735256	287536	98.432
5	3.931	1282	14	0.073
6	7.147	2673	543	0.152
Total		1762897	289536	100.000

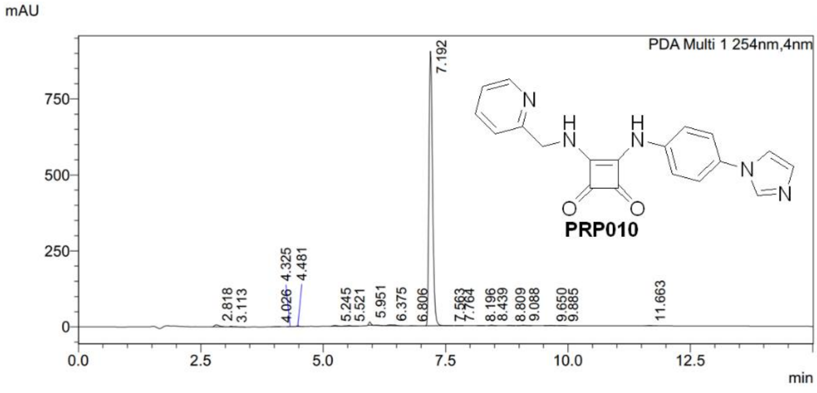

PDA Ch1 254nm

Peak#	Ret. Time	Area	Height	Area%
1	2.818	47233	6405	0.989
2	3.113	14433	2254	0.302
3	4.026	1683	439	0.035
4	4.325	1160	261	0.024
5	4.481	7281	2161	0.152
6	5.245	14424	2360	0.302
7	5.521	13464	2156	0.282
8	5.951	41380	11027	0.866
9	6.375	30416	2784	0.637
10	6.806	1644	408	0.034
11	7.192	4569060	902951	95.629
12	7.563	717	211	0.015
13	7.764	2440	523	0.051
14	8.196	764	217	0.016
15	8.439	7039	1611	0.147
16	8.809	2160	462	0.045
17	9.088	8110	1194	0.170
18	9.650	5083	1107	0.106
19	9.885	3220	627	0.067
20	11.663	6215	1023	0.130
Total		4777926	940179	100.000

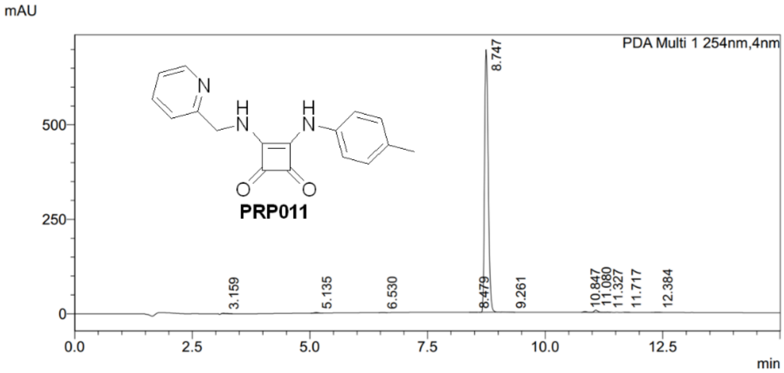

PDA Ch1 254nm

Peak#	Ret. Time	Area	Height	Area%
1	3.159	8149	1048	0.212
2	5.135	9964	1767	0.259
3	6.530	3061	645	0.080
4	8.479	1174	242	0.030
5	8.747	3773262	695105	98.064
6	9.261	2455	345	0.064
7	10.847	10651	2076	0.277
8	11.080	32079	5705	0.834
9	11.327	1869	426	0.049
10	11.717	1083	282	0.028
11	12.384	4026	648	0.105
Total		3847772	708290	100.000

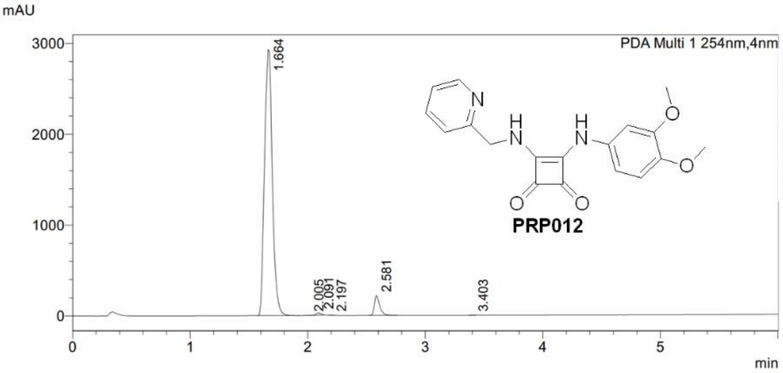

PDA Ch1 254nm

Peak#	Ret. Time	Area	Height	Area%
1	1.664	13516993	2926780	94.615
2	2.005	3931	1701	0.028
3	2.091	72044	26254	0.504
4	2.197	-838	-470	-0.006
5	2.581	692559	217624	4.848
6	3.403	1647	785	0.012
Total		14286336	3172674	100.000

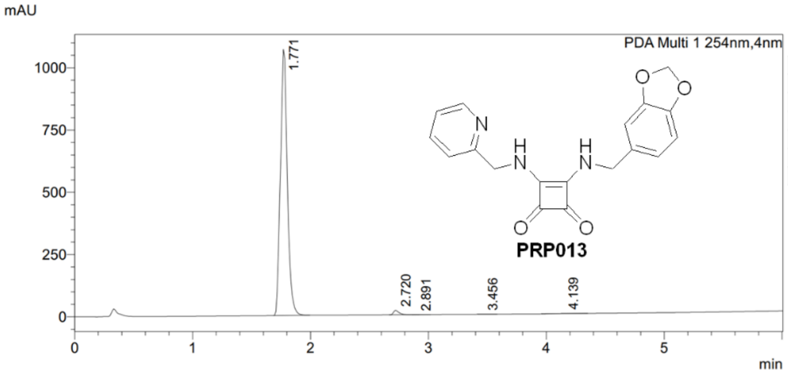

PDA Ch1 254nm

Peak#	Ret. Time	Area	Height	Area%
1	1.771	4356549	1068413	98.375
2	2.720	61127	17997	1.380
3	2.891	431	202	0.010
4	3.456	1944	565	0.044
5	4.139	8476	668	0.191
Total		4428527	1087846	100.000

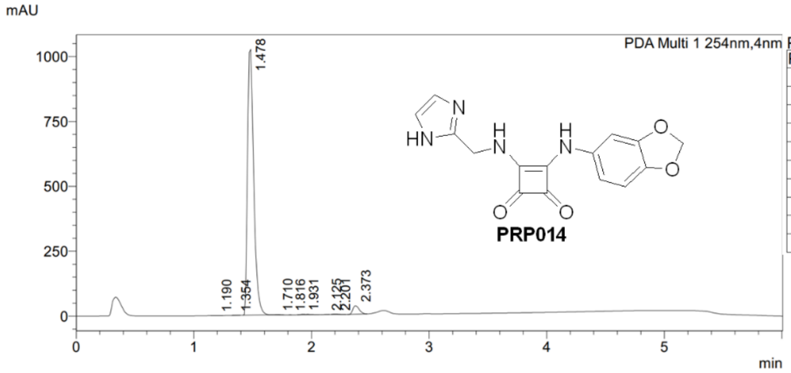

PDA Ch1 254nm

Peak#	Ret. Time	Area	Height	Area%
1	1.190	1338	329	0.034
2	1.354	696	243	0.018
3	1.478	3760619	1022886	96.177
4	1.710	3267	1008	0.084
5	1.816	281	97	0.007
6	1.931	13400	2167	0.343
7	2.125	1448	452	0.037
8	2.201	6426	1835	0.164
9	2.373	122618	32423	3.136
Total		3910093	1061441	100.000

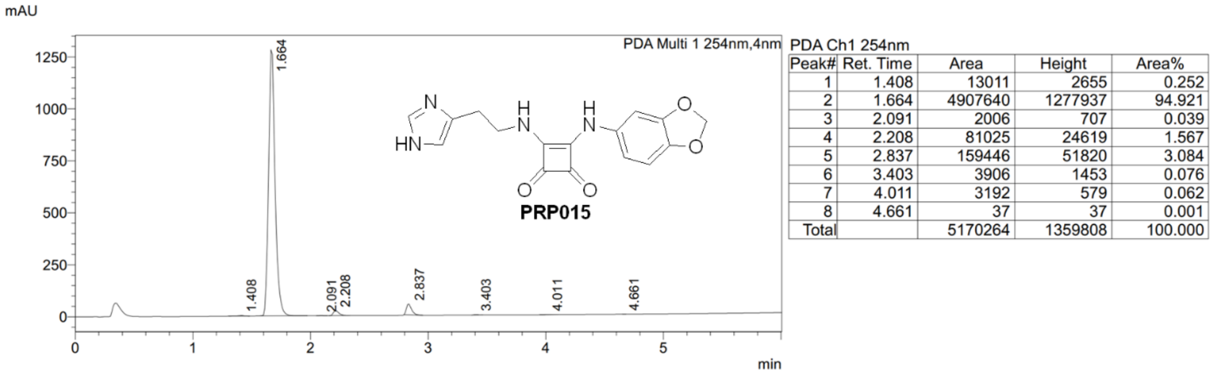

PDA Ch1 254nm

Peak#	Ret. Time	Area	Height	Area%
1	1.408	13011	2655	0.252
2	1.664	4907640	1277937	94.921
3	2.091	2006	707	0.039
4	2.208	81025	24619	1.567
5	2.837	159446	51820	3.084
6	3.403	3906	1453	0.076
7	4.011	3192	579	0.062
8	4.661	37	37	0.001
Total		5170264	1359808	100.000

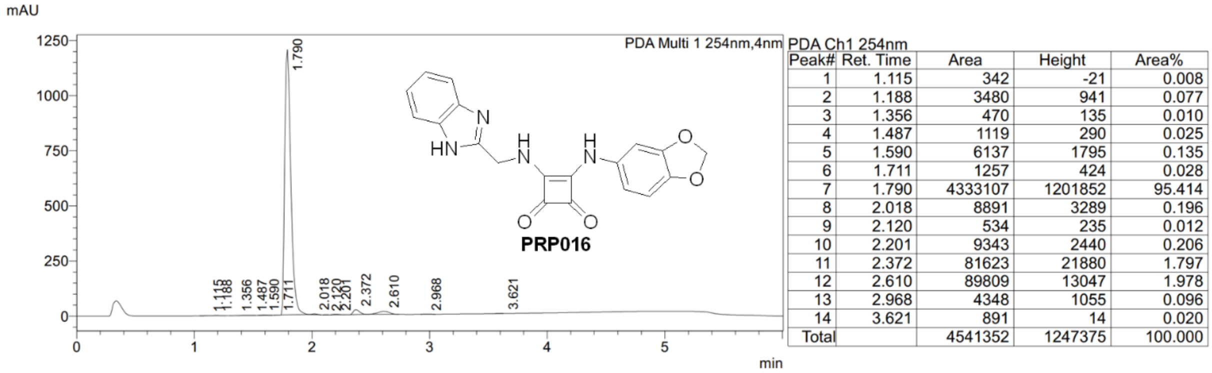

PDA Ch1 254nm

Peak#	Ret. Time	Area	Height	Area%
1	1.115	342	-21	0.008
2	1.188	3480	941	0.077
3	1.356	470	135	0.010
4	1.487	1119	290	0.025
5	1.590	6137	1795	0.135
6	1.711	1257	424	0.028
7	1.790	4333107	1201852	95.414
8	2.018	8891	3289	0.196
9	2.120	534	235	0.012
10	2.201	9343	2440	0.206
11	2.372	81623	21880	1.797
12	2.610	89809	13047	1.978
13	2.968	4348	1055	0.096
14	3.621	891	14	0.020
Total		4541352	1247375	100.000

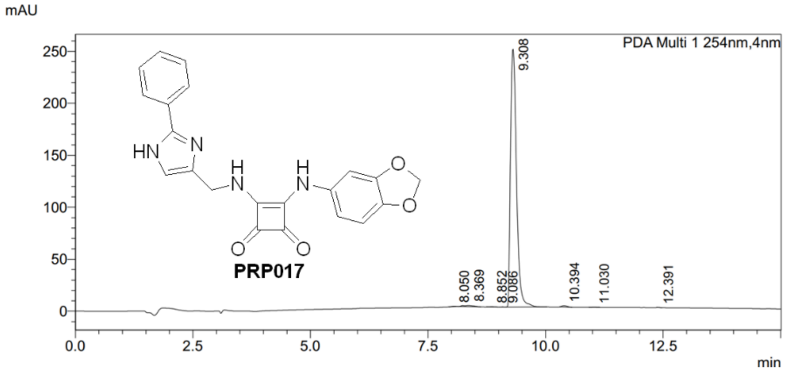

PDA Ch1 254nm

Peak#	Ret. Time	Area	Height	Area%
1	8.050	3145	455	0.141
2	8.369	19033	1123	0.852
3	8.852	1888	218	0.084
4	9.086	568	126	0.025
5	9.308	2198886	247994	98.400
6	10.394	9210	1144	0.412
7	11.030	1541	274	0.069
8	12.391	365	110	0.016
Total		2234636	251445	100.000

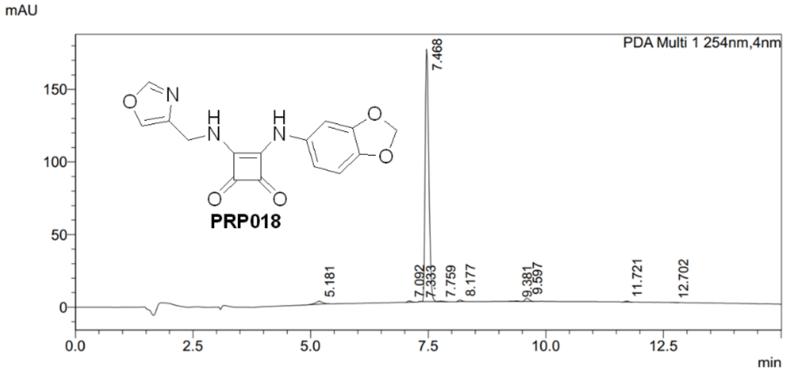

PDA Ch1 254nm

Peak#	Ret. Time	Area	Height	Area%
1	5.181	21906	2113	2.220
2	7.092	4945	895	0.501
3	7.333	534	117	0.054
4	7.468	933833	173758	94.623
5	7.759	2500	363	0.253
6	8.177	4802	984	0.487
7	9.381	1955	244	0.198
8	9.597	13040	2303	1.321
9	11.721	2967	541	0.301
10	12.702	419	94	0.042
Total		986902	181410	100.000

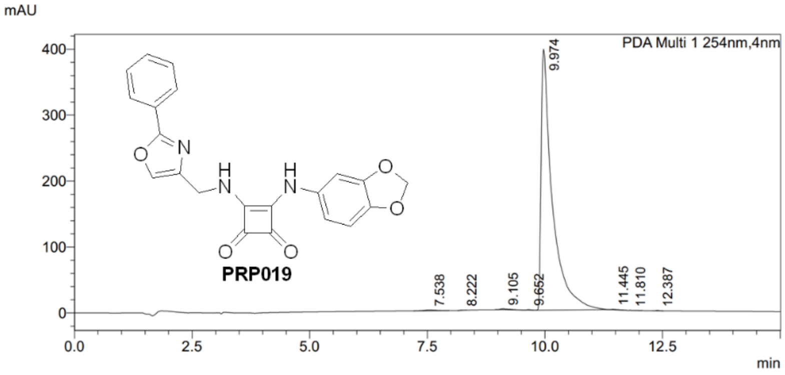

PDA Ch1 254nm

Peak#	Ret. Time	Area	Height	Area%
1	7.538	23775	1197	0.358
2	8.222	2644	537	0.040
3	9.105	22507	1452	0.339
4	9.652	4013	775	0.060
5	9.974	6574968	395607	99.006
6	11.445	7074	676	0.107
7	11.810	2128	147	0.032
8	12.387	3854	727	0.058
Total		6640963	401117	100.000

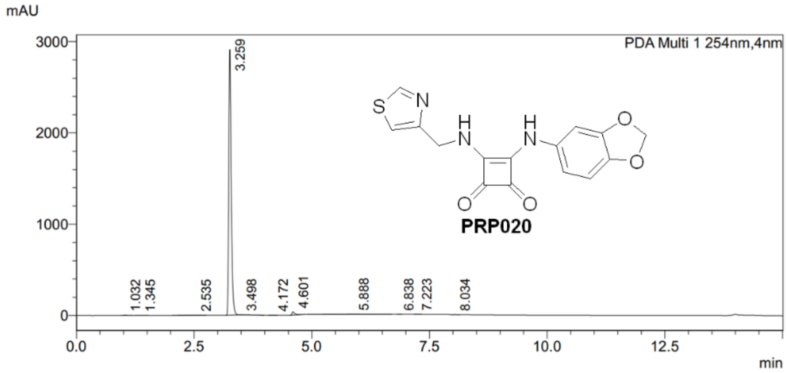

PDA Ch1 254nm

Peak#	Ret. Time	Area	Height	Area%
1	1.032	16636	2873	0.154
2	1.345	2516	430	0.023
3	2.535	32114	1377	0.298
4	3.259	10388470	2904059	96.477
5	3.498	9792	66	0.091
6	4.172	2744	836	0.025
7	4.601	164794	35681	1.530
8	5.888	145804	2381	1.354
9	6.838	446	197	0.004
10	7.223	1969	633	0.018
11	8.034	2485	741	0.023
Total		10767771	2949274	100.000

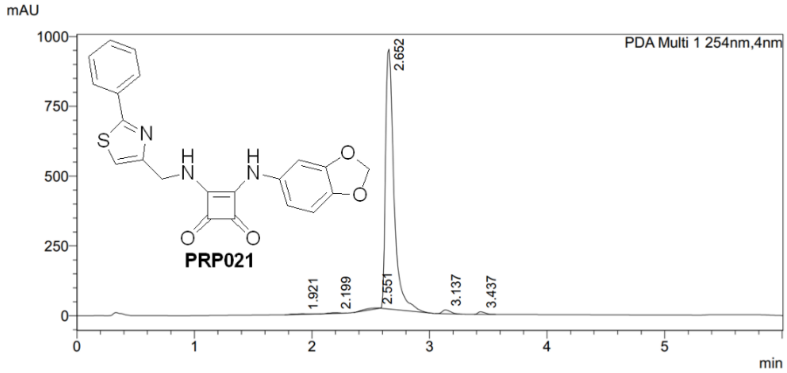

PDA Ch1 254nm

Peak#	Ret. Time	Area	Height	Area%
1	1.921	25591	2475	0.526
2	2.199	20339	2986	0.418
3	2.551	47502	3059	0.977
4	2.652	4669193	930100	95.991
5	3.137	64791	13354	1.332
6	3.437	36778	9126	0.756
Total		4864194	961100	100.000

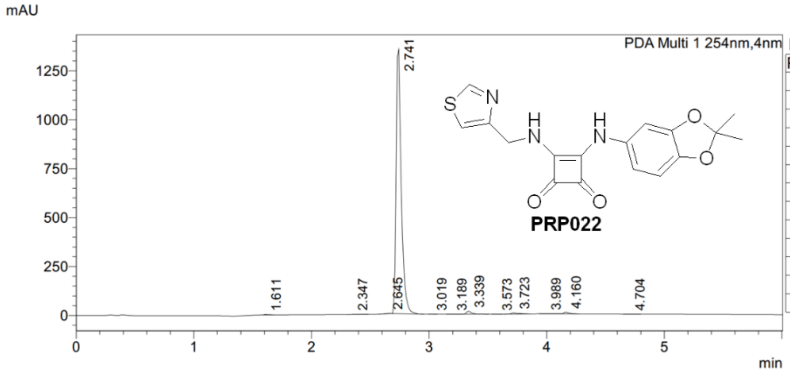

PDA Ch1 254nm

Peak#	Ret. Time	Area	Height	Area%
1	1.611	4455	1968	0.106
2	2.347	1318	252	0.031
3	2.645	3058	1228	0.073
4	2.741	4087965	1348727	97.381
5	3.019	1082	516	0.026
6	3.189	1705	610	0.041
7	3.339	45200	14356	1.077
8	3.573	614	217	0.015
9	3.723	24249	5794	0.578
10	3.989	2559	534	0.061
11	4.160	25016	8752	0.596
12	4.704	680	137	0.016
Total		4197900	1383091	100.000

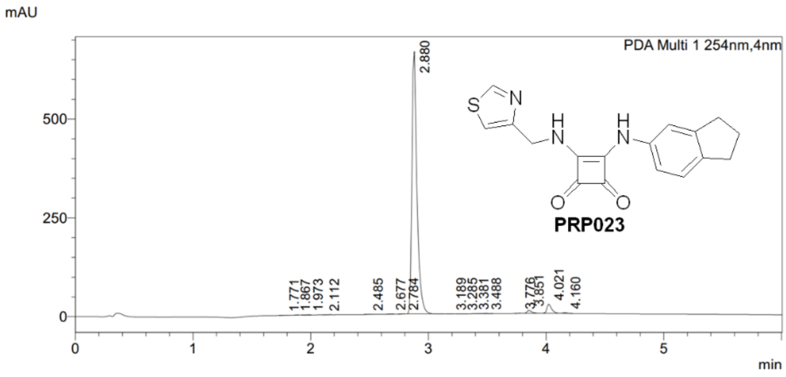

PDA Ch1 254nm

Peak#	Ret. Time	Area	Height	Area%
1	1.771	921	246	0.045
2	1.867	563	303	0.027
3	1.973	599	291	0.029
4	2.112	215	96	0.010
5	2.485	1406	579	0.068
6	2.677	917	379	0.044
7	2.784	503	260	0.024
8	2.880	1969801	663811	95.214
9	3.189	228	107	0.011
10	3.285	449	145	0.022
11	3.381	1254	434	0.061
12	3.488	1308	533	0.063
13	3.776	1138	636	0.055
14	3.851	21225	7555	1.026
15	4.021	63718	23154	3.080
16	4.160	4565	1896	0.221
Total		2068811	700424	100.000

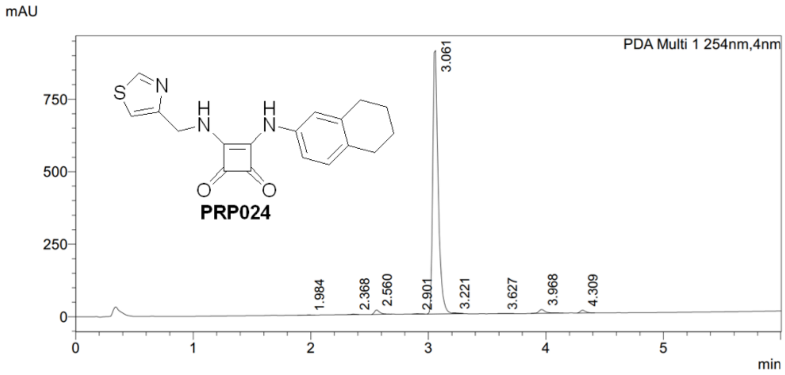

PDA Ch1 254nm

Peak#	Ret. Time	Area	Height	Area%
1	1.984	2433	587	0.079
2	2.368	4986	1634	0.162
3	2.560	46661	15291	1.517
4	2.901	4630	1315	0.151
5	3.061	2923205	907661	95.066
6	3.221	3415	1184	0.111
7	3.627	6850	1039	0.223
8	3.968	52539	13145	1.709
9	4.309	30206	9999	0.982
Total		3074926	951855	100.000

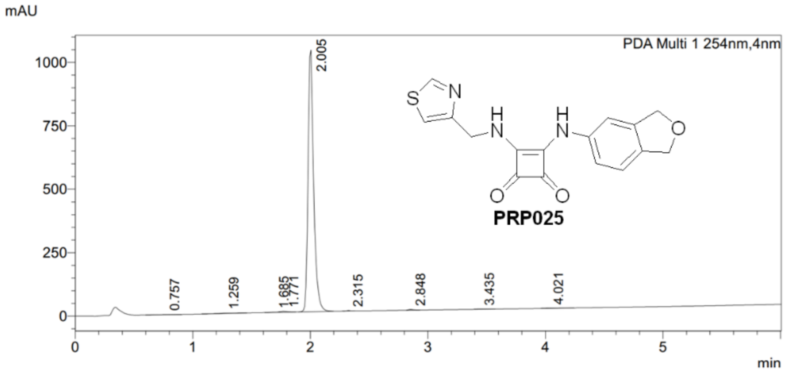

PDA Ch1 254nm

Peak#	Ret. Time	Area	Height	Area%
1	0.757	3430	291	0.099
2	1.259	8820	1101	0.254
3	1.685	2721	953	0.078
4	1.771	17141	3674	0.494
5	2.005	3414164	1030391	98.376
6	2.315	5460	1978	0.157
7	2.848	8064	2897	0.232
8	3.435	3711	1134	0.107
9	4.021	7002	724	0.202
Total		3470512	1043144	100.000

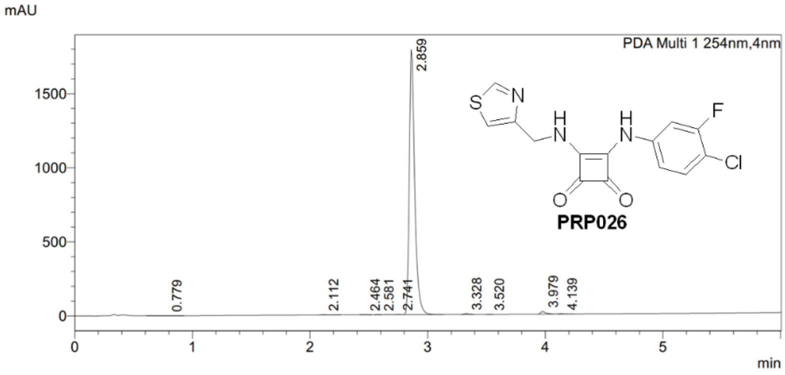

PDA Ch1 254nm

Peak#	Ret. Time	Area	Height	Area%
1	0.779	5000	362	0.087
2	2.112	3096	869	0.054
3	2.464	3145	1112	0.055
4	2.581	777	371	0.013
5	2.741	4466	1409	0.077
6	2.859	5678776	1787108	98.432
7	3.328	14370	5445	0.249
8	3.520	503	286	0.009
9	3.979	57953	17801	1.005
10	4.139	1180	404	0.020
Total		5769266	1815165	100.000

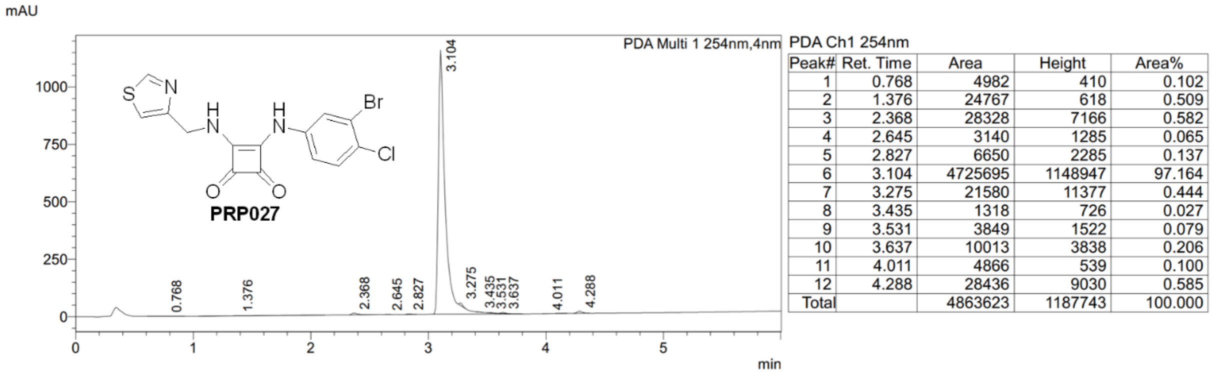

PDA Ch1 254nm

Peak#	Ret. Time	Area	Height	Area%
1	0.768	4982	410	0.102
2	1.376	24767	618	0.509
3	2.368	28328	7166	0.582
4	2.645	3140	1285	0.065
5	2.827	6650	2285	0.137
6	3.104	4725695	1148947	97.164
7	3.275	21580	11377	0.444
8	3.435	1318	726	0.027
9	3.531	3849	1522	0.079
10	3.637	10013	3838	0.206
11	4.011	4866	539	0.100
12	4.288	28436	9030	0.585
Total		4863623	1187743	100.000

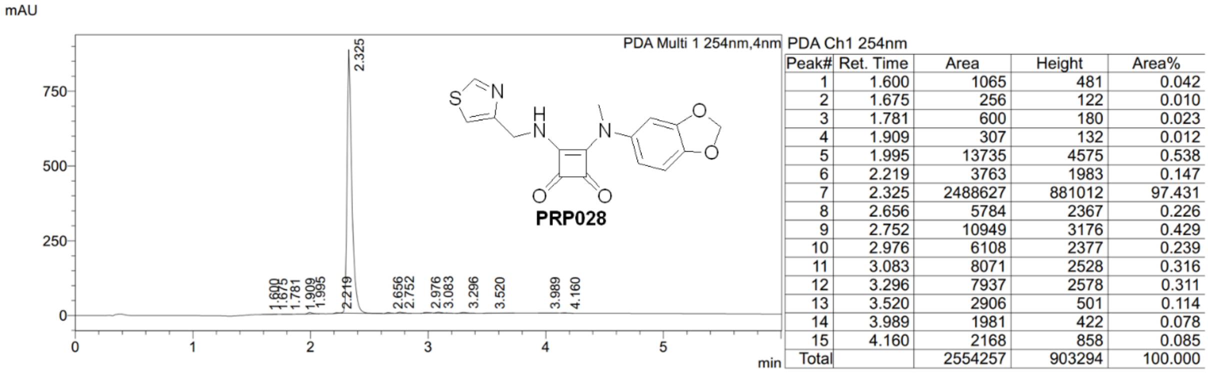

PDA Ch1 254nm

Peak#	Ret. Time	Area	Height	Area%
1	1.600	1065	481	0.042
2	1.675	256	122	0.010
3	1.781	600	180	0.023
4	1.909	307	132	0.012
5	1.995	13735	4575	0.538
6	2.219	3763	1983	0.147
7	2.325	2488627	881012	97.431
8	2.656	5784	2367	0.226
9	2.752	10949	3176	0.429
10	2.976	6108	2377	0.239
11	3.083	8071	2528	0.316
12	3.296	7937	2578	0.311
13	3.520	2906	501	0.114
14	3.989	1981	422	0.078
15	4.160	2168	858	0.085
Total		2554257	903294	100.000

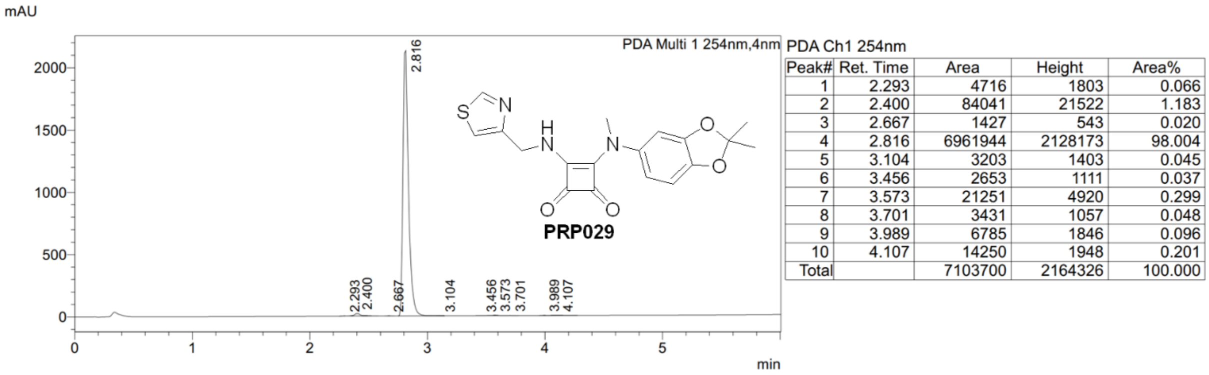

PDA Ch1 254nm

Peak#	Ret. Time	Area	Height	Area%
1	2.293	4716	1803	0.066
2	2.400	84041	21522	1.183
3	2.667	1427	543	0.020
4	2.816	6961944	2128173	98.004
5	3.104	3203	1403	0.045
6	3.456	2653	1111	0.037
7	3.573	21251	4920	0.299
8	3.701	3431	1057	0.048
9	3.989	6785	1846	0.096
10	4.107	14250	1948	0.201
Total		7103700	2164326	100.000

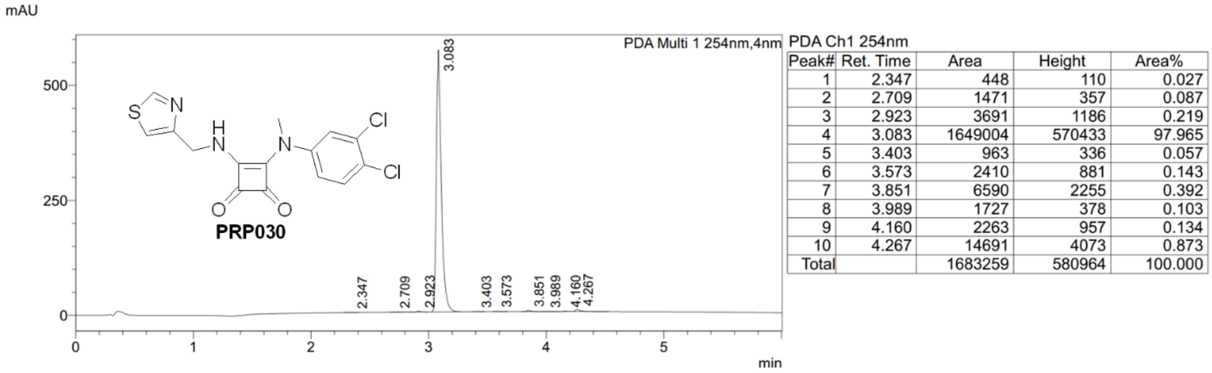

PDA Ch1 254nm

Peak#	Ret. Time	Area	Height	Area%
1	2.347	448	110	0.027
2	2.709	1471	357	0.087
3	2.923	3691	1186	0.219
4	3.083	1649004	570433	97.965
5	3.403	963	336	0.057
6	3.573	2410	881	0.143
7	3.851	6590	2255	0.392
8	3.989	1727	378	0.103
9	4.160	2263	957	0.134
10	4.267	14691	4073	0.873
Total		1683259	580964	100.000

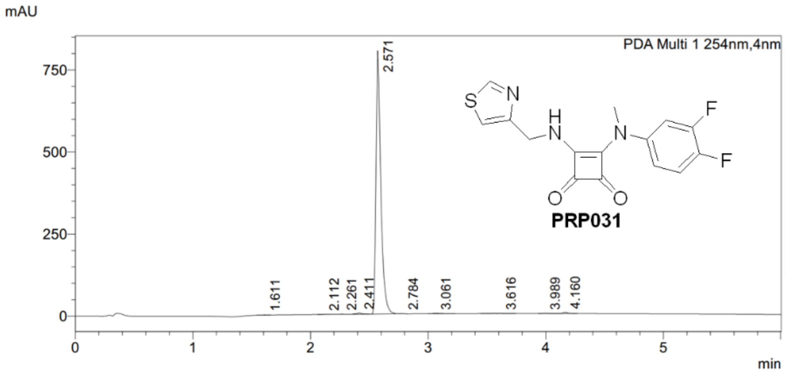

PDA Ch1 254nm

Peak#	Ret. Time	Area	Height	Area%
1	1.611	1308	389	0.056
2	2.112	767	213	0.033
3	2.261	935	371	0.040
4	2.411	12038	3538	0.517
5	2.571	2290491	802470	98.324
6	2.784	89	55	0.004
7	3.061	6981	1508	0.300
8	3.616	4972	574	0.213
9	3.989	2171	403	0.093
10	4.160	9771	3461	0.419
Total		2329524	812982	100.000
